# Comparison of polynomial fitting versus single time point analysis of ECIS data for barrier assessment

**DOI:** 10.14814/phy2.14983

**Published:** 2021-10-04

**Authors:** Karthik Suresh, Laura Servinsky, Laura Johnston, Naresh M. Punjabi, Steven M. Dudek, Mahendra Damarla

**Affiliations:** ^1^ Department of Medicine Johns Hopkins University School of Medicine Baltimore MD USA; ^2^ Department of Medicine University of Miami Coral Gables FL USA; ^3^ Department of Medicine University of Illinois Chicago IL USA

**Keywords:** barrier function, electrical cell‐substrate impedance sensing

## Abstract

Electrical cell‐substrate impedance sensing (ECIS) is an in vitro methodology for measuring the barrier integrity of a variety of cell types, including pulmonary endothelial cells. These experiments are frequently used for in vitro assessment of lung injury. The data derived from ECIS experiments consists of repeated measures of resistance across an endothelial monolayer. As such, these data reflect the dynamic changes in electrical resistance that occur over time. Currently methodologies for assessing ECIS data rely on single point assessments of barrier function, such as the maximal drop in trans‐endothelial electrical resistance (TER_Max_). However, this approach ignores the myriad of changes in resistance that occur before and after the TER_Max_ data point. Herein, we utilize polynomial curve fitting on experimentally generated ECIS data, thus allowing for comparing ECIS experiments by examining the mean polynomial coefficients between groups. We show that polynomial curves accurately fit a variety of ECIS data, and that concordance between TER_Max_ and coefficient analysis varies by type of stimulus, suggesting that TER_Max_ differences may not always correlate with a significant difference in the overall shape of the ECIS profile. Lastly, we identify factors that impact coefficient values obtained in our analyses, including the length of time devoted to baseline measurements before addition of stimuli. Polynomial coefficient analysis is another tool that can be used for more comprehensive interrogation of ECIS data to better understand the biological underpinnings that lead to changes in barrier dysfunction *in vitro*.

## Graphical abstract

Electrical cell‐substrate impedance sensing (ECIS) is a common method for assessing cell permeability. Herein, we use polynomial fitting to improve fitting and quantification of resistance measurements generated by ECIS experiments.

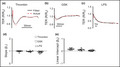

## 1 INTRODUCTION

In vivo quantification of the lung endothelial barrier function can be performed in preclinical models using a variety of well‐described methods (Matute‐Bello et al., 2008, 2011; Parker, 2011). However, while in vivo experiments are critical, it is often necessary to employ in vitro measurements of lung barrier function, in order to better dissect the cellular and molecular pathways involved. To that end, the m

easurement of barrier integrity of monolayers, particularly those composed of cultured endothelial cells, have become increasingly popular, with the rationale that these in vitro measurements serve as a proxy for loss of endothelial barrier integrity in vivo (Dudek & Garcia, 2001; Tiruppathi et al., 1992). Several techniques exist for such assessments, each with individual merits and criticisms. Electrical cell‐substrate impedance sensing (ECIS) is a powerful tool that permits investigators to analyze barrier function of a monolayer of cells in response to stimuli. Cells are grown on electrode coated cell cultureware and exposed to an electrical current. The electrical resistance across the monolayer represents the integrity of the monolayer (i.e., barrier function), with a drop in electrical resistance correlating to an increase in barrier permeability (Tiruppathi et al., 1992). A benefit of this technique is the frequent and repeated measurements of electrical resistance, allowing for assessment of rapid changes in barrier function. For instance, a stimulus that induces rapid disruption followed by recovery can be detected using ECIS; such dynamic changes in lung barrier function may be missed using methods that use a single time point assessment. Additionally, as electrical resistance is a continuous variable measured at discrete time points, ECIS allows for a more precise quantification of barrier disruption compared to methods that rely on transcellular passage of molecules of a certain molecular weight (e.g., dextrans, Martins‐Green et al., 2008). Because of these benefits, many laboratories use ECIS‐based methodology for detecting barrier function (Kawkitinarong et al., 2004; Knezevic et al., 2009; Michalick et al., 2020; Ourradi et al., 2017; Petrache et al., 2001; Suresh et al., 2019). However, the resulting experiments can lead to thousands of data points (depending on duration of experiment) and there is no consensus in how to interpret these complex data.

Recent publications have reported varied analytical approaches to these types of data (Kawkitinarong et al., 2004; Knezevic et al., 2009; Michalick et al., 2020; Ourradi et al., 2017; Suresh et al., 2019). For example, Ourradi et al measured barrier integrity on human pulmonary microvascular endothelial cells in response to VEGF using ECIS and statistical analyses included comparisons against control conditions at single time points using *t*‐tests (Ourradi et al., 2017). Other groups have statistically compared the maximum change in electrical resistance from baseline (Kawkitinarong et al., 2004; Suresh et al., 2019), also using a single time point. Distilling thousands of data points to a single value for analyses may be an oversimplification that, while making conclusions readily understandable, may lose valuable information. Here we describe a method that utilizes polynomial curve fitting to model ECIS data. We apply this method to a variety of ECIS data, and compare this method against commonly used single time point analyses.

## 2 METHODS

### 2.1 Reagents and tissues

Human lung microvascular endothelial cells (HLMVEC): Primary HLMVECs derived from individual donors (Lonza, Walkersville, MD or Cell Biologics) and maintained according to the manufacturer's recommendations. Cells were analyzed between passages 4 and 6, and were cultured in complete media with 10% FBS.

Mouse lung microvascular endothelial cells (MMVECs) were isolated using a dual‐selection magnetic bead‐based approach as previously described (Suresh et al., 2017). Briefly, peripheral lung digests were incubated with beads conjugated with CD31. Positively selected cells were grown to confluence prior to second selection with the microvascular specific marker *Griffonia simplicifolia* lectin. Cells were used at passages 4–6.

Pharmacological agonists: Caspase 3‐specific inhibition was achieved using DEVD (Cayman Chemical) at a concentration of 50 µg/ml (Han et al., 1997). After a 2‐h stabilization period in basal media, thrombin (Sigma, product #T4393) was added at a concentration of 1.25 U/ml. Dosing was based on our prior work and previously published data (Birukova et al., 2007; Chiang et al., 2009; Finigan et al., 2005; Suresh et al., 2019). Lipopolysaccharide (LPS, 0127:B8, product no. L3129; Sigma) was added at concentrations specified within each experiment (Damarla et al., 2014). We chose this particular strain of LPS, O127:B8, as it has been previously shown to induce septic shock in C57BL/6 mice when given parenterally (Damarla et al., 2014; Xu et al., 1994). The product number L3129 has a potency of >500,000 endotoxin units per mg and is obtained through phenol extraction for purification, which results in <3% impurity. For GSK1016790 (GSK) exposure, cells were treated with 1.5 µM GSK as previously described (Shen et al., 2019).

### 2.2 Culture conditions

A total of 20,000 HLMVECs or 25,000 MMVECs were plated on 0.1% gelatin‐coated gold‐plated electrode culture ware, and the following morning, culture media was changed to basal media (serum free for thrombin experiments; 5% FBS for LPS experiments; serum free for GSK experiments) to include pharmacological inhibitors. With this approach we have routinely seen electrical resistance of ~1300 ohms (Suresh et al., 2019). Furthermore, we have noted that the electrical resistance at the time of starting our experiments has plateaued. Additionally, in parallel experiments that were proportionately scaled based on cells per square centimeter, we have noted confluent cells and tight paracellular junctions as seen by phalloidin staining of the endothelial cell monolayers (Suresh et al., 2019). Given the appearance of the endothelial monolayers under our culture conditions as well as the stability of the electrical resistance and the relatively high electrical resistance observed (~1300 ohms) we predict endothelial cells within the ECIS array plates are likely at confluence or near confluence.

### 2.3 Endothelial barrier function

Agonist‐induced electrical resistance, as a marker of barrier integrity, was measured using an Electrical Cell‐substrate Impedance Sensing System (ECIS, Applied Biophysics Inc.), as previously described (Dudek et al., 2004). Our ECIS system is capable of analyzing the standard eight well array. We used the 8W10E+, which has 40 electrodes, for our assessments. According to the manufacturer's website (Applied BioPhysics), this array has the capability of measuring 2000–4000 cells by the electrodes. We have purposefully chosen this particular array for our experiments due to the large number of cells it is capable of simultaneously measuring. Furthermore, the 8W10E+ array offers the largest number of electrodes and the largest number of cells measureable within the eight well array systems designed for barrier function assessment. Pooled data from individual wells are shown as summation plots and the maximum drop in trans‐endothelial electrical resistance, TER_Max_, from individual wells is calculated.

### 2.4 Data analysis

Curve fitting analyses were performed in MATLAB. Best fit polynomials for normalized ECIS data were generated using the *polyfit*() function. A set of polynomial coefficients was generated for each experiment, and mean/SEM were calculated for each coefficient within an experimental group. The time variable was scaled from seconds to hours to improve polynomial fit. No additional centering was performed. A complete listing of the code and output is provided in the Data Supplement. A value of *p* < 0.05 was considered statistically significant.

## 3 RESULTS

### 3.1 Generation of a polynomial fitted curve

Since ECIS data can be measured continuously with sequential measurements, this can result in a substantially large data set. This data are often depicted as a *XY* plot showing the continuously measured electrical resistance, plotted on the *Y*‐axis, with time plotted on the *X*‐axis. While these curves are comprised of hundreds (if not thousands) of data points, statistical analyses often trim these large data to a comparison of a single value, at an arbitrary time point or the time point of TER_Max_. In order to incorporate the entirety of the ECIS curve and account for the repeated measurements of the data, we fit ECIS curves into a fifth‐order polynomial using the equation:

$$\text{TER}\left(t\right)=a_{0}+a_{1}t+a_{2}t^{2}+a_{3}t^{3}+a_{4}t^{4}+a_{5}t^{5},$$

where TER(*t*) represents the electrical resistance value at time *t* – TER(*t*) is calculated as a function of time and a series of coefficients a_0_ through a_5_. We chose a polynomial with an odd number of coefficients given that the TER profile for most agonists involves at least two inflections (initial barrier disruption/strengthening, followed by recovery/return to baseline). While increasing polynomial order (i.e., seventh or ninth order) continued to improve the goodness‐of‐fit (data not shown), we chose fifth‐order to strike a balance between improved fit (compared to third order) and using the lowest order polynomial needed to obtain accurate fit.

**FIGURE 1:**
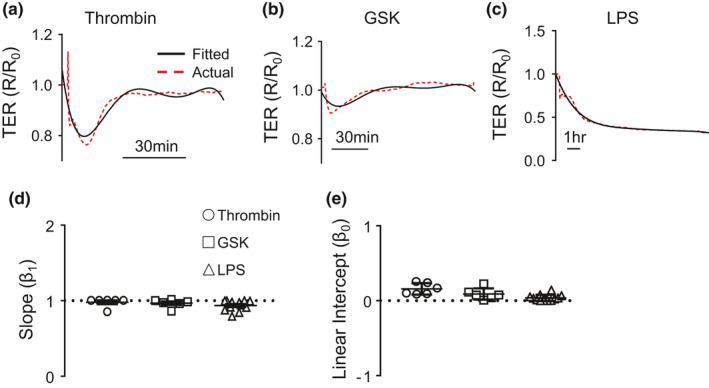
Goodness‐of‐fit of 5th order polynomials for a variety of barrier disrupting stimuli. (a–c) ECIS profiles of actual (red dashed lines) and fitted curves (black lines) for (a) thrombin, (b) GSK and (c) LPS, 100 µg/ml. (d) Scatter plots showing slope (β_1_; 1d) and linear intercept (β_0_; 1e) of observed TER values regressed against fitted values in MMVECs treated with thrombin (circles), GSK (squares) or LPS (triangles)

**FIGURE 2:**
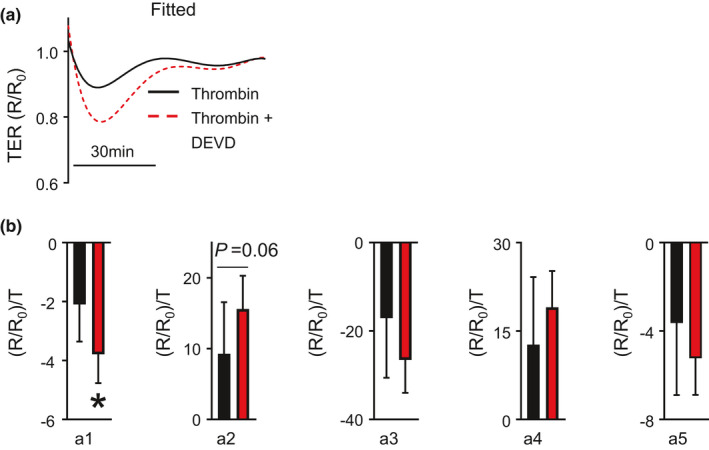
(a) Fitted TER curves for thrombin‐treated HMVECs with and without pre‐treatment with the caspase‐3 inhibitor DEVD). (b) Bar graphs showing mean ± SEM values for coefficients a_1_ – a_5_ in diluent (black) and DEVD (red) treated HMVECs following thrombin exposure. *p < 0.05

As ECIS can be used to test barrier integrity in either direction (i.e., loss or enhancement) using many stimuli, we first wanted to compare the polynomial fitting model to ECIS curves generated by a variety of stimuli. To determine the multifunctionality of the fitting, we chose stimuli with a variety of barrier effects: (1) thrombin–a rapid and transient drop in TER followed by rapid recovery, Figure 1a; (2) GSK–a rapid drop in TER followed by gradual recovery, Figure 1b; and (3) LPS (100 µg/ml)–a slow and persistent drop in TER as time progresses, Figure 1c. As shown in Figure 1a–c, fitted curves reasonably approximated ECIS curves following thrombin, GSK and LPS exposures. To quantify the goodness‐of‐fit, we regressed the observed values against the fitted values using the equation *Y* = *β*_1_*x + *β*_0_, where *β*_1_ equals the slope and *β*_0_ equals the linear intercept. With perfect concordance, the fitted values would exactly equal the observed values, resulting in *y* = 1*x + 0, i.e., *β*_1_ = 1 and a *β*_0_ = 0. The *β*_1_ for the fitted curves for thrombin, GSK, and LPS were 0.98 ± 0.06, 0.97 ± 0.05, and 0.94 ± 0.06 respectively (Figure 1d), while the *B*_0_ values were 0.15 ± 0.07, 0.09 ± 0.07, and 0.04 ± 0.04 for thrombin, GSK, and LPS exposures, respectively (Figure 1e).

### 3.2 Identification of coefficients using a polynomial fitted curve

Using the polynomial fitting model, five coefficients are identified, ***a*_1‐5_**, that shape the resultant curve. The sixth coefficient, *a*_0_, was, by definition, exactly the same across groups since ECIS data are normalized to a *t*_o_ time value. While these coefficients do not directly correlate with a specific portion of the ECIS curve, they do have distinct effects on the subsequent shape of the ECIS curve. To understand the role each coefficient plays on a fitted curve, we initially graphed a fitted curve for HLMVECs treated with thrombin and then manipulated each coefficient positively and negatively, while keeping the others fixed, and graphed the resultant curves. As shown in Figure S1A (http://doi.org/10.5281/zenodo.4542523), coefficient ***a*_1_** seems to have the most effect at the initial down sloping portion of the curve. The coefficients ***a*_2,_*a*_3_, *a*_4_, *a*_5_** affect the fitted curve as it progresses along the *X*‐axis seemingly at inflection points, as shown in Figure S1B–E. Identification/quantification of these coefficients allows for comparison between two fitted ECIS curves.

### 3.3 Comparing single point analyses to polynomial fitting: Thrombin

After having shown the relative effects of each coefficient of the polynomial equation on the resultant shape of the fitted curve, we sought to compare polynomial fitting method of analysis to single time point analyses. Thrombin exposure in HLMVECs results in a rapid drop in electrical resistance followed by a quick recovery to baseline. We have recently shown that caspase 3 inhibition, using DEVD, results in a significantly lower TER_Max_ compared to thrombin alone, demonstrating a worsened endothelial barrier function (Suresh et al., 2019). Thus, we generated polynomial fitted curves of HLMVECs exposed to thrombin and thrombin exposure following caspase 3 inhibition, Figure 2a. The fitted curves look similar to the plotted raw data (Suresh et al., 2019). Next, we compared the coefficients ***a*_1,_*a*_2,_*a*_3_, *a*_4_**, and ***a*_5_** identified using polynomial fitting for HMVECs exposed to 1.25 U/ml of thrombin and thrombin treated with DEVD. As shown in Figure 2b, we observed a significant difference in coefficient ***a*_1_** with caspase 3‐specific inhibition with DEVD compared with thrombin exposure alone. We also observed an increase in coefficient ***a*_2_** with caspase 3‐specific inhibition with DEVD compared with thrombin exposure alone that did not reach statistical significance (*p* = 0.06). The remainder of the coefficients were not significantly different.

### 3.4 Comparing single point analyses to polynomial fitting: GSK

**FIGURE 3:**
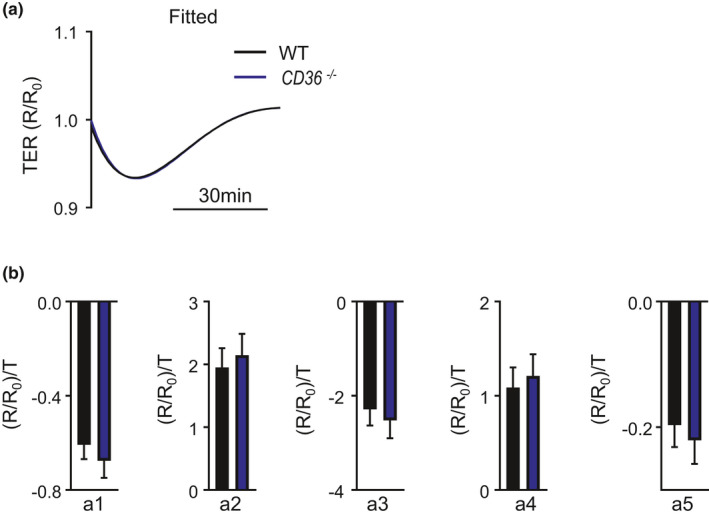
(a) Fitted TER curves for GSK‐treated WT and *CD36* ^−/−^ MMVECs. (b) Bar graphs showing mean ± SEM values for coefficients a_1_ – a_5_ for WT (black) and *CD36* ^−/−^ MMVECs following treatment with GSK

Following thrombin, we turned our attention to a different stimulus—GSK1016790 (GSK), a chemical agonist of the transient receptor potential vanilloid‐4 (TRPV4) channel (Shen et al., 2019). We sought to compare polynomial fitting to single point analyses following GSK stimulation, where the ECIS profile is characterized by a rapid drop in TER followed by gradual recovery. First to understand the role each coefficient plays on a fitted curve for MMVECs treated with GSK, we graphed a fitted curve for HMVECs treated with GSK and then manipulated each coefficient positively and negatively, while keeping the others fixed, and graphed the resultant curves. The coefficients ***a*_1,_*a*_2,_*a*_3_**, ***a*_4_**, ***a*_5_** affect the fitted curve as it progresses along the *X*‐axis seemingly at inflection points, similar to thrombin exposure (data not shown). We recently showed that in MMVECs isolated from WT or *CD36* ^−/−^ mice, GSK exposure results in a similar changes in TER_Max_, suggesting equivalent effects on endothelial barrier function (Suresh et al., 2017). Consistent with this, the generated polynomial fitted curves of MMVECs from WT or *CD36* ^−/−^ mice following GSK exposure essentially overlap (Figure 3a). To quantify the similarity between the two fitted curves, we compared the coefficients ***a*_1,_*a*_2,_*a*_3_**, ***a*_4_**, and ***a*_5_** identified using polynomial fitting for MMVECs from WT or *CD36* ^−/−^ mice exposed to GSK. As shown in Figure 3b, there are no differences in coefficients ***a*_1_** − ***a*_5_**.

### 3.5 Comparing single point analyses to polynomial fitting: LPS

**FIGURE 4:**
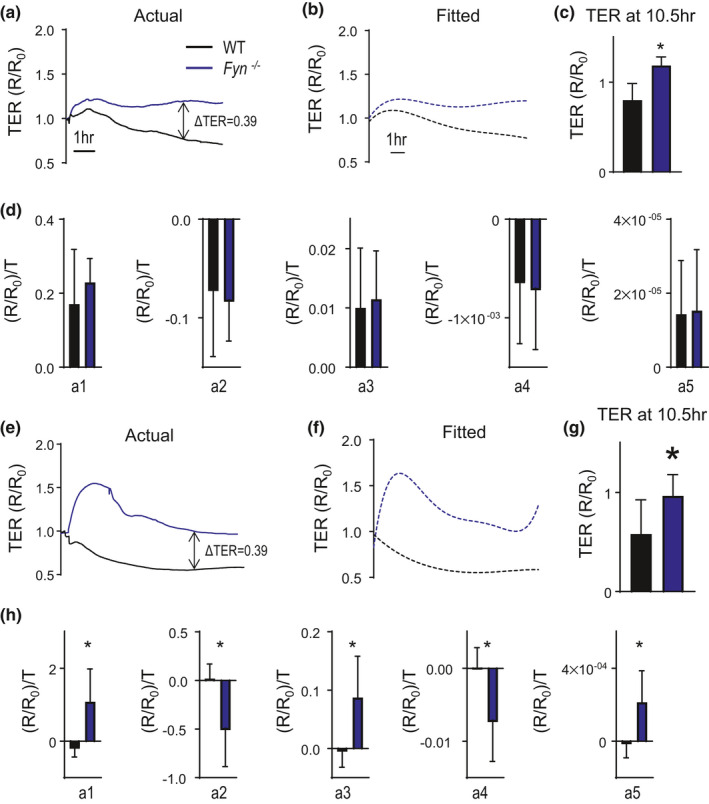
Evaluation of TER in LPS‐treated HLMVECs. Actual (a) and fitted (b) TER curves in HLMVECs following treatment with 50 µg/ml LPS. (c) Bar graphs showing mean+/− TER_Max_ at a fixed time point (10.5 h after LPS treatment) between diluent (black) and LPS (blue) treated HLMVECs. (c) Values of coefficients a_1_ − a_5_ in diluent (black) and 50 µg/ml LPS (blue) treated HLMVECs. (e–h) Actual (e), Fitted (f), TER_Max_ (g) and coefficients a_1_ − a_5_ (h) in diluent (black) and 100 µg/ml LPS (blue) treated HLMVECs. *p < 0.05

After having compared polynomial fitted curves to TER_Max_ following exposures that result in ECIS curves with a rapid and transient drop in TER followed by rapid or gradual recovery, we looked to compare polynomial fitting to single point analyses following LPS stimulation, where the drop in TER is slow and persistent as time increases (Leligdowicz et al., 2018). Given prior work implicating the src kinase Fyn on LPS‐induced MMVEC cytoskeletal rearrangement (Knezevic et al., 2009), we sought to determine the effect on Fyn loss on MMVEC barrier integrity following LPS exposure. To this end, we measured TER following LPS in MMVECs isolated from WT and *Fyn* ^−/−^ mice. We then plotted electrical resistances of wild type (WT) or *Fyn* ^−/−^ MMVECs exposed to LPS at two different doses. As shown in Figure 4a, using single time point analyses, at 10.5hrs after LPS exposure, there is a significant decrease (0.38) in TER in *Fyn* ^−/−^ MMVECs (compared to WT MMVECs) following exposure to 50 μg/ml of LPS. Additionally, exposure to 100 μg/ml of LPS also results in a similar difference in TER (0.39) at 10.5 h between WT and *Fyn* ^−/−^ MMVECs, shown in Figure 4e. We next generated polynomial fitted curves for each of these conditions (Figure 4b,f); these curves are similar to the original plotted curves (Figure 4a,e).

We next quantified the coefficients ***a*_1,_*a*_2,_*a*_3_, *a*_4_**, and ***a*_5_** identified using polynomial fitting for WT and *Fyn* ^−/−^ MMVECs exposed to 50 μg/ml of LPS. As shown in Figure 4c, there is a statistically significant difference in TER at 10.5 h between WT and *Fyn* ^−/−^ MMVECs using a *t*‐test, *p* < 0.05. However, there is no difference in any of the coefficients ***a*_1,_*a*_2,_*a*_3_, *a*_4_**, or ***a*_5_** between WT and *Fyn* ^−/−^ MMVECs exposed to 50 μg/ml of LPS (Figure 4D). This discrepancy between the TER_Max_ and the fitted polynomial coefficient results suggest that while there are significant differences in TER at single time points, when accounting for the entire time period, there does not appear be any statistical difference between the two sets of curves. Following exposure to 100 μg/ml of LPS, there is also a statistically significant difference in TER at 10.5 h between WT and *Fyn* ^−/−^ MMVECs using a *t*‐test, *p* < 0.05, Figure 4G. In contrast to exposure to 50 μg/ml of LPS, all the coefficients ***a*_1,_*a*_2,_*a*_3_, *a*_4_**, or ***a*_5_** are statistically significantly different between WT and *Fyn* ^−/−^ MMVECs; suggesting significant differences between curves across all time points (Figure 4H).

## 4 DISCUSSION

Accurate interpretation of ECIS data are important as these in vitro measurements of barrier function often serve as foundations for in vivo preclinical studies. As there are no structured guidelines for analyzing these types of data, there is significant heterogeneity in the methodology. Most analyses of ECIS data distill these large datasets into a single point for statistical comparison between treatment groups. Because ECIS measurements provide continuous data that are repeated measures, more complex data analyses may be useful to better characterize responses to injurious stimuli. Our studies highlight that polynomials can be used to effectively fit ECIS data, thus allow for a more global comparison of ECIS profiles across experimental groups. When we compare coefficients generated from these equations with single‐point analyses, we observe instances of congruent (e.g., thrombin, GSK, and LPS 100 µg) as well as divergent (LPS 50 µg) results, suggesting that single time‐point analyses may not always be fully representative of the underlying ECIS data.

Stimuli leading to rapid changes of shorter duration resulted in concordance between coefficients generated from these equations and single time point analyses (i.e., TER_Max_). For instance, when there were no observed differences in conditions treated with GSK—a barrier profile characterized by an acute drop in TER with a slow recovery—coefficient analysis was not able to identify any differences. Furthermore, as shown in Figure 2, when TER_Max_ was significantly decreased due to thrombin, there was a marked impact only on coefficient *a*_1_. This is not surprising because while the TER_Max_ was different, the time to achieve the TER_Max_ was not different between the two groups suggesting that the slope of the downward inflection is steeper, which is reflected the differences in coefficient *a*_1_ (Suresh et al., 2019). Identifications of differences in coefficients may implicate biological processes that shape specific portions of the resultant fitted ECIS curve.

Stimuli leading to slow changes over longer duration, that is, LPS as shown in Figure 4, resulted in discordant data when comparing coefficients generated from these equations with single‐point analyses. In these scenarios, the fact that polynomial fitting does not produce curves that are statistically different from each other suggests that while TER_Max_ may be difference, the curves themselves may not represent significant differences in changes in TER over time.

While this methodology provides rigorously generated data, several factors must be initially taken into account. First, before comparing coefficients one must ensure that the generated fit is adequate and representative of the original observed curve. We have taken measures to ensure the fitting of our polynomial equation is adequate. As seen in Figure 1d–e, our fits are quite accurate based on the stimuli that were tested. Thrombin, GSK and LPS result in relative gradual transitions of the observed curves with resultant inflection points. However, certain stimuli may not be suitable for this type of analyses (e.g., sphingosine‐1‐phosphate [S1P]). As seen in Figure S2A (http://doi.org/10.5281/zenodo.4542523) the representative fitted curve for FTY720, a S1P analog, stimulated cells does not overlie the plotted/observed curve very well. This is quantitatively reflected in *β*_o_ values that are significantly higher than zero. (Figure S2B). This is likely because S1P results is a very sharp rise in barrier function, as measured by a sudden and near vertical increase in the observed ECIS curve; such a sharp inflection can be difficult to fit using polynomials.

Another factor to consider is the length of ledge (time before stimulus is given). Because the purpose of the ledge is mainly to show barrier stability before a stimulus is given, the duration plotted is often chosen arbitrarily. However, the duration of the ledge included for curvilinear fitting using a polynomial equation will affect the resultant coefficients. If the ledge is prolonged, the relative duration of the stimulus effect is shortened. Thus, a longer ledge may mask the effects of the stimulus. The purpose of the ledge is mainly to anchor the starting point for the resultant curvilinear fitting. To better understand the effect of ledge length on coefficient values, we purposefully manipulated the ledge time analyzed for an ECIS curve from cells treated with thrombin. For these experiments, we trimmed to ledge sequentially and compared the coefficients for curves with larger ledges compared to shorter ledges. Changing the ledge duration affected the goodness of polynomial fit (Figure S3). Unsurprisingly, this led to differences in coefficient values depending on the chosen ledge length (data not shown). This underscores the importance of standardizing the ledge time across experiments, and recognition of the fact that the ledge length can affect both the quality of polynomial fit and the raw values of the coefficients obtained.

In conclusion, we propose the use of polynomial curves to fit ECIS data in order to gain a better understanding of differences between experimental conditions. This method does not reduce data to a single value (as is the case with TER_Max_), and may reveal additional insights into biology. Furthermore, our data suggest that TER_Max_ and polynomial fit results may be discordant in some cases, and that this difference requires further exploration. Lastly, we identify the time before stimulus treatment (i.e., the ledge) as a potential factor that could affect adequacy of fit and coefficient measurements; thus, this value should be equivalent across experimental conditions. Using a standardized, unbiased method such as polynomial fitting could provide additional rigor and reproducibility to ECIS experiments, as well as provide greater insights into the biology reflected within these complex data.

## CONFLICTS OF INTEREST

None.

## AUTHOR CONTRIBUTIONS

KS, NP, SMD, and MD were involved in the conception, hypotheses delineation, and design of the study; KS, LS, LJ, and MD performed the experiments/data acquisition or analysis; KS, NP, SMD, MD wrote the manuscript or provided substantial involvement in its revision.

## Supporting information



Fig S1‐S3

Code S1
