# Supplementary material for: Comparison of polynomial fitting versus single time point analysis of ECIS data for barrier assessment
Source: Physiol Rep. 2021 Oct 4;9(19):e14983. doi: 10.14814/phy2.14983 (PMC8488550; doi:10.14814/phy2.14983)
Supplement: Supplementary file 1 — Fig S1‐S3 [file PHY2-9-e14983-s002.pptx]

## Slide 1
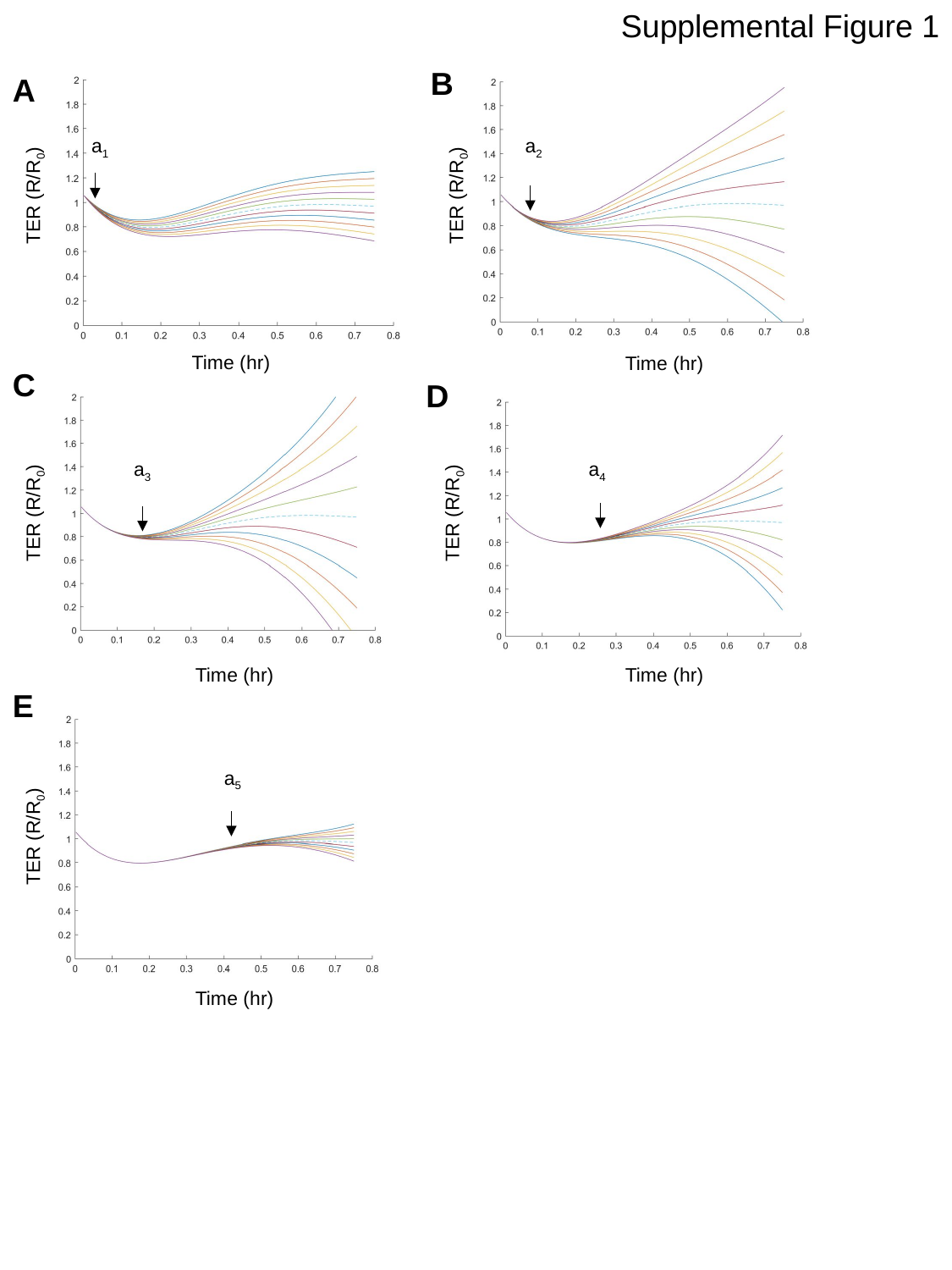

Supplemental Figure 1
A
TER (R/R0)
a1
Time (hr)
B
a2
Time (hr)
TER (R/R0)
C
a3
TER (R/R0)
Time (hr)
D
a4
Time (hr)
TER (R/R0)
E
a5
TER (R/R0)
Time (hr)

## Slide 2
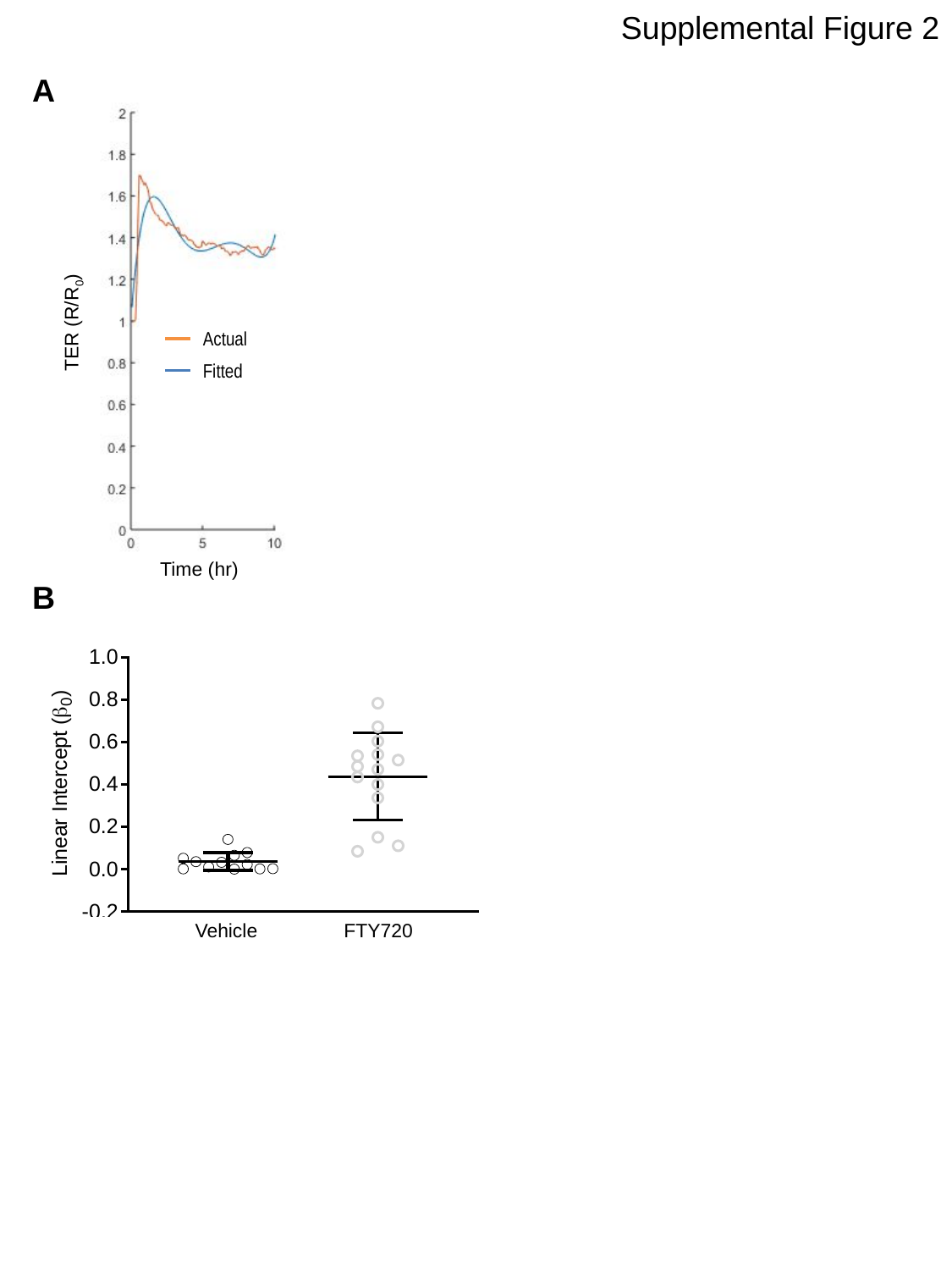

Supplemental Figure 2
A
Actual
Fitted
TER (R/R0)
Time (hr)
B
Vehicle
FTY720

## Slide 3
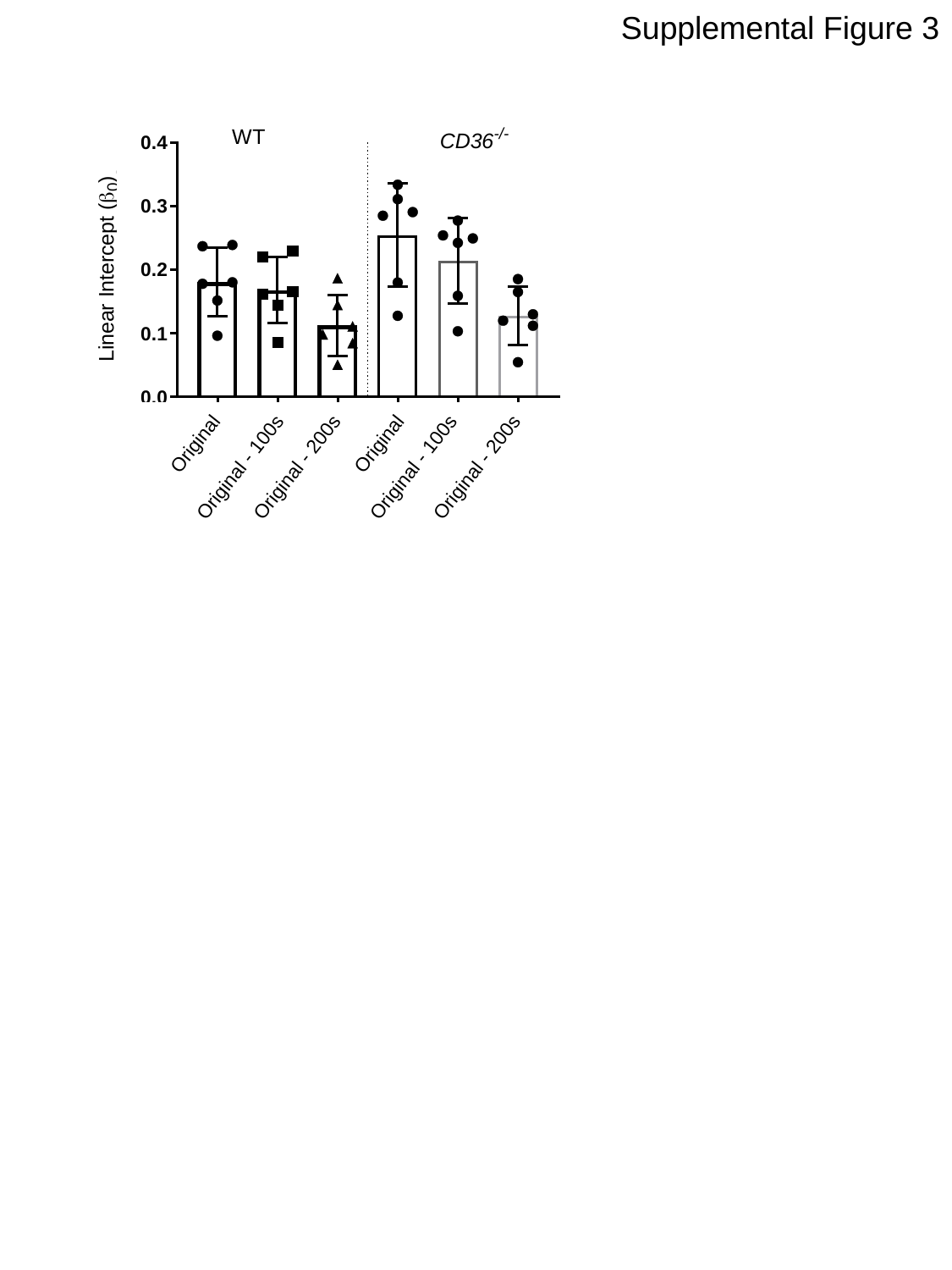

Supplemental Figure 3
Original
Original
Original - 100s
Original - 200s
Original - 100s
Original - 200s
