# Supplementary material for: Comparison of polynomial fitting versus single time point analysis of ECIS data for barrier assessment
Source: Physiol Rep. 2021 Oct 4;9(19):e14983. doi: 10.14814/phy2.14983 (PMC8488550; doi:10.14814/phy2.14983)
Supplement: Supplementary file 2 — Code S1 [file PHY2-9-e14983-s001.pdf]

ECIS Curvefitting

Karthik Suresh, Winter 2021.

Run on Matlab r2020b, Update 3

The idea here is to feed normalized (and non-normalized) ECIS data to this matlab script so that we can generate fitted polynomials for these curves.

For polynomial fits, we have scaled the X axis by a factor of 400. The reason for this is two fold: first, polys fit better with smaller numbers for the x axis. Second, the ECIS machine records values per well at a sample rate that approximates 1/400 of an hour per read. So, 400 values=1 hour of time.

Load Raw Data

```
% ECIS  Curvefitting
% Initial Code: 07-08/2018
% Revised: 2019, 2020, 02/2021

% load raw normalized data
wt = load("data/wt_lps.csv");
fyn = load("data/fyn_lps.csv");
fyn_lps50 = load("data/fyn_lps_50.csv");
wt_lps50=load("data/wt_lps_50.csv");
thrombin=load("data/wt_thrombin.csv");
thrombindevd=load("data/wt_thrombin+devd.csv");
WT_GSK = load("data/wt_gsk_2.csv");
CD36_GSK = load("data/cd36_gsk_2.csv");
fyn_GSK = load("data/fyn_GSK_2.csv");
unn_WT_GSK=load("data/unn_wt_GSK.csv");
unn_Fyn_GSK=load("data/unn_fyn_GSK.csv");
unn_CD36_GSK = load("data/unn_CD36_gsk.csv")
```

```
unn_CD36_GSK = 561×12
10^3 ×
    1.2559    1.2150    1.2364    1.2494    1.0791    1.1947    0.8084    0.9211 ···
    1.2557    1.2149    1.2361    1.2496    1.0798    1.1951    0.7397    0.8815
    1.2561    1.2144    1.2362    1.2493    1.0800    1.1949    0.7305    0.8685
    1.2556    1.2141    1.2360    1.2494    1.0796    1.1948    0.7198    0.8555
    1.2555    1.2139    1.2362    1.2497    1.0795    1.1945    0.7085    0.8427
    1.2558    1.2139    1.2363    1.2498    1.0793    1.1947    0.6958    0.8276
    1.2559    1.2132    1.2363    1.2504    1.0796    1.1950    0.6840    0.8115
    1.2561    1.2127    1.2360    1.2505    1.0796    1.1961    0.6708    0.7948
    1.2559    1.2127    1.2361    1.2500    1.0798    1.1965    0.6580    0.7777
    1.2560    1.2132    1.2367    1.2496    1.0798    1.1969    0.6457    0.7601
      ⋮
```

```
s1p_Tys = load("data\s1p-Tys.csv")
```

```
s1p_Tys = 121×14
    1.0000    1.0000    1.0000    1.0000    1.0000    1.0000    1.0000    1.0000 ···
    0.9990    1.0000    0.9640    0.9990    0.9060    1.0070    0.9970    1.0060
    0.9930    0.9930    0.9370    0.9960    0.8350    1.0030    1.0060    0.9960
    0.9880    0.9890    0.9170    0.9940    0.7790    0.9990    1.0010    0.9960
    1.1470    1.1380    0.9060    1.0230    0.7430    1.0940    1.1380    1.0800
    1.5640    1.5830    1.0450    1.5690    0.7150    1.5280    1.3770    1.3070
    1.6290    1.6250    1.0970    1.6520    0.6910    1.4940    1.3830    1.3330
    1.6590    1.5860    1.0990    1.6470    0.6760    1.4460    1.3600    1.2990
    1.6620    1.5690    1.0900    1.6680    0.6680    1.4160    1.3330    1.2790
    1.6630    1.5620    1.0880    1.6630    0.6580    1.4050    1.3190    1.2650
      ⋮
```

```
% load raw unnormalized data
unn_wt = load("data/unn_wt_lps.csv")
```

```
unn_wt = 6532×12
10^3 ×
    1.5383    1.3447    0.8722    1.1224    1.4438    1.4159    1.2220    1.7666 ···
    1.5378    1.3440    0.8721    1.1225    1.4433    1.4154    1.2217    1.7654
    1.5375    1.3433    0.8721    1.1226    1.4422    1.4154    1.2215    1.7656
    1.5379    1.3432    0.8720    1.1223    1.4425    1.4153    1.2213    1.7644
    1.5376    1.3428    0.8722    1.1231    1.4437    1.4152    1.2212    1.7642
    1.5369    1.3425    0.8721    1.1230    1.4434    1.4154    1.2210    1.7631
    1.5361    1.3419    0.8723    1.1230    1.4439    1.4153    1.2210    1.7651
    1.5356    1.3421    0.8722    1.1234    1.4435    1.4147    1.2213    1.7656
    1.5344    1.3420    0.8721    1.1230    1.4443    1.4143    1.2220    1.7644
    1.5341    1.3417    0.8718    1.1233    1.4447    1.4143    1.2212    1.7660
      ⋮
```

```
unn_fyn = load("data/unn_fyn_lps.csv")
```

```
unn_fyn = 7178×8
10^3 ×
    0.7934    0.7547    0.5159    0.4790    0.4982    0.5743    0.7856    0.6982
    0.7936    0.7552    0.5161    0.4791    0.4992    0.5745    0.7858    0.6979
    0.7935    0.7552    0.5161    0.4791    0.5004    0.5747    0.7859    0.6979
    0.7936    0.7553    0.5160    0.4791    0.5014    0.5748    0.7859    0.6976
    0.7934    0.7551    0.5161    0.4791    0.5025    0.5751    0.7859    0.6976
    0.7934    0.7553    0.5162    0.4791    0.5035    0.5754    0.7859    0.6977
    0.7933    0.7554    0.5161    0.4791    0.5047    0.5754    0.7859    0.6979
    0.7934    0.7555    0.5163    0.4792    0.5059    0.5756    0.7864    0.6979
    0.7936    0.7556    0.5163    0.4791    0.5071    0.5758    0.7865    0.6981
    0.7938    0.7559    0.5164    0.4791    0.5084    0.5758    0.7868    0.6982
      ⋮
```

```
unn_wt50 = load("data/unn_wt_lps50.csv")
```

```
unn_wt50 = 7039×4
10^3 ×
    2.0809    1.9900    0.8246    0.7101
    2.0131    1.9438    0.8157    0.7044
    1.9736    1.9173    0.8125    0.7015
    1.9357    1.8893    0.8094    0.6990
    1.9003    1.8554    0.8061    0.6967
    1.8646    1.8216    0.8027    0.6944
    1.8323    1.7956    0.8000    0.6917
```

```
    1.8007    1.7701    0.7962    0.6892
    1.7723    1.7468    0.7936    0.6869
    1.7438    1.7247    0.7910    0.6845
      ⋮
```

```
unn_fyn50 = load("data/unn_fyn_lps50.csv")
```

```
unn_fyn50 = 7060×3
10^3 ×
    0.7907    0.7361    1.2233
    0.7886    0.7340    1.2213
    0.7867    0.7321    1.2187
    0.7849    0.7302    1.2161
    0.7830    0.7286    1.2144
    0.7810    0.7275    1.2123
    0.7792    0.7260    1.2098
    0.7770    0.7246    1.2065
    0.7753    0.7226    1.2042
    0.7730    0.7206    1.2022
      ⋮
```

```
wttimes = 1:size(wt,1);
fyntimes = 1:size(fyn,1);
wttimes=wttimes.';
fyntimes=fyntimes.';




unn_wt50 = unn_wt50(1000:7000,:)
```

```
unn_wt50 = 6001×4
10^3 ×
    1.3253    1.2358    0.8713    0.7800
    1.3249    1.2354    0.8707    0.7797
    1.3241    1.2357    0.8702    0.7799
    1.3235    1.2366    0.8703    0.7801
    1.3236    1.2374    0.8705    0.7800
    1.3142    1.2474    0.8710    0.7802
    1.2825    1.2351    0.8711    0.7806
    1.2536    1.1908    0.8709    0.7810
    1.2296    1.1604    0.8710    0.7813
    1.2106    1.1457    0.8708    0.7811
      ⋮
```

```
unn_fyn50=unn_fyn50(1000:7000,:)
```

```
unn_fyn50 = 6001×3
10^3 ×
    0.7472    0.7549    1.1581
    0.7476    0.7550    1.1578
    0.7481    0.7551    1.1576
    0.7483    0.7552    1.1581
    0.7490    0.7554    1.1581
    0.7495    0.7555    1.1586
    0.7499    0.7558    1.1586
    0.7505    0.7559    1.1589
    0.7509    0.7559    1.1597
```

```
    0.7512    0.7563    1.1603
     ⋮
```

Summary statistics

```
avgWT = mean(wt.')
```

```
avgWT = 1×4668
    0.9961    0.9964    0.9967    0.9970    0.9971    0.9973    0.9975    0.9978 ⋯
```

```
avgFyn = mean(fyn.')
```

```
avgFyn = 1×4505
    1.0083    1.0085    1.0086    1.0086    1.0087    1.0089    1.0090    1.0090 ⋯
```

```
avgWT2 = avgWT(1:length(avgFyn))
```

```
avgWT2 = 1×4505
    0.9961    0.9964    0.9967    0.9970    0.9971    0.9973    0.9975    0.9978 ⋯
```

```
wtFynMeanDiff=avgWT2-avgFyn
```

```
wtFynMeanDiff = 1×4505
   -0.0122   -0.0120   -0.0119   -0.0116   -0.0116   -0.0116   -0.0115   -0.0112 ⋯
```

```
avgWT_lps50 = mean(wt_lps50.')
```

```
avgWT_lps50 = 1×6210
    0.9852    0.9867    0.9879    0.9892    0.9906    0.9919    0.9930    0.9941 ⋯
```

```
avgFyn_lps50=mean(fyn_lps50.')
```

```
avgFyn_lps50 = 1×6210
    1.0000    1.0003    1.0005    1.0005    1.0008    1.0012    1.0014    1.0015 ⋯
```

```
wtFyn_lps50_MeanDiff = avgWT_lps50-avgFyn_lps50
```

```
wtFyn_lps50_MeanDiff = 1×6210
   -0.0148   -0.0136   -0.0126   -0.0113   -0.0103   -0.0093   -0.0084   -0.0074 ⋯
```

Goodness of fit testing on polynomial fits of various orders

In order to figure out what type of polynomial to use, we calculate goodness of fit betwen the fitted polynomial and the empiric data using a 3rd, 5th or 7th order polynomial. The dataset used for these test fits is 'wt', which is the WT MLMVEC response to 100ug LPS.

```
%There is a question of whether a 5th or a 9th order polynomial is best.
%Let's do some goodness of fit with MSE as the fit test

g1=CalcGOF(wt,3);
```

```
g2=CalcGOF(wt,5);
g3=CalcGOF(wt,7);

g4=CalcGOF(fyn,3);
g5=CalcGOF(fyn,5);
g6=CalcGOF(fyn,7);

g7=CalcGOF(thrombin,3);
g8=CalcGOF(thrombin,5);
g9=CalcGOF(thrombin,7);

% this is the GOF of 2 random polynomials, which should have a GOF of 1
grand=goodnessOfFit(rand(5),rand(5), 'MSE');
```

graphing GOF results

```
figure
hold on
bar([3,5,7],[ mean(g1),mean(g2),mean(g3)])
errorbar([3,5,7],[mean(g1),mean(g2),mean(g3)], [std(g1)/sqrt(length(g1)), std(g2)/sqrt(length(g
hold off
```

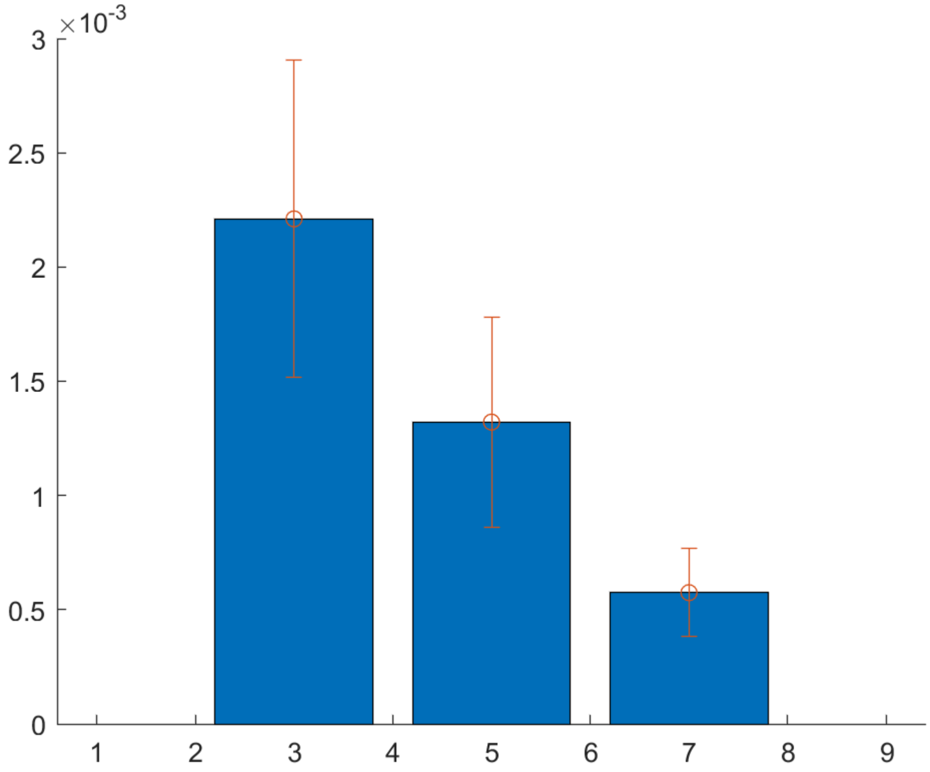

Exploratory graphs

Here, we can use plotSpline() to plot individual fits, plotSpline accepts a vector of times, the output of FitPoly(which fits the polynomial) and the original data. It returns a plot with the fitted poly and the original data.

```
%------------EARLY EXPLORATORY GRAPHS---------------
figure
% plot a spline for the first WT condition with a 5th and a 9th order
plotSpline(wttimes/400, FitPoly(wttimes/400,wt(:,4),5),wt(:,4));
```

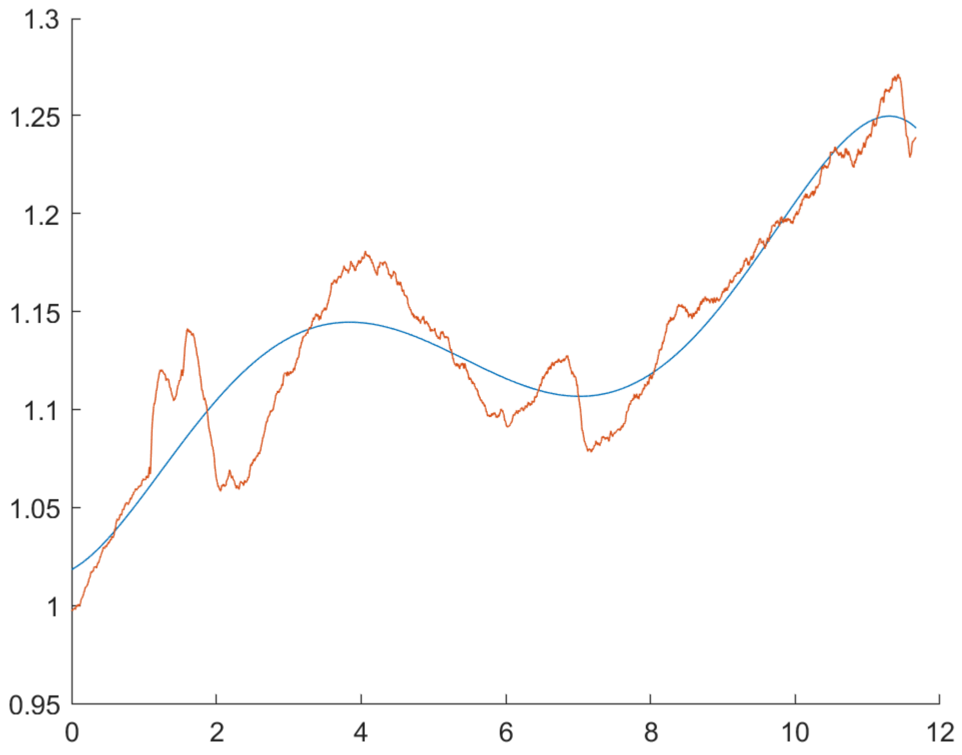


```
figure
plotSpline(wttimes/400, FitPoly(wttimes/400,wt(:,5), 5),wt(:,5));
```

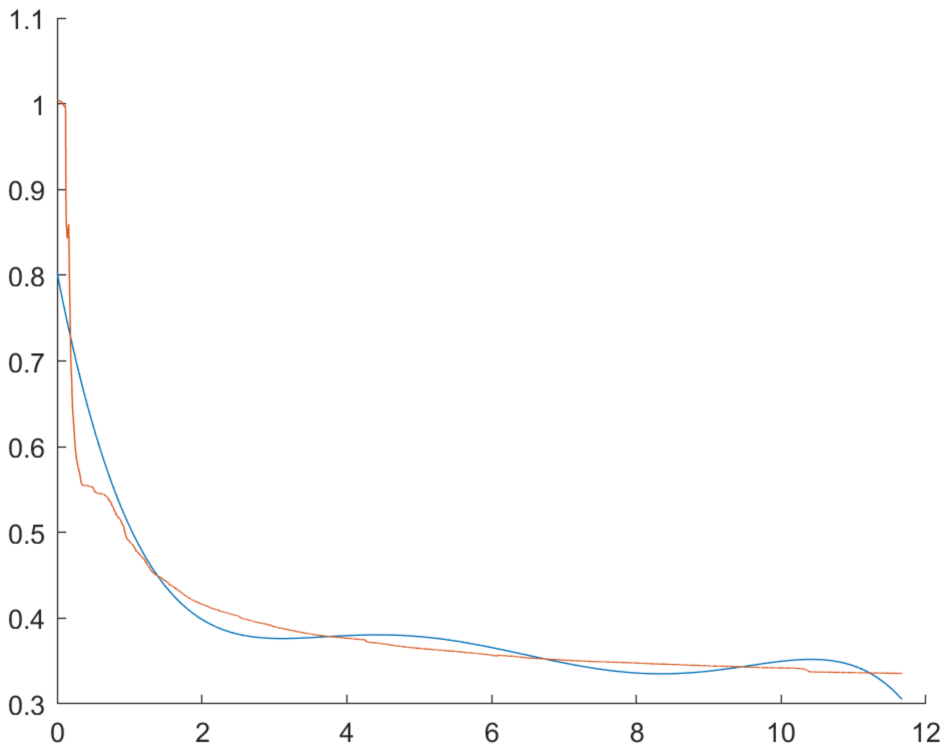


```
figure
hold on
subplot(1,2,1)

plotSpline(wttimes/400, FitPoly(wttimes/400,wt(:,2), 5),wt(:,2));
ylim([0,2.5])
subplot(1,2,2)
plotSpline(fyntimes/400, FitPoly(fyntimes/400,fyn(:,3), 5),fyn(:,3));
ylim([0,2.5])
hold off
```

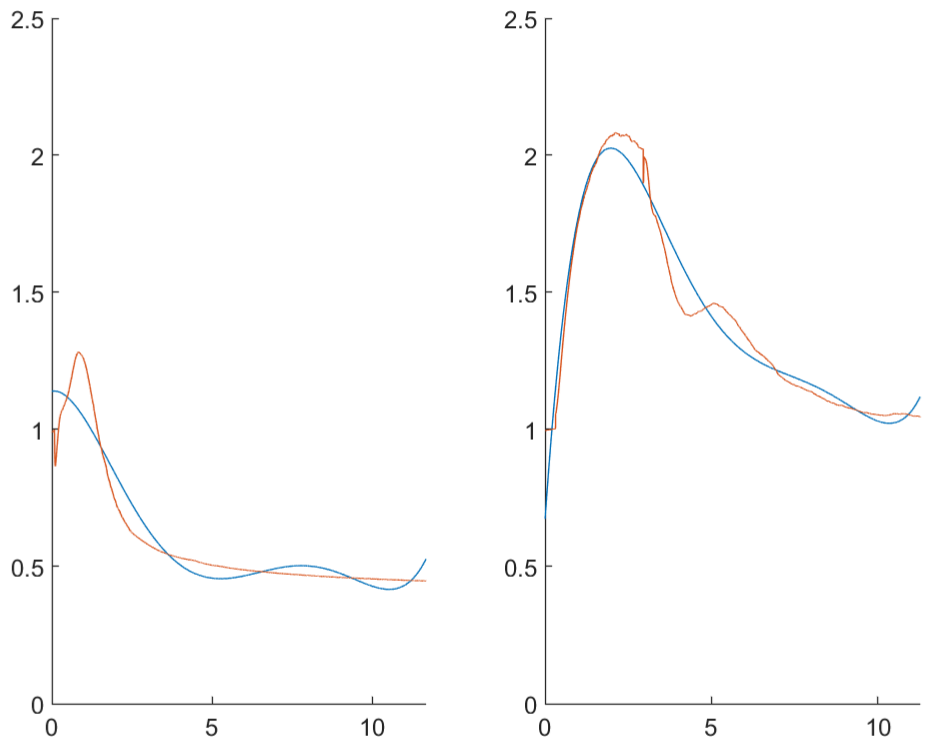


```
fynsplines = [fyntimes/400, FitPoly(fyntimes/400, fyn(:,3),5),fyn(:,3)]
```

```
fynsplines = 4505×3
    0.0025    0.6742    0.9957
    0.0050    0.6786    0.9957
    0.0075    0.6829    0.9957
    0.0100    0.6873    0.9961
    0.0125    0.6916    0.9959
    0.0150    0.6959    0.9961
    0.0175    0.7002    0.9963
    0.0200    0.7045    0.9963
    0.0225    0.7088    0.9965
    0.0250    0.7130    0.9967
      ⋮
```

```
%plotSpline(fyntimes, FitPoly(fyntimes,fyn(:,2), 9),fyn(:,2));
%plotSpline(fyntimes, FitPoly(fyntimes,fyn(:,3), 9),fyn(:,3));
%-------------------------------------------------------------
```

Run fits for an entire condition at one time

plotFits accepts the following arguments: plotFits(cond,order,n, ymin,ymax). It will plot fits for the condition *cond* using a poly fit of order *order.* This is a basically a wrapper for plotSpline() that recursively calls plotSpline() for each subplot. The number of n's you would like simulataneously displayed is *n. ymin/ymax* dictate y axis for output.

plotFits2 does the same but allows specification of beginning and end (so, you can ask for fits for experiments 3 - 5 by specifying plotFits2(cond, order,3,5,ymin,max), for instance

For all of our (JHU) ECIS data, the delta t between time points is the same. the delta t for the S1P data is different, so the scaling factor is 12, not 400. So, for this data set, we use PlotFits_s1p rather than PlotFits2. For future, probably a better way to do this is to specify scaling factor as an argument to the PlotFits function.

```
plotFits2(wt,5,4,6,0,2)
```

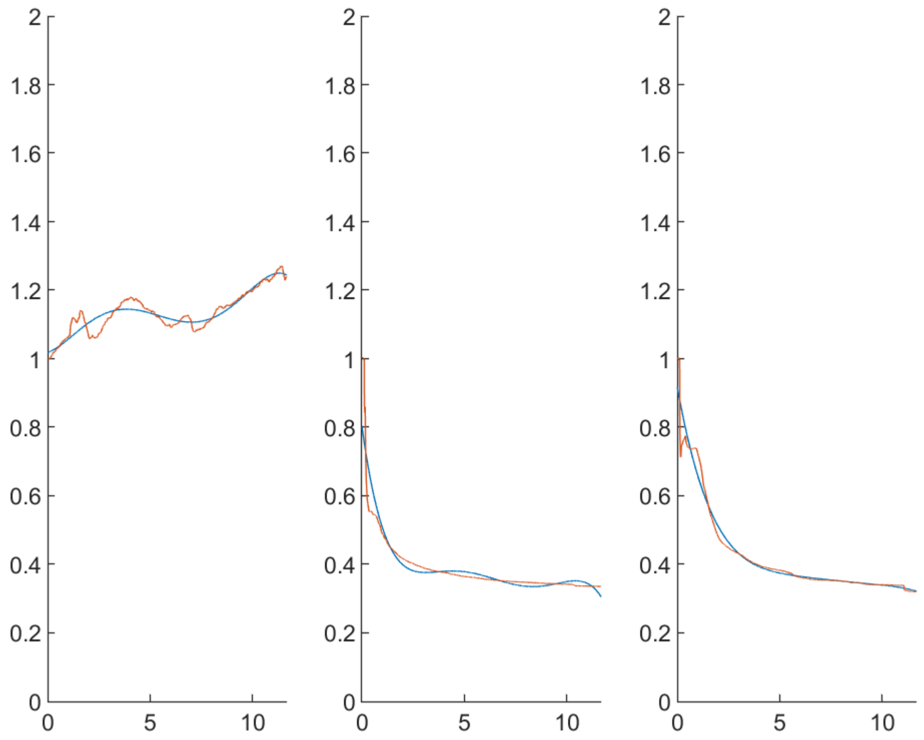

```
ans =
  Figure (5) with properties:

      Number: 5
        Name: ''
       Color: [1 1 1]
    Position: [681 679 560 420]
       Units: 'pixels'

  Show all properties
```

```
WTSpline = [wttimes/400, FitPoly(wttimes/400, wt(:,6), 5), wt(:,6)]
```

```
WTSpline = 4668×3
    0.0025    0.9177    1.0008
    0.0050    0.9169    1.0004
    0.0075    0.9161    1.0008
    0.0100    0.9152    1.0006
    0.0125    0.9144    1.0001
    0.0150    0.9136    1.0001
```

```
    0.0175    0.9128    1.0004
    0.0200    0.9119    1.0011
    0.0225    0.9111    1.0012
    0.0250    0.9103    1.0007
      ⋮
```

```
plotFits2(thrombin,5,3,5, 0,2)
```

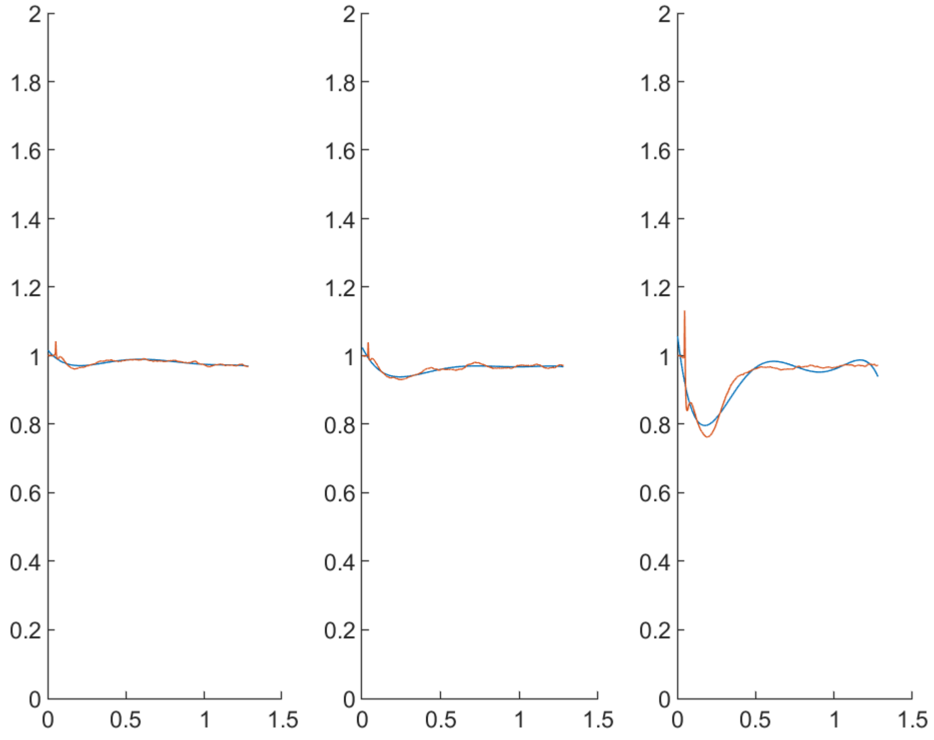

```
ans = 
  Figure (6) with properties:

      Number: 6
        Name: ''
       Color: [1 1 1]
    Position: [681 679 560 420]
       Units: 'pixels'

  Show all properties
```

```
thrombintimes = 1:size(thrombin,1)
```

```
thrombintimes = 1×512
     1     2     3     4     5     6     7     8     9    10    11    12    13 ···
```

```
thrombintimes=thrombintimes.'
```

```
thrombintimes = 512×1
     1
     2
     3
     4
     5
     6
```

```
     7
     8
     9
    10
     :
```

```
ThrombinSpline = [thrombintimes/400, FitPoly(thrombintimes/400, thrombin(:,5),5),thrombin(:,5)]
```

```
ThrombinSpline = 512×3
    0.0025    1.0567    1.0000
    0.0050    1.0476    0.9999
    0.0075    1.0387    1.0005
    0.0100    1.0300    1.0011
    0.0125    1.0215    1.0007
    0.0150    1.0132    1.0009
    0.0175    1.0051    0.9999
    0.0200    0.9972    0.9988
    0.0225    0.9896    0.9971
    0.0250    0.9821    0.9968
      :
```

```
plotFits_s1p(s1p_Tys,5,12,14, 0,2)
```

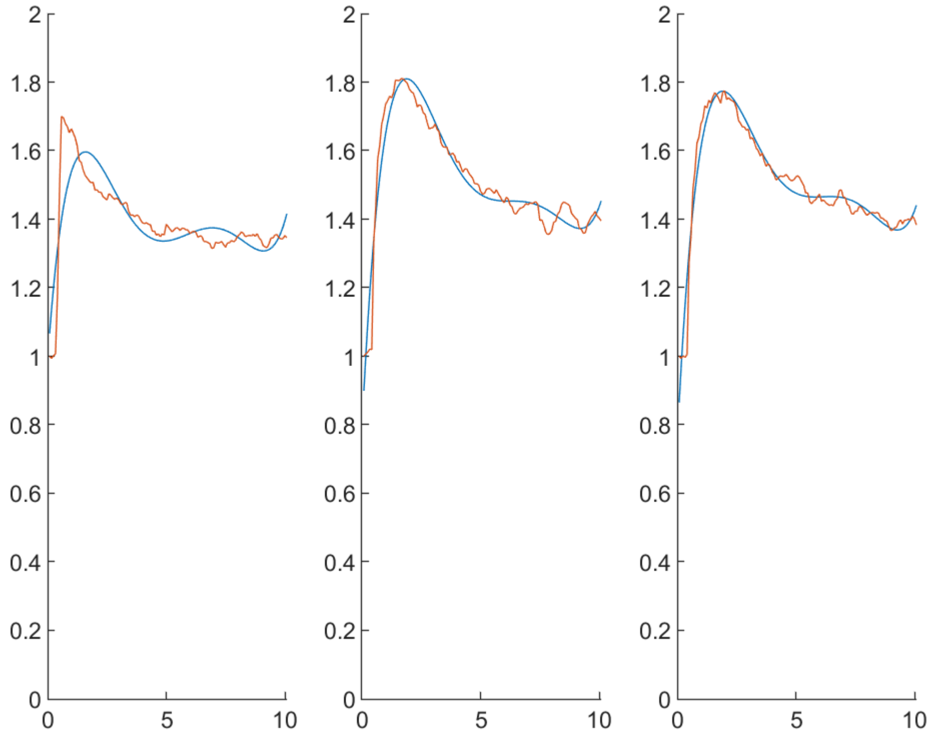


```
ans =
  Figure (7) with properties:

      Number: 7
        Name: ''
       Color: [1 1 1]
    Position: [681 679 560 420]
       Units: 'pixels'
```

```
  Show all properties
```

```
plotFits2(WT_GSK, 5,1,3,0.8,1.2)
```

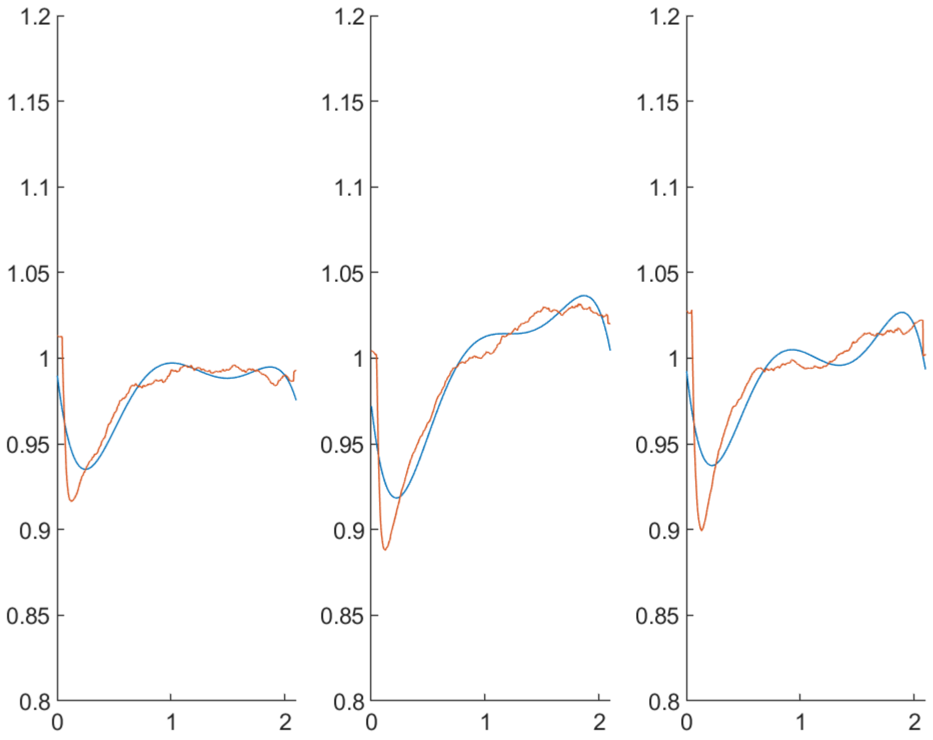


```
ans = 
  Figure (8) with properties:

      Number: 8
        Name: ''
       Color: [1 1 1]
    Position: [681 679 560 420]
       Units: 'pixels'

  Show all properties
```

```
plotFits2(CD36_GSK,5,1,3,0.8,1.2)
```

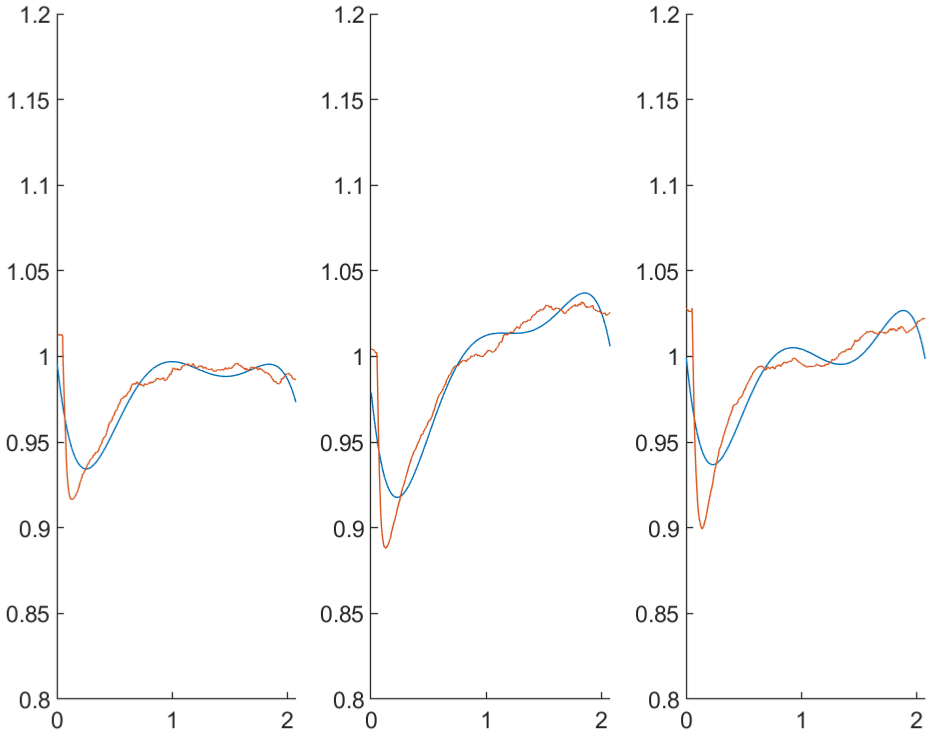


```
ans = 
  Figure (9) with properties:

      Number: 9
        Name: ''
       Color: [1 1 1]
    Position: [681 679 560 420]
       Units: 'pixels'

  Show all properties
```

```
plotFits2(fyn_GSK,5,1,3,0.8,1.2)
```

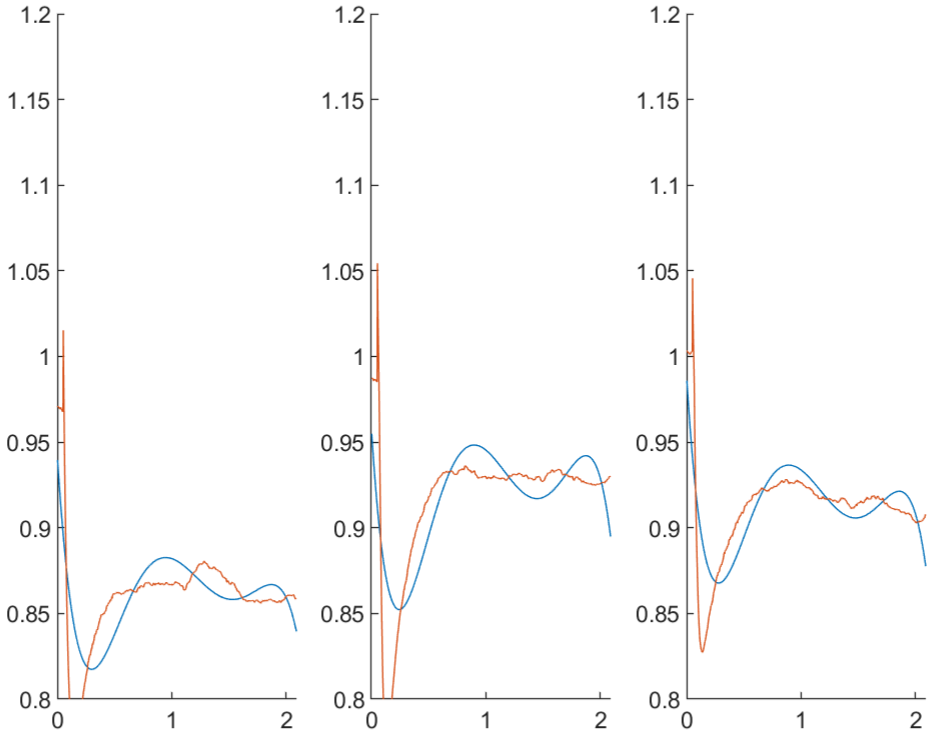


```
ans = 
  Figure (10) with properties:

      Number: 10
        Name: ''
       Color: [1 1 1]
    Position: [681 679 560 420]
       Units: 'pixels'

  Show all properties
```

```
GSKtimes=1:size(WT_GSK, 1)
```

```
GSKtimes = 1×840
     1     2     3     4     5     6     7     8     9    10    11    12    13 ···
```

```
GSKtimes=GSKtimes.'
```

```
GSKtimes = 840×1
     1
     2
     3
     4
     5
     6
     7
     8
     9
    10
     ⋮
```

```
GSKSpline = [GSKtimes/400, FitPoly(GSKtimes/400, WT_GSK(:,3), 5), WT_GSK(:,3)]
```

```
GSKSpline = 840×3
    0.0025    0.9924    1.0274
    0.0050    0.9910    1.0276
    0.0075    0.9895    1.0276
    0.0100    0.9881    1.0272
    0.0125    0.9867    1.0271
    0.0150    0.9854    1.0266
    0.0175    0.9840    1.0265
    0.0200    0.9827    1.0262
    0.0225    0.9814    1.0263
    0.0250    0.9802    1.0262
      :
```

```
plotFits(fyn,5,3, 0,2.5)
```

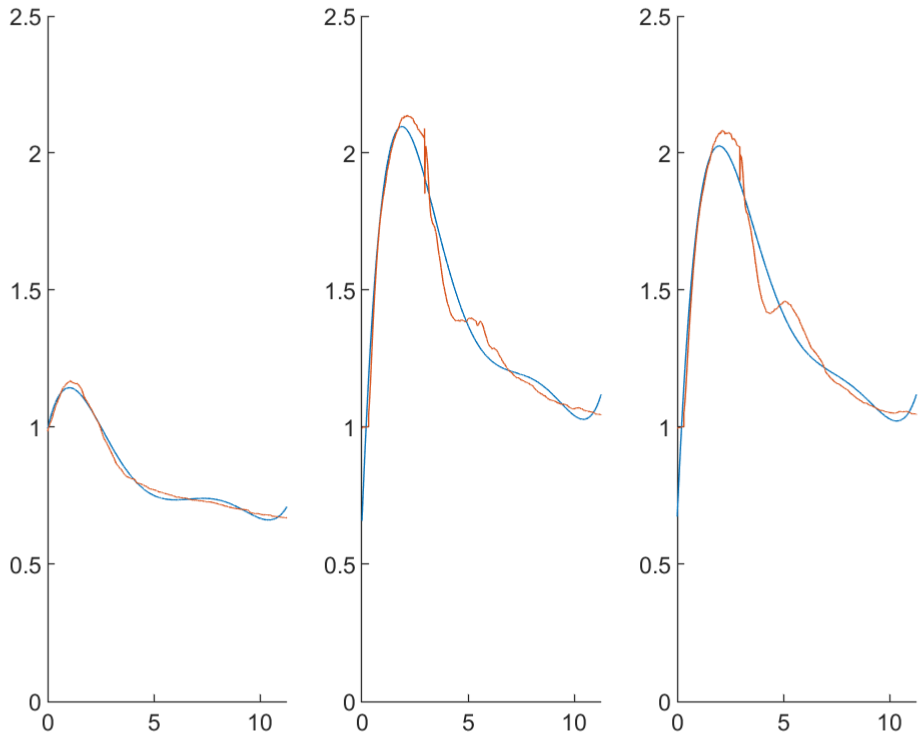


```
ans =
  Figure (11) with properties:

      Number: 11
        Name: ''
       Color: [1 1 1]
    Position: [681 679 560 420]
       Units: 'pixels'

  Show all properties
```

```
plotFits(unn_wt(1:5000,:), 5,2,200,1500)
```

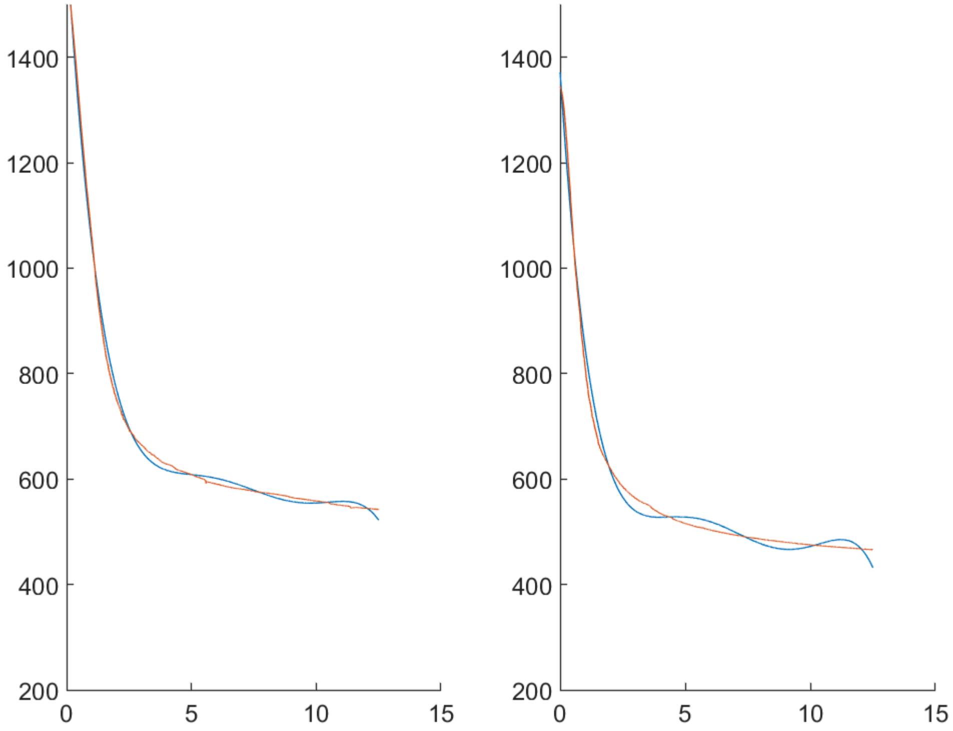


```
ans =
  Figure (12) with properties:

      Number: 12
        Name: ''
       Color: [1 1 1]
    Position: [681 679 560 420]
       Units: 'pixels'

  Show all properties
```

```
plotFits(unn_fyn(1:5000,:), 5,2,200,1500)
```

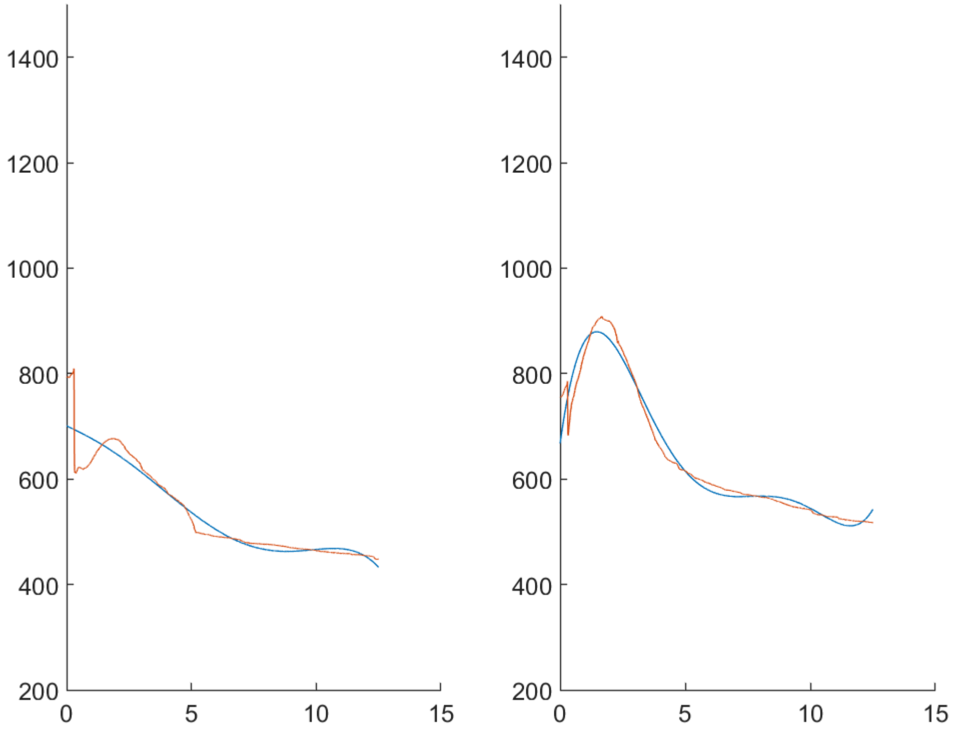


```
ans = 
  Figure (13) with properties:

      Number: 13
        Name: ''
       Color: [1 1 1]
    Position: [681 679 560 420]
       Units: 'pixels'

  Show all properties
```

```
plotFits(unn_wt50(1:5000,:), 5,2,200,2000)
```

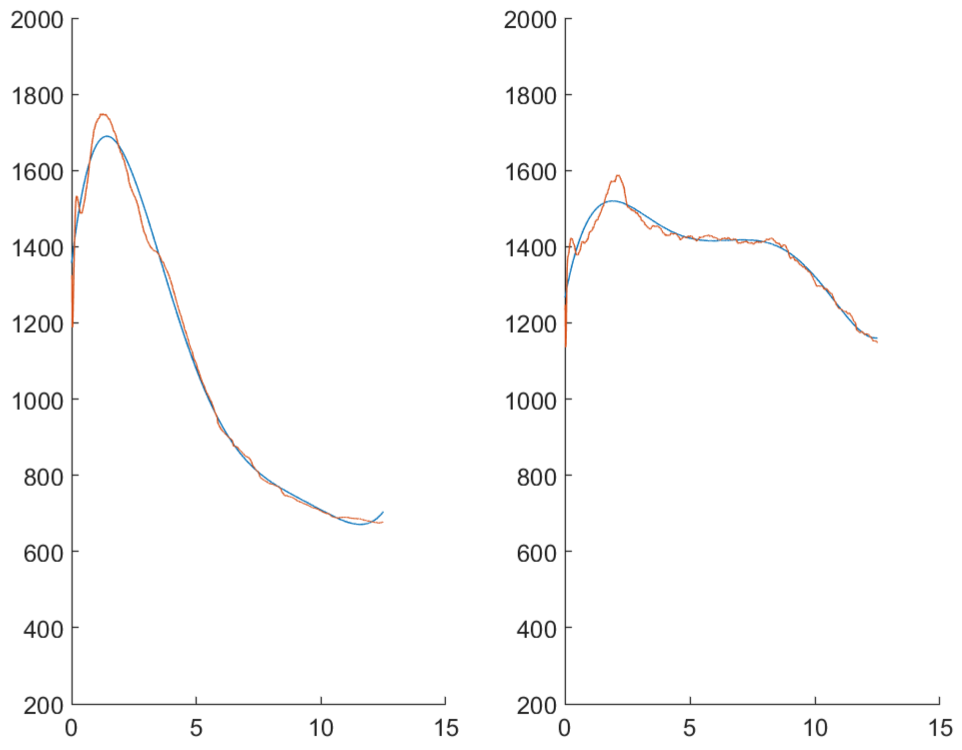


```
ans = 
  Figure (14) with properties:

      Number: 14
        Name: ''
       Color: [1 1 1]
    Position: [681 679 560 420]
       Units: 'pixels'

  Show all properties
```

```
plotFits(unn_fyn50(1:5000,:), 5,2,200,1500)
```

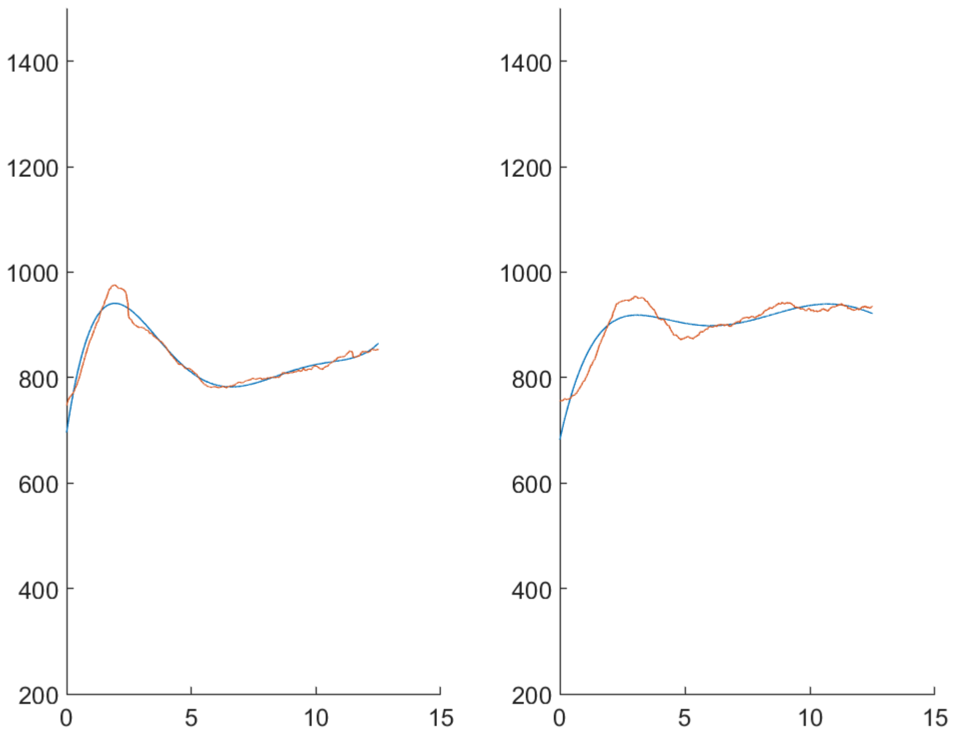


```
ans =
  Figure (15) with properties:

      Number: 15
        Name: ''
       Color: [1 1 1]
    Position: [681 679 560 420]
       Units: 'pixels'

  Show all properties
```

plot the raw data for WT and FYn response to 100ug LPS

```
% normalized data

figure
hold on
t=1:length(wt.');
t2=1:length(fyn.');
t=t/400;
t2=t2/400;
plot(t.', mean(wt.'),t2.', mean(fyn.'))
plot(t.', mean(wt.')+std(wt.')/sqrt(12), 'k--')
plot(t.', mean(wt.')-std(wt.')/sqrt(12), 'k--')

plot(t2.', mean(fyn.')+std(fyn.')/sqrt(8), 'k--')
plot(t2.', mean(fyn.')-std(fyn.')/sqrt(8), 'k--')
ylim([0,2.5])
hold off
```

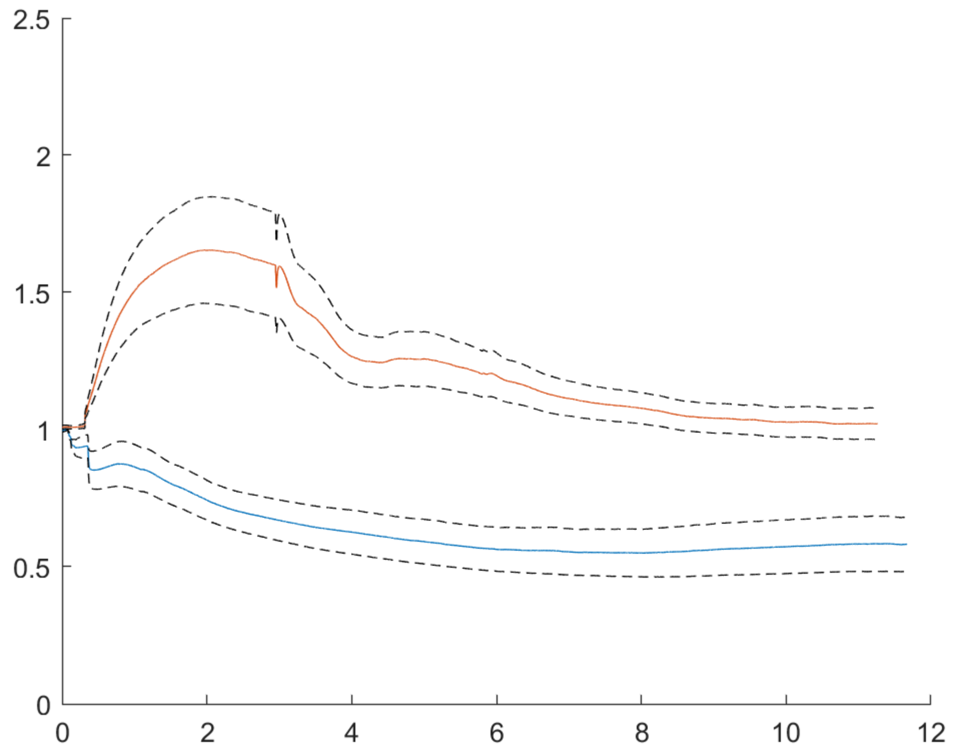


```
[h,p]=ttest(wt(3000,1:7),fyn(3000,1:7))
```

```
h = 1
p = 0.0148
```

```
% unnormalized data

figure
hold on
t3=1:length(unn_wt.');
t4=1:length(unn_fyn.');
t3=t3/1000;
t4=t4/1000;
plot(t3.', mean(unn_wt.'),t4.', mean(unn_fyn.'))
plot(t3.', mean(unn_wt.')+std(unn_wt.')/sqrt(12), 'k--')
plot(t3.', mean(unn_wt.')-std(unn_wt.')/sqrt(12), 'k--')

plot(t4.', mean(unn_fyn.')+std(unn_fyn.')/sqrt(8), 'k--')
plot(t4.', mean(unn_fyn.')-std(unn_fyn.')/sqrt(8), 'k--')

hold off
```

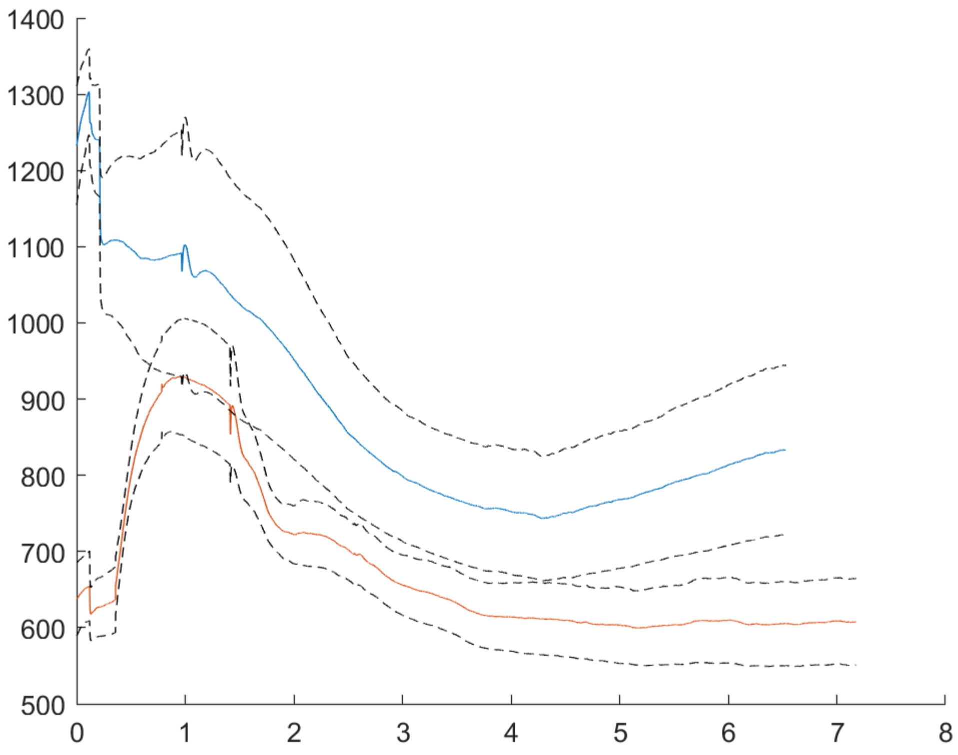

Collecting Coefficients

Here, we create matrices of dimension mxn where m=number of n's per condition, and n=numbebr of coeffcients. So, all the matrices below are of dimension i x 6 (since we're using a 5th order poly). CollectCoefficients accepts the matrix of data, the order of the poly an the number of n - aka the number of columns in the data)

```
WTCoeffs = CollectCoefficients(wt,5,12)
```

```
WTCoeffs = 12×6
    0.0002   -0.0046    0.0483   -0.1939    0.1164    1.0669
    0.0001   -0.0042    0.0423   -0.1565    0.0217    1.1396
   -0.0000    0.0003   -0.0013   -0.0044    0.0588    0.9928
   -0.0000    0.0012   -0.0102    0.0277    0.0198    1.0183
   -0.0001    0.0024   -0.0294    0.1661   -0.4352    0.8034
   -0.0000    0.0007   -0.0104    0.0821   -0.3315    0.9185
   -0.0001    0.0035   -0.0418    0.2310   -0.5774    0.9177
   -0.0001    0.0046   -0.0549    0.2970   -0.7053    0.9190
    0.0000   -0.0009    0.0126   -0.0652    0.0237    0.9587
    0.0000   -0.0004    0.0073   -0.0465    0.0237    0.9669
       ⋮
```

```
FynCoeffs = CollectCoefficients(fyn,5,7)
```

```
FynCoeffs = 7×6
    0.0001   -0.0044    0.0494   -0.2312    0.3362    0.9939
```

```
    0.0004   -0.0125    0.1560   -0.8713    1.9326    0.6542
    0.0003   -0.0106    0.1334   -0.7606    1.7455    0.6699
    0.0003   -0.0111    0.1385   -0.7842    1.7862    0.7402
    0.0003   -0.0114    0.1406   -0.7794    1.7224    0.7478
   -0.0000    0.0009   -0.0072    0.0169    0.0183    0.9852
    0.0000   -0.0002    0.0015   -0.0031    0.0265    1.0111
```

```
FynCoeffslps50 = CollectCoefficients(fyn_lps50,5,3)
```

```
FynCoeffslps50 = 3×6
    0.0000   -0.0012    0.0183   -0.1162    0.2602    1.0105
    0.0000   -0.0009    0.0137   -0.0935    0.2724    0.9385
   -0.0000   -0.0000    0.0024   -0.0347    0.1548    1.0479
```

```
WTCoeffslps50=CollectCoefficients(wt_lps50,5,4)
```

```
WTCoeffslps50 = 4×6
    0.0000   -0.0014    0.0236   -0.1645    0.3570    1.0136
    0.0000   -0.0008    0.0116   -0.0753    0.2004    1.0359
    0.0000   -0.0002    0.0041   -0.0358    0.1197    0.8766
    0.0000   -0.0000    0.0008   -0.0066    0.0053    0.9080
```

```
thrombinCoeffs = CollectCoefficients(thrombin,5,6)
```

```
thrombinCoeffs = 6×6
   -1.5271    5.7135   -7.9975    5.1096   -1.3698    1.0173
   -2.8748   10.4046  -13.7944    8.0597   -1.8670    1.0387
   -0.7093    2.7213   -3.8186    2.3284   -0.5641    1.0172
   -0.9057    3.5704   -5.2466    3.4664   -0.9433    1.0264
   -6.5922   23.5983  -30.8245   17.4915   -3.7788    1.0661
   -8.7290   30.1015  -37.1329   19.2039   -3.5326    1.0393
```

```
thrombindevdCoeffs = CollectCoefficients(thrombindevd,5,12)
```

```
thrombindevdCoeffs = 12×6
   -5.2593   18.5980  -24.1472   13.8399   -3.1296    1.0333
   -3.5164   13.6400  -19.2150   11.7339   -2.7975    1.0457
   -2.7006    9.7339  -12.9441    7.6548   -1.8202    1.0333
   -2.3765    9.0525  -12.8147    8.1844   -2.1952    1.0643
   -7.9188   28.4269  -37.2497   21.2832   -4.6698    1.0805
   -6.5831   24.0887  -32.4757   19.2981   -4.4695    1.0942
   -6.5584   23.4658  -30.7786   17.7068   -3.9202    1.0727
   -4.9980   18.5565  -25.3960   15.3479   -3.6125    1.0820
   -4.9061   18.3327  -25.2233   15.3137   -3.6454    1.0823
   -6.8308   24.9547  -33.4195   19.5335   -4.3337    1.0862
       ⋮
```

```
WT_GSK_Coeffs = CollectCoefficients(WT_GSK, 5,6)
```

```
WT_GSK_Coeffs = 6×6
   -0.1535    0.8872   -1.8635    1.6756   -0.5399    0.9912
   -0.1973    1.1088   -2.2639    1.9786   -0.5870    0.9734
   -0.2262    1.2405   -2.4423    2.0380   -0.5998    0.9939
   -0.1770    1.0102   -2.1027    1.8905   -0.6126    1.0207
   -0.2484    1.4134   -2.8935    2.4869   -0.7231    0.9850
   -0.1498    0.8603   -1.8092    1.6407   -0.5124    0.9879
```

```
CD36_GSK_Coeffs = CollectCoefficients(CD36_GSK, 5,6)
```

```
CD36_GSK_Coeffs = 6×6
   -0.1741    0.9925   -2.0594    1.8377   -0.5970    0.9973
   -0.2186    1.2196   -2.4751    2.1582   -0.6522    0.9804
```

-0.2420	1.3267	-2.6146	2.1927	-0.6591	1.0007
-0.1995	1.1248	-2.3161	2.0673	-0.6751	1.0274
-0.2849	1.5989	-3.2369	2.7677	-0.8194	0.9945
-0.1784	1.0032	-2.0681	1.8465	-0.5809	0.9945

```
fyn_GSK_Coeffs = CollectCoefficients(fyn_GSK, 5,7)
```

fyn_GSK_Coeffs = 7×6

-0.2535	1.4736	-3.1254	2.8821	-1.0369	0.9422
-0.3541	1.9743	-3.9630	3.3774	-1.0468	0.9575
-0.3122	1.7596	-3.5951	3.1654	-1.0725	0.9885
-0.1715	0.9879	-2.1065	1.9984	-0.7608	0.9873
-0.2728	1.5489	-3.1975	2.8558	-0.9792	0.9689
-0.0937	0.5712	-1.3254	1.4000	-0.5387	0.9770
0.0343	-0.1806	0.3062	-0.1530	-0.0277	0.9543

```
unn_WT_GSK_Coeffs = CollectCoefficients(unn_WT_GSK, 5,11)
```

unn_WT_GSK_Coeffs = 11×6

10^4 ×

-0.1015	0.4170	-0.6051	0.3514	-0.0434	0.1444
-0.1047	0.4174	-0.5895	0.3303	-0.0360	0.1402
-0.0831	0.3277	-0.4501	0.2307	-0.0072	0.1652
-0.0847	0.3654	-0.5778	0.4046	-0.1141	0.0934
-0.0907	0.3694	-0.5571	0.3801	-0.1024	0.1338
-0.2560	1.0737	-1.6639	1.1512	-0.3160	0.1390
-0.0412	0.1659	-0.2499	0.1783	-0.0611	0.0890
-0.1142	0.4834	-0.7517	0.5129	-0.1329	0.1328
-0.4156	1.8351	-3.0211	2.2367	-0.6590	0.1592
-0.1264	0.5246	-0.8127	0.5732	-0.1689	0.1985
⋮					

```
unn_CD36_GSK_Coeffs = CollectCoefficients(unn_CD36_GSK, 5,12)
```

unn_CD36_GSK_Coeffs = 12×6

10^4 ×

-0.1530	0.5967	-0.8555	0.5387	-0.1293	0.1248
-0.1737	0.6865	-0.9919	0.6241	-0.1441	0.1201
-0.2022	0.7928	-1.1298	0.6943	-0.1586	0.1226
-0.2124	0.8255	-1.1715	0.7257	-0.1719	0.1259
-0.1841	0.7260	-1.0481	0.6559	-0.1498	0.1059
-0.2135	0.8271	-1.1651	0.7128	-0.1639	0.1207
-0.2681	1.0429	-1.4613	0.8563	-0.1619	0.0661
-0.3694	1.4253	-1.9917	1.1780	-0.2339	0.0782
-0.3649	1.4324	-2.0449	1.2472	-0.2663	0.0934
-0.3623	1.4092	-1.9916	1.2051	-0.2584	0.0726
⋮					

```
unn_fyn_GSK_Coeffs = CollectCoefficients(unn_Fyn_GSK, 5,8)
```

unn_fyn_GSK_Coeffs = 8×6

10^3 ×

-0.2399	0.6864	-0.6481	0.2372	0.0353	0.6539
0.7923	-3.1272	4.5163	-2.8431	0.7115	0.6336
-0.4882	1.6743	-1.9655	0.8608	-0.0325	0.6426
-1.0336	3.5756	-4.2860	1.9839	-0.1596	0.7288
-0.8600	2.7812	-2.9148	0.8594	0.2672	1.1000
-0.7214	2.2692	-1.9988	-0.1979	0.9302	1.3494
-0.4267	1.5497	-1.8328	0.5124	0.3649	1.5204
-0.8846	3.1010	-3.6785	1.5707	0.0059	1.8897

```
unnWTCoeffs = CollectCoefficients(unn_wt, 5,12)
```

```
unnWTCoeffs = 12×6
10^3 ×
   -0.0000    0.0011   -0.0196    0.1658   -0.6560    1.5746
   -0.0000    0.0011   -0.0188    0.1527   -0.5739    1.3095
   -0.0000    0.0001   -0.0020    0.0153   -0.0203    0.9178
    0.0000   -0.0005    0.0076   -0.0476    0.1183    1.1044
   -0.0000    0.0012   -0.0209    0.1638   -0.5807    1.2595
   -0.0000    0.0006   -0.0125    0.1141   -0.5056    1.3989
   -0.0000    0.0012   -0.0202    0.1576   -0.5531    1.1623
   -0.0000    0.0021   -0.0359    0.2745   -0.9204    1.6455
    0.0000   -0.0012    0.0256   -0.2343    0.7387    1.1060
    0.0000   -0.0009    0.0207   -0.1999    0.6790    1.0646
      ⋮
```

```
unnFynCoeffs = CollectCoefficients(unn_fyn,5,8)
```

```
unnFynCoeffs = 8×6
    0.0032   -0.1534    2.5722  -16.0517    1.5650  692.4246
    0.0115   -0.5514    9.4392  -66.7975  138.0485  755.4407
    0.0247   -1.2781   24.3950 -205.4306  671.0452  302.3544
    0.0198   -1.0403   20.2217 -174.1583  584.8448  281.4729
    0.0222   -1.1691   22.8428 -197.9794  666.2531  337.7599
    0.0243   -1.2632   24.3119 -206.8210  682.3440  367.3711
   -0.0043    0.1875   -2.9858   21.9674  -69.3805  796.1516
   -0.0010    0.0578   -1.1089    7.2516    4.5186  705.9190
```

```
unnWTCoeffslps50=CollectCoefficients(unn_wt50,5,4)
```

```
unnWTCoeffslps50 = 4×6
10^3 ×
    0.0000   -0.0020    0.0319   -0.2155    0.4407    1.3972
    0.0000   -0.0011    0.0154   -0.0950    0.2368    1.3066
   -0.0000    0.0004   -0.0053    0.0235   -0.0219    0.8671
   -0.0000    0.0002   -0.0021    0.0119   -0.0419    0.7971
```

```
unnFynCoeffslps50=CollectCoefficients(unn_fyn50,5,3)
```

```
unnFynCoeffslps50 = 3×6
10^3 ×
    0.0000   -0.0010    0.0156   -0.1055    0.2665    0.7140
    0.0000   -0.0006    0.0095   -0.0675    0.2101    0.6839
   -0.0000    0.0004   -0.0021   -0.0243    0.2296    1.1171
```

```
s1p_Tys_Coeffs = CollectCoefficients_s1p(s1p_Tys,5,14)
```

```
s1p_Tys_Coeffs = 14×6
    0.0004   -0.0102    0.1028   -0.4583    0.8272    1.0311
    0.0003   -0.0082    0.0855   -0.3968    0.7560    1.0155
    0.0001   -0.0016    0.0145   -0.0598    0.1020    0.9781
    0.0004   -0.0108    0.1115   -0.5169    1.0150    0.9523
   -0.0002    0.0046   -0.0472    0.2150   -0.4106    0.9137
    0.0002   -0.0066    0.0666   -0.2930    0.5176    1.0404
    0.0001   -0.0044    0.0477   -0.2303    0.4360    1.0229
    0.0001   -0.0036    0.0396   -0.1936    0.3962    1.0011
    0.0003   -0.0087    0.0895   -0.4125    0.7795    0.9578
    0.0003   -0.0084    0.0863   -0.3964    0.7429    0.9984
      ⋮
```

Ledge Testing

So, the idea here is that we don't know what the optimal "ledge" or stability period is for a given experiment. For determine whether ledge length affects goodness of fit, we alter the ledge by making it shorter and reassess GOF.

Ledge testing for GSK

```
%-------------LEDGE TESTING for GSK------------------
% collect coefficients with changes in ledge
WT_GSK_Coeffs_minus10 = CollectCoefficients(WT_GSK(10:end,1:end),5,6)
```

```
WT_GSK_Coeffs_minus10 = 6×6
   -0.1283    0.7276   -1.4898    1.2823   -0.3632    0.9678
   -0.1635    0.8953   -1.7695    1.4663   -0.3617    0.9450
   -0.1899    1.0105   -1.9085    1.4888   -0.3624    0.9641
   -0.1498    0.8364   -1.6943    1.4597   -0.4183    0.9949
   -0.2049    1.1396   -2.2590    1.8294   -0.4359    0.9491
   -0.1231    0.6925   -1.4203    1.2348   -0.3310    0.9644
```

```
WT_GSK_Coeffs_minus20 = CollectCoefficients(WT_GSK(20:end,1:end),5,6)
```

```
WT_GSK_Coeffs_minus20 = 6×6
   -0.0750    0.4141   -0.8105    0.6238   -0.0933    0.9357
   -0.0952    0.4938   -0.9029    0.6329   -0.0244    0.9064
   -0.1160    0.5741   -0.9661    0.5870   -0.0020    0.9230
   -0.0871    0.4680   -0.8974    0.6891   -0.1028    0.9572
   -0.1190    0.6346   -1.1676    0.7789   -0.0128    0.9011
   -0.0637    0.3455   -0.6745    0.5178   -0.0391    0.9303
```

```
WT_GSK_Coeffs_minus30 = CollectCoefficients(WT_GSK(30:end,1:end),5,6)
```

```
WT_GSK_Coeffs_minus30 = 6×6
   -0.0294    0.1536   -0.2639    0.1134    0.1049    0.9149
   -0.0356    0.1538   -0.1934   -0.0221    0.2255    0.8816
   -0.0444    0.1658   -0.1162   -0.1909    0.2898    0.8937
    0.0019   -0.0327    0.1358   -0.2571    0.2592    0.9183
   -0.0525    0.2539   -0.3679    0.0360    0.2699    0.8737
    0.0088   -0.0616    0.1635   -0.2487    0.2536    0.8997
```

```
CD36_GSK_Coeffs_minus10 = CollectCoefficients(CD36_GSK(10:end,1:end),5,6)
```

```
CD36_GSK_Coeffs_minus10 = 6×6
   -0.1522    0.8504   -1.7205    1.4747   -0.4310    0.9748
   -0.1881    1.0248   -2.0186    1.6796   -0.4389    0.9531
   -0.2087    1.1133   -2.1159    1.6754   -0.4332    0.9719
   -0.1758    0.9703   -1.9460    1.6698   -0.4925    1.0026
   -0.2467    1.3536   -2.6586    2.1590   -0.5494    0.9603
   -0.1558    0.8571   -1.7209    1.4763   -0.4120    0.9722
```

```
CD36_GSK_Coeffs_minus20 = CollectCoefficients(CD36_GSK(20:end,1:end),5,6)
```

```
CD36_GSK_Coeffs_minus20 = 6×6
   -0.1008    0.5483   -1.0654    0.8384   -0.1690    0.9434
   -0.1223    0.6388   -1.1861    0.8785   -0.1137    0.9156
```

```
   -0.1375    0.6947   -1.2143    0.8132   -0.0880    0.9323
   -0.1194    0.6378   -1.2236    0.9673   -0.2025    0.9677
   -0.1611    0.8511   -1.5734    1.1142   -0.1275    0.9119
   -0.1017    0.5392   -1.0343    0.8124   -0.1396    0.9401
```

```
CD36_GSK_Coeffs_minus30 = CollectCoefficients(CD36_GSK(30:end,1:end),5,6)
```

```
CD36_GSK_Coeffs_minus30 = 6×6
   -0.0460    0.2377   -0.4185    0.2383    0.0636    0.9184
   -0.0487    0.2233   -0.3270    0.0908    0.1867    0.8847
   -0.0510    0.2076   -0.2108   -0.0989    0.2543    0.8970
   -0.0201    0.0825   -0.0851   -0.0702    0.1938    0.9244
   -0.0829    0.4058   -0.6434    0.2541    0.2003    0.8791
   -0.0194    0.0794   -0.0913   -0.0474    0.1887    0.9051
```

```
fyn_GSK_Coeffs_minus10 = CollectCoefficients(fyn_GSK(10:end,1:end),5,6)
```

```
fyn_GSK_Coeffs_minus10 = 6×6
   -0.2160    1.2342   -2.5599    2.2792   -0.7600    0.9027
   -0.3156    1.7181   -3.3416    2.7092   -0.7449    0.9176
   -0.2876    1.5862   -3.1535    2.6680   -0.8357    0.9544
   -0.1556    0.8793   -1.8344    1.6911   -0.6095    0.9637
   -0.2437    1.3544   -2.7224    2.3369   -0.7375    0.9346
   -0.0600    0.3716   -0.8853    0.9571   -0.3416    0.9499
```

```
fyn_GSK_Coeffs_minus20 = CollectCoefficients(fyn_GSK(20:end,1:end),5,6)
```

```
fyn_GSK_Coeffs_minus20 = 6×6
   -0.1295    0.7273   -1.4626    1.2135   -0.3190    0.8475
   -0.2270    1.1868   -2.1723    1.5649   -0.2756    0.8621
   -0.2215    1.1835   -2.2514    1.7660   -0.4544    0.9064
   -0.1119    0.6171   -1.2542    1.1125   -0.3605    0.9304
   -0.1725    0.9287   -1.7851    1.4142   -0.3523    0.8866
    0.0107   -0.0284   -0.0510    0.1739   -0.0241    0.9112
```

```
fyn_GSK_Coeffs_minus30 = CollectCoefficients(fyn_GSK(30:end,1:end),5,6)
```

```
fyn_GSK_Coeffs_minus30 = 6×6
    0.0088   -0.0470    0.1293   -0.2432    0.2412    0.7843
   -0.0524    0.2021   -0.1368   -0.2980    0.4333    0.7853
   -0.0985    0.4818   -0.7828    0.4009    0.0767    0.8462
   -0.0110    0.0505   -0.0848    0.0360    0.0595    0.8812
   -0.0383    0.1718   -0.2188   -0.0261    0.2027    0.8242
    0.1087   -0.5638    1.0223   -0.7855    0.3398    0.8711
```

Ledge Testing for LPS

```
%-------------LEDGE TESTING for LPS

% 100 ug LPS: wt and fyn

wt_Coeffs_lps50_minus10 = CollectCoefficients(wt_lps50(10:end, 1:end), 5,4)
```

```
wt_Coeffs_lps50_minus10 = 4×6
    0.0000   -0.0014    0.0233   -0.1623    0.3480    1.0230
    0.0000   -0.0008    0.0114   -0.0737    0.1949    1.0422
    0.0000   -0.0002    0.0044   -0.0377    0.1245    0.8736
```

```
    0.0000   -0.0000    0.0010   -0.0081    0.0095    0.9041
```

```
wt_Coeffs_lps50_minus20 = CollectCoefficients(wt_lps50(20:end, 1:end), 5,4)
```

```
wt_Coeffs_lps50_minus20 = 4×6
    0.0000   -0.0014    0.0231   -0.1598    0.3378    1.0335
    0.0000   -0.0008    0.0112   -0.0719    0.1883    1.0496
    0.0000   -0.0003    0.0048   -0.0403    0.1311    0.8692
    0.0000   -0.0001    0.0013   -0.0102    0.0155    0.8987
```

```
wt_Coeffs_lps50_minus30 = CollectCoefficients(wt_lps50(30:end, 1:end), 5,4)
```

```
wt_Coeffs_lps50_minus30 = 4×6
    0.0000   -0.0014    0.0228   -0.1572    0.3273    1.0441
    0.0000   -0.0008    0.0109   -0.0698    0.1812    1.0574
    0.0000   -0.0003    0.0052   -0.0431    0.1380    0.8646
    0.0000   -0.0001    0.0017   -0.0125    0.0220    0.8930
```

```
fyn_Coeffs_lps50_minus10 = CollectCoefficients(fyn_lps50(10:end, 1:end), 5,3)
```

```
fyn_Coeffs_lps50_minus10 = 3×6
    0.0000   -0.0012    0.0182   -0.1147    0.2542    1.0170
    0.0000   -0.0009    0.0138   -0.0937    0.2717    0.9415
   -0.0000    0.0000    0.0023   -0.0335    0.1503    1.0539
```

```
fyn_Coeffs_lps50_minus20 = CollectCoefficients(fyn_lps50(20:end, 1:end), 5,3)
```

```
fyn_Coeffs_lps50_minus20 = 3×6
    0.0000   -0.0012    0.0180   -0.1130    0.2475    1.0242
    0.0000   -0.0009    0.0139   -0.0940    0.2707    0.9450
   -0.0000    0.0000    0.0021   -0.0322    0.1452    1.0606
```

```
fyn_Coeffs_lps50_minus30 = CollectCoefficients(fyn_lps50(30:end, 1:end), 5,3)
```

```
fyn_Coeffs_lps50_minus30 = 3×6
    0.0000   -0.0012    0.0178   -0.1112    0.2405    1.0315
    0.0000   -0.0009    0.0139   -0.0942    0.2696    0.9486
   -0.0000    0.0000    0.0019   -0.0307    0.1398    1.0676
```

```
% 50 ug LPS: wt and fyn

wt_Coeffs_minus10 = CollectCoefficients(wt(10:end, 1:end), 5,12)
```

```
wt_Coeffs_minus10 = 12×6
    0.0001   -0.0045    0.0467   -0.1845    0.0943    1.0784
    0.0001   -0.0040    0.0405   -0.1464   -0.0011    1.1505
   -0.0000    0.0003   -0.0012   -0.0047    0.0590    0.9938
   -0.0000    0.0012   -0.0103    0.0281    0.0188    1.0202
   -0.0001    0.0022   -0.0272    0.1540   -0.4057    0.7791
   -0.0000    0.0006   -0.0095    0.0772   -0.3186    0.9050
   -0.0001    0.0034   -0.0407    0.2240   -0.5581    0.8989
   -0.0001    0.0045   -0.0536    0.2886   -0.6819    0.8965
    0.0000   -0.0009    0.0128   -0.0663    0.0249    0.9565
    0.0000   -0.0004    0.0076   -0.0481    0.0261    0.9644
      ⋮
```

```
wt_Coeffs_minus20 = CollectCoefficients(wt(20:end, 1:end), 5,12)
```

```
wt_Coeffs_minus20 = 12×6
```

```
    0.0001   -0.0043    0.0449   -0.1737    0.0693    1.0911
    0.0001   -0.0039    0.0384   -0.1348   -0.0270    1.1627
   -0.0000    0.0003   -0.0011   -0.0051    0.0595    0.9948
   -0.0000    0.0012   -0.0104    0.0285    0.0175    1.0225
   -0.0001    0.0020   -0.0245    0.1394   -0.3706    0.7509
   -0.0000    0.0005   -0.0084    0.0710   -0.3029    0.8893
   -0.0001    0.0033   -0.0392    0.2152   -0.5344    0.8768
   -0.0001    0.0044   -0.0519    0.2783   -0.6538    0.8705
    0.0000   -0.0010    0.0133   -0.0680    0.0275    0.9532
    0.0000   -0.0005    0.0081   -0.0500    0.0292    0.9614
      ⋮
```

```
wt_Coeffs_minus30 = CollectCoefficients(wt(30:end, 1:end), 5,12)
```

```
wt_Coeffs_minus30 = 12×6
    0.0001   -0.0042    0.0431   -0.1635    0.0458    1.1025
    0.0001   -0.0037    0.0362   -0.1226   -0.0539    1.1753
   -0.0000    0.0003   -0.0010   -0.0055    0.0599    0.9959
   -0.0000    0.0012   -0.0105    0.0290    0.0161    1.0248
   -0.0001    0.0018   -0.0216    0.1233   -0.3329    0.7214
   -0.0000    0.0004   -0.0071    0.0639   -0.2853    0.8725
   -0.0001    0.0031   -0.0375    0.2052   -0.5083    0.8534
   -0.0001    0.0042   -0.0500    0.2667   -0.6233    0.8434
    0.0000   -0.0010    0.0137   -0.0699    0.0303    0.9498
    0.0000   -0.0005    0.0085   -0.0519    0.0321    0.9586
      ⋮
```

```
fyn_Coeffs_minus10 = CollectCoefficients(fyn(10:end, 1:end), 5,7)
```

```
fyn_Coeffs_minus10 = 7×6
    0.0001   -0.0044    0.0489   -0.2274    0.3249    1.0020
    0.0004   -0.0128    0.1583   -0.8783    1.9304    0.6737
    0.0003   -0.0109    0.1358   -0.7682    1.7465    0.6864
    0.0003   -0.0113    0.1403   -0.7888    1.7804    0.7613
    0.0004   -0.0116    0.1421   -0.7824    1.7137    0.7695
   -0.0000    0.0009   -0.0068    0.0146    0.0229    0.9832
    0.0000   -0.0003    0.0018   -0.0050    0.0304    1.0091
```

```
fyn_Coeffs_minus20 = CollectCoefficients(fyn(20:end, 1:end), 5,7)
```

```
fyn_Coeffs_minus20 = 7×6
    0.0001   -0.0044    0.0484   -0.2232    0.3123    1.0108
    0.0004   -0.0131    0.1608   -0.8848    1.9253    0.6969
    0.0003   -0.0112    0.1382   -0.7757    1.7453    0.7060
    0.0004   -0.0116    0.1420   -0.7923    1.7705    0.7867
    0.0004   -0.0118    0.1434   -0.7843    1.7011    0.7951
   -0.0000    0.0008   -0.0063    0.0121    0.0279    0.9811
    0.0000   -0.0003    0.0023   -0.0071    0.0348    1.0069
```

```
fyn_Coeffs_minus30 = CollectCoefficients(fyn(30:end, 1:end), 5,7)
```

```
fyn_Coeffs_minus30 = 7×6
    0.0001   -0.0043    0.0478   -0.2188    0.2996    1.0195
    0.0004   -0.0133    0.1629   -0.8899    1.9173    0.7217
    0.0003   -0.0114    0.1404   -0.7820    1.7414    0.7272
    0.0004   -0.0117    0.1433   -0.7940    1.7570    0.8140
    0.0004   -0.0120    0.1445   -0.7848    1.6854    0.8224
   -0.0000    0.0008   -0.0058    0.0096    0.0326    0.9792
    0.0000   -0.0003    0.0027   -0.0093    0.0395    1.0045
```

GOF testing

Now, we compare the GOFs of the various ledges.

GOF testing for WT LPS 100 at various ledge lengths

```
GOF_wt = PolyGOF(WT_GSK, WT_GSK_Coeffs,6)
```

```
GOFMatrix = 1×2
     0     0
pred = 1×840
    0.9898    0.9885    0.9872    0.9859    0.9847    0.9835    0.9822    0.9810 ⋯
observed = 840×1
    1.0131
    1.0132
    1.0131
    1.0127
    1.0124
    1.0125
    1.0127
    1.0125
    1.0128
    1.0124
      ⋮
observed2 = 840×2
    1.0000    1.0131
    1.0000    1.0132
    1.0000    1.0131
    1.0000    1.0127
    1.0000    1.0124
    1.0000    1.0125
    1.0000    1.0127
    1.0000    1.0125
    1.0000    1.0128
    1.0000    1.0124
      ⋮
linreg = 2×1
    0.1853
    0.8108
GOFMatrix = 2×2
         0         0
    0.1853    0.8108
pred = 1×840
    0.9720    0.9706    0.9691    0.9678    0.9664    0.9651    0.9638    0.9625 ⋯
observed = 840×1
    1.0045
    1.0041
    1.0041
    1.0041
    1.0040
    1.0040
    1.0040
    1.0039
    1.0035
    1.0032
```

```
     ⋮
observed2 = 840×2
    1.0000    1.0045
    1.0000    1.0041
    1.0000    1.0041
    1.0000    1.0041
    1.0000    1.0040
    1.0000    1.0040
    1.0000    1.0040
    1.0000    1.0039
    1.0000    1.0035
    1.0000    1.0032
     ⋮
linreg = 2×1
    0.0971
    0.9023
GOFMatrix = 3×2
         0         0
    0.1853    0.8108
    0.0971    0.9023
pred = 1×840
    0.9924    0.9910    0.9895    0.9881    0.9867    0.9854    0.9840    0.9827 ···
observed = 840×1
    1.0274
    1.0276
    1.0276
    1.0272
    1.0271
    1.0266
    1.0265
    1.0262
    1.0263
    1.0262
     ⋮
observed2 = 840×2
    1.0000    1.0274
    1.0000    1.0276
    1.0000    1.0276
    1.0000    1.0272
    1.0000    1.0271
    1.0000    1.0266
    1.0000    1.0265
    1.0000    1.0262
    1.0000    1.0263
    1.0000    1.0262
     ⋮
linreg = 2×1
    0.2388
    0.7591
GOFMatrix = 4×2
         0         0
    0.1853    0.8108
    0.0971    0.9023
    0.2388    0.7591
pred = 1×840
    1.0192    1.0177    1.0162    1.0148    1.0134    1.0120    1.0106    1.0092 ···
observed = 840×1
    1.0443
    1.0439
    1.0438
    1.0438
```

```
    1.0437
    1.0438
    1.0434
    1.0436
    1.0433
    1.0434
      :
      :
observed2 = 840×2
    1.0000    1.0443
    1.0000    1.0439
    1.0000    1.0438
    1.0000    1.0438
    1.0000    1.0437
    1.0000    1.0438
    1.0000    1.0434
    1.0000    1.0436
    1.0000    1.0433
    1.0000    1.0434
      :
      :
linreg = 2×1
    0.2407
    0.7624
GOFMatrix = 5×2
         0         0
    0.1853    0.8108
    0.0971    0.9023
    0.2388    0.7591
    0.2407    0.7624
pred = 1×840
    0.9832    0.9814    0.9797    0.9780    0.9763    0.9747    0.9730    0.9715 ···
observed = 840×1
    1.0244
    1.0243
    1.0243
    1.0243
    1.0245
    1.0246
    1.0248
    1.0255
    1.0256
    1.0253
      :
      :
observed2 = 840×2
    1.0000    1.0244
    1.0000    1.0243
    1.0000    1.0243
    1.0000    1.0243
    1.0000    1.0245
    1.0000    1.0246
    1.0000    1.0248
    1.0000    1.0255
    1.0000    1.0256
    1.0000    1.0253
      :
      :
linreg = 2×1
    0.1891
    0.8088
GOFMatrix = 6×2
         0         0
    0.1853    0.8108
    0.0971    0.9023
```

```
    0.2388    0.7591
    0.2407    0.7624
    0.1891    0.8088
pred = 1×840
    0.9866    0.9854    0.9841    0.9829    0.9817    0.9806    0.9794    0.9783 ⋯
observed = 840×1
    1.0102
    1.0107
    1.0110
    1.0118
    1.0119
    1.0120
    1.0119
    1.0122
    1.0120
    1.0119
      ⋮
observed2 = 840×2
    1.0000    1.0102
    1.0000    1.0107
    1.0000    1.0110
    1.0000    1.0118
    1.0000    1.0119
    1.0000    1.0120
    1.0000    1.0119
    1.0000    1.0122
    1.0000    1.0120
    1.0000    1.0119
      ⋮
linreg = 2×1
    0.1578
    0.8420
GOFMatrix = 7×2
         0         0
    0.1853    0.8108
    0.0971    0.9023
    0.2388    0.7591
    0.2407    0.7624
    0.1891    0.8088
    0.1578    0.8420
GOF_wt = 7×2
         0         0
    0.1853    0.8108
    0.0971    0.9023
    0.2388    0.7591
    0.2407    0.7624
    0.1891    0.8088
    0.1578    0.8420
```

```
GOF_wt2 = PolyGOF(WT_GSK(10:end,1:end), WT_GSK_Coeffs_minus10,6)
```

```
GOFMatrix = 1×2
     0     0
pred = 1×831
    0.9669    0.9660    0.9651    0.9643    0.9635    0.9626    0.9618    0.9610 ⋯
observed = 831×1
    1.0124
    1.0123
    1.0126
    1.0127
    1.0128
    1.0127
```

```
    1.0127
    1.0123
    1.0119
    1.0013
      ⋮
observed2 = 831×2
    1.0000    1.0124
    1.0000    1.0123
    1.0000    1.0126
    1.0000    1.0127
    1.0000    1.0128
    1.0000    1.0127
    1.0000    1.0127
    1.0000    1.0123
    1.0000    1.0119
    1.0000    1.0013
      ⋮
linreg = 2×1
    0.1647
    0.8318
GOFMatrix = 2×2
         0         0
    0.1647    0.8318
pred = 1×831
    0.9441    0.9433    0.9424    0.9416    0.9407    0.9399    0.9391    0.9384 ⋯
observed = 831×1
    1.0032
    1.0031
    1.0031
    1.0025
    1.0021
    1.0021
    1.0025
    1.0025
    1.0021
    1.0000
      ⋮
observed2 = 831×2
    1.0000    1.0032
    1.0000    1.0031
    1.0000    1.0031
    1.0000    1.0025
    1.0000    1.0021
    1.0000    1.0021
    1.0000    1.0025
    1.0000    1.0025
    1.0000    1.0021
    1.0000    1.0000
      ⋮
linreg = 2×1
    0.0832
    0.9162
GOFMatrix = 3×2
         0         0
    0.1647    0.8318
    0.0832    0.9162
pred = 1×831
    0.9632    0.9623    0.9615    0.9606    0.9598    0.9590    0.9582    0.9574 ⋯
observed = 831×1
    1.0262
    1.0263
```

```
    1.0264
    1.0264
    1.0262
    1.0262
    1.0267
    1.0267
    1.0267
    1.0283
      :
      :
observed2 = 831×2
    1.0000    1.0262
    1.0000    1.0263
    1.0000    1.0264
    1.0000    1.0264
    1.0000    1.0262
    1.0000    1.0262
    1.0000    1.0267
    1.0000    1.0267
    1.0000    1.0267
    1.0000    1.0283
      :
      :
linreg = 2×1
    0.2126
    0.7854
GOFMatrix = 4×2
         0         0
    0.1647    0.8318
    0.0832    0.9162
    0.2126    0.7854
pred = 1×831
    0.9938    0.9928    0.9918    0.9908    0.9899    0.9889    0.9880    0.9871 ···
observed = 831×1
    1.0434
    1.0437
    1.0438
    1.0443
    1.0443
    1.0439
    1.0436
    1.0440
    1.0441
    1.0729
      :
      :
observed2 = 831×2
    1.0000    1.0434
    1.0000    1.0437
    1.0000    1.0438
    1.0000    1.0443
    1.0000    1.0443
    1.0000    1.0439
    1.0000    1.0436
    1.0000    1.0440
    1.0000    1.0441
    1.0000    1.0729
      :
      :
linreg = 2×1
    0.2276
    0.7752
GOFMatrix = 5×2
         0         0
    0.1647    0.8318
```

```
    0.0832    0.9162
    0.2126    0.7854
    0.2276    0.7752
pred = 1×831
    0.9480    0.9470    0.9459    0.9449    0.9439    0.9430    0.9420    0.9411 ···
observed = 831×1
    1.0253
    1.0252
    1.0250
    1.0253
    1.0253
    1.0255
    1.0255
    1.0251
    1.0250
    0.9819
      ⋮
observed2 = 831×2
    1.0000    1.0253
    1.0000    1.0252
    1.0000    1.0250
    1.0000    1.0253
    1.0000    1.0253
    1.0000    1.0255
    1.0000    1.0255
    1.0000    1.0251
    1.0000    1.0250
    1.0000    0.9819
      ⋮
linreg = 2×1
    0.1610
    0.8371
GOFMatrix = 6×2
         0         0
    0.1647    0.8318
    0.0832    0.9162
    0.2126    0.7854
    0.2276    0.7752
    0.1610    0.8371
pred = 1×831
    0.9636    0.9628    0.9620    0.9612    0.9604    0.9597    0.9590    0.9582 ···
observed = 831×1
    1.0119
    1.0117
    1.0119
    1.0121
    1.0130
    1.0134
    1.0137
    1.0139
    1.0141
    1.0276
      ⋮
observed2 = 831×2
    1.0000    1.0119
    1.0000    1.0117
    1.0000    1.0119
    1.0000    1.0121
    1.0000    1.0130
    1.0000    1.0134
    1.0000    1.0137
    1.0000    1.0139
```

```
    1.0000    1.0141
    1.0000    1.0276
      ⋮
linreg = 2×1
    0.1460
    0.8539
GOFMatrix = 7×2
         0         0
    0.1647    0.8318
    0.0832    0.9162
    0.2126    0.7854
    0.2276    0.7752
    0.1610    0.8371
    0.1460    0.8539
GOF_wt2 = 7×2
         0         0
    0.1647    0.8318
    0.0832    0.9162
    0.2126    0.7854
    0.2276    0.7752
    0.1610    0.8371
    0.1460    0.8539
```

```
GOF_wt3 = PolyGOF(WT_GSK(20:end,1:end), WT_GSK_Coeffs_minus20,6)
```

```
GOFMatrix = 1×2
     0     0
pred = 1×821
    0.9355    0.9353    0.9351    0.9349    0.9347    0.9345    0.9343    0.9341 ⋯
observed = 821×1
    0.9953
    0.9891
    0.9833
    0.9783
    0.9732
    0.9685
    0.9634
    0.9590
    0.9547
    0.9501
      ⋮
observed2 = 821×2
    1.0000    0.9953
    1.0000    0.9891
    1.0000    0.9833
    1.0000    0.9783
    1.0000    0.9732
    1.0000    0.9685
    1.0000    0.9634
    1.0000    0.9590
    1.0000    0.9547
    1.0000    0.9501
      ⋮
linreg = 2×1
    0.0797
    0.9185
GOFMatrix = 2×2
         0         0
    0.0797    0.9185
pred = 1×821
    0.9064    0.9063    0.9063    0.9062    0.9062    0.9062    0.9062    0.9062 ⋯
```

```
observed = 821×1
    0.9913
    0.9828
    0.9736
    0.9637
    0.9555
    0.9479
    0.9403
    0.9341
    0.9281
    0.9231
      ⋮

observed2 = 821×2
    1.0000    0.9913
    1.0000    0.9828
    1.0000    0.9736
    1.0000    0.9637
    1.0000    0.9555
    1.0000    0.9479
    1.0000    0.9403
    1.0000    0.9341
    1.0000    0.9281
    1.0000    0.9231
      ⋮

linreg = 2×1
    0.0392
    0.9606
GOFMatrix = 3×2
         0         0
    0.0797    0.9185
    0.0392    0.9606
pred = 1×821
    0.9230    0.9230    0.9230    0.9230    0.9230    0.9231    0.9231    0.9231 ⋯
observed = 821×1
    1.0189
    1.0096
    1.0007
    0.9916
    0.9829
    0.9748
    0.9671
    0.9605
    0.9539
    0.9480
      ⋮

observed2 = 821×2
    1.0000    1.0189
    1.0000    1.0096
    1.0000    1.0007
    1.0000    0.9916
    1.0000    0.9829
    1.0000    0.9748
    1.0000    0.9671
    1.0000    0.9605
    1.0000    0.9539
    1.0000    0.9480
      ⋮

linreg = 2×1
    0.1171
    0.8817
GOFMatrix = 4×2
```

```
         0         0
    0.0797    0.9185
    0.0392    0.9606
    0.1171    0.8817
pred = 1×821
    0.9570    0.9567    0.9565    0.9563    0.9561    0.9559    0.9556    0.9555 ···
observed = 821×1
    1.0651
    1.0571
    1.0492
    1.0407
    1.0316
    1.0229
    1.0148
    1.0077
    1.0007
    0.9941
      :
      :
observed2 = 821×2
    1.0000    1.0651
    1.0000    1.0571
    1.0000    1.0492
    1.0000    1.0407
    1.0000    1.0316
    1.0000    1.0229
    1.0000    1.0148
    1.0000    1.0077
    1.0000    1.0007
    1.0000    0.9941
      :
      :
linreg = 2×1
    0.1644
    0.8376
GOFMatrix = 5×2
         0         0
    0.0797    0.9185
    0.0392    0.9606
    0.1171    0.8817
    0.1644    0.8376
pred = 1×821
    0.9011    0.9010    0.9010    0.9010    0.9010    0.9011    0.9011    0.9011 ···
observed = 821×1
    0.9782
    0.9738
    0.9690
    0.9640
    0.9585
    0.9529
    0.9472
    0.9418
    0.9364
    0.9311
      :
      :
observed2 = 821×2
    1.0000    0.9782
    1.0000    0.9738
    1.0000    0.9690
    1.0000    0.9640
    1.0000    0.9585
    1.0000    0.9529
    1.0000    0.9472
    1.0000    0.9418
```

```
    1.0000    0.9364
    1.0000    0.9311
      ⋮

linreg = 2×1
    0.0705
    0.9286
GOFMatrix = 6×2
         0         0
    0.0797    0.9185
    0.0392    0.9606
    0.1171    0.8817
    0.1644    0.8376
    0.0705    0.9286
pred = 1×821
    0.9302    0.9301    0.9300    0.9299    0.9299    0.9298    0.9298    0.9297 ⋯
observed = 821×1
    1.0209
    1.0150
    1.0073
    0.9988
    0.9909
    0.9837
    0.9765
    0.9704
    0.9646
    0.9587
      ⋮

observed2 = 821×2
    1.0000    1.0209
    1.0000    1.0150
    1.0000    1.0073
    1.0000    0.9988
    1.0000    0.9909
    1.0000    0.9837
    1.0000    0.9765
    1.0000    0.9704
    1.0000    0.9646
    1.0000    0.9587
      ⋮

linreg = 2×1
    0.0995
    0.9004
GOFMatrix = 7×2
         0         0
    0.0797    0.9185
    0.0392    0.9606
    0.1171    0.8817
    0.1644    0.8376
    0.0705    0.9286
    0.0995    0.9004
GOF_wt3 = 7×2
         0         0
    0.0797    0.9185
    0.0392    0.9606
    0.1171    0.8817
    0.1644    0.8376
    0.0705    0.9286
    0.0995    0.9004
```

```
GOF_wt4 = PolyGOF(WT_GSK(30:end,1:end), WT_GSK_Coeffs_minus30,6)
```

```
GOFMatrix = 1×2
     0     0
pred = 1×811
    0.9152    0.9155    0.9157    0.9160    0.9163    0.9165    0.9168    0.9171 ···
observed = 811×1
    0.9453
    0.9399
    0.9360
    0.9332
    0.9305
    0.9283
    0.9264
    0.9247
    0.9233
    0.9219
      ⋮
observed2 = 811×2
    1.0000    0.9453
    1.0000    0.9399
    1.0000    0.9360
    1.0000    0.9332
    1.0000    0.9305
    1.0000    0.9283
    1.0000    0.9264
    1.0000    0.9247
    1.0000    0.9233
    1.0000    0.9219
      ⋮
linreg = 2×1
    0.0219
    0.9777
GOFMatrix = 2×2
         0         0
    0.0219    0.9777
pred = 1×811
    0.8822    0.8828    0.8833    0.8839    0.8845    0.8850    0.8856    0.8861 ···
observed = 811×1
    0.9180
    0.9122
    0.9084
    0.9050
    0.9019
    0.8997
    0.8978
    0.8962
    0.8948
    0.8935
      ⋮
observed2 = 811×2
    1.0000    0.9180
    1.0000    0.9122
    1.0000    0.9084
    1.0000    0.9050
    1.0000    0.9019
    1.0000    0.8997
    1.0000    0.8978
    1.0000    0.8962
    1.0000    0.8948
    1.0000    0.8935
      ⋮
```

```
linreg = 2×1
    0.0086
    0.9913
GOFMatrix = 3×2
         0         0
    0.0219    0.9777
    0.0086    0.9913
pred = 1×811
    0.8945    0.8952    0.8959    0.8966    0.8973    0.8980    0.8987    0.8995 ⋯
observed = 811×1
    0.9430
    0.9388
    0.9347
    0.9309
    0.9278
    0.9244
    0.9212
    0.9180
    0.9152
    0.9127
      ⋮
observed2 = 811×2
    1.0000    0.9430
    1.0000    0.9388
    1.0000    0.9347
    1.0000    0.9309
    1.0000    0.9278
    1.0000    0.9244
    1.0000    0.9212
    1.0000    0.9180
    1.0000    0.9152
    1.0000    0.9127
      ⋮
linreg = 2×1
    0.0346
    0.9650
GOFMatrix = 4×2
         0         0
    0.0219    0.9777
    0.0086    0.9913
    0.0346    0.9650
pred = 1×811
    0.9189    0.9195    0.9202    0.9208    0.9215    0.9221    0.9227    0.9233 ⋯
observed = 811×1
    0.9875
    0.9811
    0.9751
    0.9699
    0.9650
    0.9603
    0.9529
    0.9482
    0.9440
    0.9405
      ⋮
observed2 = 811×2
    1.0000    0.9875
    1.0000    0.9811
    1.0000    0.9751
    1.0000    0.9699
    1.0000    0.9650
    1.0000    0.9603
```

```
    1.0000    0.9529
    1.0000    0.9482
    1.0000    0.9440
    1.0000    0.9405
      :
      :
linreg = 2×1
    0.0514
    0.9492
GOFMatrix = 5×2
         0         0
    0.0219    0.9777
    0.0086    0.9913
    0.0346    0.9650
    0.0514    0.9492
pred = 1×811
    0.8744    0.8751    0.8757    0.8764    0.8771    0.8778    0.8784    0.8791 ···
observed = 811×1
    0.9259
    0.9211
    0.9162
    0.9118
    0.9081
    0.9047
    0.9005
    0.8972
    0.8947
    0.8924
      :
      :
observed2 = 811×2
    1.0000    0.9259
    1.0000    0.9211
    1.0000    0.9162
    1.0000    0.9118
    1.0000    0.9081
    1.0000    0.9047
    1.0000    0.9005
    1.0000    0.8972
    1.0000    0.8947
    1.0000    0.8924
      :
      :
linreg = 2×1
    0.0231
    0.9766
GOFMatrix = 6×2
         0         0
    0.0219    0.9777
    0.0086    0.9913
    0.0346    0.9650
    0.0514    0.9492
    0.0231    0.9766
pred = 1×811
    0.9003    0.9009    0.9015    0.9022    0.9028    0.9034    0.9040    0.9046 ···
observed = 811×1
    0.9532
    0.9483
    0.9438
    0.9398
    0.9362
    0.9331
    0.9295
    0.9269
    0.9245
```

```
    0.9219
      ⋮

observed2 = 811×2
    1.0000    0.9532
    1.0000    0.9483
    1.0000    0.9438
    1.0000    0.9398
    1.0000    0.9362
    1.0000    0.9331
    1.0000    0.9295
    1.0000    0.9269
    1.0000    0.9245
    1.0000    0.9219
      ⋮

linreg = 2×1
    0.0396
    0.9604
GOFMatrix = 7×2
         0         0
    0.0219    0.9777
    0.0086    0.9913
    0.0346    0.9650
    0.0514    0.9492
    0.0231    0.9766
    0.0396    0.9604
GOF_wt4 = 7×2
         0         0
    0.0219    0.9777
    0.0086    0.9913
    0.0346    0.9650
    0.0514    0.9492
    0.0231    0.9766
    0.0396    0.9604
```

GOF testing for CD36 GSK response

```
GOF_CD36 = PolyGOF(CD36_GSK, CD36_GSK_Coeffs,6)
```

```
GOFMatrix = 1×2
     0     0
pred = 1×830
    0.9958    0.9943    0.9929    0.9915    0.9901    0.9887    0.9874    0.9860 ⋯
observed = 830×1
    1.0131
    1.0132
    1.0131
    1.0132
    1.0131
    1.0127
    1.0124
    1.0125
    1.0127
    1.0125
      ⋮

observed2 = 830×2
    1.0000    1.0131
    1.0000    1.0132
```

```
    1.0000    1.0131
    1.0000    1.0132
    1.0000    1.0131
    1.0000    1.0127
    1.0000    1.0124
    1.0000    1.0125
    1.0000    1.0127
    1.0000    1.0125
      :
      :
linreg = 2×1
    0.1783
    0.8179
GOFMatrix = 2×2
         0         0
    0.1783    0.8179
pred = 1×830
    0.9788    0.9772    0.9757    0.9741    0.9726    0.9711    0.9697    0.9682 ···
observed = 830×1
    1.0045
    1.0042
    1.0045
    1.0041
    1.0041
    1.0041
    1.0040
    1.0040
    1.0040
    1.0039
      :
      :
observed2 = 830×2
    1.0000    1.0045
    1.0000    1.0042
    1.0000    1.0045
    1.0000    1.0041
    1.0000    1.0041
    1.0000    1.0041
    1.0000    1.0040
    1.0000    1.0040
    1.0000    1.0040
    1.0000    1.0039
      :
      :
linreg = 2×1
    0.0965
    0.9029
GOFMatrix = 3×2
         0         0
    0.1783    0.8179
    0.0965    0.9029
pred = 1×830
    0.9991    0.9975    0.9959    0.9943    0.9928    0.9913    0.9898    0.9884 ···
observed = 830×1
    1.0281
    1.0276
    1.0274
    1.0276
    1.0276
    1.0272
    1.0271
    1.0266
    1.0265
    1.0262
```

```
       :
       :
observed2 = 830×2
    1.0000    1.0281
    1.0000    1.0276
    1.0000    1.0274
    1.0000    1.0276
    1.0000    1.0276
    1.0000    1.0272
    1.0000    1.0271
    1.0000    1.0266
    1.0000    1.0265
    1.0000    1.0262
       :
       :
linreg = 2×1
    0.2391
    0.7588
GOFMatrix = 4×2
         0         0
    0.1783    0.8179
    0.0965    0.9029
    0.2391    0.7588
pred = 1×830
    1.0258    1.0241    1.0225    1.0209    1.0193    1.0178    1.0162    1.0147 ···
observed = 830×1
    1.0441
    1.0444
    1.0443
    1.0439
    1.0438
    1.0438
    1.0437
    1.0438
    1.0434
    1.0436
       :
       :
observed2 = 830×2
    1.0000    1.0441
    1.0000    1.0444
    1.0000    1.0443
    1.0000    1.0439
    1.0000    1.0438
    1.0000    1.0438
    1.0000    1.0437
    1.0000    1.0438
    1.0000    1.0434
    1.0000    1.0436
       :
       :
linreg = 2×1
    0.2373
    0.7656
GOFMatrix = 5×2
         0         0
    0.1783    0.8179
    0.0965    0.9029
    0.2391    0.7588
    0.2373    0.7656
pred = 1×830
    0.9925    0.9905    0.9886    0.9866    0.9847    0.9829    0.9810    0.9792 ···
observed = 830×1
    1.0236
    1.0241
```

```
    1.0244
    1.0243
    1.0243
    1.0243
    1.0245
    1.0246
    1.0248
    1.0255
      :
      :
observed2 = 830×2
    1.0000    1.0236
    1.0000    1.0241
    1.0000    1.0244
    1.0000    1.0243
    1.0000    1.0243
    1.0000    1.0243
    1.0000    1.0245
    1.0000    1.0246
    1.0000    1.0248
    1.0000    1.0255
      :
      :
linreg = 2×1
    0.1806
    0.8173
GOFMatrix = 6×2
         0         0
    0.1783    0.8179
    0.0965    0.9029
    0.2391    0.7588
    0.2373    0.7656
    0.1806    0.8173
pred = 1×830
    0.9931    0.9917    0.9903    0.9889    0.9876    0.9862    0.9849    0.9837 ⋯
observed = 830×1
    1.0101
    1.0102
    1.0102
    1.0107
    1.0110
    1.0118
    1.0119
    1.0120
    1.0119
    1.0122
      :
      :
observed2 = 830×2
    1.0000    1.0101
    1.0000    1.0102
    1.0000    1.0102
    1.0000    1.0107
    1.0000    1.0110
    1.0000    1.0118
    1.0000    1.0119
    1.0000    1.0120
    1.0000    1.0119
    1.0000    1.0122
      :
      :
linreg = 2×1
    0.1519
    0.8480
GOFMatrix = 7×2
```

```
         0         0
    0.1783    0.8179
    0.0965    0.9029
    0.2391    0.7588
    0.2373    0.7656
    0.1806    0.8173
    0.1519    0.8480
GOF_CD36 = 7×2
         0         0
    0.1783    0.8179
    0.0965    0.9029
    0.2391    0.7588
    0.2373    0.7656
    0.1806    0.8173
    0.1519    0.8480
```

```
GOF_CD36_2 = PolyGOF(CD36_GSK(10:end,1:end), CD36_GSK_Coeffs_minus10,6)
```

```
GOFMatrix = 1×2
     0     0
pred = 1×821
    0.9738    0.9727    0.9717    0.9707    0.9697    0.9687    0.9677    0.9668 ···
observed = 821×1
    1.0125
    1.0128
    1.0124
    1.0123
    1.0126
    1.0127
    1.0128
    1.0127
    1.0127
    1.0123
      ⋮
observed2 = 821×2
    1.0000    1.0125
    1.0000    1.0128
    1.0000    1.0124
    1.0000    1.0123
    1.0000    1.0126
    1.0000    1.0127
    1.0000    1.0128
    1.0000    1.0127
    1.0000    1.0127
    1.0000    1.0123
      ⋮
linreg = 2×1
    0.1660
    0.8304
GOFMatrix = 2×2
         0         0
    0.1660    0.8304
pred = 1×821
    0.9520    0.9510    0.9499    0.9489    0.9479    0.9469    0.9459    0.9450 ···
observed = 821×1
    1.0039
    1.0035
    1.0032
    1.0031
    1.0031
    1.0025
    1.0021
```

```
    1.0021
    1.0025
    1.0025
      :
      :
observed2 = 821×2
    1.0000    1.0039
    1.0000    1.0035
    1.0000    1.0032
    1.0000    1.0031
    1.0000    1.0031
    1.0000    1.0025
    1.0000    1.0021
    1.0000    1.0021
    1.0000    1.0025
    1.0000    1.0025
      :
      :
linreg = 2×1
    0.0866
    0.9128
GOFMatrix = 3×2
         0         0
    0.1660    0.8304
    0.0866    0.9128
pred = 1×821
    0.9709    0.9698    0.9688    0.9678    0.9668    0.9658    0.9649    0.9639 ···
observed = 821×1
    1.0262
    1.0263
    1.0262
    1.0263
    1.0264
    1.0264
    1.0262
    1.0262
    1.0267
    1.0267
      :
      :
observed2 = 821×2
    1.0000    1.0262
    1.0000    1.0263
    1.0000    1.0262
    1.0000    1.0263
    1.0000    1.0264
    1.0000    1.0264
    1.0000    1.0262
    1.0000    1.0262
    1.0000    1.0267
    1.0000    1.0267
      :
      :
linreg = 2×1
    0.2208
    0.7772
GOFMatrix = 4×2
         0         0
    0.1660    0.8304
    0.0866    0.9128
    0.2208    0.7772
pred = 1×821
    1.0014    1.0002    0.9990    0.9978    0.9967    0.9956    0.9945    0.9934 ···
observed = 821×1
    1.0436
```

```
    1.0433
    1.0434
    1.0437
    1.0438
    1.0443
    1.0443
    1.0439
    1.0436
    1.0440
      ⋮
observed2 = 821×2
    1.0000    1.0436
    1.0000    1.0433
    1.0000    1.0434
    1.0000    1.0437
    1.0000    1.0438
    1.0000    1.0443
    1.0000    1.0443
    1.0000    1.0439
    1.0000    1.0436
    1.0000    1.0440
      ⋮
linreg = 2×1
    0.2296
    0.7732
GOFMatrix = 5×2
         0         0
    0.1660    0.8304
    0.0866    0.9128
    0.2208    0.7772
    0.2296    0.7732
pred = 1×821
    0.9589    0.9576    0.9563    0.9550    0.9537    0.9525    0.9513    0.9501 ⋯
observed = 821×1
    1.0255
    1.0256
    1.0253
    1.0252
    1.0250
    1.0253
    1.0253
    1.0255
    1.0255
    1.0251
      ⋮
observed2 = 821×2
    1.0000    1.0255
    1.0000    1.0256
    1.0000    1.0253
    1.0000    1.0252
    1.0000    1.0250
    1.0000    1.0253
    1.0000    1.0253
    1.0000    1.0255
    1.0000    1.0255
    1.0000    1.0251
      ⋮
linreg = 2×1
    0.1622
    0.8358
GOFMatrix = 6×2
```

```
         0         0
    0.1660    0.8304
    0.0866    0.9128
    0.2208    0.7772
    0.2296    0.7732
    0.1622    0.8358
pred = 1×821
    0.9712    0.9702    0.9692    0.9682    0.9673    0.9663    0.9654    0.9645 ···
observed = 821×1
    1.0122
    1.0120
    1.0119
    1.0117
    1.0119
    1.0121
    1.0130
    1.0134
    1.0137
    1.0139
      ⋮

observed2 = 821×2
    1.0000    1.0122
    1.0000    1.0120
    1.0000    1.0119
    1.0000    1.0117
    1.0000    1.0119
    1.0000    1.0121
    1.0000    1.0130
    1.0000    1.0134
    1.0000    1.0137
    1.0000    1.0139
      ⋮

linreg = 2×1
    0.1444
    0.8554
GOFMatrix = 7×2
         0         0
    0.1660    0.8304
    0.0866    0.9128
    0.2208    0.7772
    0.2296    0.7732
    0.1622    0.8358
    0.1444    0.8554
GOF_CD36_2 = 7×2
         0         0
    0.1660    0.8304
    0.0866    0.9128
    0.2208    0.7772
    0.2296    0.7732
    0.1622    0.8358
    0.1444    0.8554
```

```
GOF_CD36_3 = PolyGOF(CD36_GSK(20:end,1:end), CD36_GSK_Coeffs_minus20,6)
```

```
GOFMatrix = 1×2
     0     0
pred = 1×811
    0.9430    0.9426    0.9422    0.9418    0.9414    0.9411    0.9407    0.9403 ···
observed = 811×1
    1.0119
    1.0013
    0.9953
```

```
    0.9891
    0.9833
    0.9783
    0.9732
    0.9685
    0.9634
    0.9590
      :
      :
observed2 = 811×2
    1.0000    1.0119
    1.0000    1.0013
    1.0000    0.9953
    1.0000    0.9891
    1.0000    0.9833
    1.0000    0.9783
    1.0000    0.9732
    1.0000    0.9685
    1.0000    0.9634
    1.0000    0.9590
      :
      :
linreg = 2×1
    0.0985
    0.8994
GOFMatrix = 2×2
         0         0
    0.0985    0.8994
pred = 1×811
    0.9153    0.9150    0.9148    0.9145    0.9143    0.9141    0.9138    0.9136 ···
observed = 811×1
    1.0021
    1.0000
    0.9913
    0.9828
    0.9736
    0.9637
    0.9555
    0.9479
    0.9403
    0.9341
      :
      :
observed2 = 811×2
    1.0000    1.0021
    1.0000    1.0000
    1.0000    0.9913
    1.0000    0.9828
    1.0000    0.9736
    1.0000    0.9637
    1.0000    0.9555
    1.0000    0.9479
    1.0000    0.9403
    1.0000    0.9341
      :
      :
linreg = 2×1
    0.0510
    0.9487
GOFMatrix = 3×2
         0         0
    0.0985    0.8994
    0.0510    0.9487
pred = 1×811
    0.9320    0.9318    0.9316    0.9315    0.9313    0.9311    0.9310    0.9308 ···
```

```
observed = 811×1
    1.0267
    1.0283
    1.0189
    1.0096
    1.0007
    0.9916
    0.9829
    0.9748
    0.9671
    0.9605
      ⋮

observed2 = 811×2
    1.0000    1.0267
    1.0000    1.0283
    1.0000    1.0189
    1.0000    1.0096
    1.0000    1.0007
    1.0000    0.9916
    1.0000    0.9829
    1.0000    0.9748
    1.0000    0.9671
    1.0000    0.9605
      ⋮

linreg = 2×1
    0.1449
    0.8537
GOFMatrix = 4×2
         0         0
    0.0985    0.8994
    0.0510    0.9487
    0.1449    0.8537
pred = 1×811
    0.9672    0.9667    0.9662    0.9657    0.9653    0.9648    0.9644    0.9640 ⋯
observed = 811×1
    1.0441
    1.0729
    1.0651
    1.0571
    1.0492
    1.0407
    1.0316
    1.0229
    1.0148
    1.0077
      ⋮

observed2 = 811×2
    1.0000    1.0441
    1.0000    1.0729
    1.0000    1.0651
    1.0000    1.0571
    1.0000    1.0492
    1.0000    1.0407
    1.0000    1.0316
    1.0000    1.0229
    1.0000    1.0148
    1.0000    1.0077
      ⋮

linreg = 2×1
    0.1869
    0.8153
```

```
GOFMatrix = 5×2
         0         0
    0.0985    0.8994
    0.0510    0.9487
    0.1449    0.8537
    0.1869    0.8153
pred = 1×811
    0.9116    0.9113    0.9110    0.9108    0.9105    0.9103    0.9100    0.9098 ···
observed = 811×1
    1.0250
    0.9819
    0.9782
    0.9738
    0.9690
    0.9640
    0.9585
    0.9529
    0.9472
    0.9418
      ⋮
observed2 = 811×2
    1.0000    1.0250
    1.0000    0.9819
    1.0000    0.9782
    1.0000    0.9738
    1.0000    0.9690
    1.0000    0.9640
    1.0000    0.9585
    1.0000    0.9529
    1.0000    0.9472
    1.0000    0.9418
      ⋮
linreg = 2×1
    0.0849
    0.9140
GOFMatrix = 6×2
         0         0
    0.0985    0.8994
    0.0510    0.9487
    0.1449    0.8537
    0.1869    0.8153
    0.0849    0.9140
pred = 1×811
    0.9397    0.9394    0.9391    0.9387    0.9384    0.9382    0.9379    0.9376 ···
observed = 811×1
    1.0141
    1.0276
    1.0209
    1.0150
    1.0073
    0.9988
    0.9909
    0.9837
    0.9765
    0.9704
      ⋮
observed2 = 811×2
    1.0000    1.0141
    1.0000    1.0276
    1.0000    1.0209
    1.0000    1.0150
    1.0000    1.0073
```

```
    1.0000    0.9988
    1.0000    0.9909
    1.0000    0.9837
    1.0000    0.9765
    1.0000    0.9704
      ⋮
linreg = 2×1
    0.1111
    0.8888
GOFMatrix = 7×2
         0         0
    0.0985    0.8994
    0.0510    0.9487
    0.1449    0.8537
    0.1869    0.8153
    0.0849    0.9140
    0.1111    0.8888
GOF_CD36_3 = 7×2
         0         0
    0.0985    0.8994
    0.0510    0.9487
    0.1449    0.8537
    0.1869    0.8153
    0.0849    0.9140
    0.1111    0.8888
```

```
GOF_CD36_4 = PolyGOF(CD36_GSK(30:end,1:end), CD36_GSK_Coeffs_minus30,6)
```

```
GOFMatrix = 1×2
     0     0
pred = 1×801
    0.9186    0.9187    0.9189    0.9191    0.9193    0.9194    0.9196    0.9198 ⋯
observed = 801×1
    0.9547
    0.9501
    0.9453
    0.9399
    0.9360
    0.9332
    0.9305
    0.9283
    0.9264
    0.9247
      ⋮
observed2 = 801×2
    1.0000    0.9547
    1.0000    0.9501
    1.0000    0.9453
    1.0000    0.9399
    1.0000    0.9360
    1.0000    0.9332
    1.0000    0.9305
    1.0000    0.9283
    1.0000    0.9264
    1.0000    0.9247
      ⋮
linreg = 2×1
    0.0264
    0.9730
GOFMatrix = 2×2
         0         0
```

```
    0.0264    0.9730
pred = 1×801
    0.8852    0.8857    0.8861    0.8866    0.8871    0.8876    0.8880    0.8885 ⋯
observed = 801×1
    0.9281
    0.9231
    0.9180
    0.9122
    0.9084
    0.9050
    0.9019
    0.8997
    0.8978
    0.8962
      ⋮
observed2 = 801×2
    1.0000    0.9281
    1.0000    0.9231
    1.0000    0.9180
    1.0000    0.9122
    1.0000    0.9084
    1.0000    0.9050
    1.0000    0.9019
    1.0000    0.8997
    1.0000    0.8978
    1.0000    0.8962
      ⋮
linreg = 2×1
    0.0112
    0.9887
GOFMatrix = 3×2
         0         0
    0.0264    0.9730
    0.0112    0.9887
pred = 1×801
    0.8976    0.8983    0.8989    0.8995    0.9002    0.9008    0.9014    0.9020 ⋯
observed = 801×1
    0.9539
    0.9480
    0.9430
    0.9388
    0.9347
    0.9309
    0.9278
    0.9244
    0.9212
    0.9180
      ⋮
observed2 = 801×2
    1.0000    0.9539
    1.0000    0.9480
    1.0000    0.9430
    1.0000    0.9388
    1.0000    0.9347
    1.0000    0.9309
    1.0000    0.9278
    1.0000    0.9244
    1.0000    0.9212
    1.0000    0.9180
      ⋮
linreg = 2×1
```

```
    0.0422
    0.9574
GOFMatrix = 4×2
         0         0
    0.0264    0.9730
    0.0112    0.9887
    0.0422    0.9574
pred = 1×801
    0.9249    0.9254    0.9259    0.9264    0.9268    0.9273    0.9278    0.9283 ⋯
observed = 801×1
    1.0007
    0.9941
    0.9875
    0.9811
    0.9751
    0.9699
    0.9650
    0.9603
    0.9529
    0.9482
      ⋮
observed2 = 801×2
    1.0000    1.0007
    1.0000    0.9941
    1.0000    0.9875
    1.0000    0.9811
    1.0000    0.9751
    1.0000    0.9699
    1.0000    0.9650
    1.0000    0.9603
    1.0000    0.9529
    1.0000    0.9482
      ⋮
linreg = 2×1
    0.0664
    0.9343
GOFMatrix = 5×2
         0         0
    0.0264    0.9730
    0.0112    0.9887
    0.0422    0.9574
    0.0664    0.9343
pred = 1×801
    0.8796    0.8801    0.8806    0.8811    0.8816    0.8821    0.8826    0.8832 ⋯
observed = 801×1
    0.9364
    0.9311
    0.9259
    0.9211
    0.9162
    0.9118
    0.9081
    0.9047
    0.9005
    0.8972
      ⋮
observed2 = 801×2
    1.0000    0.9364
    1.0000    0.9311
    1.0000    0.9259
    1.0000    0.9211
    1.0000    0.9162
```

```
    1.0000    0.9118
    1.0000    0.9081
    1.0000    0.9047
    1.0000    0.9005
    1.0000    0.8972
      :
      :
linreg = 2×1
    0.0266
    0.9731
GOFMatrix = 6×2
         0         0
    0.0264    0.9730
    0.0112    0.9887
    0.0422    0.9574
    0.0664    0.9343
    0.0266    0.9731
pred = 1×801
    0.9055    0.9060    0.9065    0.9069    0.9074    0.9079    0.9083    0.9088 ···
observed = 801×1
    0.9646
    0.9587
    0.9532
    0.9483
    0.9438
    0.9398
    0.9362
    0.9331
    0.9295
    0.9269
      :
      :
observed2 = 801×2
    1.0000    0.9646
    1.0000    0.9587
    1.0000    0.9532
    1.0000    0.9483
    1.0000    0.9438
    1.0000    0.9398
    1.0000    0.9362
    1.0000    0.9331
    1.0000    0.9295
    1.0000    0.9269
      :
      :
linreg = 2×1
    0.0447
    0.9552
GOFMatrix = 7×2
         0         0
    0.0264    0.9730
    0.0112    0.9887
    0.0422    0.9574
    0.0664    0.9343
    0.0266    0.9731
    0.0447    0.9552
GOF_CD36_4 = 7×2
         0         0
    0.0264    0.9730
    0.0112    0.9887
    0.0422    0.9574
    0.0664    0.9343
    0.0266    0.9731
    0.0447    0.9552
```

GOF testing for Fyn GSK response

```
GOF_Fyn = PolyGOF(fyn_GSK, fyn_GSK_Coeffs,6)
```

```
GOFMatrix = 1×2
     0     0
pred = 1×836
    0.9396    0.9371    0.9346    0.9321    0.9297    0.9273    0.9249    0.9226 ···
observed = 836×1
    0.9711
    0.9708
    0.9707
    0.9707
    0.9704
    0.9703
    0.9700
    0.9697
    0.9695
    0.9694
      ⋮
observed2 = 836×2
    1.0000    0.9711
    1.0000    0.9708
    1.0000    0.9707
    1.0000    0.9707
    1.0000    0.9704
    1.0000    0.9703
    1.0000    0.9700
    1.0000    0.9697
    1.0000    0.9695
    1.0000    0.9694
      ⋮
linreg = 2×1
    0.4613
    0.4638
GOFMatrix = 2×2
         0         0
    0.4613    0.4638
pred = 1×836
    0.9549    0.9523    0.9498    0.9474    0.9449    0.9425    0.9402    0.9379 ···
observed = 836×1
    0.9873
    0.9871
    0.9873
    0.9873
    0.9869
    0.9863
    0.9861
    0.9858
    0.9860
    0.9862
      ⋮
observed2 = 836×2
    1.0000    0.9873
    1.0000    0.9871
    1.0000    0.9873
    1.0000    0.9873
```

```
    1.0000    0.9869
    1.0000    0.9863
    1.0000    0.9861
    1.0000    0.9858
    1.0000    0.9860
    1.0000    0.9862
      :
      :
linreg = 2×1
    0.4191
    0.5431
GOFMatrix = 3×2
         0         0
    0.4613    0.4638
    0.4191    0.5431
pred = 1×836
    0.9859    0.9832    0.9807    0.9781    0.9756    0.9731    0.9707    0.9683 ···
observed = 836×1
    1.0035
    1.0032
    1.0031
    1.0026
    1.0024
    1.0019
    1.0021
    1.0022
    1.0017
    1.0017
      :
      :
observed2 = 836×2
    1.0000    1.0035
    1.0000    1.0032
    1.0000    1.0031
    1.0000    1.0026
    1.0000    1.0024
    1.0000    1.0019
    1.0000    1.0021
    1.0000    1.0022
    1.0000    1.0017
    1.0000    1.0017
      :
      :
linreg = 2×1
    0.3957
    0.5658
GOFMatrix = 4×2
         0         0
    0.4613    0.4638
    0.4191    0.5431
    0.3957    0.5658
pred = 1×836
    0.9854    0.9835    0.9817    0.9798    0.9781    0.9763    0.9745    0.9728 ···
observed = 836×1
    0.9958
    0.9960
    0.9961
    0.9960
    0.9957
    0.9950
    0.9952
    0.9948
    0.9947
    0.9945
```

```
      :
      :
observed2 = 836×2
    1.0000    0.9958
    1.0000    0.9960
    1.0000    0.9961
    1.0000    0.9960
    1.0000    0.9957
    1.0000    0.9950
    1.0000    0.9952
    1.0000    0.9948
    1.0000    0.9947
    1.0000    0.9945
      :
      :
linreg = 2×1
    0.4060
    0.5607
GOFMatrix = 5×2
         0         0
    0.4613    0.4638
    0.4191    0.5431
    0.3957    0.5658
    0.4060    0.5607
pred = 1×836
    0.9664    0.9640    0.9617    0.9594    0.9571    0.9548    0.9526    0.9504 ···
observed = 836×1
    0.9894
    0.9893
    0.9893
    0.9891
    0.9889
    0.9884
    0.9883
    0.9881
    0.9880
    0.9880
      :
      :
observed2 = 836×2
    1.0000    0.9894
    1.0000    0.9893
    1.0000    0.9893
    1.0000    0.9891
    1.0000    0.9889
    1.0000    0.9884
    1.0000    0.9883
    1.0000    0.9881
    1.0000    0.9880
    1.0000    0.9880
      :
      :
linreg = 2×1
    0.4418
    0.5109
GOFMatrix = 6×2
         0         0
    0.4613    0.4638
    0.4191    0.5431
    0.3957    0.5658
    0.4060    0.5607
    0.4418    0.5109
pred = 1×836
    0.9757    0.9744    0.9731    0.9718    0.9705    0.9693    0.9680    0.9668 ···
observed = 836×1
```

```
    1.0070
    1.0070
    1.0068
    1.0067
    1.0067
    1.0067
    1.0068
    1.0066
    1.0067
    1.0070
      ⋮

observed2 = 836×2
    1.0000    1.0070
    1.0000    1.0070
    1.0000    1.0068
    1.0000    1.0067
    1.0000    1.0067
    1.0000    1.0067
    1.0000    1.0068
    1.0000    1.0066
    1.0000    1.0067
    1.0000    1.0070
      ⋮

linreg = 2×1
    0.1012
    0.8973
GOFMatrix = 7×2
         0         0
    0.4613    0.4638
    0.4191    0.5431
    0.3957    0.5658
    0.4060    0.5607
    0.4418    0.5109
    0.1012    0.8973
GOF_Fyn = 7×2
         0         0
    0.4613    0.4638
    0.4191    0.5431
    0.3957    0.5658
    0.4060    0.5607
    0.4418    0.5109
    0.1012    0.8973
```

```
GOF_Fyn_2 = PolyGOF(fyn_GSK(10:end,1:end), fyn_GSK_Coeffs_minus10,6)
```

```
GOFMatrix = 1×2
     0     0
pred = 1×827
    0.9009    0.8990    0.8972    0.8954    0.8936    0.8918    0.8901    0.8884 ⋯
observed = 827×1
    0.9694
    0.9696
    0.9701
    0.9701
    0.9698
    0.9692
    0.9684
    0.9682
    0.9681
    0.9679
      ⋮
```

```
observed2 = 827×2
    1.0000    0.9694
    1.0000    0.9696
    1.0000    0.9701
    1.0000    0.9701
    1.0000    0.9698
    1.0000    0.9692
    1.0000    0.9684
    1.0000    0.9682
    1.0000    0.9681
    1.0000    0.9679
      ⋮
linreg = 2×1
    0.5102
    0.4061
GOFMatrix = 2×2
         0         0
    0.5102    0.4061
pred = 1×827
    0.9158    0.9140    0.9122    0.9104    0.9087    0.9070    0.9054    0.9038 ⋯
observed = 827×1
    0.9862
    0.9864
    0.9869
    0.9864
    0.9864
    0.9864
    0.9860
    0.9857
    0.9858
    0.9854
      ⋮
observed2 = 827×2
    1.0000    0.9862
    1.0000    0.9864
    1.0000    0.9869
    1.0000    0.9864
    1.0000    0.9864
    1.0000    0.9864
    1.0000    0.9860
    1.0000    0.9857
    1.0000    0.9858
    1.0000    0.9854
      ⋮
linreg = 2×1
    0.4163
    0.5458
GOFMatrix = 3×2
         0         0
    0.5102    0.4061
    0.4163    0.5458
pred = 1×827
    0.9524    0.9503    0.9483    0.9463    0.9444    0.9425    0.9406    0.9388 ⋯
observed = 827×1
    1.0017
    1.0012
    1.0014
    1.0013
    1.0020
    1.0022
    1.0026
    1.0027
```

```
    1.0027
    1.0031
      :
      :
observed2 = 827×2
    1.0000    1.0017
    1.0000    1.0012
    1.0000    1.0014
    1.0000    1.0013
    1.0000    1.0020
    1.0000    1.0022
    1.0000    1.0026
    1.0000    1.0027
    1.0000    1.0027
    1.0000    1.0031
      :
      :
linreg = 2×1
    0.4329
    0.5244
GOFMatrix = 4×2
         0         0
    0.5102    0.4061
    0.4163    0.5458
    0.4329    0.5244
pred = 1×827
    0.9622    0.9607    0.9592    0.9577    0.9563    0.9549    0.9535    0.9521 ···
observed = 827×1
    0.9945
    0.9950
    0.9954
    0.9946
    0.9948
    0.9944
    0.9940
    0.9935
    0.9937
    0.9937
      :
      :
observed2 = 827×2
    1.0000    0.9945
    1.0000    0.9950
    1.0000    0.9954
    1.0000    0.9946
    1.0000    0.9948
    1.0000    0.9944
    1.0000    0.9940
    1.0000    0.9935
    1.0000    0.9937
    1.0000    0.9937
      :
      :
linreg = 2×1
    0.4436
    0.5197
GOFMatrix = 5×2
         0         0
    0.5102    0.4061
    0.4163    0.5458
    0.4329    0.5244
    0.4436    0.5197
pred = 1×827
    0.9328    0.9310    0.9292    0.9275    0.9258    0.9241    0.9224    0.9208 ···
observed = 827×1
```

```
    0.9880
    0.9881
    0.9885
    0.9881
    0.9882
    0.9881
    0.9878
    0.9875
    0.9876
    0.9875
      ⋮
observed2 = 827×2
    1.0000    0.9880
    1.0000    0.9881
    1.0000    0.9885
    1.0000    0.9881
    1.0000    0.9882
    1.0000    0.9881
    1.0000    0.9878
    1.0000    0.9875
    1.0000    0.9876
    1.0000    0.9875
      ⋮
linreg = 2×1
    0.4689
    0.4804
GOFMatrix = 6×2
         0         0
    0.5102    0.4061
    0.4163    0.5458
    0.4329    0.5244
    0.4436    0.5197
    0.4689    0.4804
pred = 1×827
    0.9491    0.9482    0.9474    0.9466    0.9458    0.9450    0.9442    0.9435 ⋯
observed = 827×1
    1.0070
    1.0071
    1.0073
    1.0073
    1.0077
    1.0078
    1.0082
    1.0084
    1.0084
    1.0084
      ⋮
observed2 = 827×2
    1.0000    1.0070
    1.0000    1.0071
    1.0000    1.0073
    1.0000    1.0073
    1.0000    1.0077
    1.0000    1.0078
    1.0000    1.0082
    1.0000    1.0084
    1.0000    1.0084
    1.0000    1.0084
      ⋮
linreg = 2×1
    0.0927
```

```
    0.9059
GOFMatrix = 7×2
         0         0
    0.5102    0.4061
    0.4163    0.5458
    0.4329    0.5244
    0.4436    0.5197
    0.4689    0.4804
    0.0927    0.9059
GOF_Fyn_2 = 7×2
         0         0
    0.5102    0.4061
    0.4163    0.5458
    0.4329    0.5244
    0.4436    0.5197
    0.4689    0.4804
    0.0927    0.9059
```

```
GOF_Fyn_3 = PolyGOF(fyn_GSK(20:end,1:end), fyn_GSK_Coeffs_minus20,6)
```

```
GOFMatrix = 1×2
     0     0
pred = 1×817
    0.8467    0.8459    0.8452    0.8444    0.8437    0.8430    0.8423    0.8416 ⋯
observed = 817×1
    0.9678
    1.0154
    0.9941
    0.9774
    0.9625
    0.9476
    0.9337
    0.9210
    0.9101
    0.8999
      ⋮
observed2 = 817×2
    1.0000    0.9678
    1.0000    1.0154
    1.0000    0.9941
    1.0000    0.9774
    1.0000    0.9625
    1.0000    0.9476
    1.0000    0.9337
    1.0000    0.9210
    1.0000    0.9101
    1.0000    0.8999
      ⋮
linreg = 2×1
    0.4811
    0.4391
GOFMatrix = 2×2
         0         0
    0.4811    0.4391
pred = 1×817
    0.8614    0.8607    0.8601    0.8595    0.8589    0.8583    0.8577    0.8572 ⋯
observed = 817×1
    0.9850
    1.0544
    1.0367
    1.0236
    1.0125
```

```
    1.0009
    0.9886
    0.9755
    0.9612
    0.9442
      ⋮
observed2 = 817×2
    1.0000    0.9850
    1.0000    1.0544
    1.0000    1.0367
    1.0000    1.0236
    1.0000    1.0125
    1.0000    1.0009
    1.0000    0.9886
    1.0000    0.9755
    1.0000    0.9612
    1.0000    0.9442
      ⋮
linreg = 2×1
    0.3573
    0.6098
GOFMatrix = 3×2
         0         0
    0.4811    0.4391
    0.3573    0.6098
pred = 1×817
    0.9052    0.9041    0.9030    0.9020    0.9009    0.8999    0.8989    0.8980 ⋯
observed = 817×1
    1.0032
    1.0457
    1.0279
    1.0161
    1.0070
    0.9997
    0.9910
    0.9806
    0.9630
    0.9508
      ⋮
observed2 = 817×2
    1.0000    1.0032
    1.0000    1.0457
    1.0000    1.0279
    1.0000    1.0161
    1.0000    1.0070
    1.0000    0.9997
    1.0000    0.9910
    1.0000    0.9806
    1.0000    0.9630
    1.0000    0.9508
      ⋮
linreg = 2×1
    0.4082
    0.5510
GOFMatrix = 4×2
         0         0
    0.4811    0.4391
    0.3573    0.6098
    0.4082    0.5510
pred = 1×817
    0.9295    0.9286    0.9278    0.9269    0.9261    0.9252    0.9244    0.9236 ⋯
```

```
observed = 817×1
    0.9940
    1.0465
    1.0363
    1.0258
    1.0150
    1.0061
    0.9984
    0.9910
    0.9795
    0.9700
      ⋮
observed2 = 817×2
    1.0000    0.9940
    1.0000    1.0465
    1.0000    1.0363
    1.0000    1.0258
    1.0000    1.0150
    1.0000    1.0061
    1.0000    0.9984
    1.0000    0.9910
    1.0000    0.9795
    1.0000    0.9700
      ⋮
linreg = 2×1
    0.4464
    0.5163
GOFMatrix = 5×2
         0         0
    0.4811    0.4391
    0.3573    0.6098
    0.4082    0.5510
    0.4464    0.5163
pred = 1×817
    0.8857    0.8848    0.8840    0.8832    0.8824    0.8816    0.8808    0.8801 ⋯
observed = 817×1
    0.9875
    1.0405
    1.0237
    1.0107
    0.9993
    0.9885
    0.9779
    0.9670
    0.9534
    0.9412
      ⋮
observed2 = 817×2
    1.0000    0.9875
    1.0000    1.0405
    1.0000    1.0237
    1.0000    1.0107
    1.0000    0.9993
    1.0000    0.9885
    1.0000    0.9779
    1.0000    0.9670
    1.0000    0.9534
    1.0000    0.9412
      ⋮
linreg = 2×1
    0.4311
```

```
    0.5217
GOFMatrix = 6×2
         0         0
    0.4811    0.4391
    0.3573    0.6098
    0.4082    0.5510
    0.4464    0.5163
    0.4311    0.5217
pred = 1×817
    0.9111    0.9111    0.9110    0.9110    0.9109    0.9109    0.9108    0.9108 ···
observed = 817×1
    1.0084
    1.0084
    1.0084
    1.0088
    1.0201
    1.0000
    0.9814
    0.9644
    0.9485
    0.9338
      ⋮
observed2 = 817×2
    1.0000    1.0084
    1.0000    1.0084
    1.0000    1.0084
    1.0000    1.0088
    1.0000    1.0201
    1.0000    1.0000
    1.0000    0.9814
    1.0000    0.9644
    1.0000    0.9485
    1.0000    0.9338
      ⋮
linreg = 2×1
    0.0630
    0.9360
GOFMatrix = 7×2
         0         0
    0.4811    0.4391
    0.3573    0.6098
    0.4082    0.5510
    0.4464    0.5163
    0.4311    0.5217
    0.0630    0.9360
GOF_Fyn_3 = 7×2
         0         0
    0.4811    0.4391
    0.3573    0.6098
    0.4082    0.5510
    0.4464    0.5163
    0.4311    0.5217
    0.0630    0.9360
```

```
GOF_Fyn_4 = PolyGOF(fyn_GSK(30:end,1:end), fyn_GSK_Coeffs_minus30,6)
```

```
GOFMatrix = 1×2
     0     0
pred = 1×807
    0.7849    0.7855    0.7861    0.7867    0.7873    0.7879    0.7885    0.7890 ···
observed = 807×1
    0.8898
```

```
    0.8798
    0.8694
    0.8593
    0.8508
    0.8431
    0.8359
    0.8289
    0.8224
    0.8168
      :
      :
observed2 = 807×2
    1.0000    0.8898
    1.0000    0.8798
    1.0000    0.8694
    1.0000    0.8593
    1.0000    0.8508
    1.0000    0.8431
    1.0000    0.8359
    1.0000    0.8289
    1.0000    0.8224
    1.0000    0.8168
      :
      :
linreg = 2×1
    0.1885
    0.7799
GOFMatrix = 2×2
         0         0
    0.1885    0.7799
pred = 1×807
    0.7864    0.7874    0.7885    0.7896    0.7907    0.7917    0.7928    0.7938 ···
observed = 807×1
    0.9280
    0.9125
    0.8988
    0.8836
    0.8707
    0.8597
    0.8482
    0.8383
    0.8280
    0.8185
      :
      :
observed2 = 807×2
    1.0000    0.9280
    1.0000    0.9125
    1.0000    0.8988
    1.0000    0.8836
    1.0000    0.8707
    1.0000    0.8597
    1.0000    0.8482
    1.0000    0.8383
    1.0000    0.8280
    1.0000    0.8185
      :
      :
linreg = 2×1
    0.1087
    0.8811
GOFMatrix = 3×2
         0         0
    0.1885    0.7799
    0.1087    0.8811
```

```
pred = 1×807
    0.8464    0.8466    0.8468    0.8470    0.8472    0.8475    0.8477    0.8479 ⋯
observed = 807×1
    0.9386
    0.9271
    0.9171
    0.9084
    0.9002
    0.8921
    0.8848
    0.8780
    0.8713
    0.8650
      ⋮
observed2 = 807×2
    1.0000    0.9386
    1.0000    0.9271
    1.0000    0.9171
    1.0000    0.9084
    1.0000    0.9002
    1.0000    0.8921
    1.0000    0.8848
    1.0000    0.8780
    1.0000    0.8713
    1.0000    0.8650
      ⋮
linreg = 2×1
    0.1308
    0.8560
GOFMatrix = 4×2
         0         0
    0.1885    0.7799
    0.1087    0.8811
    0.1308    0.8560
pred = 1×807
    0.8813    0.8815    0.8816    0.8818    0.8819    0.8821    0.8822    0.8824 ⋯
observed = 807×1
    0.9630
    0.9543
    0.9471
    0.9400
    0.9332
    0.9271
    0.9214
    0.9158
    0.9104
    0.9053
      ⋮
observed2 = 807×2
    1.0000    0.9630
    1.0000    0.9543
    1.0000    0.9471
    1.0000    0.9400
    1.0000    0.9332
    1.0000    0.9271
    1.0000    0.9214
    1.0000    0.9158
    1.0000    0.9104
    1.0000    0.9053
      ⋮
linreg = 2×1
```

```
    0.2036
    0.7791
GOFMatrix = 5×2
         0         0
    0.1885    0.7799
    0.1087    0.8811
    0.1308    0.8560
    0.2036    0.7791
pred = 1×807
    0.8248    0.8253    0.8258    0.8263    0.8268    0.8273    0.8278    0.8283 ⋯
observed = 807×1
    0.9298
    0.9184
    0.9081
    0.8979
    0.8887
    0.8805
    0.8726
    0.8653
    0.8580
    0.8514
      ⋮
observed2 = 807×2
    1.0000    0.9298
    1.0000    0.9184
    1.0000    0.9081
    1.0000    0.8979
    1.0000    0.8887
    1.0000    0.8805
    1.0000    0.8726
    1.0000    0.8653
    1.0000    0.8580
    1.0000    0.8514
      ⋮
linreg = 2×1
    0.1478
    0.8358
GOFMatrix = 6×2
         0         0
    0.1885    0.7799
    0.1087    0.8811
    0.1308    0.8560
    0.2036    0.7791
    0.1478    0.8358
pred = 1×807
    0.8720    0.8728    0.8737    0.8745    0.8753    0.8761    0.8769    0.8776 ⋯
observed = 807×1
    0.9203
    0.9090
    0.8989
    0.8903
    0.8829
    0.8771
    0.8726
    0.8692
    0.8671
    0.8658
      ⋮
observed2 = 807×2
    1.0000    0.9203
    1.0000    0.9090
    1.0000    0.8989
```

```
    1.0000    0.8903
    1.0000    0.8829
    1.0000    0.8771
    1.0000    0.8726
    1.0000    0.8692
    1.0000    0.8671
    1.0000    0.8658
      :
linreg = 2×1
    0.0121
    0.9877
GOFMatrix = 7×2
         0         0
    0.1885    0.7799
    0.1087    0.8811
    0.1308    0.8560
    0.2036    0.7791
    0.1478    0.8358
    0.0121    0.9877
GOF_Fyn_4 = 7×2
         0         0
    0.1885    0.7799
    0.1087    0.8811
    0.1308    0.8560
    0.2036    0.7791
    0.1478    0.8358
    0.0121    0.9877
```

GOF Testing for wt and Fyn lps responses

```
GOF_wtlps = PolyGOF(wt, WTCoeffs,12)
```

```
GOFMatrix = 1×2
     0     0
pred = 1×4668
    1.0672    1.0675    1.0678    1.0681    1.0684    1.0686    1.0689    1.0692 ···
observed = 4668×1
    0.9432
    0.9431
    0.9432
    0.9426
    0.9422
    0.9422
    0.9424
    0.9419
    0.9425
    0.9436
      :
observed2 = 4668×2
    1.0000    0.9432
    1.0000    0.9431
    1.0000    0.9432
    1.0000    0.9426
    1.0000    0.9422
    1.0000    0.9422
    1.0000    0.9424
    1.0000    0.9419
    1.0000    0.9425
```

```
    1.0000    0.9436
      :
      :
linreg = 2×1
    0.0322
    0.9448
GOFMatrix = 2×2
         0         0
    0.0322    0.9448
pred = 1×4668
    1.1397    1.1398    1.1398    1.1399    1.1399    1.1399    1.1400    1.1400 ···
observed = 4668×1
    0.9930
    0.9923
    0.9919
    0.9922
    0.9926
    0.9927
    0.9922
    0.9922
    0.9929
    0.9936
      :
      :
observed2 = 4668×2
    1.0000    0.9930
    1.0000    0.9923
    1.0000    0.9919
    1.0000    0.9922
    1.0000    0.9926
    1.0000    0.9927
    1.0000    0.9922
    1.0000    0.9922
    1.0000    0.9929
    1.0000    0.9936
      :
      :
linreg = 2×1
    0.0510
    0.9143
GOFMatrix = 3×2
         0         0
    0.0322    0.9448
    0.0510    0.9143
pred = 1×4668
    0.9930    0.9931    0.9933    0.9934    0.9935    0.9937    0.9938    0.9940 ···
observed = 4668×1
    0.9967
    0.9970
    0.9974
    0.9980
    0.9982
    0.9981
    0.9982
    0.9986
    0.9992
    0.9988
      :
      :
observed2 = 4668×2
    1.0000    0.9967
    1.0000    0.9970
    1.0000    0.9974
    1.0000    0.9980
    1.0000    0.9982
```

```
    1.0000    0.9981
    1.0000    0.9982
    1.0000    0.9986
    1.0000    0.9992
    1.0000    0.9988
      :
      :
linreg = 2×1
    0.0235
    0.9804
GOFMatrix = 4×2
         0         0
    0.0322    0.9448
    0.0510    0.9143
    0.0235    0.9804
pred = 1×4668
    1.0184    1.0184    1.0185    1.0185    1.0186    1.0186    1.0187    1.0187 ···
observed = 4668×1
    0.9972
    0.9975
    0.9975
    0.9978
    0.9975
    0.9976
    0.9979
    0.9978
    0.9978
    0.9980
      :
      :
observed2 = 4668×2
    1.0000    0.9972
    1.0000    0.9975
    1.0000    0.9975
    1.0000    0.9978
    1.0000    0.9975
    1.0000    0.9976
    1.0000    0.9979
    1.0000    0.9978
    1.0000    0.9978
    1.0000    0.9980
      :
      :
linreg = 2×1
    0.1507
    0.8672
GOFMatrix = 5×2
         0         0
    0.0322    0.9448
    0.0510    0.9143
    0.0235    0.9804
    0.1507    0.8672
pred = 1×4668
    0.8023    0.8012    0.8001    0.7991    0.7980    0.7969    0.7958    0.7948 ···
observed = 4668×1
    1.0041
    1.0040
    1.0038
    1.0038
    1.0032
    1.0034
    1.0034
    1.0041
    1.0039
    1.0037
```

```
      ⋮
observed2 = 4668×2
    1.0000    1.0041
    1.0000    1.0040
    1.0000    1.0038
    1.0000    1.0038
    1.0000    1.0032
    1.0000    1.0034
    1.0000    1.0034
    1.0000    1.0041
    1.0000    1.0039
    1.0000    1.0037
      ⋮
linreg = 2×1
    0.0466
    0.8802
GOFMatrix = 6×2
         0         0
    0.0322    0.9448
    0.0510    0.9143
    0.0235    0.9804
    0.1507    0.8672
    0.0466    0.8802
pred = 1×4668
    0.9177    0.9169    0.9161    0.9152    0.9144    0.9136    0.9128    0.9119 ···
observed = 4668×1
    1.0008
    1.0004
    1.0008
    1.0006
    1.0001
    1.0001
    1.0004
    1.0011
    1.0012
    1.0007
      ⋮
observed2 = 4668×2
    1.0000    1.0008
    1.0000    1.0004
    1.0000    1.0008
    1.0000    1.0006
    1.0000    1.0001
    1.0000    1.0001
    1.0000    1.0004
    1.0000    1.0011
    1.0000    1.0012
    1.0000    1.0007
      ⋮
linreg = 2×1
    0.0123
    0.9710
GOFMatrix = 7×2
         0         0
    0.0322    0.9448
    0.0510    0.9143
    0.0235    0.9804
    0.1507    0.8672
    0.0466    0.8802
    0.0123    0.9710
pred = 1×4668
```

```
    0.9163    0.9148    0.9134    0.9120    0.9105    0.9091    0.9077    0.9063 ···
observed = 4668×1
    0.9935
    0.9936
    0.9934
    0.9952
    0.9950
    0.9945
    0.9948
    0.9950
    0.9959
    0.9959
      ⋮
observed2 = 4668×2
    1.0000    0.9935
    1.0000    0.9936
    1.0000    0.9934
    1.0000    0.9952
    1.0000    0.9950
    1.0000    0.9945
    1.0000    0.9948
    1.0000    0.9950
    1.0000    0.9959
    1.0000    0.9959
      ⋮
linreg = 2×1
    0.0909
    0.7820
GOFMatrix = 8×2
         0         0
    0.0322    0.9448
    0.0510    0.9143
    0.0235    0.9804
    0.1507    0.8672
    0.0466    0.8802
    0.0123    0.9710
    0.0909    0.7820
pred = 1×4668
    0.9172    0.9154    0.9137    0.9119    0.9102    0.9084    0.9067    0.9050 ···
observed = 4668×1
    1.0065
    1.0064
    1.0060
    1.0050
    1.0047
    1.0044
    1.0042
    1.0043
    1.0042
    1.0042
      ⋮
observed2 = 4668×2
    1.0000    1.0065
    1.0000    1.0064
    1.0000    1.0060
    1.0000    1.0050
    1.0000    1.0047
    1.0000    1.0044
    1.0000    1.0042
    1.0000    1.0043
    1.0000    1.0042
    1.0000    1.0042
```

```
     ⋮
linreg = 2×1
    0.1107
    0.7045
GOFMatrix = 9×2
         0         0
    0.0322    0.9448
    0.0510    0.9143
    0.0235    0.9804
    0.1507    0.8672
    0.0466    0.8802
    0.0123    0.9710
    0.0909    0.7820
    0.1107    0.7045
pred = 1×4668
    0.9588    0.9589    0.9589    0.9590    0.9590    0.9591    0.9591    0.9592 ⋯
observed = 4668×1
    0.9936
    0.9947
    0.9953
    0.9964
    0.9975
    0.9984
    0.9996
    1.0008
    1.0014
    1.0030
     ⋮
observed2 = 4668×2
    1.0000    0.9936
    1.0000    0.9947
    1.0000    0.9953
    1.0000    0.9964
    1.0000    0.9975
    1.0000    0.9984
    1.0000    0.9996
    1.0000    1.0008
    1.0000    1.0014
    1.0000    1.0030
     ⋮
linreg = 2×1
    0.0026
    0.9958
GOFMatrix = 10×2
         0         0
    0.0322    0.9448
    0.0510    0.9143
    0.0235    0.9804
    0.1507    0.8672
    0.0466    0.8802
    0.0123    0.9710
    0.0909    0.7820
    0.1107    0.7045
    0.0026    0.9958
pred = 1×4668
    0.9670    0.9670    0.9671    0.9671    0.9672    0.9673    0.9673    0.9674 ⋯
observed = 4668×1
    1.0058
    1.0072
    1.0091
    1.0096
    1.0103
```

```
    1.0110
    1.0110
    1.0112
    1.0117
    1.0114
      ⋮

observed2 = 4668×2
    1.0000    1.0058
    1.0000    1.0072
    1.0000    1.0091
    1.0000    1.0096
    1.0000    1.0103
    1.0000    1.0110
    1.0000    1.0110
    1.0000    1.0112
    1.0000    1.0117
    1.0000    1.0114
      ⋮

linreg = 2×1
    0.0008
    0.9987
GOFMatrix = 11×2
         0         0
    0.0322    0.9448
    0.0510    0.9143
    0.0235    0.9804
    0.1507    0.8672
    0.0466    0.8802
    0.0123    0.9710
    0.0909    0.7820
    0.1107    0.7045
    0.0026    0.9958
      ⋮

pred = 1×4668
    0.9599    0.9599    0.9600    0.9601    0.9602    0.9603    0.9604    0.9605 ···
observed = 4668×1
    1.0134
    1.0146
    1.0157
    1.0164
    1.0172
    1.0179
    1.0185
    1.0185
    1.0182
    1.0180
      ⋮

observed2 = 4668×2
    1.0000    1.0134
    1.0000    1.0146
    1.0000    1.0157
    1.0000    1.0164
    1.0000    1.0172
    1.0000    1.0179
    1.0000    1.0185
    1.0000    1.0185
    1.0000    1.0182
    1.0000    1.0180
      ⋮
```

```
linreg = 2×1
    0.0011
    0.9982
GOFMatrix = 12×2
         0         0
    0.0322    0.9448
    0.0510    0.9143
    0.0235    0.9804
    0.1507    0.8672
    0.0466    0.8802
    0.0123    0.9710
    0.0909    0.7820
    0.1107    0.7045
    0.0026    0.9958
      ⋮
pred = 1×4668
    0.9726    0.9726    0.9726    0.9727    0.9727    0.9728    0.9728    0.9729 ⋯
observed = 4668×1
    1.0059
    1.0063
    1.0065
    1.0068
    1.0068
    1.0072
    1.0075
    1.0078
    1.0086
    1.0091
      ⋮
observed2 = 4668×2
    1.0000    1.0059
    1.0000    1.0063
    1.0000    1.0065
    1.0000    1.0068
    1.0000    1.0068
    1.0000    1.0072
    1.0000    1.0075
    1.0000    1.0078
    1.0000    1.0086
    1.0000    1.0091
      ⋮
linreg = 2×1
    0.0009
    0.9986
GOFMatrix = 13×2
         0         0
    0.0322    0.9448
    0.0510    0.9143
    0.0235    0.9804
    0.1507    0.8672
    0.0466    0.8802
    0.0123    0.9710
    0.0909    0.7820
    0.1107    0.7045
    0.0026    0.9958
      ⋮
GOF_wtlps = 13×2
         0         0
    0.0322    0.9448
    0.0510    0.9143
    0.0235    0.9804
```

```
    0.1507    0.8672
    0.0466    0.8802
    0.0123    0.9710
    0.0909    0.7820
    0.1107    0.7045
    0.0026    0.9958
      ⋮
```

```
GOF_wtlps_2 = PolyGOF(wt(10:end,1:end), wt_Coeffs_minus10,12)
```

```
GOFMatrix = 1×2
     0     0
pred = 1×4659
    1.0786    1.0788    1.0791    1.0793    1.0795    1.0797    1.0800    1.0802 ⋯
observed = 4659×1
    0.9436
    0.9440
    0.9447
    0.9454
    0.9461
    0.9465
    0.9479
    0.9489
    0.9504
    0.9526
      ⋮
observed2 = 4659×2
    1.0000    0.9436
    1.0000    0.9440
    1.0000    0.9447
    1.0000    0.9454
    1.0000    0.9461
    1.0000    0.9465
    1.0000    0.9479
    1.0000    0.9489
    1.0000    0.9504
    1.0000    0.9526
      ⋮
linreg = 2×1
    0.0319
    0.9452
GOFMatrix = 2×2
         0         0
    0.0319    0.9452
pred = 1×4659
    1.1505    1.1505    1.1505    1.1505    1.1505    1.1505    1.1504    1.1504 ⋯
observed = 4659×1
    0.9936
    0.9944
    0.9948
    0.9950
    0.9950
    0.9953
    0.9949
    0.9944
    0.9942
    0.9939
      ⋮
observed2 = 4659×2
    1.0000    0.9936
```

```
    1.0000    0.9944
    1.0000    0.9948
    1.0000    0.9950
    1.0000    0.9950
    1.0000    0.9953
    1.0000    0.9949
    1.0000    0.9944
    1.0000    0.9942
    1.0000    0.9939
      :
      :
linreg = 2×1
    0.0507
    0.9147
GOFMatrix = 3×2
         0         0
    0.0319    0.9452
    0.0507    0.9147
pred = 1×4659
    0.9940    0.9941    0.9943    0.9944    0.9946    0.9947    0.9949    0.9950 ···
observed = 4659×1
    0.9988
    0.9989
    0.9994
    0.9998
    1.0001
    1.0005
    1.0010
    1.0014
    1.0018
    1.0017
      :
      :
observed2 = 4659×2
    1.0000    0.9988
    1.0000    0.9989
    1.0000    0.9994
    1.0000    0.9998
    1.0000    1.0001
    1.0000    1.0005
    1.0000    1.0010
    1.0000    1.0014
    1.0000    1.0018
    1.0000    1.0017
      :
      :
linreg = 2×1
    0.0236
    0.9803
GOFMatrix = 4×2
         0         0
    0.0319    0.9452
    0.0507    0.9147
    0.0236    0.9803
pred = 1×4659
    1.0203    1.0203    1.0204    1.0204    1.0205    1.0205    1.0206    1.0206 ···
observed = 4659×1
    0.9980
    0.9980
    0.9982
    0.9985
    0.9985
    0.9985
    0.9986
    0.9987
```

```
    0.9990
    0.9982
      :
observed2 = 4659×2
    1.0000    0.9980
    1.0000    0.9980
    1.0000    0.9982
    1.0000    0.9985
    1.0000    0.9985
    1.0000    0.9985
    1.0000    0.9986
    1.0000    0.9987
    1.0000    0.9990
    1.0000    0.9982
      :
linreg = 2×1
    0.1520
    0.8661
GOFMatrix = 5×2
         0         0
    0.0319    0.9452
    0.0507    0.9147
    0.0236    0.9803
    0.1520    0.8661
pred = 1×4659
    0.7781    0.7771    0.7761    0.7751    0.7741    0.7731    0.7720    0.7710 ···
observed = 4659×1
    1.0037
    1.0036
    1.0034
    1.0032
    1.0037
    1.0032
    1.0026
    1.0027
    1.0034
    1.0031
      :
observed2 = 4659×2
    1.0000    1.0037
    1.0000    1.0036
    1.0000    1.0034
    1.0000    1.0032
    1.0000    1.0037
    1.0000    1.0032
    1.0000    1.0026
    1.0000    1.0027
    1.0000    1.0034
    1.0000    1.0031
      :
linreg = 2×1
    0.0464
    0.8802
GOFMatrix = 6×2
         0         0
    0.0319    0.9452
    0.0507    0.9147
    0.0236    0.9803
    0.1520    0.8661
    0.0464    0.8802
pred = 1×4659
```

```
    0.9042    0.9034    0.9026    0.9018    0.9010    0.9003    0.8995    0.8987 ···
observed = 4659×1
    1.0007
    0.9996
    0.9987
    0.9988
    0.9991
    0.9999
    0.9997
    0.9998
    1.0002
    1.0012
      ⋮
observed2 = 4659×2
    1.0000    1.0007
    1.0000    0.9996
    1.0000    0.9987
    1.0000    0.9988
    1.0000    0.9991
    1.0000    0.9999
    1.0000    0.9997
    1.0000    0.9998
    1.0000    1.0002
    1.0000    1.0012
      ⋮
linreg = 2×1
    0.0123
    0.9708
GOFMatrix = 7×2
         0         0
    0.0319    0.9452
    0.0507    0.9147
    0.0236    0.9803
    0.1520    0.8661
    0.0464    0.8802
    0.0123    0.9708
pred = 1×4659
    0.8975    0.8961    0.8947    0.8933    0.8919    0.8906    0.8892    0.8878 ···
observed = 4659×1
    0.9959
    0.9959
    0.9959
    0.9956
    0.9965
    0.9963
    0.9960
    0.9963
    0.9964
    0.9967
      ⋮
observed2 = 4659×2
    1.0000    0.9959
    1.0000    0.9959
    1.0000    0.9959
    1.0000    0.9956
    1.0000    0.9965
    1.0000    0.9963
    1.0000    0.9960
    1.0000    0.9963
    1.0000    0.9964
    1.0000    0.9967
```

```
      ⋮
linreg = 2×1
    0.0954
    0.7706
GOFMatrix = 8×2
         0         0
    0.0319    0.9452
    0.0507    0.9147
    0.0236    0.9803
    0.1520    0.8661
    0.0464    0.8802
    0.0123    0.9708
    0.0954    0.7706
pred = 1×4659
    0.8948    0.8931    0.8914    0.8897    0.8880    0.8863    0.8847    0.8830 ⋯
observed = 4659×1
    1.0042
    1.0041
    1.0032
    1.0030
    1.0035
    1.0032
    1.0026
    1.0022
    1.0010
    0.9999
      ⋮
observed2 = 4659×2
    1.0000    1.0042
    1.0000    1.0041
    1.0000    1.0032
    1.0000    1.0030
    1.0000    1.0035
    1.0000    1.0032
    1.0000    1.0026
    1.0000    1.0022
    1.0000    1.0010
    1.0000    0.9999
      ⋮
linreg = 2×1
    0.1166
    0.6877
GOFMatrix = 9×2
         0         0
    0.0319    0.9452
    0.0507    0.9147
    0.0236    0.9803
    0.1520    0.8661
    0.0464    0.8802
    0.0123    0.9708
    0.0954    0.7706
    0.1166    0.6877
pred = 1×4659
    0.9566    0.9566    0.9567    0.9568    0.9568    0.9569    0.9569    0.9570 ⋯
observed = 4659×1
    1.0030
    1.0036
    1.0047
    1.0053
    1.0061
    1.0065
    1.0067
```

```
    1.0073
    1.0071
    1.0070
      :
      :
observed2 = 4659×2
    1.0000    1.0030
    1.0000    1.0036
    1.0000    1.0047
    1.0000    1.0053
    1.0000    1.0061
    1.0000    1.0065
    1.0000    1.0067
    1.0000    1.0073
    1.0000    1.0071
    1.0000    1.0070
      :
      :
linreg = 2×1
    0.0026
    0.9958
GOFMatrix = 10×2
         0         0
    0.0319    0.9452
    0.0507    0.9147
    0.0236    0.9803
    0.1520    0.8661
    0.0464    0.8802
    0.0123    0.9708
    0.0954    0.7706
    0.1166    0.6877
    0.0026    0.9958
pred = 1×4659
    0.9645    0.9645    0.9646    0.9647    0.9647    0.9648    0.9649    0.9649 ···
observed = 4659×1
    1.0114
    1.0113
    1.0111
    1.0107
    1.0108
    1.0104
    1.0098
    1.0098
    1.0097
    1.0094
      :
      :
observed2 = 4659×2
    1.0000    1.0114
    1.0000    1.0113
    1.0000    1.0111
    1.0000    1.0107
    1.0000    1.0108
    1.0000    1.0104
    1.0000    1.0098
    1.0000    1.0098
    1.0000    1.0097
    1.0000    1.0094
      :
      :
linreg = 2×1
    0.0007
    0.9988
GOFMatrix = 11×2
         0         0
```

```
    0.0319    0.9452
    0.0507    0.9147
    0.0236    0.9803
    0.1520    0.8661
    0.0464    0.8802
    0.0123    0.9708
    0.0954    0.7706
    0.1166    0.6877
    0.0026    0.9958
      :
      :
pred = 1×4659
    0.9566    0.9567    0.9568    0.9569    0.9570    0.9571    0.9572    0.9573 ···
observed = 4659×1
    1.0180
    1.0178
    1.0176
    1.0171
    1.0168
    1.0161
    1.0157
    1.0155
    1.0152
    1.0149
      :
      :
observed2 = 4659×2
    1.0000    1.0180
    1.0000    1.0178
    1.0000    1.0176
    1.0000    1.0171
    1.0000    1.0168
    1.0000    1.0161
    1.0000    1.0157
    1.0000    1.0155
    1.0000    1.0152
    1.0000    1.0149
      :
      :
linreg = 2×1
    0.0010
    0.9984
GOFMatrix = 12×2
         0         0
    0.0319    0.9452
    0.0507    0.9147
    0.0236    0.9803
    0.1520    0.8661
    0.0464    0.8802
    0.0123    0.9708
    0.0954    0.7706
    0.1166    0.6877
    0.0026    0.9958
      :
      :
pred = 1×4659
    0.9705    0.9706    0.9706    0.9707    0.9707    0.9708    0.9708    0.9709 ···
observed = 4659×1
    1.0091
    1.0092
    1.0087
    1.0089
    1.0087
    1.0085
    1.0085
```

```
    1.0081
    1.0076
    1.0071
      ⋮

observed2 = 4659×2
    1.0000    1.0091
    1.0000    1.0092
    1.0000    1.0087
    1.0000    1.0089
    1.0000    1.0087
    1.0000    1.0085
    1.0000    1.0085
    1.0000    1.0081
    1.0000    1.0076
    1.0000    1.0071
      ⋮

linreg = 2×1
    0.0009
    0.9987
GOFMatrix = 13×2
         0         0
    0.0319    0.9452
    0.0507    0.9147
    0.0236    0.9803
    0.1520    0.8661
    0.0464    0.8802
    0.0123    0.9708
    0.0954    0.7706
    0.1166    0.6877
    0.0026    0.9958
      ⋮

GOF_wtlps_2 = 13×2
         0         0
    0.0319    0.9452
    0.0507    0.9147
    0.0236    0.9803
    0.1520    0.8661
    0.0464    0.8802
    0.0123    0.9708
    0.0954    0.7706
    0.1166    0.6877
    0.0026    0.9958
      ⋮
```

```
GOF_wtlps_3 = PolyGOF(wt(20:end,1:end), wt_Coeffs_minus20,12)
```

```
GOFMatrix = 1×2
     0     0
pred = 1×4649
    1.0913    1.0914    1.0916    1.0918    1.0919    1.0921    1.0923    1.0924 ⋯
observed = 4649×1
    0.9548
    0.9577
    0.9598
    0.9624
    0.9647
    0.9672
    0.9699
    0.9744
    0.9818
```

```
    0.9887
      ⋮

observed2 = 4649×2
    1.0000    0.9548
    1.0000    0.9577
    1.0000    0.9598
    1.0000    0.9624
    1.0000    0.9647
    1.0000    0.9672
    1.0000    0.9699
    1.0000    0.9744
    1.0000    0.9818
    1.0000    0.9887
      ⋮

linreg = 2×1
    0.0315
    0.9458
GOFMatrix = 2×2
         0         0
    0.0315    0.9458
pred = 1×4649
    1.1626    1.1626    1.1625    1.1624    1.1624    1.1623    1.1622    1.1621 ⋯
observed = 4649×1
    0.9937
    0.9935
    0.9928
    0.9923
    0.9929
    0.9933
    0.9937
    0.9946
    0.9952
    0.9971
      ⋮

observed2 = 4649×2
    1.0000    0.9937
    1.0000    0.9935
    1.0000    0.9928
    1.0000    0.9923
    1.0000    0.9929
    1.0000    0.9933
    1.0000    0.9937
    1.0000    0.9946
    1.0000    0.9952
    1.0000    0.9971
      ⋮

linreg = 2×1
    0.0503
    0.9152
GOFMatrix = 3×2
         0         0
    0.0315    0.9458
    0.0503    0.9152
pred = 1×4649
    0.9950    0.9951    0.9953    0.9954    0.9956    0.9957    0.9959    0.9960 ⋯
observed = 4649×1
    1.0019
    1.0015
    1.0015
    1.0012
    1.0013
```

```
    1.0014
    1.0014
    1.0014
    1.0011
    1.0008
      :
      :
observed2 = 4649×2
    1.0000    1.0019
    1.0000    1.0015
    1.0000    1.0015
    1.0000    1.0012
    1.0000    1.0013
    1.0000    1.0014
    1.0000    1.0014
    1.0000    1.0014
    1.0000    1.0011
    1.0000    1.0008
      :
      :
linreg = 2×1
    0.0238
    0.9802
GOFMatrix = 4×2
         0         0
    0.0315    0.9458
    0.0503    0.9152
    0.0238    0.9802
pred = 1×4649
    1.0225    1.0226    1.0226    1.0227    1.0227    1.0228    1.0228    1.0228 ···
observed = 4649×1
    0.9983
    0.9978
    0.9982
    0.9989
    0.9990
    0.9990
    0.9995
    1.0002
    1.0002
    1.0000
      :
      :
observed2 = 4649×2
    1.0000    0.9983
    1.0000    0.9978
    1.0000    0.9982
    1.0000    0.9989
    1.0000    0.9990
    1.0000    0.9990
    1.0000    0.9995
    1.0000    1.0002
    1.0000    1.0002
    1.0000    1.0000
      :
      :
linreg = 2×1
    0.1535
    0.8648
GOFMatrix = 5×2
         0         0
    0.0315    0.9458
    0.0503    0.9152
    0.0238    0.9802
    0.1535    0.8648
```

```
pred = 1×4649
    0.7500    0.7490    0.7481    0.7472    0.7463    0.7454    0.7445    0.7435 ···
observed = 4649×1
    1.0025
    1.0022
    1.0025
    1.0024
    1.0027
    1.0024
    1.0016
    1.0011
    1.0012
    1.0011
      ⋮
observed2 = 4649×2
    1.0000    1.0025
    1.0000    1.0022
    1.0000    1.0025
    1.0000    1.0024
    1.0000    1.0027
    1.0000    1.0024
    1.0000    1.0016
    1.0000    1.0011
    1.0000    1.0012
    1.0000    1.0011
      ⋮
linreg = 2×1
    0.0448
    0.8840
GOFMatrix = 6×2
         0         0
    0.0315    0.9458
    0.0503    0.9152
    0.0238    0.9802
    0.1535    0.8648
    0.0448    0.8840
pred = 1×4649
    0.8885    0.8878    0.8870    0.8862    0.8855    0.8847    0.8840    0.8832 ···
observed = 4649×1
    1.0017
    1.0018
    1.0018
    1.0021
    1.0025
    1.0023
    1.0018
    1.0020
    1.0018
    1.0011
      ⋮
observed2 = 4649×2
    1.0000    1.0017
    1.0000    1.0018
    1.0000    1.0018
    1.0000    1.0021
    1.0000    1.0025
    1.0000    1.0023
    1.0000    1.0018
    1.0000    1.0020
    1.0000    1.0018
    1.0000    1.0011
```

```
      ⋮
linreg = 2×1
    0.0123
    0.9709
GOFMatrix = 7×2
         0         0
    0.0315    0.9458
    0.0503    0.9152
    0.0238    0.9802
    0.1535    0.8648
    0.0448    0.8840
    0.0123    0.9709
pred = 1×4649
    0.8755    0.8741    0.8728    0.8715    0.8701    0.8688    0.8675    0.8662 ⋯
observed = 4649×1
    0.9970
    0.9973
    0.9976
    0.9978
    0.9976
    0.9976
    0.9980
    0.9981
    0.9977
    0.9983
      ⋮
observed2 = 4649×2
    1.0000    0.9970
    1.0000    0.9973
    1.0000    0.9976
    1.0000    0.9978
    1.0000    0.9976
    1.0000    0.9976
    1.0000    0.9980
    1.0000    0.9981
    1.0000    0.9977
    1.0000    0.9983
      ⋮
linreg = 2×1
    0.1007
    0.7569
GOFMatrix = 8×2
         0         0
    0.0315    0.9458
    0.0503    0.9152
    0.0238    0.9802
    0.1535    0.8648
    0.0448    0.8840
    0.0123    0.9709
    0.1007    0.7569
pred = 1×4649
    0.8689    0.8673    0.8656    0.8640    0.8624    0.8608    0.8592    0.8576 ⋯
observed = 4649×1
    0.9995
    0.9992
    0.9994
    0.9997
    0.9998
    0.9999
    0.9999
    0.9998
    0.9998
```

```
    1.0006
      ⋮
observed2 = 4649×2
    1.0000    0.9995
    1.0000    0.9992
    1.0000    0.9994
    1.0000    0.9997
    1.0000    0.9998
    1.0000    0.9999
    1.0000    0.9999
    1.0000    0.9998
    1.0000    0.9998
    1.0000    1.0006
      ⋮
linreg = 2×1
    0.1237
    0.6674
GOFMatrix = 9×2
         0         0
    0.0315    0.9458
    0.0503    0.9152
    0.0238    0.9802
    0.1535    0.8648
    0.0448    0.8840
    0.0123    0.9709
    0.1007    0.7569
    0.1237    0.6674
pred = 1×4649
    0.9533    0.9534    0.9534    0.9535    0.9536    0.9536    0.9537    0.9538 ⋯
observed = 4649×1
    1.0069
    1.0070
    1.0067
    1.0066
    1.0064
    1.0059
    1.0053
    1.0046
    1.0038
    1.0030
      ⋮
observed2 = 4649×2
    1.0000    1.0069
    1.0000    1.0070
    1.0000    1.0067
    1.0000    1.0066
    1.0000    1.0064
    1.0000    1.0059
    1.0000    1.0053
    1.0000    1.0046
    1.0000    1.0038
    1.0000    1.0030
      ⋮
linreg = 2×1
    0.0025
    0.9960
GOFMatrix = 10×2
         0         0
    0.0315    0.9458
    0.0503    0.9152
    0.0238    0.9802
```

```
    0.1535    0.8648
    0.0448    0.8840
    0.0123    0.9709
    0.1007    0.7569
    0.1237    0.6674
    0.0025    0.9960
pred = 1×4649
    0.9615    0.9616    0.9616    0.9617    0.9618    0.9618    0.9619    0.9620 ···
observed = 4649×1
    1.0090
    1.0084
    1.0078
    1.0072
    1.0068
    1.0063
    1.0055
    1.0050
    1.0038
    1.0029
      ⋮
observed2 = 4649×2
    1.0000    1.0090
    1.0000    1.0084
    1.0000    1.0078
    1.0000    1.0072
    1.0000    1.0068
    1.0000    1.0063
    1.0000    1.0055
    1.0000    1.0050
    1.0000    1.0038
    1.0000    1.0029
      ⋮
linreg = 2×1
    0.0006
    0.9990
GOFMatrix = 11×2
         0         0
    0.0315    0.9458
    0.0503    0.9152
    0.0238    0.9802
    0.1535    0.8648
    0.0448    0.8840
    0.0123    0.9709
    0.1007    0.7569
    0.1237    0.6674
    0.0025    0.9960
      ⋮
pred = 1×4649
    0.9529    0.9530    0.9531    0.9532    0.9533    0.9534    0.9535    0.9536 ···
observed = 4649×1
    1.0141
    1.0132
    1.0120
    1.0113
    1.0103
    1.0093
    1.0082
    1.0070
    1.0056
    1.0043
      ⋮
```

```
observed2 = 4649×2
    1.0000    1.0141
    1.0000    1.0132
    1.0000    1.0120
    1.0000    1.0113
    1.0000    1.0103
    1.0000    1.0093
    1.0000    1.0082
    1.0000    1.0070
    1.0000    1.0056
    1.0000    1.0043
      ⋮
linreg = 2×1
    0.0009
    0.9986
GOFMatrix = 12×2
         0         0
    0.0315    0.9458
    0.0503    0.9152
    0.0238    0.9802
    0.1535    0.8648
    0.0448    0.8840
    0.0123    0.9709
    0.1007    0.7569
    0.1237    0.6674
    0.0025    0.9960
      ⋮
pred = 1×4649
    0.9680    0.9681    0.9681    0.9682    0.9683    0.9683    0.9684    0.9684 ⋯
observed = 4649×1
    1.0066
    1.0061
    1.0058
    1.0053
    1.0050
    1.0046
    1.0036
    1.0031
    1.0025
    1.0022
      ⋮
observed2 = 4649×2
    1.0000    1.0066
    1.0000    1.0061
    1.0000    1.0058
    1.0000    1.0053
    1.0000    1.0050
    1.0000    1.0046
    1.0000    1.0036
    1.0000    1.0031
    1.0000    1.0025
    1.0000    1.0022
      ⋮
linreg = 2×1
    0.0008
    0.9988
GOFMatrix = 13×2
         0         0
    0.0315    0.9458
    0.0503    0.9152
    0.0238    0.9802
```

```
    0.1535    0.8648
    0.0448    0.8840
    0.0123    0.9709
    0.1007    0.7569
    0.1237    0.6674
    0.0025    0.9960
      :
      :
GOF_wtlps_3 = 13×2
         0         0
    0.0315    0.9458
    0.0503    0.9152
    0.0238    0.9802
    0.1535    0.8648
    0.0448    0.8840
    0.0123    0.9709
    0.1007    0.7569
    0.1237    0.6674
    0.0025    0.9960
      :
      :
```

```
GOF_fynlps = PolyGOF(fyn, FynCoeffs,7)
```

```
GOFMatrix = 1×2
     0     0
pred = 1×4505
    0.9948    0.9956    0.9964    0.9973    0.9981    0.9989    0.9998    1.0006 ...
observed = 4505×1
    0.9853
    0.9863
    0.9876
    0.9887
    0.9892
    0.9903
    0.9911
    0.9921
    0.9930
    0.9941
      :
      :
observed2 = 4505×2
    1.0000    0.9853
    1.0000    0.9863
    1.0000    0.9876
    1.0000    0.9887
    1.0000    0.9892
    1.0000    0.9903
    1.0000    0.9911
    1.0000    0.9921
    1.0000    0.9930
    1.0000    0.9941
      :
      :
linreg = 2×1
    0.0079
    0.9904
GOFMatrix = 2×2
         0         0
    0.0079    0.9904
pred = 1×4505
    0.6590    0.6638    0.6686    0.6734    0.6782    0.6830    0.6878    0.6925 ...
observed = 4505×1
```

```
    0.9977
    0.9979
    0.9977
    0.9977
    0.9979
    0.9981
    0.9981
    0.9981
    0.9981
    0.9985
      :
      :
observed2 = 4505×2
    1.0000    0.9977
    1.0000    0.9979
    1.0000    0.9977
    1.0000    0.9977
    1.0000    0.9979
    1.0000    0.9981
    1.0000    0.9981
    1.0000    0.9981
    1.0000    0.9981
    1.0000    0.9985
      :
      :
linreg = 2×1
    0.0439
    0.9689
GOFMatrix = 3×2
         0         0
    0.0079    0.9904
    0.0439    0.9689
pred = 1×4505
    0.6742    0.6786    0.6829    0.6873    0.6916    0.6959    0.7002    0.7045 ···
observed = 4505×1
    0.9957
    0.9957
    0.9957
    0.9961
    0.9959
    0.9961
    0.9963
    0.9963
    0.9965
    0.9967
      :
      :
observed2 = 4505×2
    1.0000    0.9957
    1.0000    0.9957
    1.0000    0.9957
    1.0000    0.9961
    1.0000    0.9959
    1.0000    0.9961
    1.0000    0.9963
    1.0000    0.9963
    1.0000    0.9965
    1.0000    0.9967
      :
      :
linreg = 2×1
    0.0511
    0.9636
GOFMatrix = 4×2
         0         0
```

```
    0.0079    0.9904
    0.0439    0.9689
    0.0511    0.9636
pred = 1×4505
    0.7447    0.7491    0.7536    0.7580    0.7624    0.7668    0.7712    0.7756 ···
observed = 4505×1
    1.0183
    1.0179
    1.0175
    1.0168
    1.0164
    1.0162
    1.0160
    1.0159
    1.0151
    1.0149
      ⋮
observed2 = 4505×2
    1.0000    1.0183
    1.0000    1.0179
    1.0000    1.0175
    1.0000    1.0168
    1.0000    1.0164
    1.0000    1.0162
    1.0000    1.0160
    1.0000    1.0159
    1.0000    1.0151
    1.0000    1.0149
      ⋮
linreg = 2×1
    0.0328
    0.9774
GOFMatrix = 5×2
         0         0
    0.0079    0.9904
    0.0439    0.9689
    0.0511    0.9636
    0.0328    0.9774
pred = 1×4505
    0.7522    0.7564    0.7607    0.7650    0.7693    0.7735    0.7778    0.7820 ···
observed = 4505×1
    0.9961
    0.9962
    0.9964
    0.9964
    0.9964
    0.9966
    0.9966
    0.9966
    0.9962
    0.9969
      ⋮
observed2 = 4505×2
    1.0000    0.9961
    1.0000    0.9962
    1.0000    0.9964
    1.0000    0.9964
    1.0000    0.9964
    1.0000    0.9966
    1.0000    0.9966
    1.0000    0.9966
    1.0000    0.9962
```

```
    1.0000    0.9969
      :
linreg = 2×1
    0.0477
    0.9660
GOFMatrix = 6×2
         0         0
    0.0079    0.9904
    0.0439    0.9689
    0.0511    0.9636
    0.0328    0.9774
    0.0477    0.9660
pred = 1×4505
    0.9853    0.9853    0.9854    0.9854    0.9855    0.9855    0.9856    0.9856 ···
observed = 4505×1
    1.0189
    1.0188
    1.0186
    1.0181
    1.0183
    1.0182
    1.0181
    1.0176
    1.0179
    1.0175
      :
observed2 = 4505×2
    1.0000    1.0189
    1.0000    1.0188
    1.0000    1.0186
    1.0000    1.0181
    1.0000    1.0183
    1.0000    1.0182
    1.0000    1.0181
    1.0000    1.0176
    1.0000    1.0179
    1.0000    1.0175
      :
linreg = 2×1
    0.0461
    0.9567
GOFMatrix = 7×2
         0         0
    0.0079    0.9904
    0.0439    0.9689
    0.0511    0.9636
    0.0328    0.9774
    0.0477    0.9660
    0.0461    0.9567
pred = 1×4505
    1.0111    1.0112    1.0113    1.0113    1.0114    1.0115    1.0115    1.0116 ···
observed = 4505×1
    1.0460
    1.0464
    1.0466
    1.0467
    1.0467
    1.0467
    1.0467
    1.0461
    1.0461
    1.0461
```

```
      ⋮
observed2 = 4505×2
    1.0000    1.0460
    1.0000    1.0464
    1.0000    1.0466
    1.0000    1.0467
    1.0000    1.0467
    1.0000    1.0467
    1.0000    1.0467
    1.0000    1.0461
    1.0000    1.0461
    1.0000    1.0461
      ⋮
linreg = 2×1
    0.0733
    0.9343
GOFMatrix = 8×2
         0         0
    0.0079    0.9904
    0.0439    0.9689
    0.0511    0.9636
    0.0328    0.9774
    0.0477    0.9660
    0.0461    0.9567
    0.0733    0.9343
GOF_fynlps = 8×2
         0         0
    0.0079    0.9904
    0.0439    0.9689
    0.0511    0.9636
    0.0328    0.9774
    0.0477    0.9660
    0.0461    0.9567
    0.0733    0.9343
```

```
GOF_fynlps_minus10 = PolyGOF(fyn(10:end,1:end), fyn_Coeffs_minus10,7)
```

```
GOFMatrix = 1×2
     0     0
pred = 1×4496
    1.0028    1.0037    1.0045    1.0053    1.0061    1.0069    1.0077    1.0084 ⋯
observed = 4496×1
    0.9941
    0.9950
    0.9956
    0.9963
    0.9965
    0.9969
    0.9973
    0.9977
    0.9982
    0.9990
      ⋮
observed2 = 4496×2
    1.0000    0.9941
    1.0000    0.9950
    1.0000    0.9956
    1.0000    0.9963
    1.0000    0.9965
    1.0000    0.9969
    1.0000    0.9973
```

```
    1.0000    0.9977
    1.0000    0.9982
    1.0000    0.9990
      :
      :
linreg = 2×1
    0.0079
    0.9904
GOFMatrix = 2×2
         0         0
    0.0079    0.9904
pred = 1×4496
    0.6786    0.6834    0.6882    0.6930    0.6977    0.7025    0.7073    0.7120 ···
observed = 4496×1
    0.9985
    0.9985
    0.9989
    0.9991
    0.9992
    0.9996
    1.0002
    1.0002
    1.0002
    1.0004
      :
      :
observed2 = 4496×2
    1.0000    0.9985
    1.0000    0.9985
    1.0000    0.9989
    1.0000    0.9991
    1.0000    0.9992
    1.0000    0.9996
    1.0000    1.0002
    1.0000    1.0002
    1.0000    1.0002
    1.0000    1.0004
      :
      :
linreg = 2×1
    0.0417
    0.9705
GOFMatrix = 3×2
         0         0
    0.0079    0.9904
    0.0417    0.9705
pred = 1×4496
    0.6907    0.6951    0.6994    0.7038    0.7081    0.7124    0.7167    0.7210 ···
observed = 4496×1
    0.9967
    0.9967
    0.9967
    0.9969
    0.9971
    0.9971
    0.9973
    0.9973
    0.9973
    0.9976
      :
      :
observed2 = 4496×2
    1.0000    0.9967
    1.0000    0.9967
    1.0000    0.9967
```

```
    1.0000    0.9969
    1.0000    0.9971
    1.0000    0.9971
    1.0000    0.9973
    1.0000    0.9973
    1.0000    0.9973
    1.0000    0.9976
      :
      :
linreg = 2×1
    0.0489
    0.9651
GOFMatrix = 4×2
         0         0
    0.0079    0.9904
    0.0417    0.9705
    0.0489    0.9651
pred = 1×4496
    0.7658    0.7702    0.7746    0.7791    0.7835    0.7879    0.7922    0.7966 ⋯
observed = 4496×1
    1.0149
    1.0146
    1.0144
    1.0142
    1.0133
    1.0129
    1.0129
    1.0124
    1.0124
    1.0120
      :
      :
observed2 = 4496×2
    1.0000    1.0149
    1.0000    1.0146
    1.0000    1.0144
    1.0000    1.0142
    1.0000    1.0133
    1.0000    1.0129
    1.0000    1.0129
    1.0000    1.0124
    1.0000    1.0124
    1.0000    1.0120
      :
      :
linreg = 2×1
    0.0315
    0.9784
GOFMatrix = 5×2
         0         0
    0.0079    0.9904
    0.0417    0.9705
    0.0489    0.9651
    0.0315    0.9784
pred = 1×4496
    0.7738    0.7781    0.7823    0.7866    0.7908    0.7950    0.7993    0.8035 ⋯
observed = 4496×1
    0.9969
    0.9966
    0.9969
    0.9966
    0.9966
    0.9964
    0.9968
    0.9973
```

```
    0.9974
    0.9976
      :
      :
observed2 = 4496×2
    1.0000    0.9969
    1.0000    0.9966
    1.0000    0.9969
    1.0000    0.9966
    1.0000    0.9966
    1.0000    0.9964
    1.0000    0.9968
    1.0000    0.9973
    1.0000    0.9974
    1.0000    0.9976
      :
      :
linreg = 2×1
    0.0465
    0.9668
GOFMatrix = 6×2
         0         0
    0.0079    0.9904
    0.0417    0.9705
    0.0489    0.9651
    0.0315    0.9784
    0.0465    0.9668
pred = 1×4496
    0.9833    0.9834    0.9834    0.9835    0.9835    0.9836    0.9837    0.9837 ⋯
observed = 4496×1
    1.0175
    1.0173
    1.0172
    1.0172
    1.0171
    1.0171
    1.0171
    1.0168
    1.0164
    1.0162
      :
      :
observed2 = 4496×2
    1.0000    1.0175
    1.0000    1.0173
    1.0000    1.0172
    1.0000    1.0172
    1.0000    1.0171
    1.0000    1.0171
    1.0000    1.0171
    1.0000    1.0168
    1.0000    1.0164
    1.0000    1.0162
      :
      :
linreg = 2×1
    0.0447
    0.9580
GOFMatrix = 7×2
         0         0
    0.0079    0.9904
    0.0417    0.9705
    0.0489    0.9651
    0.0315    0.9784
    0.0465    0.9668
```

```
    0.0447    0.9580
pred = 1×4496
    1.0091    1.0092    1.0093    1.0094    1.0095    1.0095    1.0096    1.0097 ⋯
observed = 4496×1
    1.0461
    1.0463
    1.0461
    1.0461
    1.0461
    1.0457
    1.0456
    1.0455
    1.0455
    1.0452
      ⋮
observed2 = 4496×2
    1.0000    1.0461
    1.0000    1.0463
    1.0000    1.0461
    1.0000    1.0461
    1.0000    1.0461
    1.0000    1.0457
    1.0000    1.0456
    1.0000    1.0455
    1.0000    1.0455
    1.0000    1.0452
      ⋮
linreg = 2×1
    0.0722
    0.9352
GOFMatrix = 8×2
         0         0
    0.0079    0.9904
    0.0417    0.9705
    0.0489    0.9651
    0.0315    0.9784
    0.0465    0.9668
    0.0447    0.9580
    0.0722    0.9352
GOF_fynlps_minus10 = 8×2
         0         0
    0.0079    0.9904
    0.0417    0.9705
    0.0489    0.9651
    0.0315    0.9784
    0.0465    0.9668
    0.0447    0.9580
    0.0722    0.9352
```

```
GOF_fynlps_minus20 = PolyGOF(fyn(20:end,1:end), fyn_Coeffs_minus20,7)
```

```
GOFMatrix = 1×2
     0     0
pred = 1×4486
    1.0116    1.0124    1.0131    1.0139    1.0147    1.0155    1.0162    1.0170 ⋯
observed = 4486×1
    0.9996
    1.0003
    1.0012
    1.0018
    1.0026
    1.0031
```

```
    1.0033
    1.0039
    1.0046
    1.0049
      :
observed2 = 4486×2
    1.0000    0.9996
    1.0000    1.0003
    1.0000    1.0012
    1.0000    1.0018
    1.0000    1.0026
    1.0000    1.0031
    1.0000    1.0033
    1.0000    1.0039
    1.0000    1.0046
    1.0000    1.0049
      :
linreg = 2×1
    0.0080
    0.9904
GOFMatrix = 2×2
         0         0
    0.0080    0.9904
pred = 1×4486
    0.7017    0.7065    0.7113    0.7161    0.7208    0.7256    0.7303    0.7351 ...
observed = 4486×1
    1.0000
    1.0000
    1.0002
    1.0006
    1.0004
    1.0008
    1.0008
    1.0006
    1.0008
    1.0009
      :
observed2 = 4486×2
    1.0000    1.0000
    1.0000    1.0000
    1.0000    1.0002
    1.0000    1.0006
    1.0000    1.0004
    1.0000    1.0008
    1.0000    1.0008
    1.0000    1.0006
    1.0000    1.0008
    1.0000    1.0009
      :
linreg = 2×1
    0.0395
    0.9721
GOFMatrix = 3×2
         0         0
    0.0080    0.9904
    0.0395    0.9721
pred = 1×4486
    0.7104    0.7147    0.7191    0.7234    0.7277    0.7320    0.7363    0.7406 ...
observed = 4486×1
    0.9976
    0.9973
```

```
    0.9976
    0.9973
    0.9978
    0.9978
    0.9980
    0.9978
    0.9982
    0.9980
      :
      :
observed2 = 4486×2
    1.0000    0.9976
    1.0000    0.9973
    1.0000    0.9976
    1.0000    0.9973
    1.0000    0.9978
    1.0000    0.9978
    1.0000    0.9980
    1.0000    0.9978
    1.0000    0.9982
    1.0000    0.9980
      :
      :
linreg = 2×1
    0.0468
    0.9667
GOFMatrix = 4×2
         0         0
    0.0080    0.9904
    0.0395    0.9721
    0.0468    0.9667
pred = 1×4486
    0.7911    0.7955    0.7999    0.8043    0.8087    0.8130    0.8174    0.8218 ···
observed = 4486×1
    1.0118
    1.0114
    1.0111
    1.0109
    1.0105
    1.0105
    1.0103
    1.0100
    1.0098
    1.0092
      :
      :
observed2 = 4486×2
    1.0000    1.0118
    1.0000    1.0114
    1.0000    1.0111
    1.0000    1.0109
    1.0000    1.0105
    1.0000    1.0105
    1.0000    1.0103
    1.0000    1.0100
    1.0000    1.0098
    1.0000    1.0092
      :
      :
linreg = 2×1
    0.0303
    0.9792
GOFMatrix = 5×2
         0         0
    0.0080    0.9904
```

```
    0.0395    0.9721
    0.0468    0.9667
    0.0303    0.9792
pred = 1×4486
    0.7994    0.8036    0.8078    0.8121    0.8163    0.8205    0.8247    0.8288 ···
observed = 4486×1
    0.9976
    0.9979
    0.9981
    0.9983
    0.9983
    0.9985
    0.9983
    0.9986
    0.9988
    0.9988
      ⋮
observed2 = 4486×2
    1.0000    0.9976
    1.0000    0.9979
    1.0000    0.9981
    1.0000    0.9983
    1.0000    0.9983
    1.0000    0.9985
    1.0000    0.9983
    1.0000    0.9986
    1.0000    0.9988
    1.0000    0.9988
      ⋮
linreg = 2×1
    0.0455
    0.9676
GOFMatrix = 6×2
         0         0
    0.0080    0.9904
    0.0395    0.9721
    0.0468    0.9667
    0.0303    0.9792
    0.0455    0.9676
pred = 1×4486
    0.9812    0.9812    0.9813    0.9814    0.9814    0.9815    0.9816    0.9817 ···
observed = 4486×1
    1.0164
    1.0161
    1.0158
    1.0148
    1.0141
    1.0135
    1.0128
    1.0114
    1.0088
    1.0077
      ⋮
observed2 = 4486×2
    1.0000    1.0164
    1.0000    1.0161
    1.0000    1.0158
    1.0000    1.0148
    1.0000    1.0141
    1.0000    1.0135
    1.0000    1.0128
    1.0000    1.0114
```

```
    1.0000    1.0088
    1.0000    1.0077
      :
      :
linreg = 2×1
    0.0431
    0.9595
GOFMatrix = 7×2
         0         0
    0.0080    0.9904
    0.0395    0.9721
    0.0468    0.9667
    0.0303    0.9792
    0.0455    0.9676
    0.0431    0.9595
pred = 1×4486
    1.0069    1.0070    1.0071    1.0072    1.0073    1.0074    1.0075    1.0075 ···
observed = 4486×1
    1.0456
    1.0459
    1.0460
    1.0460
    1.0461
    1.0463
    1.0460
    1.0460
    1.0459
    1.0455
      :
      :
observed2 = 4486×2
    1.0000    1.0456
    1.0000    1.0459
    1.0000    1.0460
    1.0000    1.0460
    1.0000    1.0461
    1.0000    1.0463
    1.0000    1.0460
    1.0000    1.0460
    1.0000    1.0459
    1.0000    1.0455
      :
      :
linreg = 2×1
    0.0709
    0.9364
GOFMatrix = 8×2
         0         0
    0.0080    0.9904
    0.0395    0.9721
    0.0468    0.9667
    0.0303    0.9792
    0.0455    0.9676
    0.0431    0.9595
    0.0709    0.9364
GOF_fynlps_minus20 = 8×2
         0         0
    0.0080    0.9904
    0.0395    0.9721
    0.0468    0.9667
    0.0303    0.9792
    0.0455    0.9676
    0.0431    0.9595
    0.0709    0.9364
```

```
GOF_wtlps50 = PolyGOF(wt_lps50, WTCoeffslps50,4)
```

```
GOFMatrix = 1×2
     0     0
pred = 1×6210
    1.0145    1.0154    1.0163    1.0171    1.0180    1.0189    1.0198    1.0207 ⋯
observed = 6210×1
    0.9908
    0.9908
    0.9902
    0.9902
    0.9909
    0.9913
    0.9916
    0.9919
    0.9924
    0.9928
      ⋮
observed2 = 6210×2
    1.0000    0.9908
    1.0000    0.9908
    1.0000    0.9902
    1.0000    0.9902
    1.0000    0.9909
    1.0000    0.9913
    1.0000    0.9916
    1.0000    0.9919
    1.0000    0.9924
    1.0000    0.9928
      ⋮
linreg = 2×1
    0.0052
    0.9930
GOFMatrix = 2×2
         0         0
    0.0052    0.9930
pred = 1×6210
    1.0364    1.0369    1.0374    1.0379    1.0384    1.0389    1.0393    1.0398 ⋯
observed = 6210×1
    1.0031
    1.0026
    1.0028
    1.0027
    1.0028
    1.0028
    1.0024
    1.0018
    1.0015
    1.0017
      ⋮
observed2 = 6210×2
    1.0000    1.0031
    1.0000    1.0026
    1.0000    1.0028
    1.0000    1.0027
    1.0000    1.0028
    1.0000    1.0028
    1.0000    1.0024
    1.0000    1.0018
    1.0000    1.0015
```

```
    1.0000    1.0017
      ⋮
linreg = 2×1
    0.0315
    0.9707
GOFMatrix = 3×2
         0         0
    0.0052    0.9930
    0.0315    0.9707
pred = 1×6210
    0.8769    0.8772    0.8775    0.8778    0.8781    0.8784    0.8787    0.8790 ⋯
observed = 6210×1
    0.9746
    0.9779
    0.9802
    0.9828
    0.9854
    0.9882
    0.9900
    0.9927
    0.9946
    0.9969
      ⋮
observed2 = 6210×2
    1.0000    0.9746
    1.0000    0.9779
    1.0000    0.9802
    1.0000    0.9828
    1.0000    0.9854
    1.0000    0.9882
    1.0000    0.9900
    1.0000    0.9927
    1.0000    0.9946
    1.0000    0.9969
      ⋮
linreg = 2×1
    0.0524
    0.9403
GOFMatrix = 4×2
         0         0
    0.0052    0.9930
    0.0315    0.9707
    0.0524    0.9403
pred = 1×6210
    0.9080    0.9080    0.9080    0.9080    0.9081    0.9081    0.9081    0.9081 ⋯
observed = 6210×1
    0.9723
    0.9755
    0.9785
    0.9812
    0.9830
    0.9854
    0.9879
    0.9902
    0.9922
    0.9938
      ⋮
observed2 = 6210×2
    1.0000    0.9723
    1.0000    0.9755
    1.0000    0.9785
```

```
    1.0000    0.9812
    1.0000    0.9830
    1.0000    0.9854
    1.0000    0.9879
    1.0000    0.9902
    1.0000    0.9922
    1.0000    0.9938
      ⋮

linreg = 2×1
    0.3699
    0.5611
GOFMatrix = 5×2
         0         0
    0.0052    0.9930
    0.0315    0.9707
    0.0524    0.9403
    0.3699    0.5611
GOF_wtlps50 = 5×2
         0         0
    0.0052    0.9930
    0.0315    0.9707
    0.0524    0.9403
    0.3699    0.5611
```

```
GOF_wtlps50_2 = PolyGOF(wt_lps50(10:end,1:end), wt_Coeffs_lps50_minus10,4)
```

```
GOFMatrix = 1×2
     0     0
pred = 1×6201
    1.0238    1.0247    1.0256    1.0264    1.0273    1.0281    1.0290    1.0299 ⋯
observed = 6201×1
    0.9928
    0.9932
    0.9939
    0.9946
    0.9949
    0.9952
    0.9960
    0.9963
    0.9971
    0.9983
      ⋮

observed2 = 6201×2
    1.0000    0.9928
    1.0000    0.9932
    1.0000    0.9939
    1.0000    0.9946
    1.0000    0.9949
    1.0000    0.9952
    1.0000    0.9960
    1.0000    0.9963
    1.0000    0.9971
    1.0000    0.9983
      ⋮

linreg = 2×1
    0.0052
    0.9930
GOFMatrix = 2×2
         0         0
    0.0052    0.9930
pred = 1×6201
```

```
    1.0427    1.0432    1.0437    1.0442    1.0447    1.0451    1.0456    1.0461 ···
observed = 6201×1
    1.0017
    1.0016
    1.0018
    1.0019
    1.0015
    1.0016
    1.0008
    1.0006
    1.0005
    1.0005
      ⋮
observed2 = 6201×2
    1.0000    1.0017
    1.0000    1.0016
    1.0000    1.0018
    1.0000    1.0019
    1.0000    1.0015
    1.0000    1.0016
    1.0000    1.0008
    1.0000    1.0006
    1.0000    1.0005
    1.0000    1.0005
      ⋮
linreg = 2×1
    0.0313
    0.9708
GOFMatrix = 3×2
         0         0
    0.0052    0.9930
    0.0313    0.9708
pred = 1×6201
    0.8739    0.8742    0.8745    0.8748    0.8751    0.8754    0.8757    0.8760 ···
observed = 6201×1
    0.9969
    0.9986
    1.0007
    1.0004
    1.0012
    1.0020
    1.0028
    1.0038
    1.0043
    1.0046
      ⋮
observed2 = 6201×2
    1.0000    0.9969
    1.0000    0.9986
    1.0000    1.0007
    1.0000    1.0004
    1.0000    1.0012
    1.0000    1.0020
    1.0000    1.0028
    1.0000    1.0038
    1.0000    1.0043
    1.0000    1.0046
      ⋮
linreg = 2×1
    0.0509
    0.9420
```

```
GOFMatrix = 4×2
         0         0
    0.0052    0.9930
    0.0313    0.9708
    0.0509    0.9420
pred = 1×6201
    0.9042    0.9042    0.9042    0.9042    0.9043    0.9043    0.9043    0.9043 ⋯
observed = 6201×1
    0.9938
    0.9952
    0.9968
    0.9984
    0.9996
    1.0006
    1.0018
    1.0029
    1.0035
    1.0041
      ⋮
observed2 = 6201×2
    1.0000    0.9938
    1.0000    0.9952
    1.0000    0.9968
    1.0000    0.9984
    1.0000    0.9996
    1.0000    1.0006
    1.0000    1.0018
    1.0000    1.0029
    1.0000    1.0035
    1.0000    1.0041
      ⋮
linreg = 2×1
    0.3714
    0.5592
GOFMatrix = 5×2
         0         0
    0.0052    0.9930
    0.0313    0.9708
    0.0509    0.9420
    0.3714    0.5592
GOF_wtlps50_2 = 5×2
         0         0
    0.0052    0.9930
    0.0313    0.9708
    0.0509    0.9420
    0.3714    0.5592
```

```
GOF_wtlps50_3 = PolyGOF(wt_lps50(20:end,1:end), wt_Coeffs_lps50_minus20,4)
```

```
GOFMatrix = 1×2
     0     0
pred = 1×6191
    1.0343    1.0352    1.0360    1.0368    1.0377    1.0385    1.0393    1.0402 ⋯
observed = 6191×1
    0.9995
    0.9999
    1.0001
    1.0000
    0.9999
    1.0005
    1.0013
    1.0010
```

```
    1.0010
    1.0004
      :
      :
observed2 = 6191×2
    1.0000    0.9995
    1.0000    0.9999
    1.0000    1.0001
    1.0000    1.0000
    1.0000    0.9999
    1.0000    1.0005
    1.0000    1.0013
    1.0000    1.0010
    1.0000    1.0010
    1.0000    1.0004
      :
      :
linreg = 2×1
    0.0052
    0.9930
GOFMatrix = 2×2
         0         0
    0.0052    0.9930
pred = 1×6191
    1.0501    1.0506    1.0510    1.0515    1.0520    1.0524    1.0529    1.0534 ···
observed = 6191×1
    0.9999
    0.9996
    0.9989
    0.9991
    0.9990
    0.9992
    0.9992
    0.9989
    0.9994
    0.9993
      :
      :
observed2 = 6191×2
    1.0000    0.9999
    1.0000    0.9996
    1.0000    0.9989
    1.0000    0.9991
    1.0000    0.9990
    1.0000    0.9992
    1.0000    0.9992
    1.0000    0.9989
    1.0000    0.9994
    1.0000    0.9993
      :
      :
linreg = 2×1
    0.0312
    0.9710
GOFMatrix = 3×2
         0         0
    0.0052    0.9930
    0.0312    0.9710
pred = 1×6191
    0.8695    0.8699    0.8702    0.8705    0.8708    0.8712    0.8715    0.8718 ···
observed = 6191×1
    1.0049
    1.0050
    1.0049
    1.0055
```

```
    1.0050
    1.0049
    1.0041
    1.0035
    1.0031
    1.0024
      :
      :
observed2 = 6191×2
    1.0000    1.0049
    1.0000    1.0050
    1.0000    1.0049
    1.0000    1.0055
    1.0000    1.0050
    1.0000    1.0049
    1.0000    1.0041
    1.0000    1.0035
    1.0000    1.0031
    1.0000    1.0024
      :
      :
linreg = 2×1
    0.0487
    0.9445
GOFMatrix = 4×2
         0         0
    0.0052    0.9930
    0.0312    0.9710
    0.0487    0.9445
pred = 1×6191
    0.8988    0.8988    0.8989    0.8989    0.8989    0.8990    0.8990    0.8990 ···
observed = 6191×1
    1.0045
    1.0040
    1.0039
    1.0039
    1.0035
    1.0034
    1.0031
    1.0020
    1.0017
    1.0011
      :
      :
observed2 = 6191×2
    1.0000    1.0045
    1.0000    1.0040
    1.0000    1.0039
    1.0000    1.0039
    1.0000    1.0035
    1.0000    1.0034
    1.0000    1.0031
    1.0000    1.0020
    1.0000    1.0017
    1.0000    1.0011
      :
      :
linreg = 2×1
    0.3721
    0.5582
GOFMatrix = 5×2
         0         0
    0.0052    0.9930
    0.0312    0.9710
    0.0487    0.9445
```

```
    0.3721    0.5582
GOF_wtlps50_3 = 5×2
         0         0
    0.0052    0.9930
    0.0312    0.9710
    0.0487    0.9445
    0.3721    0.5582
```

```
GOF_fynlps50 = PolyGOF(fyn_lps50, FynCoeffslps50,3)
```

```
GOFMatrix = 1×2
     0     0
pred = 1×6210
    1.0112    1.0118    1.0125    1.0131    1.0138    1.0144    1.0151    1.0157 ⋯
observed = 6210×1
    1.0001
    1.0004
    1.0004
    1.0005
    1.0010
    1.0018
    1.0020
    1.0021
    1.0024
    1.0028
      ⋮
observed2 = 6210×2
    1.0000    1.0001
    1.0000    1.0004
    1.0000    1.0004
    1.0000    1.0005
    1.0000    1.0010
    1.0000    1.0018
    1.0000    1.0020
    1.0000    1.0021
    1.0000    1.0024
    1.0000    1.0028
      ⋮
linreg = 2×1
    0.0848
    0.9210
GOFMatrix = 2×2
         0         0
    0.0848    0.9210
pred = 1×6210
    0.9391    0.9398    0.9405    0.9412    0.9419    0.9425    0.9432    0.9439 ⋯
observed = 6210×1
    0.9997
    0.9999
    1.0000
    0.9999
    0.9999
    0.9999
    1.0000
    0.9999
    1.0000
    0.9995
      ⋮
observed2 = 6210×2
    1.0000    0.9997
```

```
    1.0000    0.9999
    1.0000    1.0000
    1.0000    0.9999
    1.0000    0.9999
    1.0000    0.9999
    1.0000    1.0000
    1.0000    0.9999
    1.0000    1.0000
    1.0000    0.9995
      :
      :
linreg = 2×1
    0.1864
    0.8442
GOFMatrix = 3×2
         0         0
    0.0848    0.9210
    0.1864    0.8442
pred = 1×6210
    1.0482    1.0486    1.0490    1.0494    1.0498    1.0502    1.0506    1.0509 ···
observed = 6210×1
    1.0001
    1.0006
    1.0012
    1.0012
    1.0017
    1.0019
    1.0023
    1.0026
    1.0031
    1.0036
      :
      :
observed2 = 6210×2
    1.0000    1.0001
    1.0000    1.0006
    1.0000    1.0012
    1.0000    1.0012
    1.0000    1.0017
    1.0000    1.0019
    1.0000    1.0023
    1.0000    1.0026
    1.0000    1.0031
    1.0000    1.0036
      :
      :
linreg = 2×1
    0.3860
    0.6868
GOFMatrix = 4×2
         0         0
    0.0848    0.9210
    0.1864    0.8442
    0.3860    0.6868
GOF_fynlps50 = 4×2
         0         0
    0.0848    0.9210
    0.1864    0.8442
    0.3860    0.6868
```

```
GOF_fynlps50_2 = PolyGOF(fyn_lps50(10:end,1:end), fyn_Coeffs_lps50_minus10,3)
```

```
GOFMatrix = 1×2
     0     0
```

```
pred = 1×6201
    1.0176    1.0183    1.0189    1.0195    1.0202    1.0208    1.0214    1.0220 ···
observed = 6201×1
    1.0028
    1.0030
    1.0038
    1.0044
    1.0047
    1.0052
    1.0052
    1.0059
    1.0060
    1.0061
      ⋮
observed2 = 6201×2
    1.0000    1.0028
    1.0000    1.0030
    1.0000    1.0038
    1.0000    1.0044
    1.0000    1.0047
    1.0000    1.0052
    1.0000    1.0052
    1.0000    1.0059
    1.0000    1.0060
    1.0000    1.0061
      ⋮
linreg = 2×1
    0.0850
    0.9209
GOFMatrix = 2×2
         0         0
    0.0850    0.9209
pred = 1×6201
    0.9421    0.9428    0.9435    0.9442    0.9449    0.9455    0.9462    0.9469 ···
observed = 6201×1
    0.9995
    0.9995
    0.9992
    1.0000
    1.0003
    1.0004
    1.0000
    1.0005
    1.0008
    1.0007
      ⋮
observed2 = 6201×2
    1.0000    0.9995
    1.0000    0.9995
    1.0000    0.9992
    1.0000    1.0000
    1.0000    1.0003
    1.0000    1.0004
    1.0000    1.0000
    1.0000    1.0005
    1.0000    1.0008
    1.0000    1.0007
      ⋮
linreg = 2×1
    0.1882
    0.8428
```

```
GOFMatrix = 3×2
         0         0
    0.0850    0.9209
    0.1882    0.8428
pred = 1×6201
    1.0542    1.0546    1.0550    1.0554    1.0557    1.0561    1.0565    1.0569 ⋯
observed = 6201×1
    1.0036
    1.0040
    1.0044
    1.0044
    1.0046
    1.0045
    1.0052
    1.0051
    1.0054
    1.0058
      ⋮
observed2 = 6201×2
    1.0000    1.0036
    1.0000    1.0040
    1.0000    1.0044
    1.0000    1.0044
    1.0000    1.0046
    1.0000    1.0045
    1.0000    1.0052
    1.0000    1.0051
    1.0000    1.0054
    1.0000    1.0058
      ⋮
linreg = 2×1
    0.3948
    0.6797
GOFMatrix = 4×2
         0         0
    0.0850    0.9209
    0.1882    0.8428
    0.3948    0.6797
GOF_fynlps50_2 = 4×2
         0         0
    0.0850    0.9209
    0.1882    0.8428
    0.3948    0.6797
```

```
GOF_fynlps50_3 = PolyGOF(fyn_lps50(20:end,1:end), fyn_Coeffs_lps50_minus20,3)
```

```
GOFMatrix = 1×2
     0     0
pred = 1×6191
    1.0248    1.0255    1.0261    1.0267    1.0273    1.0279    1.0285    1.0291 ⋯
observed = 6191×1
    1.0066
    1.0065
    1.0066
    1.0074
    1.0080
    1.0082
    1.0088
    1.0094
    1.0100
    1.0107
```

```
      ⋮
observed2 = 6191×2
    1.0000    1.0066
    1.0000    1.0065
    1.0000    1.0066
    1.0000    1.0074
    1.0000    1.0080
    1.0000    1.0082
    1.0000    1.0088
    1.0000    1.0094
    1.0000    1.0100
    1.0000    1.0107
      ⋮
linreg = 2×1
    0.0850
    0.9208
GOFMatrix = 2×2
         0         0
    0.0850    0.9208
pred = 1×6191
    0.9456    0.9463    0.9470    0.9477    0.9483    0.9490    0.9497    0.9503 ⋯
observed = 6191×1
    1.0008
    1.0003
    1.0003
    1.0005
    1.0011
    1.0008
    1.0004
    1.0007
    1.0007
    1.0008
      ⋮
observed2 = 6191×2
    1.0000    1.0008
    1.0000    1.0003
    1.0000    1.0003
    1.0000    1.0005
    1.0000    1.0011
    1.0000    1.0008
    1.0000    1.0004
    1.0000    1.0007
    1.0000    1.0007
    1.0000    1.0008
      ⋮
linreg = 2×1
    0.1905
    0.8409
GOFMatrix = 3×2
         0         0
    0.0850    0.9208
    0.1905    0.8409
pred = 1×6191
    1.0610    1.0614    1.0617    1.0621    1.0624    1.0628    1.0632    1.0635 ⋯
observed = 6191×1
    1.0062
    1.0066
    1.0066
    1.0067
    1.0067
    1.0062
```

```
    1.0063
    1.0066
    1.0069
    1.0070
      ⋮

observed2 = 6191×2
    1.0000    1.0062
    1.0000    1.0066
    1.0000    1.0066
    1.0000    1.0067
    1.0000    1.0067
    1.0000    1.0062
    1.0000    1.0063
    1.0000    1.0066
    1.0000    1.0069
    1.0000    1.0070
      ⋮

linreg = 2×1
    0.4046
    0.6718
GOFMatrix = 4×2
         0         0
    0.0850    0.9208
    0.1905    0.8409
    0.4046    0.6718
GOF_fynlps50_3 = 4×2
         0         0
    0.0850    0.9208
    0.1905    0.8409
    0.4046    0.6718
```

Poly fits for WT and Fyn-/- responses to 100ug LPS

multiPlotCoeffs takes the coefficients collected by CollectCoefficients and plots their mean+/-SE. This is recreated in GraphPad by just taking the raw data and plotting these figures in that program, for a cleaner look.

```
figure
hold on
multiPlotCoeffs(WTCoeffs.',FynCoeffs.',5)
```

```
ans = 
  Bar with properties:

    BarLayout: 'grouped'
     BarWidth: 0.8000
    FaceColor: 'flat'
    EdgeColor: [0 0 0]
    BaseValue: 0
        XData: [1 2]
        YData: [-3.8312e-06 2.1365e-04]

  Show all properties
ans = 
  Bar with properties:

    BarLayout: 'grouped'
     BarWidth: 0.8000
    FaceColor: 'flat'
```

```
    EdgeColor: [0 0 0]
    BaseValue: 0
        XData: [1 2]
        YData: [1.1265e-04 -0.0071]

  Show all properties
ans =
  Bar with properties:

    BarLayout: 'grouped'
     BarWidth: 0.8000
    FaceColor: 'flat'
    EdgeColor: [0 0 0]
    BaseValue: 0
        XData: [1 2]
        YData: [-0.0016 0.0874]

  Show all properties
ans =
  Bar with properties:

    BarLayout: 'grouped'
     BarWidth: 0.8000
    FaceColor: 'flat'
    EdgeColor: [0 0 0]
    BaseValue: 0
        XData: [1 2]
        YData: [0.0194 -0.4876]

  Show all properties
ans =
  Bar with properties:

    BarLayout: 'grouped'
     BarWidth: 0.8000
    FaceColor: 'flat'
    EdgeColor: [0 0 0]
    BaseValue: 0
        XData: [1 2]
        YData: [-0.1443 1.0811]

  Show all properties
```

```
hold off
```

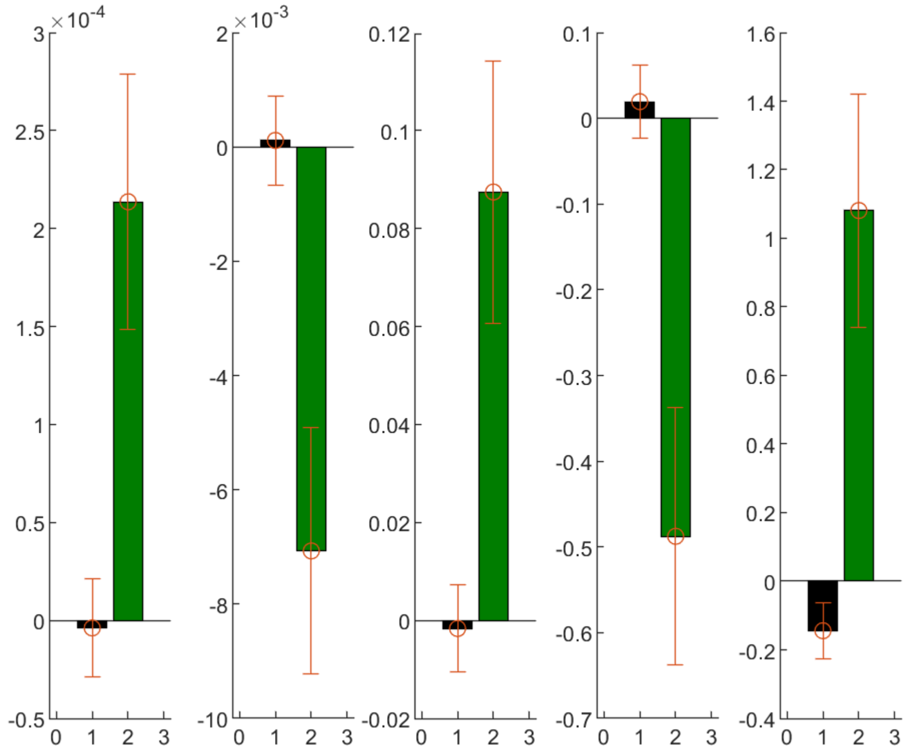


```
figure
hold on
multiPlotCoeffs(WTCoeffslps50.', FynCoeffslps50.',5)
```

```
ans =
  Bar with properties:

    BarLayout: 'grouped'
     BarWidth: 0.8000
    FaceColor: 'flat'
    EdgeColor: [0 0 0]
    BaseValue: 0
        XData: [1 2]
        YData: [1.4316e-05 1.5297e-05]

  Show all properties
ans =
  Bar with properties:

    BarLayout: 'grouped'
     BarWidth: 0.8000
    FaceColor: 'flat'
    EdgeColor: [0 0 0]
    BaseValue: 0
        XData: [1 2]
        YData: [-6.2411e-04 -6.9585e-04]

  Show all properties
ans =
  Bar with properties:

    BarLayout: 'grouped'
```

```
     BarWidth: 0.8000
    FaceColor: 'flat'
    EdgeColor: [0 0 0]
    BaseValue: 0
        XData: [1 2]
        YData: [0.0100 0.0115]

  Show all properties
ans = 
  Bar with properties:

    BarLayout: 'grouped'
     BarWidth: 0.8000
    FaceColor: 'flat'
    EdgeColor: [0 0 0]
    BaseValue: 0
        XData: [1 2]
        YData: [-0.0705 -0.0815]

  Show all properties
ans = 
  Bar with properties:

    BarLayout: 'grouped'
     BarWidth: 0.8000
    FaceColor: 'flat'
    EdgeColor: [0 0 0]
    BaseValue: 0
        XData: [1 2]
        YData: [0.1706 0.2291]

  Show all properties
```

```
hold off
```

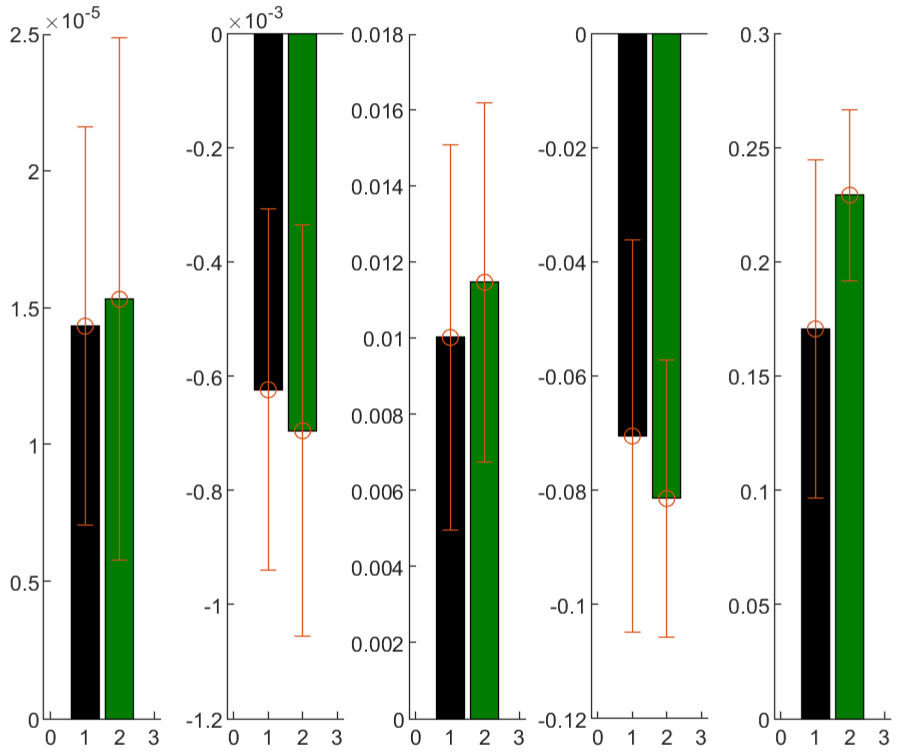

```
figure
hold on
multiPlotCoeffs(unnWTCoeffslps50.', unnFynCoeffslps50.',6)
```

```
ans =
  Bar with properties:

    BarLayout: 'grouped'
     BarWidth: 0.8000
    FaceColor: 'flat'
    EdgeColor: [0 0 0]
    BaseValue: 0
        XData: [1 2]
        YData: [0.0147 0.0073]

  Show all properties
ans =
  Bar with properties:

    BarLayout: 'grouped'
     BarWidth: 0.8000
    FaceColor: 'flat'
    EdgeColor: [0 0 0]
    BaseValue: 0
        XData: [1 2]
        YData: [-0.6319 -0.3924]

  Show all properties
ans =
  Bar with properties:

    BarLayout: 'grouped'
     BarWidth: 0.8000
    FaceColor: 'flat'
    EdgeColor: [0 0 0]
    BaseValue: 0
        XData: [1 2]
        YData: [9.9681 7.6675]

  Show all properties
ans =
  Bar with properties:

    BarLayout: 'grouped'
     BarWidth: 0.8000
    FaceColor: 'flat'
    EdgeColor: [0 0 0]
    BaseValue: 0
        XData: [1 2]
        YData: [-68.7718 -65.7296]

  Show all properties
ans =
  Bar with properties:

    BarLayout: 'grouped'
     BarWidth: 0.8000
    FaceColor: 'flat'
    EdgeColor: [0 0 0]
    BaseValue: 0
        XData: [1 2]
        YData: [153.4398 235.4257]
```

Show all properties

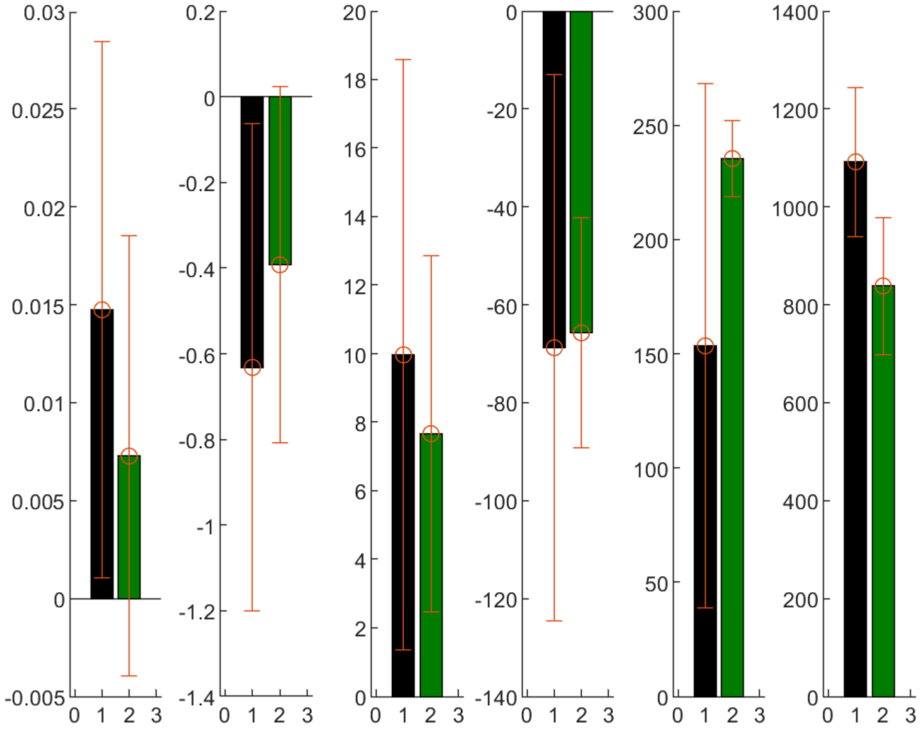


```
ans =
  Bar with properties:

    BarLayout: 'grouped'
     BarWidth: 0.8000
    FaceColor: 'flat'
    EdgeColor: [0 0 0]
    BaseValue: 0
        XData: [1 2]
        YData: [1.0920e+03 838.3303]

  Show all properties
```

Raw data plots for WT and Fyn-/- respones to 50ug LPS

In an earlier iteration, we looked at both normalized and un-normalized data. So, we have plots and fits here for both normalized and un-normalized data for the 50ug LPS ECIS curves.

```
figure
hold on
t3=1:length(wt_lps50.');
t4=1:length(fyn_lps50.');
t3=t3/400;
t4=t4/400;

plot((1:length(wt_lps50)).'/400, mean(wt_lps50.'),(1:length(fyn_lps50)).'/400, mean(fyn_lps50.'
```

```
plot(t3.', mean(wt_lps50.')+std(wt_lps50.')/sqrt(4), 'k--')
plot(t3.', mean(wt_lps50.')-std(wt_lps50.')/sqrt(4), 'k--')

plot(t4.', mean(fyn_lps50.')+std(fyn_lps50.')/sqrt(3), 'k--')
plot(t4.', mean(fyn_lps50.')-std(fyn_lps50.')/sqrt(3), 'k--')
ylim([0,2.5])
hold off
```

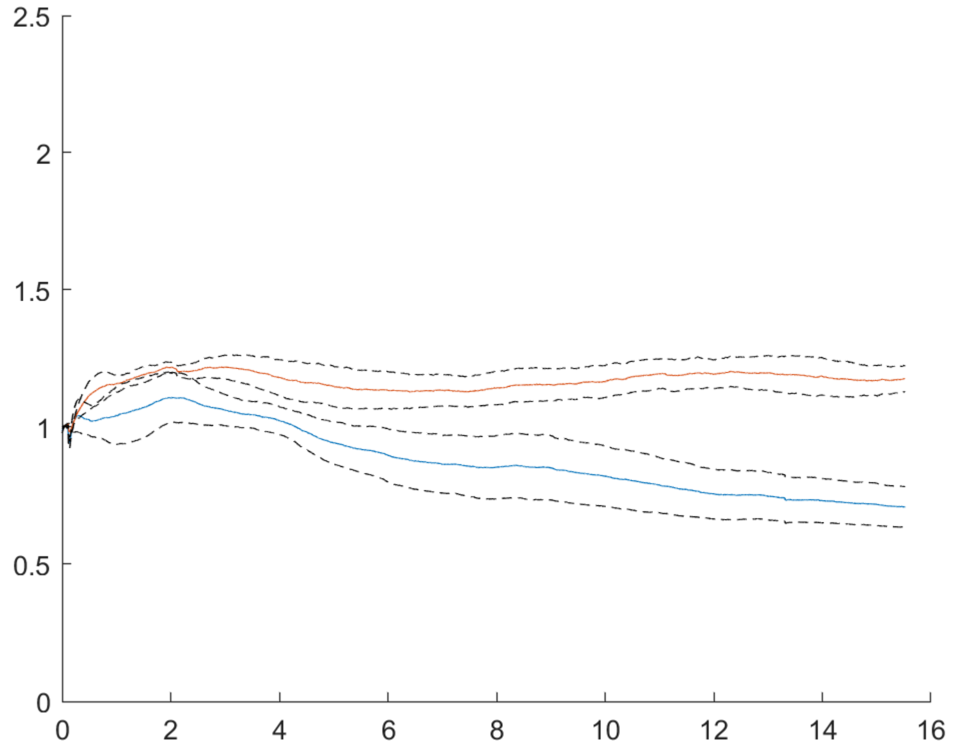


```
% check t-test at a static time point
[h,p] = ttest(wt_lps50(4800,1:3), fyn_lps50(4800,1:3))
```

```
h = 1
p = 0.0384
```

```
figure
hold on
t3=1:length(unn_wt50.');
t4=1:length(unn_fyn50.');
t3=t3/400;
t4=t4/400;
plot((1:length(unn_wt50)).'/400, mean(unn_wt50.'),(1:length(unn_fyn50)).'/400, mean(unn_fyn50.'
plot(t3.', mean(unn_wt50.')+std(unn_wt50.')/sqrt(4), 'k--')
plot(t3.', mean(unn_wt50.')-std(unn_wt50.')/sqrt(4), 'k--')

plot(t4.', mean(unn_fyn50.')+std(unn_fyn50.')/sqrt(3), 'k--')
plot(t4.', mean(unn_fyn50.')-std(unn_fyn50.')/sqrt(3), 'k--')
hold off
```

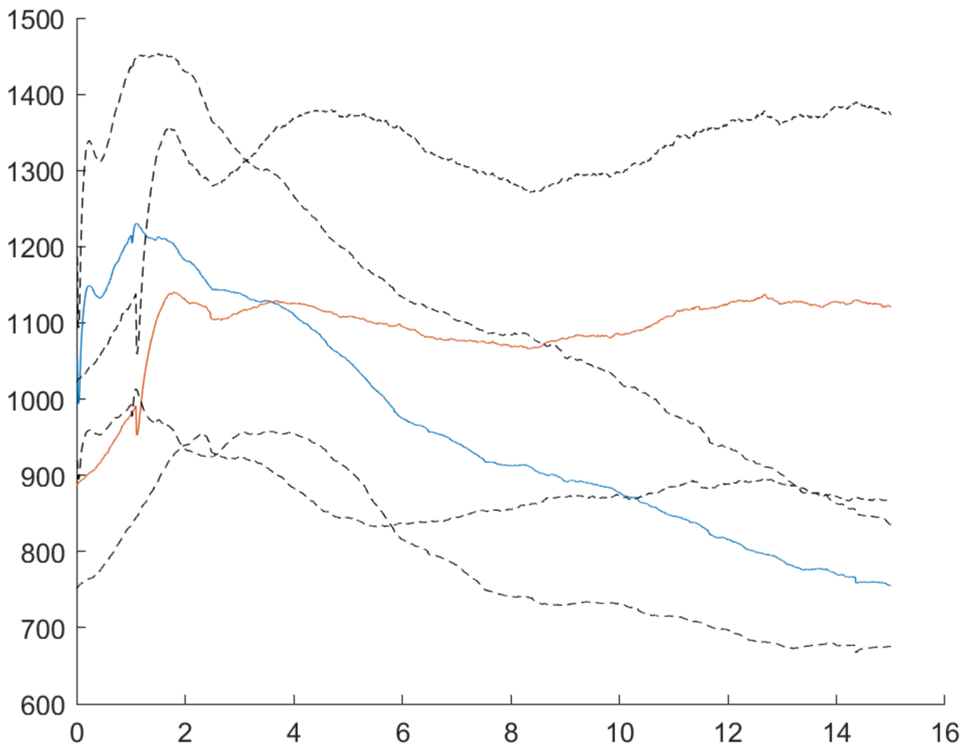

Polynomial fits of Thrombin +/- DEVD in MLMVECs

```
% run poly fits for thrombin with and without DEVD
plotFits(thrombin,5,2,0.5,1.3);
```

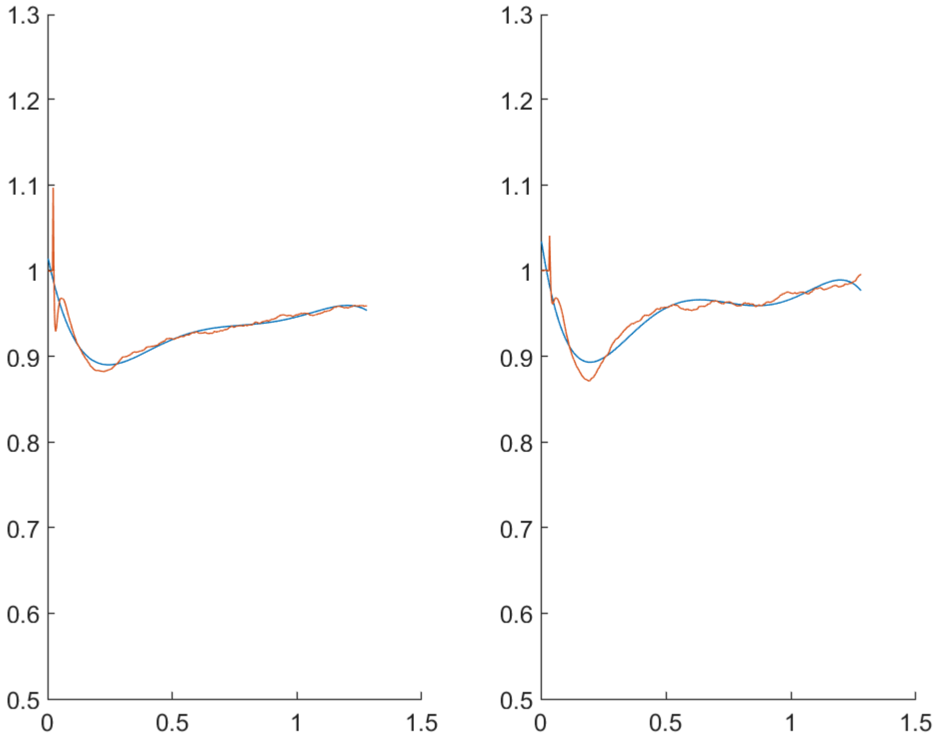


```
plotFits(thrombindevd,5,2,0.5,1.3);
```

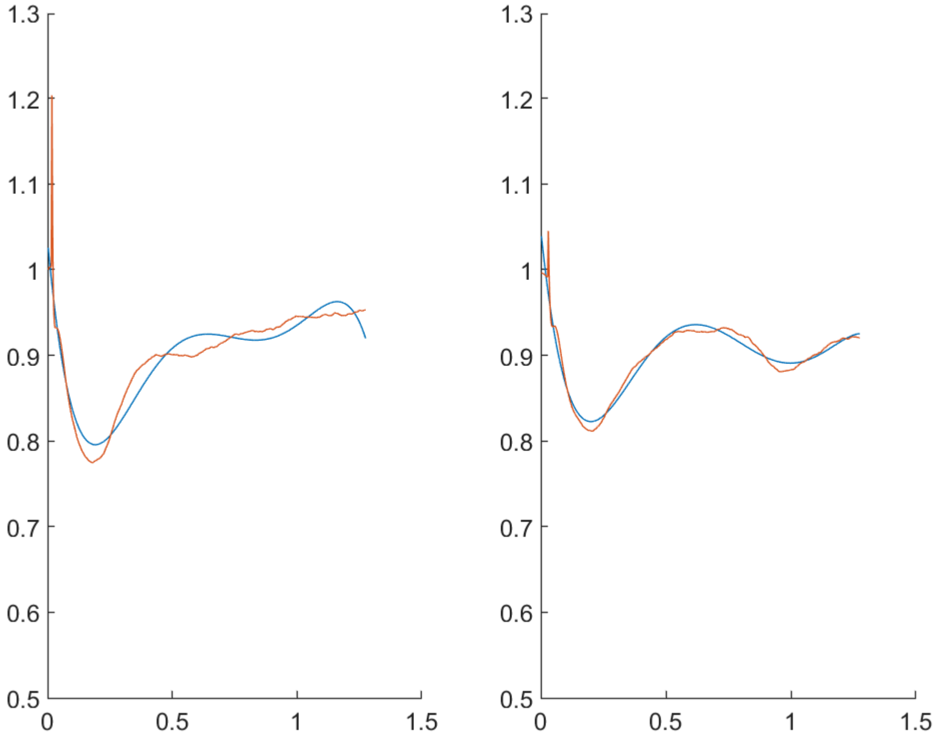

```
multiPlotCoeffs(thrombinCoeffs.', thrombindevdCoeffs.',5);
```

```
ans = 
  Bar with properties:

    BarLayout: 'grouped'
     BarWidth: 0.8000
    FaceColor: 'flat'
    EdgeColor: [0 0 0]
    BaseValue: 0
        XData: [1 2]
        YData: [-3.5564 -5.1518]

  Show all properties
ans = 
  Bar with properties:

    BarLayout: 'grouped'
     BarWidth: 0.8000
    FaceColor: 'flat'
    EdgeColor: [0 0 0]
    BaseValue: 0
        XData: [1 2]
        YData: [12.6849 19.0441]

  Show all properties
ans = 
  Bar with properties:

    BarLayout: 'grouped'
     BarWidth: 0.8000
    FaceColor: 'flat'
    EdgeColor: [0 0 0]
    BaseValue: 0
        XData: [1 2]
        YData: [-16.4691 -25.9404]

  Show all properties
ans = 
  Bar with properties:

    BarLayout: 'grouped'
     BarWidth: 0.8000
    FaceColor: 'flat'
    EdgeColor: [0 0 0]
    BaseValue: 0
        XData: [1 2]
        YData: [9.2766 15.6192]

  Show all properties
```

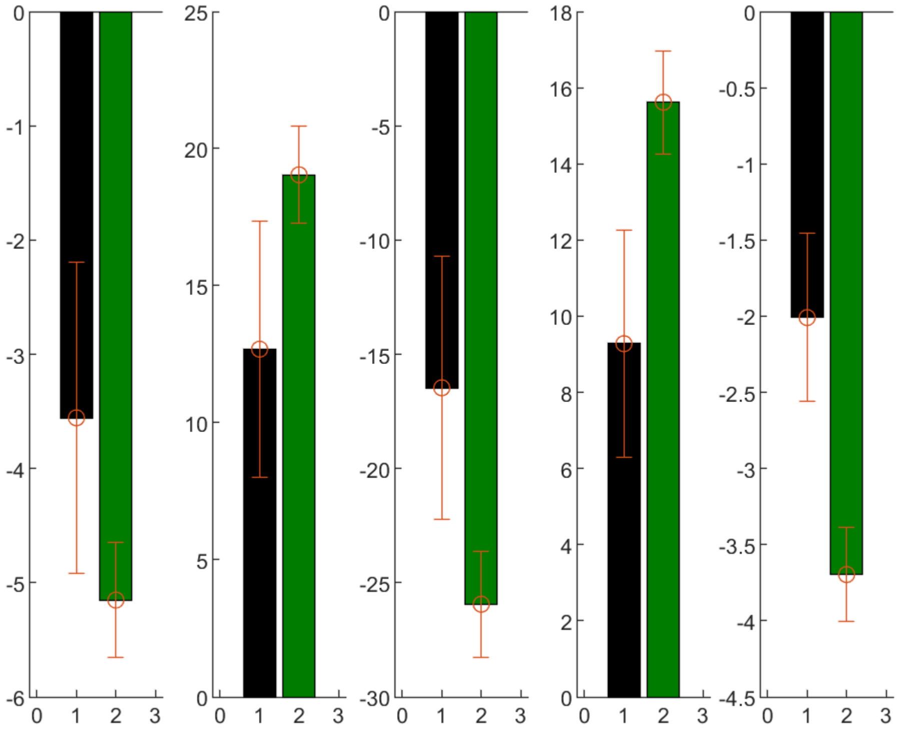


```
ans =
  Bar with properties:

    BarLayout: 'grouped'
     BarWidth: 0.8000
    FaceColor: 'flat'
    EdgeColor: [0 0 0]
    BaseValue: 0
        XData: [1 2]
        YData: [-2.0093 -3.6963]

  Show all properties
```

Polynomial fits of unnormalized wt and fyn 100ug LPS response

```
figure
multiPlotCoeffs(unnWTCoeffs.', unnFynCoeffs.', 6);
```

```
ans =
  Bar with properties:

    BarLayout: 'grouped'
     BarWidth: 0.8000
    FaceColor: 'flat'
    EdgeColor: [0 0 0]
    BaseValue: 0
        XData: [1 2]
        YData: [-0.0065 0.0126]

  Show all properties
ans =
  Bar with properties:

    BarLayout: 'grouped'
```

```
     BarWidth: 0.8000
    FaceColor: 'flat'
    EdgeColor: [0 0 0]
    BaseValue: 0
        XData: [1 2]
        YData: [0.2328 -0.6513]

  Show all properties
ans =
  Bar with properties:

    BarLayout: 'grouped'
     BarWidth: 0.8000
    FaceColor: 'flat'
    EdgeColor: [0 0 0]
    BaseValue: 0
        XData: [1 2]
        YData: [-2.6632 12.4610]

  Show all properties
ans =
  Bar with properties:

    BarLayout: 'grouped'
     BarWidth: 0.8000
    FaceColor: 'flat'
    EdgeColor: [0 0 0]
    BaseValue: 0
        XData: [1 2]
        YData: [13.0555 -104.7524]

  Show all properties
ans =
  Bar with properties:

    BarLayout: 'grouped'
     BarWidth: 0.8000
    FaceColor: 'flat'
    EdgeColor: [0 0 0]
    BaseValue: 0
        XData: [1 2]
        YData: [-79.8018 334.9048]

  Show all properties
```

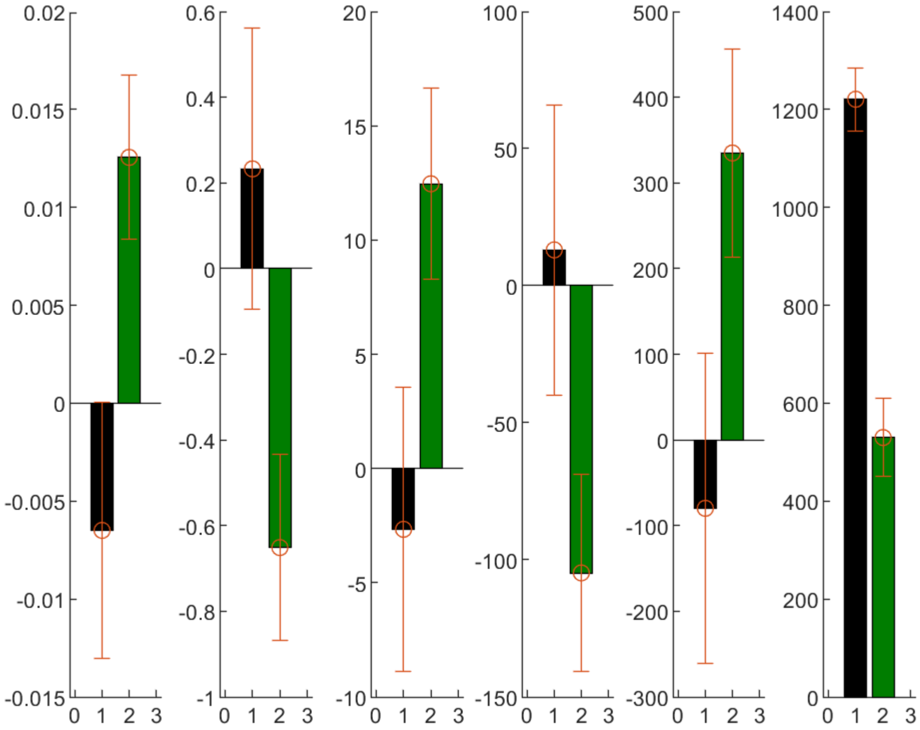


```
ans =
  Bar with properties:

    BarLayout: 'grouped'
     BarWidth: 0.8000
    FaceColor: 'flat'
    EdgeColor: [0 0 0]
    BaseValue: 0
        XData: [1 2]
        YData: [1.2209e+03 529.8618]

  Show all properties
```

Polynomial Fits of WT, CD36 and Fyn-/- MLMVEC GSK responses

```
plotFits(WT_GSK,5,2, 0.7,1.3);
```

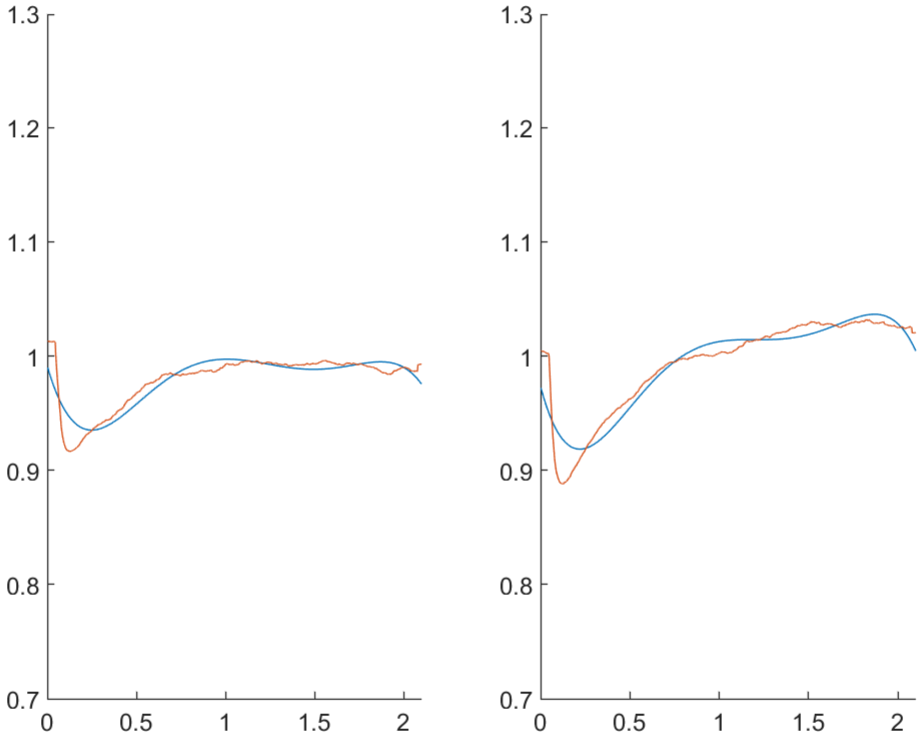


```
plotFits(CD36_GSK, 5,2,0.7,1.3);
```

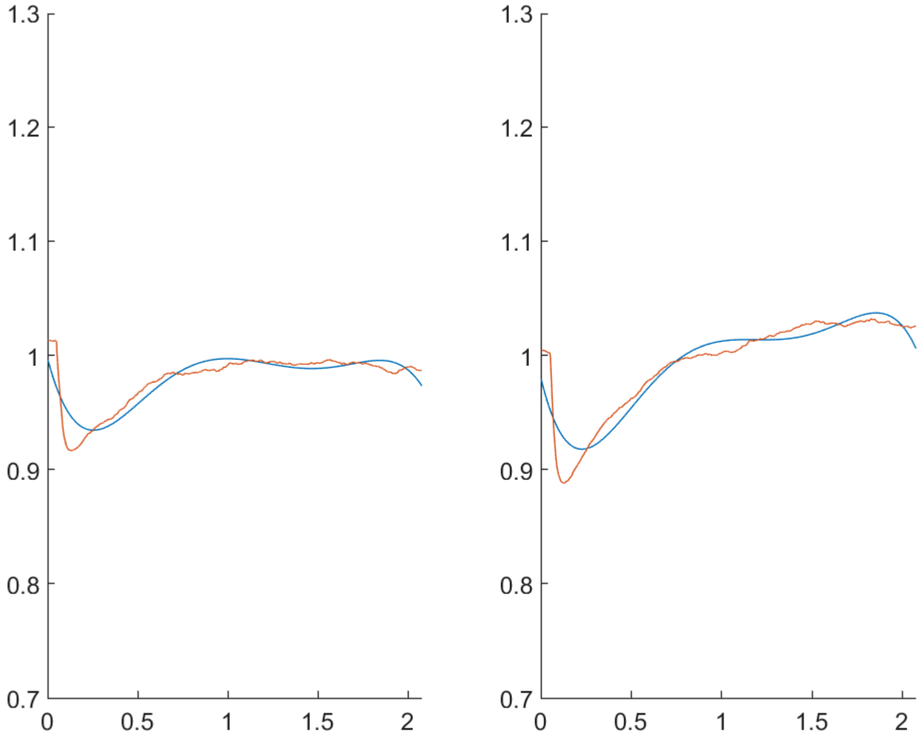

```
plotFits(fyn_GSK, 5,2,0.7,1.3);
```

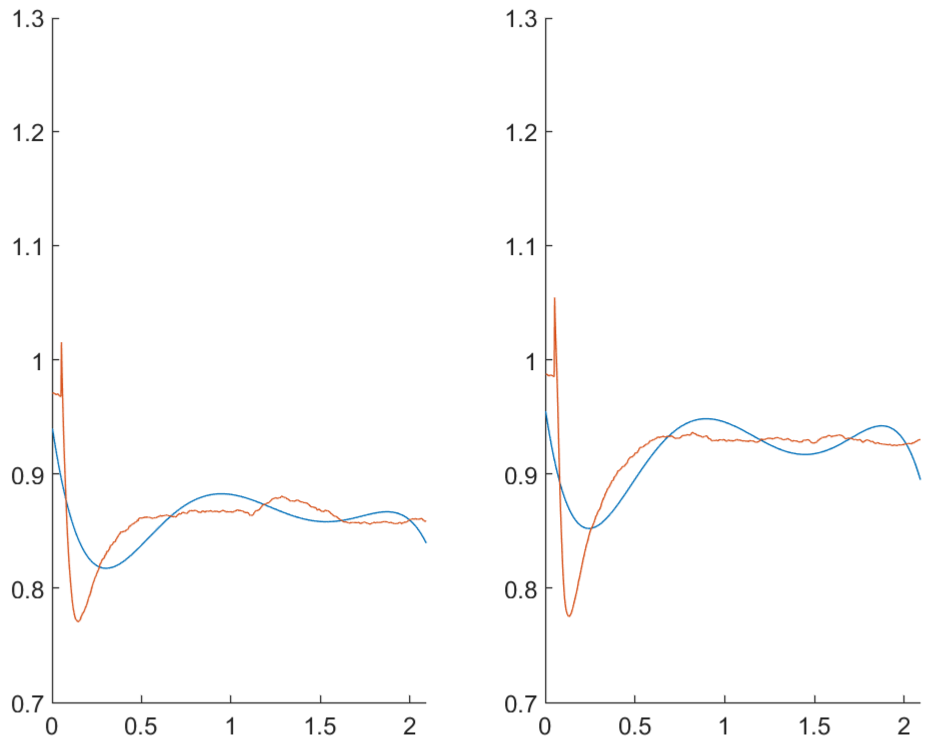


```
plotFits(unn_WT_GSK,5,2, 0,2000);
```

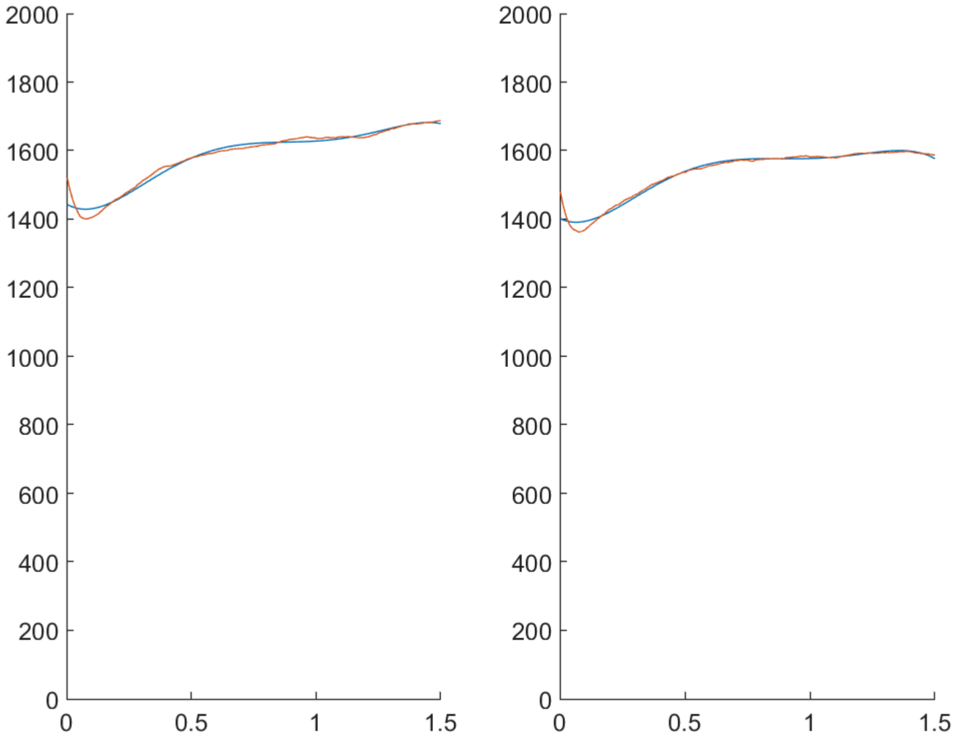


```
plotFits(unn_CD36_GSK, 5,2,0,1500);
```

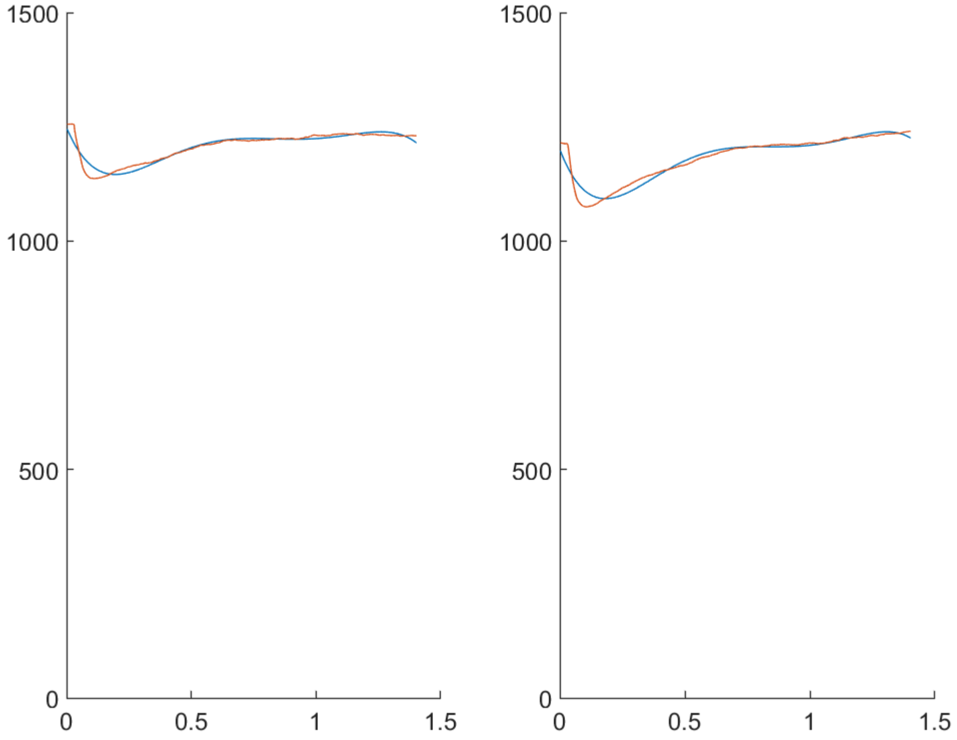

```
plotFits(unn_Fyn_GSK, 5,2,500,800);
```

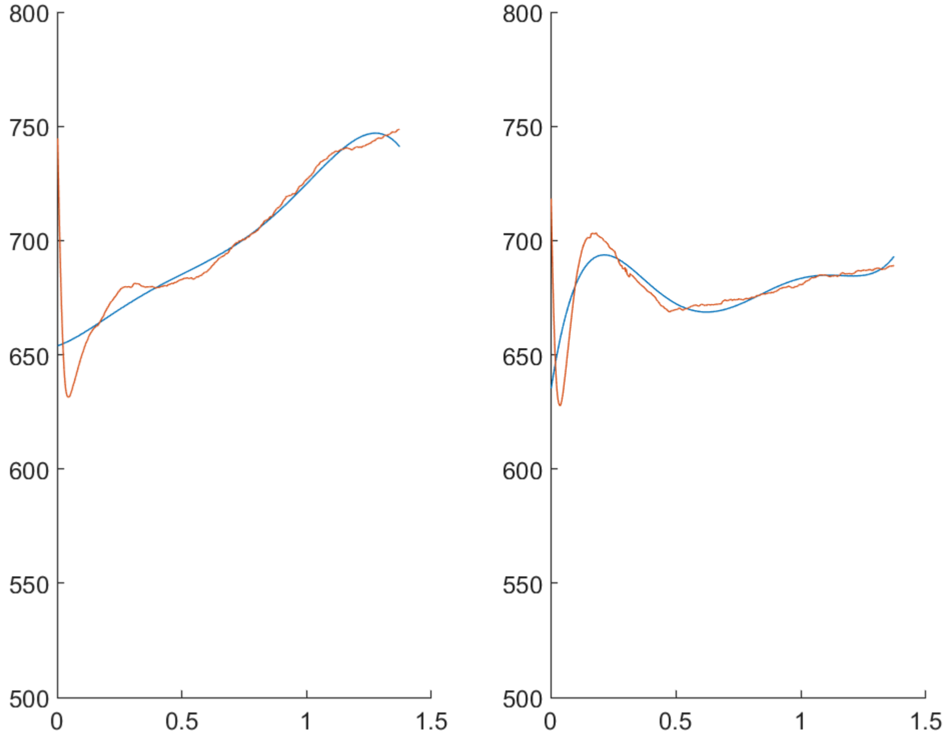


```
figure
hold on
multiPlotCoeffs3way(WT_GSK_Coeffs.', CD36_GSK_Coeffs.', fyn_GSK_Coeffs.',5);
hold off
```

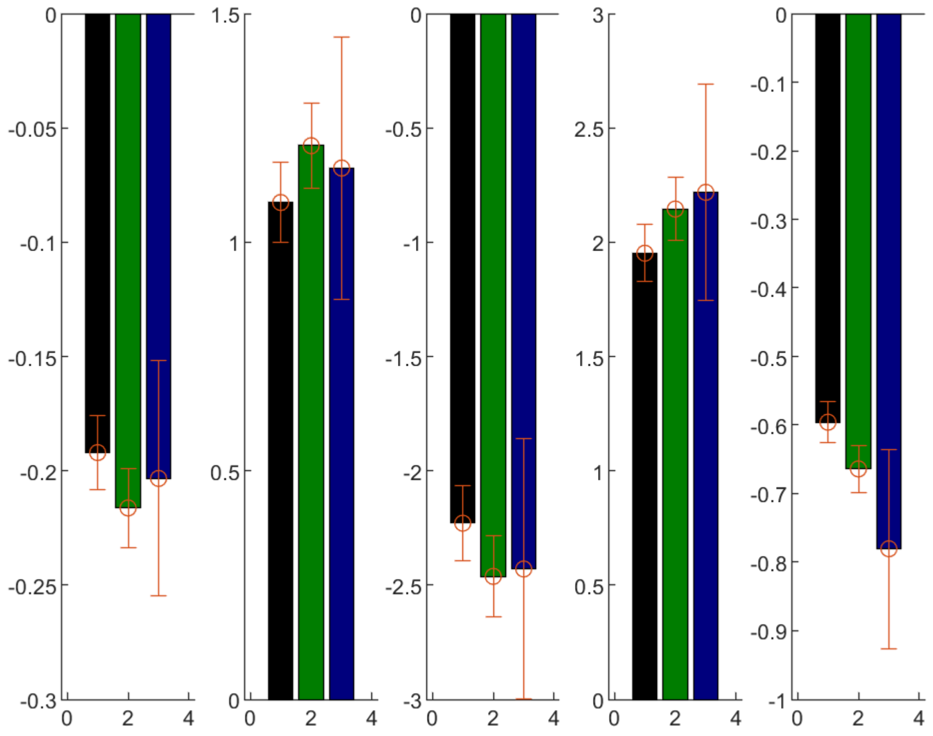


```
figure
hold on
multiPlotCoeffs3way(WT_GSK_Coeffs_minus10.', CD36_GSK_Coeffs_minus10.', fyn_GSK_Coeffs_minus10.
hold off
```

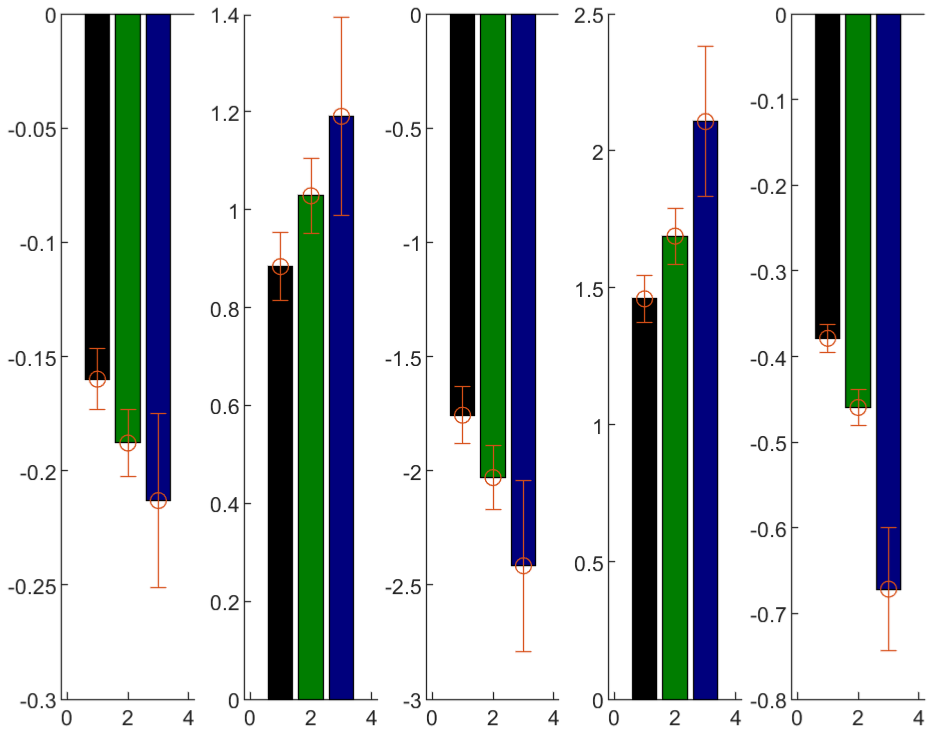


```
figure
hold on
multiPlotCoeffs3way(WT_GSK_Coeffs_minus20.', CD36_GSK_Coeffs_minus20.', fyn_GSK_Coeffs_minus20.
hold off
```

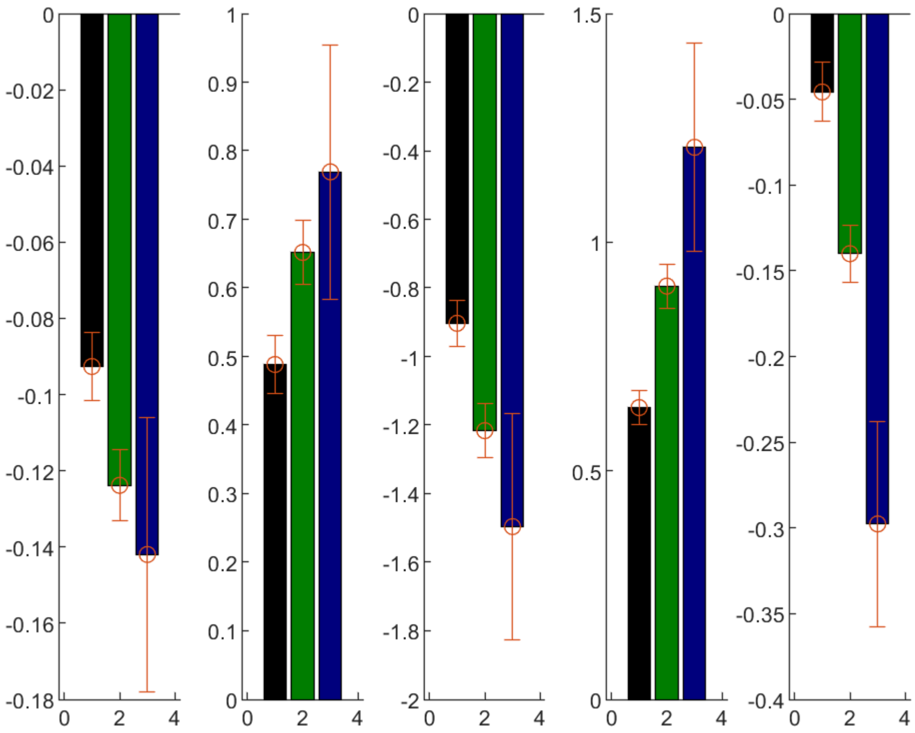

Correlations between the coefficients

```
%look at correlations between coefficients
figure
hold on
%corrplot(unnFynCoeffslps50, 'varNames', {"x^5", "x^4", "x^3", "x^2", "x", "c"}, 'testR', 'on')
hold off
```

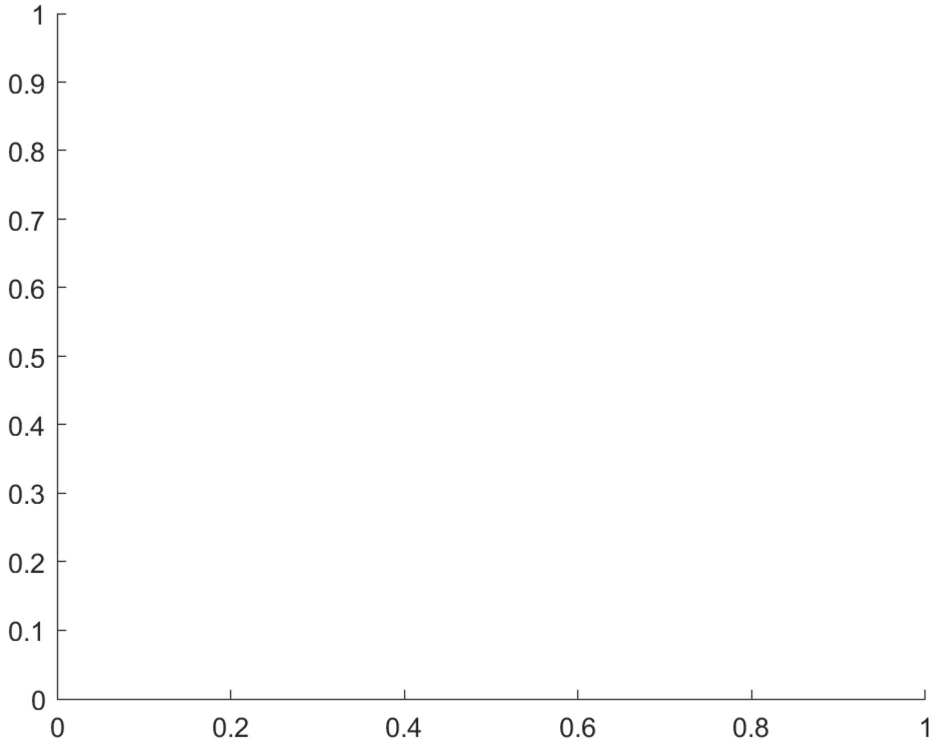

Determine TERmax for fits and compare these values to the observed TERmax values

```
% let's start with GSK
% collect fitted TERm
TERm_fitted = zeros(1,6)
```

```
TERm_fitted = 1×6
     0     0     0     0     0     0
```

```
for i=1:6
TERm = roots(polyder(WT_GSK_Coeffs(i,:)))
TERm_fitted(i) = max(1-TERm)
end
```

```
TERm = 4×1
    1.8703
    1.4950
    1.0097
    0.2492
TERm_fitted = 1×6
    0.7508         0         0         0         0         0
TERm = 4×1
    1.8695
    1.2329
    1.1726
    0.2201
TERm_fitted = 1×6
    0.7508    0.7799         0         0         0         0
TERm = 4×1
    1.8935
```

```
    1.3416
    0.9281
    0.2250
TERm_fitted = 1×6
    0.7508    0.7799    0.7750         0         0         0
TERm = 4×1
    1.8708
    1.3670
    1.0764
    0.2515
TERm_fitted = 1×6
    0.7508    0.7799    0.7750    0.7485         0         0
TERm = 4×1
    1.8741
    1.5045
    0.9569
    0.2157
TERm_fitted = 1×6
    0.7508    0.7799    0.7750    0.7485    0.7843         0
TERm = 4×1
   1.8391
   1.2603
   1.2603
   0.2339
TERm_fitted = 1×6
    0.7508    0.7799    0.7750    0.7485    0.7843   -0.8391
```

```
% collect observed Term
TERm_observed=zeros(1,6)
```

```
TERm_observed = 1×6
     0     0     0     0     0     0
```

```
for i=1:6
TERm_observed(i) = min(WT_GSK(:,i))
end
```

```
TERm_observed = 1×6
    0.9164         0         0         0         0         0
TERm_observed = 1×6
    0.9164    0.8880         0         0         0         0
TERm_observed = 1×6
    0.9164    0.8880    0.8993         0         0         0
TERm_observed = 1×6
    0.9164    0.8880    0.8993    0.9182         0         0
TERm_observed = 1×6
    0.9164    0.8880    0.8993    0.9182    0.8783         0
TERm_observed = 1×6
    0.9164    0.8880    0.8993    0.9182    0.8783    0.9059
```

```
% repeat above for Thrombin

TERm_fitted_thrombin = zeros(1,6)
```

```
TERm_fitted_thrombin = 1×6
     0     0     0     0     0     0
```

```
for i=1:6
TERm = roots(polyder(thrombinCoeffs(i,:)))
TERm_fitted_thrombin(i) = max(1-TERm)
```

```
end
```

```
TERm = 4×1
   1.2020
   0.7736
   0.7736
   0.2439
TERm_fitted_thrombin = 1×6
    0.7561         0         0         0         0         0
TERm = 4×1
    1.1981
    0.8634
    0.6366
    0.1972
TERm_fitted_thrombin = 1×6
    0.7561    0.8028         0         0         0         0
TERm = 4×1
   1.1337
   1.1337
   0.5939
   0.2078
TERm_fitted_thrombin = 1×6
    0.7561    0.8028    0.7922         0         0         0
TERm = 4×1
    1.1986
    0.9835
    0.7293
    0.2423
TERm_fitted_thrombin = 1×6
    0.7561    0.8028    0.7922    0.7577         0         0
TERm = 4×1
    1.1660
    0.9059
    0.6156
    0.1763
TERm_fitted_thrombin = 1×6
    0.7561    0.8028    0.7922    0.7577    0.8237         0
TERm = 4×1
    1.1657
    0.9180
    0.5332
    0.1418
TERm_fitted_thrombin = 1×6
    0.7561    0.8028    0.7922    0.7577    0.8237    0.8582
```

```
% collect observed Term
TERm_observed_thrombin=zeros(1,6)
```

```
TERm_observed_thrombin = 1×6
     0     0     0     0     0     0
```

```
for i=1:6
TERm_observed_thrombin(i) = min(thrombin(:,i))
end
```

```
TERm_observed_thrombin = 1×6
    0.8821         0         0         0         0         0
TERm_observed_thrombin = 1×6
    0.8821    0.8711         0         0         0         0
TERm_observed_thrombin = 1×6
    0.8821    0.8711    0.9611         0         0         0
TERm_observed_thrombin = 1×6
```

```
    0.8821    0.8711    0.9611    0.9302         0         0
TERm_observed_thrombin = 1×6
    0.8821    0.8711    0.9611    0.9302    0.7625         0
TERm_observed_thrombin = 1×6
    0.8821    0.8711    0.9611    0.9302    0.7625    0.7821
```

```
% now for lps. here,we can't use derivative because the inflection is not crisp - will need to
TERm_fitted_lps = zeros(1,12)
```

```
TERm_fitted_lps = 1×12
     0     0     0     0     0     0     0     0     0     0     0     0
```

```
for i=1:12
TERm_fitted_lps(i) = min(polyval(WTCoeffs(i,:), 0:0.01:12))
end
```

```
TERm_fitted_lps = 1×12
    0.4043         0         0         0         0         0         0         0 ···
TERm_fitted_lps = 1×12
    0.4043    0.4170         0         0         0         0         0         0 ···
TERm_fitted_lps = 1×12
    0.4043    0.4170    0.9928         0         0         0         0         0 ···
TERm_fitted_lps = 1×12
    0.4043    0.4170    0.9928    1.0183         0         0         0         0 ···
TERm_fitted_lps = 1×12
    0.4043    0.4170    0.9928    1.0183    0.2667         0         0         0 ···
TERm_fitted_lps = 1×12
    0.4043    0.4170    0.9928    1.0183    0.2667    0.3147         0         0 ···
TERm_fitted_lps = 1×12
    0.4043    0.4170    0.9928    1.0183    0.2667    0.3147    0.2677         0 ···
TERm_fitted_lps = 1×12
    0.4043    0.4170    0.9928    1.0183    0.2667    0.3147    0.2677    0.2036 ···
TERm_fitted_lps = 1×12
    0.4043    0.4170    0.9928    1.0183    0.2667    0.3147    0.2677    0.2036 ···
TERm_fitted_lps = 1×12
    0.4043    0.4170    0.9928    1.0183    0.2667    0.3147    0.2677    0.2036 ···
TERm_fitted_lps = 1×12
    0.4043    0.4170    0.9928    1.0183    0.2667    0.3147    0.2677    0.2036 ···
TERm_fitted_lps = 1×12
    0.4043    0.4170    0.9928    1.0183    0.2667    0.3147    0.2677    0.2036 ···
```

```
% collect observed Term
TERm_observed_lps=zeros(1,12)
```

```
TERm_observed_lps = 1×12
     0     0     0     0     0     0     0     0     0     0     0     0
```

```
for i=1:12
TERm_observed_lps(i) = min(wt(:,i))
end
```

```
TERm_observed_lps = 1×12
    0.4317         0         0         0         0         0         0         0 ···
TERm_observed_lps = 1×12
    0.4317    0.4477         0         0         0         0         0         0 ···
TERm_observed_lps = 1×12
    0.4317    0.4477    0.9943         0         0         0         0         0 ···
TERm_observed_lps = 1×12
    0.4317    0.4477    0.9943    0.9972         0         0         0         0 ···
TERm_observed_lps = 1×12
```

```
    0.4317    0.4477    0.9943    0.9972    0.3355         0         0         0 ···
TERm_observed_lps = 1×12
    0.4317    0.4477    0.9943    0.9972    0.3355    0.3206         0         0 ···
TERm_observed_lps = 1×12
    0.4317    0.4477    0.9943    0.9972    0.3355    0.3206    0.3698         0 ···
TERm_observed_lps = 1×12
    0.4317    0.4477    0.9943    0.9972    0.3355    0.3206    0.3698    0.3412 ···
TERm_observed_lps = 1×12
    0.4317    0.4477    0.9943    0.9972    0.3355    0.3206    0.3698    0.3412 ···
TERm_observed_lps = 1×12
    0.4317    0.4477    0.9943    0.9972    0.3355    0.3206    0.3698    0.3412 ···
TERm_observed_lps = 1×12
    0.4317    0.4477    0.9943    0.9972    0.3355    0.3206    0.3698    0.3412 ···
TERm_observed_lps = 1×12
    0.4317    0.4477    0.9943    0.9972    0.3355    0.3206    0.3698    0.3412 ···
```

Coefficient Modification

plotPoly(a,t,p, ymin, ymax, low_step, high_step, step) polyvals the coeffs of a at times t by varying coefficient number p between low_step and high_step by step, and graphs results between ymin and ymax. Examples shown below.In the paper, we used non-scaled x axes for the fits in order to better illustrate the effects

```
WTCoeefModifx5 = plotPolyrawdata(WTCoeffs(1,:), 1:4000, 1,0,3,0.8,1.2,0.1)
```

```
rawdata = 1×4000
    1.0672    1.0675    1.0678    1.0681    1.0684    1.0686    1.0689    1.0692 ···
count = 2
rawdata = 2×4000
    1.0672    1.0675    1.0678    1.0681    1.0684    1.0686    1.0689    1.0692 ···
    1.0672    1.0675    1.0678    1.0681    1.0684    1.0686    1.0689    1.0692
count = 3
rawdata = 3×4000
    1.0672    1.0675    1.0678    1.0681    1.0684    1.0686    1.0689    1.0692 ···
    1.0672    1.0675    1.0678    1.0681    1.0684    1.0686    1.0689    1.0692
    1.0672    1.0675    1.0678    1.0681    1.0684    1.0686    1.0689    1.0692
count = 4
rawdata = 4×4000
    1.0672    1.0675    1.0678    1.0681    1.0684    1.0686    1.0689    1.0692 ···
    1.0672    1.0675    1.0678    1.0681    1.0684    1.0686    1.0689    1.0692
    1.0672    1.0675    1.0678    1.0681    1.0684    1.0686    1.0689    1.0692
    1.0672    1.0675    1.0678    1.0681    1.0684    1.0686    1.0689    1.0692
count = 5
rawdata = 5×4000
    1.0672    1.0675    1.0678    1.0681    1.0684    1.0686    1.0689    1.0692 ···
    1.0672    1.0675    1.0678    1.0681    1.0684    1.0686    1.0689    1.0692
    1.0672    1.0675    1.0678    1.0681    1.0684    1.0686    1.0689    1.0692
    1.0672    1.0675    1.0678    1.0681    1.0684    1.0686    1.0689    1.0692
    1.0672    1.0675    1.0678    1.0681    1.0684    1.0686    1.0689    1.0692
count = 6
WTCoeefModifx5 = 5×4000
    1.0672    1.0675    1.0678    1.0681    1.0684    1.0686    1.0689    1.0692 ···
    1.0672    1.0675    1.0678    1.0681    1.0684    1.0686    1.0689    1.0692
    1.0672    1.0675    1.0678    1.0681    1.0684    1.0686    1.0689    1.0692
    1.0672    1.0675    1.0678    1.0681    1.0684    1.0686    1.0689    1.0692
    1.0672    1.0675    1.0678    1.0681    1.0684    1.0686    1.0689    1.0692
```

```
WTCoeefModifx4 = plotPolyrawdata(WTCoeffs(1,:), 1:4000, 2,0,3,0.8,1.2,0.1)
```

```
rawdata = 1×4000
    1.0672    1.0675    1.0678    1.0681    1.0684    1.0686    1.0689    1.0692 ···
count = 2
rawdata = 2×4000
```

```
    1.0672    1.0675    1.0678    1.0681    1.0684    1.0686    1.0689    1.0692 ⋯
    1.0672    1.0675    1.0678    1.0681    1.0684    1.0686    1.0689    1.0692
count = 3
rawdata = 3×4000
    1.0672    1.0675    1.0678    1.0681    1.0684    1.0686    1.0689    1.0692 ⋯
    1.0672    1.0675    1.0678    1.0681    1.0684    1.0686    1.0689    1.0692
    1.0672    1.0675    1.0678    1.0681    1.0684    1.0686    1.0689    1.0692
count = 4
rawdata = 4×4000
    1.0672    1.0675    1.0678    1.0681    1.0684    1.0686    1.0689    1.0692 ⋯
    1.0672    1.0675    1.0678    1.0681    1.0684    1.0686    1.0689    1.0692
    1.0672    1.0675    1.0678    1.0681    1.0684    1.0686    1.0689    1.0692
    1.0672    1.0675    1.0678    1.0681    1.0684    1.0686    1.0689    1.0692
count = 5
rawdata = 5×4000
    1.0672    1.0675    1.0678    1.0681    1.0684    1.0686    1.0689    1.0692 ⋯
    1.0672    1.0675    1.0678    1.0681    1.0684    1.0686    1.0689    1.0692
    1.0672    1.0675    1.0678    1.0681    1.0684    1.0686    1.0689    1.0692
    1.0672    1.0675    1.0678    1.0681    1.0684    1.0686    1.0689    1.0692
    1.0672    1.0675    1.0678    1.0681    1.0684    1.0686    1.0689    1.0692
count = 6
WTCoeefModifx4 = 5×4000
    1.0672    1.0675    1.0678    1.0681    1.0684    1.0686    1.0689    1.0692 ⋯
    1.0672    1.0675    1.0678    1.0681    1.0684    1.0686    1.0689    1.0692
    1.0672    1.0675    1.0678    1.0681    1.0684    1.0686    1.0689    1.0692
    1.0672    1.0675    1.0678    1.0681    1.0684    1.0686    1.0689    1.0692
    1.0672    1.0675    1.0678    1.0681    1.0684    1.0686    1.0689    1.0692
```

```
WTCoeefModifx3 = plotPolyrawdata(WTCoeffs(1,:), 1:4000, 3,0,3,0.8,1.2,0.1)
```

```
rawdata = 1×4000
    1.0672    1.0675    1.0678    1.0681    1.0684    1.0686    1.0689    1.0692 ⋯
count = 2
rawdata = 2×4000
    1.0672    1.0675    1.0678    1.0681    1.0684    1.0686    1.0689    1.0692 ⋯
    1.0672    1.0675    1.0678    1.0681    1.0684    1.0686    1.0689    1.0692
count = 3
rawdata = 3×4000
    1.0672    1.0675    1.0678    1.0681    1.0684    1.0686    1.0689    1.0692 ⋯
    1.0672    1.0675    1.0678    1.0681    1.0684    1.0686    1.0689    1.0692
    1.0672    1.0675    1.0678    1.0681    1.0684    1.0686    1.0689    1.0692
count = 4
rawdata = 4×4000
    1.0672    1.0675    1.0678    1.0681    1.0684    1.0686    1.0689    1.0692 ⋯
    1.0672    1.0675    1.0678    1.0681    1.0684    1.0686    1.0689    1.0692
    1.0672    1.0675    1.0678    1.0681    1.0684    1.0686    1.0689    1.0692
    1.0672    1.0675    1.0678    1.0681    1.0684    1.0686    1.0689    1.0692
count = 5
rawdata = 5×4000
    1.0672    1.0675    1.0678    1.0681    1.0684    1.0686    1.0689    1.0692 ⋯
    1.0672    1.0675    1.0678    1.0681    1.0684    1.0686    1.0689    1.0692
    1.0672    1.0675    1.0678    1.0681    1.0684    1.0686    1.0689    1.0692
    1.0672    1.0675    1.0678    1.0681    1.0684    1.0686    1.0689    1.0692
    1.0672    1.0675    1.0678    1.0681    1.0684    1.0686    1.0689    1.0692
count = 6
WTCoeefModifx3 = 5×4000
    1.0672    1.0675    1.0678    1.0681    1.0684    1.0686    1.0689    1.0692 ⋯
    1.0672    1.0675    1.0678    1.0681    1.0684    1.0686    1.0689    1.0692
    1.0672    1.0675    1.0678    1.0681    1.0684    1.0686    1.0689    1.0692
    1.0672    1.0675    1.0678    1.0681    1.0684    1.0686    1.0689    1.0692
    1.0672    1.0675    1.0678    1.0681    1.0684    1.0686    1.0689    1.0692
```

```
WTCoeefModifx2 = plotPolyrawdata(WTCoeffs(1,:), 1:4000, 4,0,3,0.8,1.2,0.1)
```

```
rawdata = 1×4000
    1.0672    1.0675    1.0678    1.0681    1.0684    1.0687    1.0689    1.0692 ⋯
count = 2
rawdata = 2×4000
    1.0672    1.0675    1.0678    1.0681    1.0684    1.0687    1.0689    1.0692 ⋯
    1.0672    1.0675    1.0678    1.0681    1.0684    1.0686    1.0689    1.0692
count = 3
rawdata = 3×4000
    1.0672    1.0675    1.0678    1.0681    1.0684    1.0687    1.0689    1.0692 ⋯
    1.0672    1.0675    1.0678    1.0681    1.0684    1.0686    1.0689    1.0692
    1.0672    1.0675    1.0678    1.0681    1.0684    1.0686    1.0689    1.0692
count = 4
rawdata = 4×4000
    1.0672    1.0675    1.0678    1.0681    1.0684    1.0687    1.0689    1.0692 ⋯
    1.0672    1.0675    1.0678    1.0681    1.0684    1.0686    1.0689    1.0692
    1.0672    1.0675    1.0678    1.0681    1.0684    1.0686    1.0689    1.0692
    1.0672    1.0675    1.0678    1.0681    1.0684    1.0686    1.0689    1.0692
count = 5
rawdata = 5×4000
    1.0672    1.0675    1.0678    1.0681    1.0684    1.0687    1.0689    1.0692 ⋯
    1.0672    1.0675    1.0678    1.0681    1.0684    1.0686    1.0689    1.0692
    1.0672    1.0675    1.0678    1.0681    1.0684    1.0686    1.0689    1.0692
    1.0672    1.0675    1.0678    1.0681    1.0684    1.0686    1.0689    1.0692
    1.0672    1.0675    1.0678    1.0681    1.0684    1.0686    1.0689    1.0692
count = 6
WTCoeefModifx2 = 5×4000
    1.0672    1.0675    1.0678    1.0681    1.0684    1.0687    1.0689    1.0692 ⋯
    1.0672    1.0675    1.0678    1.0681    1.0684    1.0686    1.0689    1.0692
    1.0672    1.0675    1.0678    1.0681    1.0684    1.0686    1.0689    1.0692
    1.0672    1.0675    1.0678    1.0681    1.0684    1.0686    1.0689    1.0692
    1.0672    1.0675    1.0678    1.0681    1.0684    1.0686    1.0689    1.0692
```

```
WTCoeefModifx1 = plotPolyrawdata(WTCoeffs(1,:), 1:4000, 5,0,3,0.8,1.2,0.1)
```

```
rawdata = 1×4000
    1.0672    1.0674    1.0676    1.0679    1.0681    1.0683    1.0685    1.0687 ⋯
count = 2
rawdata = 2×4000
    1.0672    1.0674    1.0676    1.0679    1.0681    1.0683    1.0685    1.0687 ⋯
    1.0672    1.0675    1.0677    1.0680    1.0682    1.0685    1.0687    1.0690
count = 3
rawdata = 3×4000
    1.0672    1.0674    1.0676    1.0679    1.0681    1.0683    1.0685    1.0687 ⋯
    1.0672    1.0675    1.0677    1.0680    1.0682    1.0685    1.0687    1.0690
    1.0672    1.0675    1.0678    1.0681    1.0684    1.0686    1.0689    1.0692
count = 4
rawdata = 4×4000
    1.0672    1.0674    1.0676    1.0679    1.0681    1.0683    1.0685    1.0687 ⋯
    1.0672    1.0675    1.0677    1.0680    1.0682    1.0685    1.0687    1.0690
    1.0672    1.0675    1.0678    1.0681    1.0684    1.0686    1.0689    1.0692
    1.0673    1.0676    1.0679    1.0682    1.0685    1.0688    1.0691    1.0694
count = 5
rawdata = 5×4000
    1.0672    1.0674    1.0676    1.0679    1.0681    1.0683    1.0685    1.0687 ⋯
    1.0672    1.0675    1.0677    1.0680    1.0682    1.0685    1.0687    1.0690
    1.0672    1.0675    1.0678    1.0681    1.0684    1.0686    1.0689    1.0692
    1.0673    1.0676    1.0679    1.0682    1.0685    1.0688    1.0691    1.0694
    1.0673    1.0676    1.0680    1.0683    1.0687    1.0690    1.0693    1.0697
count = 6
WTCoeefModifx1 = 5×4000
    1.0672    1.0674    1.0676    1.0679    1.0681    1.0683    1.0685    1.0687 ⋯
    1.0672    1.0675    1.0677    1.0680    1.0682    1.0685    1.0687    1.0690
    1.0672    1.0675    1.0678    1.0681    1.0684    1.0686    1.0689    1.0692
    1.0673    1.0676    1.0679    1.0682    1.0685    1.0688    1.0691    1.0694
```

```
    1.0673    1.0676    1.0680    1.0683    1.0687    1.0690    1.0693    1.0697
```

```
WTCoeefModifx0 = plotPolyrawdata(WTCoeffs(1,:), 1:4000, 6,0,3,0.8,1.2,0.1)
```

```
rawdata = 1×4000
    0.8538    0.8541    0.8544    0.8547    0.8550    0.8553    0.8555    0.8558 ···
count = 2
rawdata = 2×4000
    0.8538    0.8541    0.8544    0.8547    0.8550    0.8553    0.8555    0.8558 ···
    0.9605    0.9608    0.9611    0.9614    0.9617    0.9619    0.9622    0.9625
count = 3
rawdata = 3×4000
    0.8538    0.8541    0.8544    0.8547    0.8550    0.8553    0.8555    0.8558 ···
    0.9605    0.9608    0.9611    0.9614    0.9617    0.9619    0.9622    0.9625
    1.0672    1.0675    1.0678    1.0681    1.0684    1.0686    1.0689    1.0692
count = 4
rawdata = 4×4000
    0.8538    0.8541    0.8544    0.8547    0.8550    0.8553    0.8555    0.8558 ···
    0.9605    0.9608    0.9611    0.9614    0.9617    0.9619    0.9622    0.9625
    1.0672    1.0675    1.0678    1.0681    1.0684    1.0686    1.0689    1.0692
    1.1739    1.1742    1.1745    1.1748    1.1751    1.1753    1.1756    1.1759
count = 5
rawdata = 5×4000
    0.8538    0.8541    0.8544    0.8547    0.8550    0.8553    0.8555    0.8558 ···
    0.9605    0.9608    0.9611    0.9614    0.9617    0.9619    0.9622    0.9625
    1.0672    1.0675    1.0678    1.0681    1.0684    1.0686    1.0689    1.0692
    1.1739    1.1742    1.1745    1.1748    1.1751    1.1753    1.1756    1.1759
    1.2806    1.2809    1.2812    1.2815    1.2818    1.2820    1.2823    1.2826
count = 6
WTCoeefModifx0 = 5×4000
    0.8538    0.8541    0.8544    0.8547    0.8550    0.8553    0.8555    0.8558 ···
    0.9605    0.9608    0.9611    0.9614    0.9617    0.9619    0.9622    0.9625
    1.0672    1.0675    1.0678    1.0681    1.0684    1.0686    1.0689    1.0692
    1.1739    1.1742    1.1745    1.1748    1.1751    1.1753    1.1756    1.1759
    1.2806    1.2809    1.2812    1.2815    1.2818    1.2820    1.2823    1.2826
```

```
ThrombinCoeffsModifx5=plotPolyrawdata(thrombinCoeffs(5,:), 1:300,1,0.5,2,0.8,1.2,0.1)
```

```
rawdata = 1×500
    1.0567    1.0476    1.0387    1.0300    1.0215    1.0132    1.0051    0.9972 ···
count = 2
rawdata = 2×500
    1.0567    1.0476    1.0387    1.0300    1.0215    1.0132    1.0051    0.9972 ···
    1.0567    1.0476    1.0387    1.0300    1.0215    1.0132    1.0051    0.9972
count = 3
rawdata = 3×500
    1.0567    1.0476    1.0387    1.0300    1.0215    1.0132    1.0051    0.9972 ···
    1.0567    1.0476    1.0387    1.0300    1.0215    1.0132    1.0051    0.9972
    1.0567    1.0476    1.0387    1.0300    1.0215    1.0132    1.0051    0.9972
count = 4
rawdata = 4×500
    1.0567    1.0476    1.0387    1.0300    1.0215    1.0132    1.0051    0.9972 ···
    1.0567    1.0476    1.0387    1.0300    1.0215    1.0132    1.0051    0.9972
    1.0567    1.0476    1.0387    1.0300    1.0215    1.0132    1.0051    0.9972
    1.0567    1.0476    1.0387    1.0300    1.0215    1.0132    1.0051    0.9972
count = 5
rawdata = 5×500
    1.0567    1.0476    1.0387    1.0300    1.0215    1.0132    1.0051    0.9972 ···
    1.0567    1.0476    1.0387    1.0300    1.0215    1.0132    1.0051    0.9972
    1.0567    1.0476    1.0387    1.0300    1.0215    1.0132    1.0051    0.9972
    1.0567    1.0476    1.0387    1.0300    1.0215    1.0132    1.0051    0.9972
    1.0567    1.0476    1.0387    1.0300    1.0215    1.0132    1.0051    0.9972
count = 6
```

```
ThrombinCoeffsModifx5 = 5×500
    1.0567    1.0476    1.0387    1.0300    1.0215    1.0132    1.0051    0.9972 ···
    1.0567    1.0476    1.0387    1.0300    1.0215    1.0132    1.0051    0.9972
    1.0567    1.0476    1.0387    1.0300    1.0215    1.0132    1.0051    0.9972
    1.0567    1.0476    1.0387    1.0300    1.0215    1.0132    1.0051    0.9972
    1.0567    1.0476    1.0387    1.0300    1.0215    1.0132    1.0051    0.9972
```

```
ThrombinCoeffsModifx4=plotPolyrawdata(thrombinCoeffs(5,:), 1:300,2,0.5,2,0.8,1.2,0.1)
```

```
rawdata = 1×500
    1.0567    1.0476    1.0387    1.0300    1.0215    1.0132    1.0051    0.9972 ···
count = 2
rawdata = 2×500
    1.0567    1.0476    1.0387    1.0300    1.0215    1.0132    1.0051    0.9972 ···
    1.0567    1.0476    1.0387    1.0300    1.0215    1.0132    1.0051    0.9972
count = 3
rawdata = 3×500
    1.0567    1.0476    1.0387    1.0300    1.0215    1.0132    1.0051    0.9972 ···
    1.0567    1.0476    1.0387    1.0300    1.0215    1.0132    1.0051    0.9972
    1.0567    1.0476    1.0387    1.0300    1.0215    1.0132    1.0051    0.9972
count = 4
rawdata = 4×500
    1.0567    1.0476    1.0387    1.0300    1.0215    1.0132    1.0051    0.9972 ···
    1.0567    1.0476    1.0387    1.0300    1.0215    1.0132    1.0051    0.9972
    1.0567    1.0476    1.0387    1.0300    1.0215    1.0132    1.0051    0.9972
    1.0567    1.0476    1.0387    1.0300    1.0215    1.0132    1.0051    0.9972
count = 5
rawdata = 5×500
    1.0567    1.0476    1.0387    1.0300    1.0215    1.0132    1.0051    0.9972 ···
    1.0567    1.0476    1.0387    1.0300    1.0215    1.0132    1.0051    0.9972
    1.0567    1.0476    1.0387    1.0300    1.0215    1.0132    1.0051    0.9972
    1.0567    1.0476    1.0387    1.0300    1.0215    1.0132    1.0051    0.9972
    1.0567    1.0476    1.0387    1.0300    1.0215    1.0132    1.0051    0.9972
count = 6
ThrombinCoeffsModifx4 = 5×500
    1.0567    1.0476    1.0387    1.0300    1.0215    1.0132    1.0051    0.9972 ···
    1.0567    1.0476    1.0387    1.0300    1.0215    1.0132    1.0051    0.9972
    1.0567    1.0476    1.0387    1.0300    1.0215    1.0132    1.0051    0.9972
    1.0567    1.0476    1.0387    1.0300    1.0215    1.0132    1.0051    0.9972
    1.0567    1.0476    1.0387    1.0300    1.0215    1.0132    1.0051    0.9972
```

```
ThrombinCoeffsModifx3=plotPolyrawdata(thrombinCoeffs(5,:), 1:300,3,0.5,2,0.8,1.2,0.1)
```

```
rawdata = 1×500
    1.0567    1.0476    1.0387    1.0300    1.0215    1.0132    1.0052    0.9973 ···
count = 2
rawdata = 2×500
    1.0567    1.0476    1.0387    1.0300    1.0215    1.0132    1.0052    0.9973 ···
    1.0567    1.0476    1.0387    1.0300    1.0215    1.0132    1.0052    0.9973
count = 3
rawdata = 3×500
    1.0567    1.0476    1.0387    1.0300    1.0215    1.0132    1.0052    0.9973 ···
    1.0567    1.0476    1.0387    1.0300    1.0215    1.0132    1.0052    0.9973
    1.0567    1.0476    1.0387    1.0300    1.0215    1.0132    1.0051    0.9972
count = 4
rawdata = 4×500
    1.0567    1.0476    1.0387    1.0300    1.0215    1.0132    1.0052    0.9973 ···
    1.0567    1.0476    1.0387    1.0300    1.0215    1.0132    1.0052    0.9973
    1.0567    1.0476    1.0387    1.0300    1.0215    1.0132    1.0051    0.9972
    1.0567    1.0476    1.0387    1.0300    1.0215    1.0132    1.0051    0.9972
count = 5
rawdata = 5×500
    1.0567    1.0476    1.0387    1.0300    1.0215    1.0132    1.0052    0.9973 ···
    1.0567    1.0476    1.0387    1.0300    1.0215    1.0132    1.0052    0.9973
```

```
    1.0567    1.0476    1.0387    1.0300    1.0215    1.0132    1.0051    0.9972
    1.0567    1.0476    1.0387    1.0300    1.0215    1.0132    1.0051    0.9972
    1.0567    1.0476    1.0387    1.0300    1.0215    1.0132    1.0051    0.9972
count = 6
ThrombinCoeffsModifx3 = 5×500
    1.0567    1.0476    1.0387    1.0300    1.0215    1.0132    1.0052    0.9973 ⋯
    1.0567    1.0476    1.0387    1.0300    1.0215    1.0132    1.0052    0.9973
    1.0567    1.0476    1.0387    1.0300    1.0215    1.0132    1.0051    0.9972
    1.0567    1.0476    1.0387    1.0300    1.0215    1.0132    1.0051    0.9972
    1.0567    1.0476    1.0387    1.0300    1.0215    1.0132    1.0051    0.9972
```

```
ThrombinCoeffsModifx2=plotPolyrawdata(thrombinCoeffs(5,:), 1:300,4,0.5,2,0.8,1.2,0.1)
```

```
rawdata = 1×500
    1.0567    1.0475    1.0385    1.0297    1.0210    1.0124    1.0041    0.9958 ⋯
count = 2
rawdata = 2×500
    1.0567    1.0475    1.0385    1.0297    1.0210    1.0124    1.0041    0.9958 ⋯
    1.0567    1.0476    1.0386    1.0298    1.0212    1.0128    1.0046    0.9965
count = 3
rawdata = 3×500
    1.0567    1.0475    1.0385    1.0297    1.0210    1.0124    1.0041    0.9958 ⋯
    1.0567    1.0476    1.0386    1.0298    1.0212    1.0128    1.0046    0.9965
    1.0567    1.0476    1.0387    1.0300    1.0215    1.0132    1.0051    0.9972
count = 4
rawdata = 4×500
    1.0567    1.0475    1.0385    1.0297    1.0210    1.0124    1.0041    0.9958 ⋯
    1.0567    1.0476    1.0386    1.0298    1.0212    1.0128    1.0046    0.9965
    1.0567    1.0476    1.0387    1.0300    1.0215    1.0132    1.0051    0.9972
    1.0567    1.0477    1.0388    1.0302    1.0218    1.0136    1.0057    0.9979
count = 5
rawdata = 5×500
    1.0567    1.0475    1.0385    1.0297    1.0210    1.0124    1.0041    0.9958 ⋯
    1.0567    1.0476    1.0386    1.0298    1.0212    1.0128    1.0046    0.9965
    1.0567    1.0476    1.0387    1.0300    1.0215    1.0132    1.0051    0.9972
    1.0567    1.0477    1.0388    1.0302    1.0218    1.0136    1.0057    0.9979
    1.0568    1.0477    1.0389    1.0304    1.0221    1.0140    1.0062    0.9986
count = 6
ThrombinCoeffsModifx2 = 5×500
    1.0567    1.0475    1.0385    1.0297    1.0210    1.0124    1.0041    0.9958 ⋯
    1.0567    1.0476    1.0386    1.0298    1.0212    1.0128    1.0046    0.9965
    1.0567    1.0476    1.0387    1.0300    1.0215    1.0132    1.0051    0.9972
    1.0567    1.0477    1.0388    1.0302    1.0218    1.0136    1.0057    0.9979
    1.0568    1.0477    1.0389    1.0304    1.0221    1.0140    1.0062    0.9986
```

```
ThrombinCoeffsModifx1=plotPolyrawdata(thrombinCoeffs(5,:), 1:300,5,0.5,2,0.8,1.2,0.1)
```

```
rawdata = 1×500
    1.0586    1.0514    1.0444    1.0376    1.0310    1.0246    1.0184    1.0124 ⋯
count = 2
rawdata = 2×500
    1.0586    1.0514    1.0444    1.0376    1.0310    1.0246    1.0184    1.0124 ⋯
    1.0577    1.0495    1.0415    1.0338    1.0262    1.0189    1.0117    1.0048
count = 3
rawdata = 3×500
    1.0586    1.0514    1.0444    1.0376    1.0310    1.0246    1.0184    1.0124 ⋯
    1.0577    1.0495    1.0415    1.0338    1.0262    1.0189    1.0117    1.0048
    1.0567    1.0476    1.0387    1.0300    1.0215    1.0132    1.0051    0.9972
count = 4
rawdata = 4×500
    1.0586    1.0514    1.0444    1.0376    1.0310    1.0246    1.0184    1.0124 ⋯
    1.0577    1.0495    1.0415    1.0338    1.0262    1.0189    1.0117    1.0048
    1.0567    1.0476    1.0387    1.0300    1.0215    1.0132    1.0051    0.9972
    1.0558    1.0457    1.0359    1.0262    1.0168    1.0076    0.9985    0.9897
```

```
count = 5
rawdata = 5×500
    1.0586    1.0514    1.0444    1.0376    1.0310    1.0246    1.0184    1.0124 ···
    1.0577    1.0495    1.0415    1.0338    1.0262    1.0189    1.0117    1.0048
    1.0567    1.0476    1.0387    1.0300    1.0215    1.0132    1.0051    0.9972
    1.0558    1.0457    1.0359    1.0262    1.0168    1.0076    0.9985    0.9897
    1.0548    1.0438    1.0330    1.0224    1.0121    1.0019    0.9919    0.9821
count = 6
ThrombinCoeffsModifx1 = 5×500
    1.0586    1.0514    1.0444    1.0376    1.0310    1.0246    1.0184    1.0124 ···
    1.0577    1.0495    1.0415    1.0338    1.0262    1.0189    1.0117    1.0048
    1.0567    1.0476    1.0387    1.0300    1.0215    1.0132    1.0051    0.9972
    1.0558    1.0457    1.0359    1.0262    1.0168    1.0076    0.9985    0.9897
    1.0548    1.0438    1.0330    1.0224    1.0121    1.0019    0.9919    0.9821
```

```
ThrombinCoeffsModifx0=plotPolyrawdata(thrombinCoeffs(5,:), 1:300,6,0.5,2,0.8,1.2,0.1)
```

```
rawdata = 1×500
    0.8435    0.8344    0.8255    0.8168    0.8083    0.8000    0.7919    0.7840 ···
count = 2
rawdata = 2×500
    0.8435    0.8344    0.8255    0.8168    0.8083    0.8000    0.7919    0.7840 ···
    0.9501    0.9410    0.9321    0.9234    0.9149    0.9066    0.8985    0.8906
count = 3
rawdata = 3×500
    0.8435    0.8344    0.8255    0.8168    0.8083    0.8000    0.7919    0.7840 ···
    0.9501    0.9410    0.9321    0.9234    0.9149    0.9066    0.8985    0.8906
    1.0567    1.0476    1.0387    1.0300    1.0215    1.0132    1.0051    0.9972
count = 4
rawdata = 4×500
    0.8435    0.8344    0.8255    0.8168    0.8083    0.8000    0.7919    0.7840 ···
    0.9501    0.9410    0.9321    0.9234    0.9149    0.9066    0.8985    0.8906
    1.0567    1.0476    1.0387    1.0300    1.0215    1.0132    1.0051    0.9972
    1.1633    1.1542    1.1453    1.1366    1.1281    1.1198    1.1117    1.1039
count = 5
rawdata = 5×500
    0.8435    0.8344    0.8255    0.8168    0.8083    0.8000    0.7919    0.7840 ···
    0.9501    0.9410    0.9321    0.9234    0.9149    0.9066    0.8985    0.8906
    1.0567    1.0476    1.0387    1.0300    1.0215    1.0132    1.0051    0.9972
    1.1633    1.1542    1.1453    1.1366    1.1281    1.1198    1.1117    1.1039
    1.2699    1.2608    1.2519    1.2432    1.2347    1.2264    1.2183    1.2105
count = 6
ThrombinCoeffsModifx0 = 5×500
    0.8435    0.8344    0.8255    0.8168    0.8083    0.8000    0.7919    0.7840 ···
    0.9501    0.9410    0.9321    0.9234    0.9149    0.9066    0.8985    0.8906
    1.0567    1.0476    1.0387    1.0300    1.0215    1.0132    1.0051    0.9972
    1.1633    1.1542    1.1453    1.1366    1.1281    1.1198    1.1117    1.1039
    1.2699    1.2608    1.2519    1.2432    1.2347    1.2264    1.2183    1.2105
```

```
WT_GSK_CoeffsModifx5=plotPolyrawdata(WT_GSK_Coeffs(3,:), 1:500,1,0,2,0.8,1.2,0.1)
```

```
rawdata = 1×500
    0.9924    0.9910    0.9895    0.9881    0.9867    0.9854    0.9840    0.9827 ···
count = 2
rawdata = 2×500
    0.9924    0.9910    0.9895    0.9881    0.9867    0.9854    0.9840    0.9827 ···
    0.9924    0.9910    0.9895    0.9881    0.9867    0.9854    0.9840    0.9827
count = 3
rawdata = 3×500
    0.9924    0.9910    0.9895    0.9881    0.9867    0.9854    0.9840    0.9827 ···
    0.9924    0.9910    0.9895    0.9881    0.9867    0.9854    0.9840    0.9827
    0.9924    0.9910    0.9895    0.9881    0.9867    0.9854    0.9840    0.9827
count = 4
```

```
rawdata = 4×500
    0.9924    0.9910    0.9895    0.9881    0.9867    0.9854    0.9840    0.9827 ···
    0.9924    0.9910    0.9895    0.9881    0.9867    0.9854    0.9840    0.9827
    0.9924    0.9910    0.9895    0.9881    0.9867    0.9854    0.9840    0.9827
    0.9924    0.9910    0.9895    0.9881    0.9867    0.9854    0.9840    0.9827
count = 5
rawdata = 5×500
    0.9924    0.9910    0.9895    0.9881    0.9867    0.9854    0.9840    0.9827 ···
    0.9924    0.9910    0.9895    0.9881    0.9867    0.9854    0.9840    0.9827
    0.9924    0.9910    0.9895    0.9881    0.9867    0.9854    0.9840    0.9827
    0.9924    0.9910    0.9895    0.9881    0.9867    0.9854    0.9840    0.9827
    0.9924    0.9910    0.9895    0.9881    0.9867    0.9854    0.9840    0.9827
count = 6
WT_GSK_CoeffsModifx5 = 5×500
    0.9924    0.9910    0.9895    0.9881    0.9867    0.9854    0.9840    0.9827 ···
    0.9924    0.9910    0.9895    0.9881    0.9867    0.9854    0.9840    0.9827
    0.9924    0.9910    0.9895    0.9881    0.9867    0.9854    0.9840    0.9827
    0.9924    0.9910    0.9895    0.9881    0.9867    0.9854    0.9840    0.9827
    0.9924    0.9910    0.9895    0.9881    0.9867    0.9854    0.9840    0.9827
```

```
WT_GSK_CoeffsModifx4=plotPolyrawdata(WT_GSK_Coeffs(3,:), 1:500,2,0,2,0.8,1.2,0.1)
```

```
rawdata = 1×500
    0.9924    0.9910    0.9895    0.9881    0.9867    0.9854    0.9840    0.9827 ···
count = 2
rawdata = 2×500
    0.9924    0.9910    0.9895    0.9881    0.9867    0.9854    0.9840    0.9827 ···
    0.9924    0.9910    0.9895    0.9881    0.9867    0.9854    0.9840    0.9827
count = 3
rawdata = 3×500
    0.9924    0.9910    0.9895    0.9881    0.9867    0.9854    0.9840    0.9827 ···
    0.9924    0.9910    0.9895    0.9881    0.9867    0.9854    0.9840    0.9827
    0.9924    0.9910    0.9895    0.9881    0.9867    0.9854    0.9840    0.9827
count = 4
rawdata = 4×500
    0.9924    0.9910    0.9895    0.9881    0.9867    0.9854    0.9840    0.9827 ···
    0.9924    0.9910    0.9895    0.9881    0.9867    0.9854    0.9840    0.9827
    0.9924    0.9910    0.9895    0.9881    0.9867    0.9854    0.9840    0.9827
    0.9924    0.9910    0.9895    0.9881    0.9867    0.9854    0.9840    0.9827
count = 5
rawdata = 5×500
    0.9924    0.9910    0.9895    0.9881    0.9867    0.9854    0.9840    0.9827 ···
    0.9924    0.9910    0.9895    0.9881    0.9867    0.9854    0.9840    0.9827
    0.9924    0.9910    0.9895    0.9881    0.9867    0.9854    0.9840    0.9827
    0.9924    0.9910    0.9895    0.9881    0.9867    0.9854    0.9840    0.9827
    0.9924    0.9910    0.9895    0.9881    0.9867    0.9854    0.9840    0.9827
count = 6
WT_GSK_CoeffsModifx4 = 5×500
    0.9924    0.9910    0.9895    0.9881    0.9867    0.9854    0.9840    0.9827 ···
    0.9924    0.9910    0.9895    0.9881    0.9867    0.9854    0.9840    0.9827
    0.9924    0.9910    0.9895    0.9881    0.9867    0.9854    0.9840    0.9827
    0.9924    0.9910    0.9895    0.9881    0.9867    0.9854    0.9840    0.9827
    0.9924    0.9910    0.9895    0.9881    0.9867    0.9854    0.9840    0.9827
```

```
WT_GSK_CoeffsModifx3=plotPolyrawdata(WT_GSK_Coeffs(3,:), 1:500,3,0,2,0.8,1.2,0.1)
```

```
rawdata = 1×500
    0.9924    0.9910    0.9895    0.9881    0.9867    0.9854    0.9840    0.9827 ···
count = 2
rawdata = 2×500
    0.9924    0.9910    0.9895    0.9881    0.9867    0.9854    0.9840    0.9827 ···
    0.9924    0.9910    0.9895    0.9881    0.9867    0.9854    0.9840    0.9827
count = 3
rawdata = 3×500
```

```
    0.9924    0.9910    0.9895    0.9881    0.9867    0.9854    0.9840    0.9827 ···
    0.9924    0.9910    0.9895    0.9881    0.9867    0.9854    0.9840    0.9827
    0.9924    0.9910    0.9895    0.9881    0.9867    0.9854    0.9840    0.9827
count = 4
rawdata = 4×500
    0.9924    0.9910    0.9895    0.9881    0.9867    0.9854    0.9840    0.9827 ···
    0.9924    0.9910    0.9895    0.9881    0.9867    0.9854    0.9840    0.9827
    0.9924    0.9910    0.9895    0.9881    0.9867    0.9854    0.9840    0.9827
    0.9924    0.9910    0.9895    0.9881    0.9867    0.9854    0.9840    0.9827
count = 5
rawdata = 5×500
    0.9924    0.9910    0.9895    0.9881    0.9867    0.9854    0.9840    0.9827 ···
    0.9924    0.9910    0.9895    0.9881    0.9867    0.9854    0.9840    0.9827
    0.9924    0.9910    0.9895    0.9881    0.9867    0.9854    0.9840    0.9827
    0.9924    0.9910    0.9895    0.9881    0.9867    0.9854    0.9840    0.9827
    0.9924    0.9910    0.9895    0.9881    0.9867    0.9854    0.9840    0.9827
count = 6
WT_GSK_CoeffsModifx3 = 5×500
    0.9924    0.9910    0.9895    0.9881    0.9867    0.9854    0.9840    0.9827 ···
    0.9924    0.9910    0.9895    0.9881    0.9867    0.9854    0.9840    0.9827
    0.9924    0.9910    0.9895    0.9881    0.9867    0.9854    0.9840    0.9827
    0.9924    0.9910    0.9895    0.9881    0.9867    0.9854    0.9840    0.9827
    0.9924    0.9910    0.9895    0.9881    0.9867    0.9854    0.9840    0.9827
```

```
WT_GSK_CoeffsModifx2=plotPolyrawdata(WT_GSK_Coeffs(3,:), 1:500,4,0,2,0.8,1.2,0.1)
```

```
rawdata = 1×500
    0.9924    0.9910    0.9895    0.9881    0.9867    0.9853    0.9839    0.9825 ···
count = 2
rawdata = 2×500
    0.9924    0.9910    0.9895    0.9881    0.9867    0.9853    0.9839    0.9825 ···
    0.9924    0.9910    0.9895    0.9881    0.9867    0.9853    0.9840    0.9826
count = 3
rawdata = 3×500
    0.9924    0.9910    0.9895    0.9881    0.9867    0.9853    0.9839    0.9825 ···
    0.9924    0.9910    0.9895    0.9881    0.9867    0.9853    0.9840    0.9826
    0.9924    0.9910    0.9895    0.9881    0.9867    0.9854    0.9840    0.9827
count = 4
rawdata = 4×500
    0.9924    0.9910    0.9895    0.9881    0.9867    0.9853    0.9839    0.9825 ···
    0.9924    0.9910    0.9895    0.9881    0.9867    0.9853    0.9840    0.9826
    0.9924    0.9910    0.9895    0.9881    0.9867    0.9854    0.9840    0.9827
    0.9924    0.9910    0.9895    0.9881    0.9868    0.9854    0.9841    0.9828
count = 5
rawdata = 5×500
    0.9924    0.9910    0.9895    0.9881    0.9867    0.9853    0.9839    0.9825 ···
    0.9924    0.9910    0.9895    0.9881    0.9867    0.9853    0.9840    0.9826
    0.9924    0.9910    0.9895    0.9881    0.9867    0.9854    0.9840    0.9827
    0.9924    0.9910    0.9895    0.9881    0.9868    0.9854    0.9841    0.9828
    0.9924    0.9910    0.9895    0.9882    0.9868    0.9855    0.9842    0.9829
count = 6
WT_GSK_CoeffsModifx2 = 5×500
    0.9924    0.9910    0.9895    0.9881    0.9867    0.9853    0.9839    0.9825 ···
    0.9924    0.9910    0.9895    0.9881    0.9867    0.9853    0.9840    0.9826
    0.9924    0.9910    0.9895    0.9881    0.9867    0.9854    0.9840    0.9827
    0.9924    0.9910    0.9895    0.9881    0.9868    0.9854    0.9841    0.9828
    0.9924    0.9910    0.9895    0.9882    0.9868    0.9855    0.9842    0.9829
```

```
WT_GSK_CoeffsModifx1=plotPolyrawdata(WT_GSK_Coeffs(3,:), 1:500,5,0,2,0.8,1.2,0.1)
```

```
rawdata = 1×500
    0.9927    0.9916    0.9904    0.9893    0.9882    0.9872    0.9861    0.9851 ···
count = 2
rawdata = 2×500
```

```
    0.9927    0.9916    0.9904    0.9893    0.9882    0.9872    0.9861    0.9851 ···
    0.9926    0.9913    0.9900    0.9887    0.9875    0.9863    0.9851    0.9839
count = 3
rawdata = 3×500
    0.9927    0.9916    0.9904    0.9893    0.9882    0.9872    0.9861    0.9851 ···
    0.9926    0.9913    0.9900    0.9887    0.9875    0.9863    0.9851    0.9839
    0.9924    0.9910    0.9895    0.9881    0.9867    0.9854    0.9840    0.9827
count = 4
rawdata = 4×500
    0.9927    0.9916    0.9904    0.9893    0.9882    0.9872    0.9861    0.9851 ···
    0.9926    0.9913    0.9900    0.9887    0.9875    0.9863    0.9851    0.9839
    0.9924    0.9910    0.9895    0.9881    0.9867    0.9854    0.9840    0.9827
    0.9923    0.9907    0.9891    0.9875    0.9860    0.9845    0.9830    0.9815
count = 5
rawdata = 5×500
    0.9927    0.9916    0.9904    0.9893    0.9882    0.9872    0.9861    0.9851 ···
    0.9926    0.9913    0.9900    0.9887    0.9875    0.9863    0.9851    0.9839
    0.9924    0.9910    0.9895    0.9881    0.9867    0.9854    0.9840    0.9827
    0.9923    0.9907    0.9891    0.9875    0.9860    0.9845    0.9830    0.9815
    0.9921    0.9904    0.9886    0.9869    0.9852    0.9836    0.9819    0.9803
count = 6
WT_GSK_CoeffsModifx1 = 5×500
    0.9927    0.9916    0.9904    0.9893    0.9882    0.9872    0.9861    0.9851 ···
    0.9926    0.9913    0.9900    0.9887    0.9875    0.9863    0.9851    0.9839
    0.9924    0.9910    0.9895    0.9881    0.9867    0.9854    0.9840    0.9827
    0.9923    0.9907    0.9891    0.9875    0.9860    0.9845    0.9830    0.9815
    0.9921    0.9904    0.9886    0.9869    0.9852    0.9836    0.9819    0.9803
```

```
WT_GSK_CoeffsModifx0=plotPolyrawdata(WT_GSK_Coeffs(3,:), 1:500,6,0,2,0.8,1.2,0.1)
```

```
rawdata = 1×500
    0.7936    0.7922    0.7907    0.7893    0.7879    0.7866    0.7852    0.7839 ···
count = 2
rawdata = 2×500
    0.7936    0.7922    0.7907    0.7893    0.7879    0.7866    0.7852    0.7839 ···
    0.8930    0.8916    0.8901    0.8887    0.8873    0.8860    0.8846    0.8833
count = 3
rawdata = 3×500
    0.7936    0.7922    0.7907    0.7893    0.7879    0.7866    0.7852    0.7839 ···
    0.8930    0.8916    0.8901    0.8887    0.8873    0.8860    0.8846    0.8833
    0.9924    0.9910    0.9895    0.9881    0.9867    0.9854    0.9840    0.9827
count = 4
rawdata = 4×500
    0.7936    0.7922    0.7907    0.7893    0.7879    0.7866    0.7852    0.7839 ···
    0.8930    0.8916    0.8901    0.8887    0.8873    0.8860    0.8846    0.8833
    0.9924    0.9910    0.9895    0.9881    0.9867    0.9854    0.9840    0.9827
    1.0918    1.0904    1.0889    1.0875    1.0861    1.0848    1.0834    1.0821
count = 5
rawdata = 5×500
    0.7936    0.7922    0.7907    0.7893    0.7879    0.7866    0.7852    0.7839 ···
    0.8930    0.8916    0.8901    0.8887    0.8873    0.8860    0.8846    0.8833
    0.9924    0.9910    0.9895    0.9881    0.9867    0.9854    0.9840    0.9827
    1.0918    1.0904    1.0889    1.0875    1.0861    1.0848    1.0834    1.0821
    1.1912    1.1897    1.1883    1.1869    1.1855    1.1841    1.1828    1.1815
count = 6
WT_GSK_CoeffsModifx0 = 5×500
    0.7936    0.7922    0.7907    0.7893    0.7879    0.7866    0.7852    0.7839 ···
    0.8930    0.8916    0.8901    0.8887    0.8873    0.8860    0.8846    0.8833
    0.9924    0.9910    0.9895    0.9881    0.9867    0.9854    0.9840    0.9827
    1.0918    1.0904    1.0889    1.0875    1.0861    1.0848    1.0834    1.0821
    1.1912    1.1897    1.1883    1.1869    1.1855    1.1841    1.1828    1.1815
```

```
plotPoly(WTCoeffs(1,:), 1:4000,1,0,3,0.8,1.2,0.2)
```

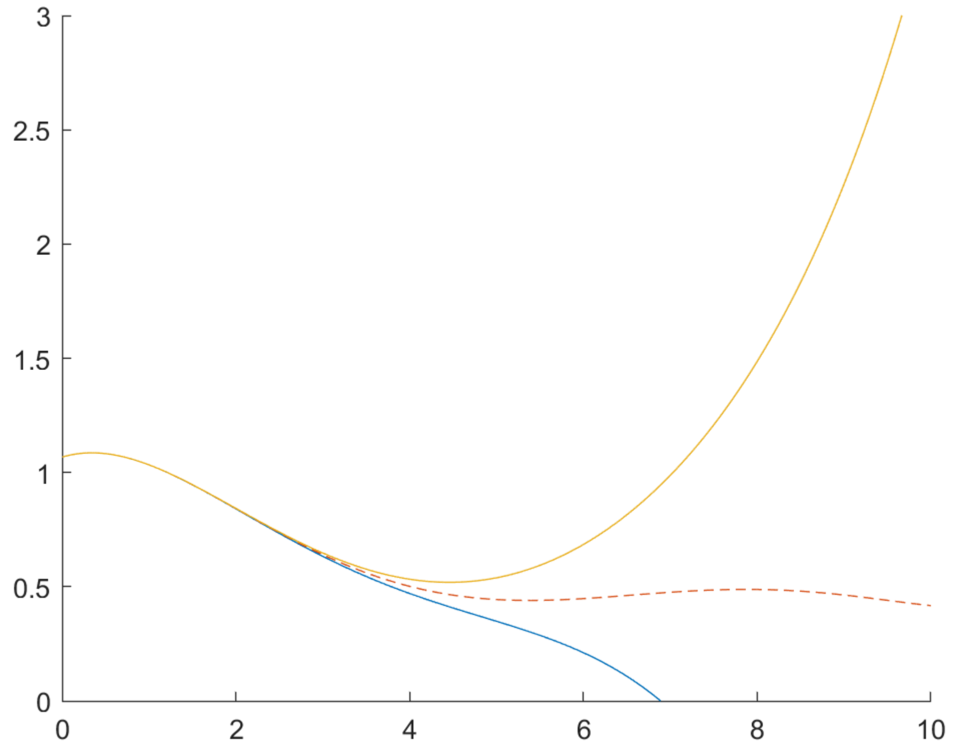


```
plotPoly(WTCoeffs(1,:), 1:4000,2,0,3,0.8,1.2,0.2)
```

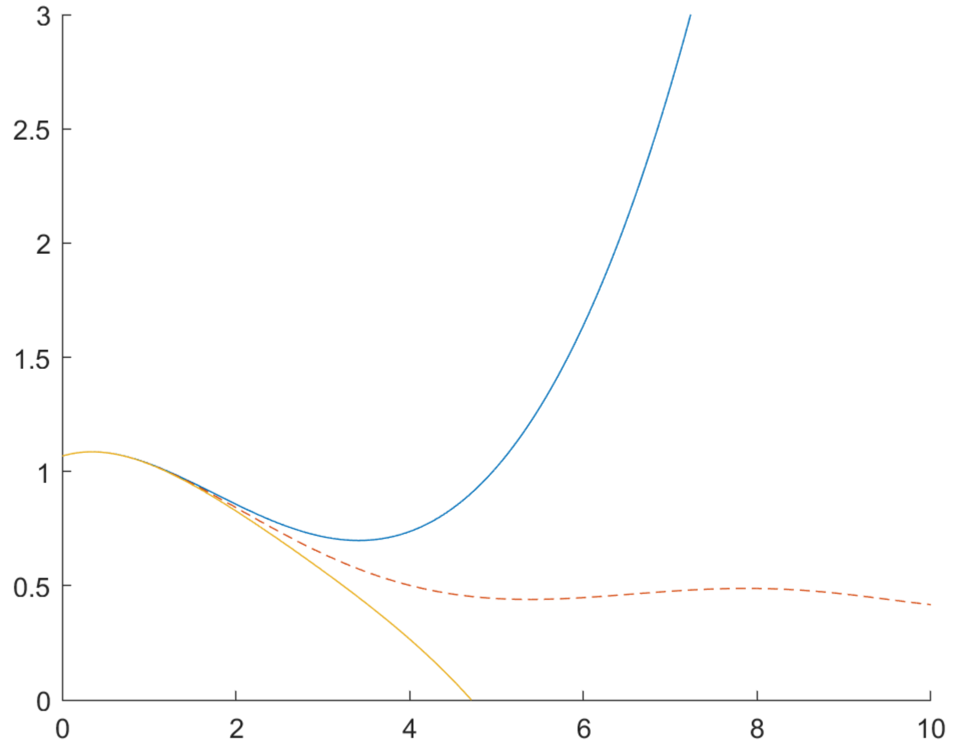

```
plotPoly(WTCoeffs(1,:), 1:4000,3,0,3,0.8,1.2,0.2)
```

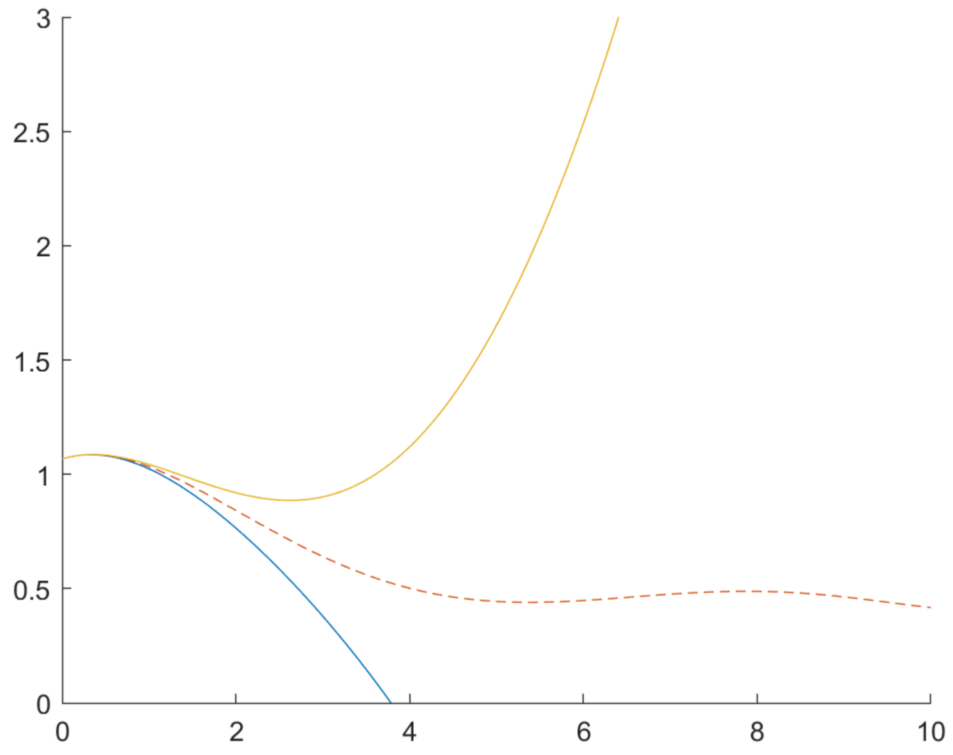


```
plotPoly(WTCoeffs(1,:), 1:4000,4,0,3,0.8,1.2,0.2)
```

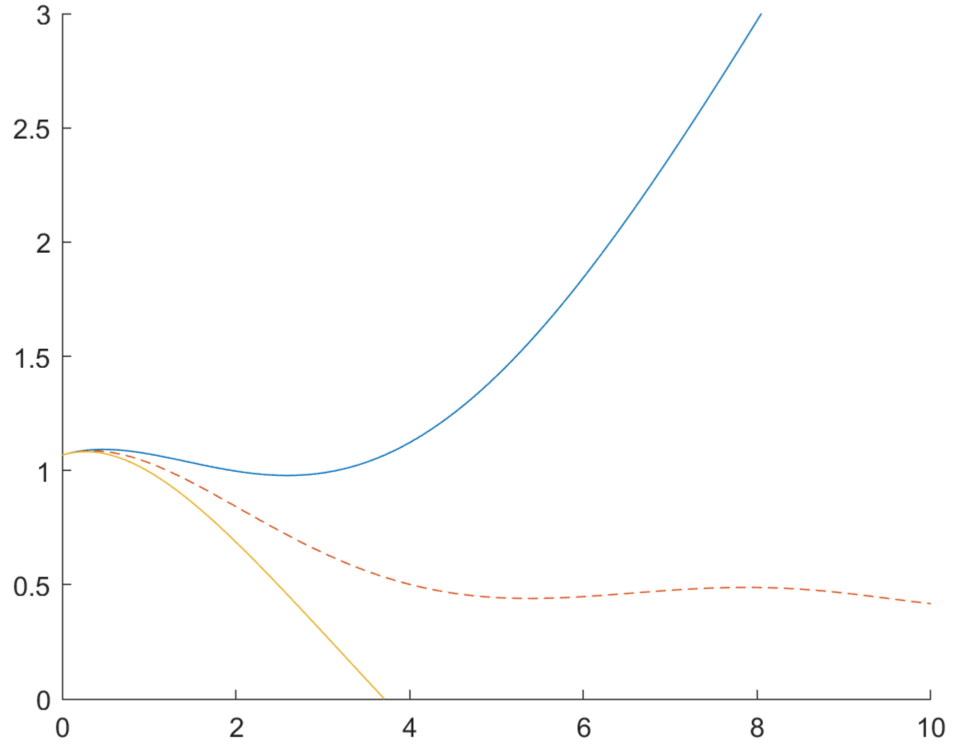


```
plotPoly(WTCoeffs(1,:), 1:4000,5,0,3,0.8,1.2,0.2)
```

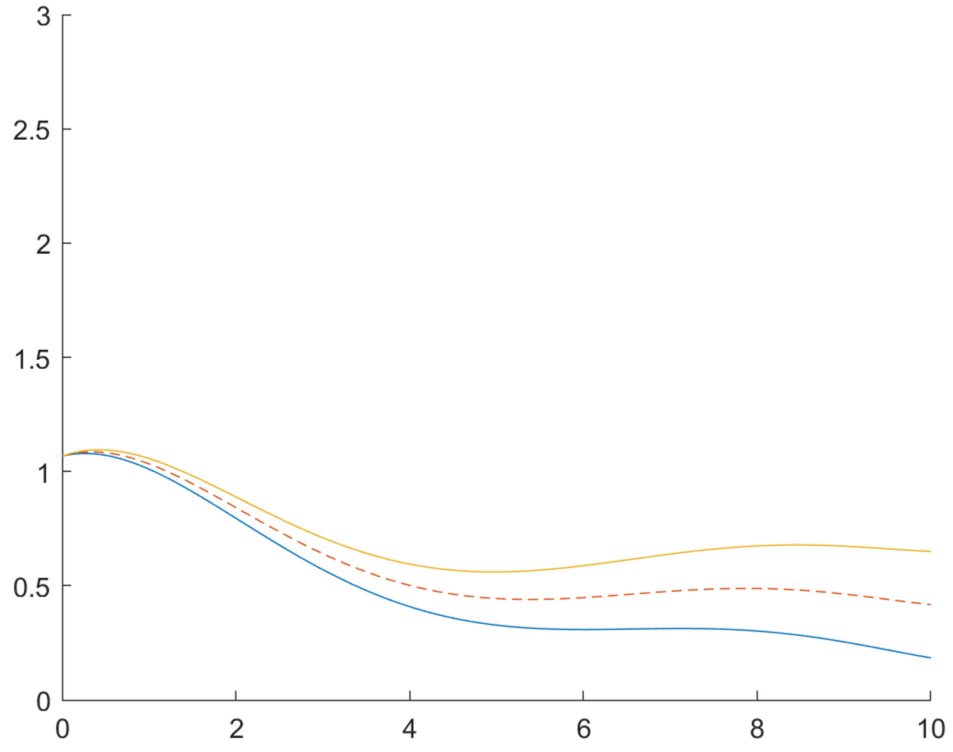

```
plotPoly(WTCoeffs(1,:), 1:4000,6,0,3,0.8,1.2,0.2)
```

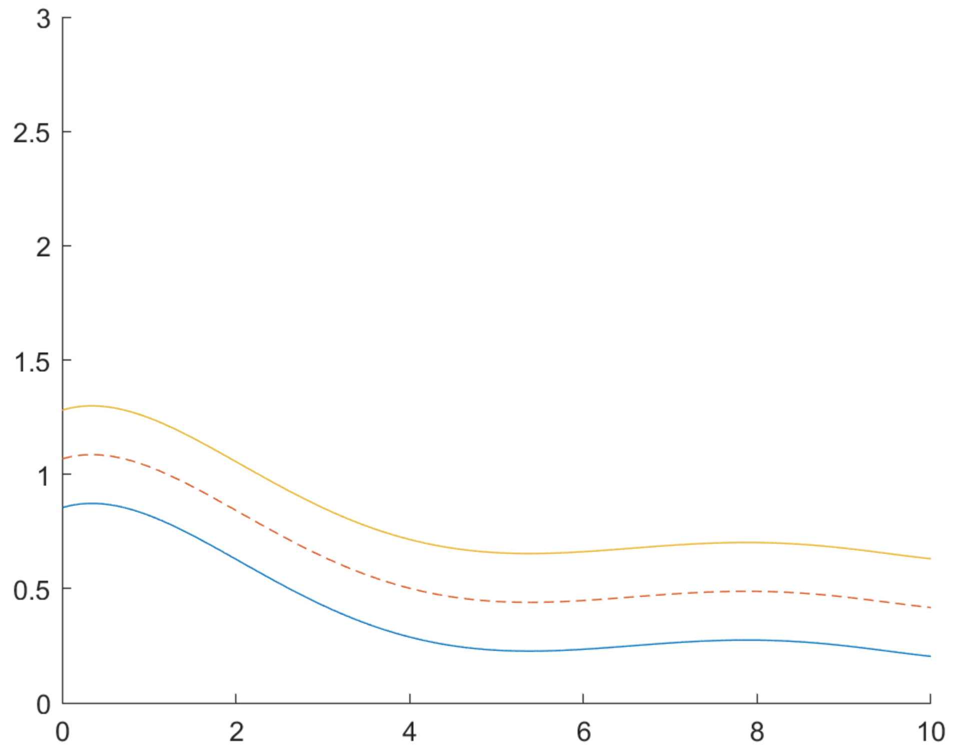


```
% Thrombin coefficient manipulation
plotPoly(thrombinCoeffs(5,:), 1:300,1,0,2,0.9,1.1,0.02)
```

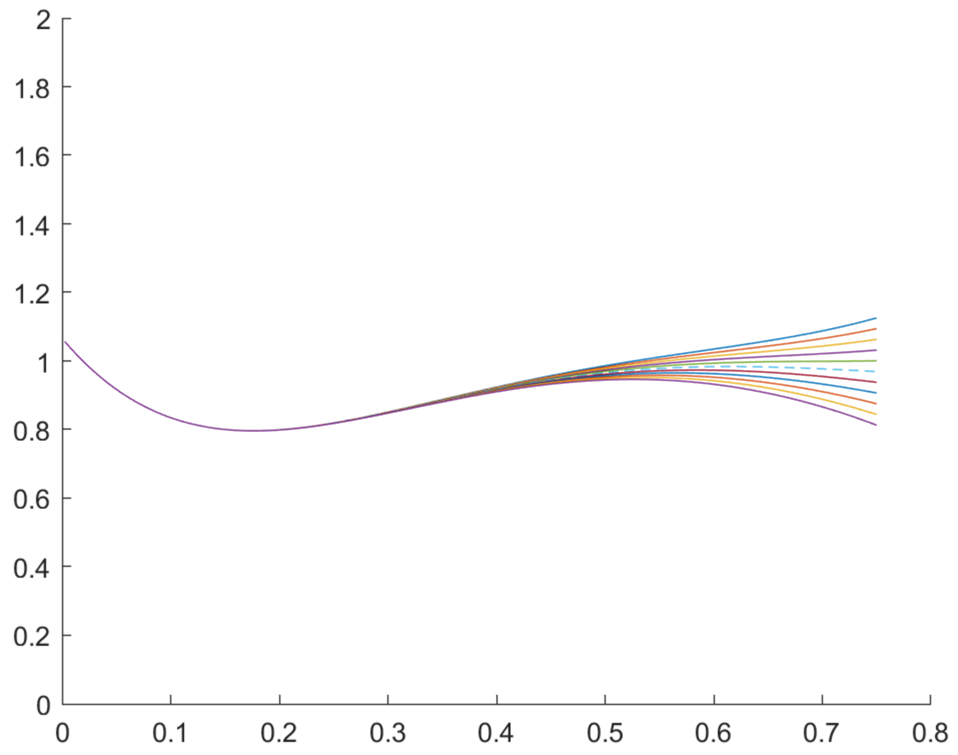


```
plotPoly(thrombinCoeffs(5,:), 1:300,2,0,2,0.9,1.1,0.02)
```

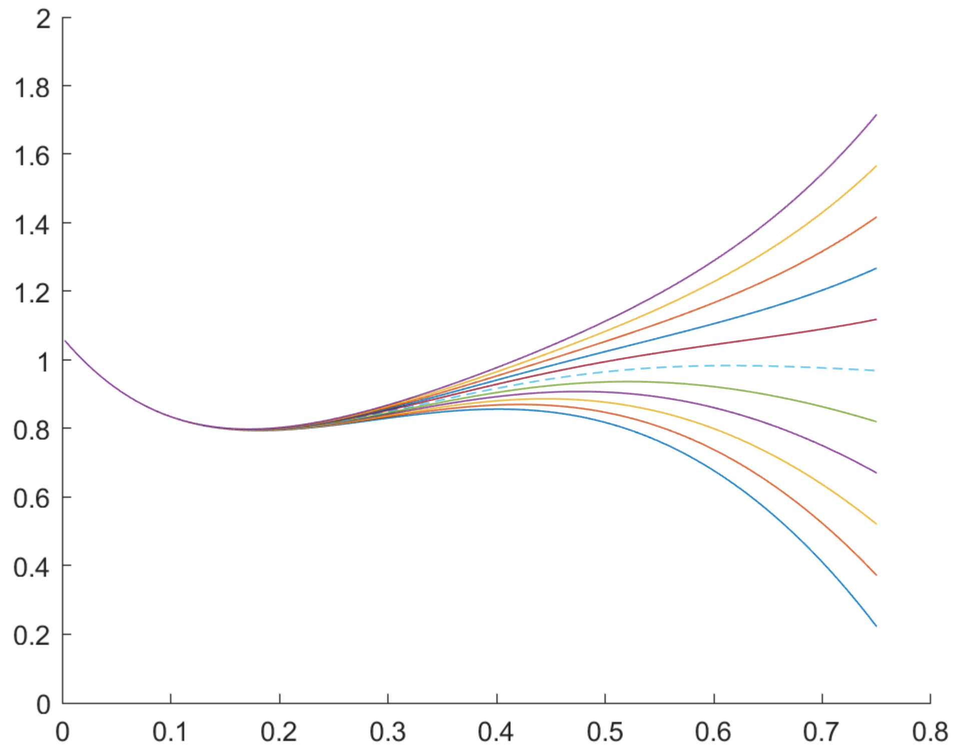

```
plotPoly(thrombinCoeffs(5,:), 1:300,3,0,2,0.9,1.1,0.02)
```

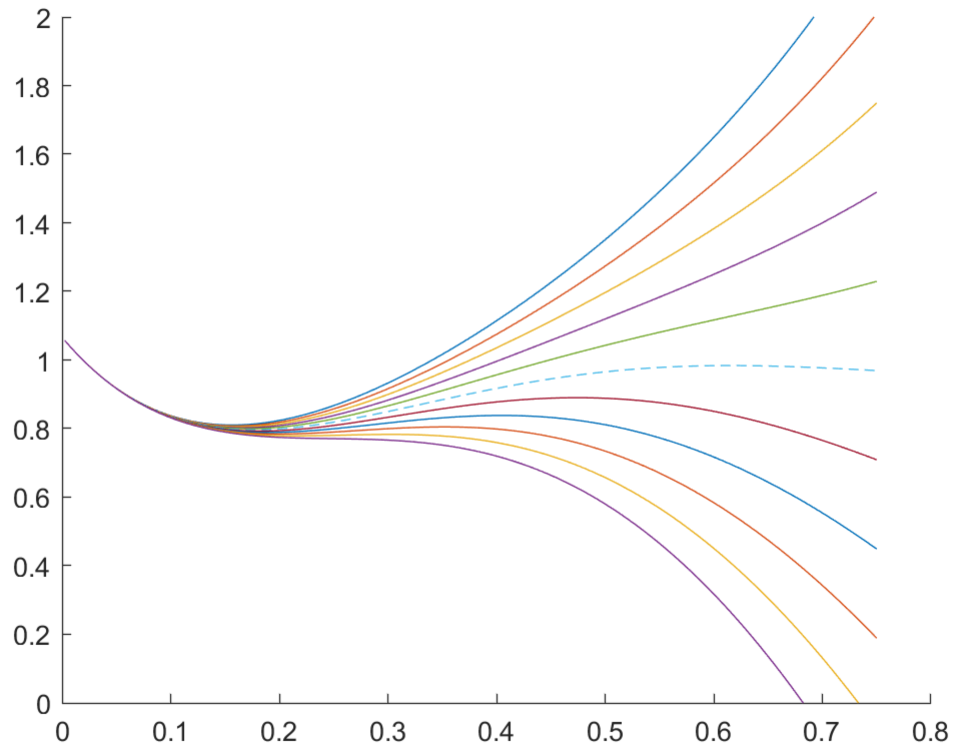


```
plotPoly(thrombinCoeffs(5,:), 1:300,4,0,2,0.9,1.1,0.02)
```

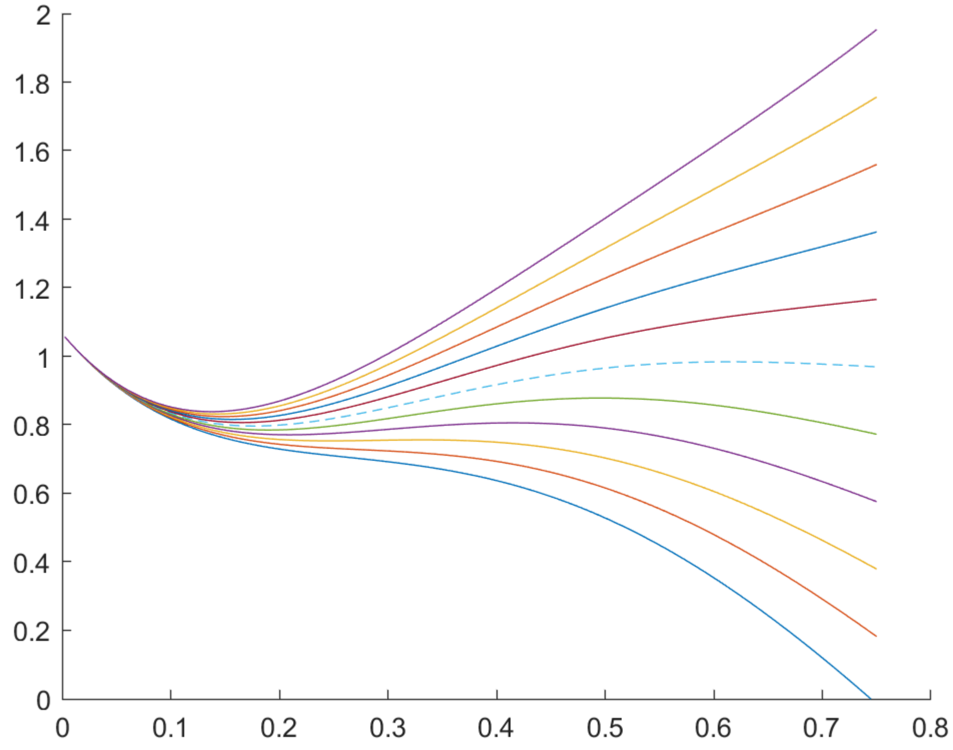


```
plotPoly(thrombinCoeffs(5,:), 1:300,5,0,2,0.9,1.1,0.02)
```

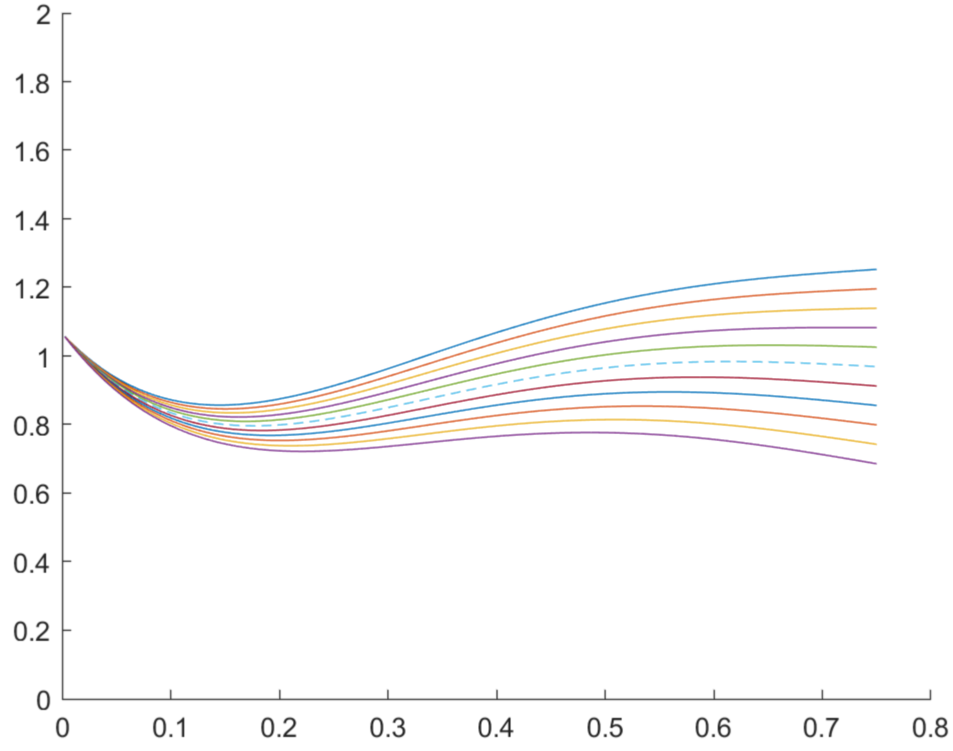

```
plotPoly(thrombinCoeffs(5,:), 1:300,6,0,2,0.9,1.1,0.02)
```

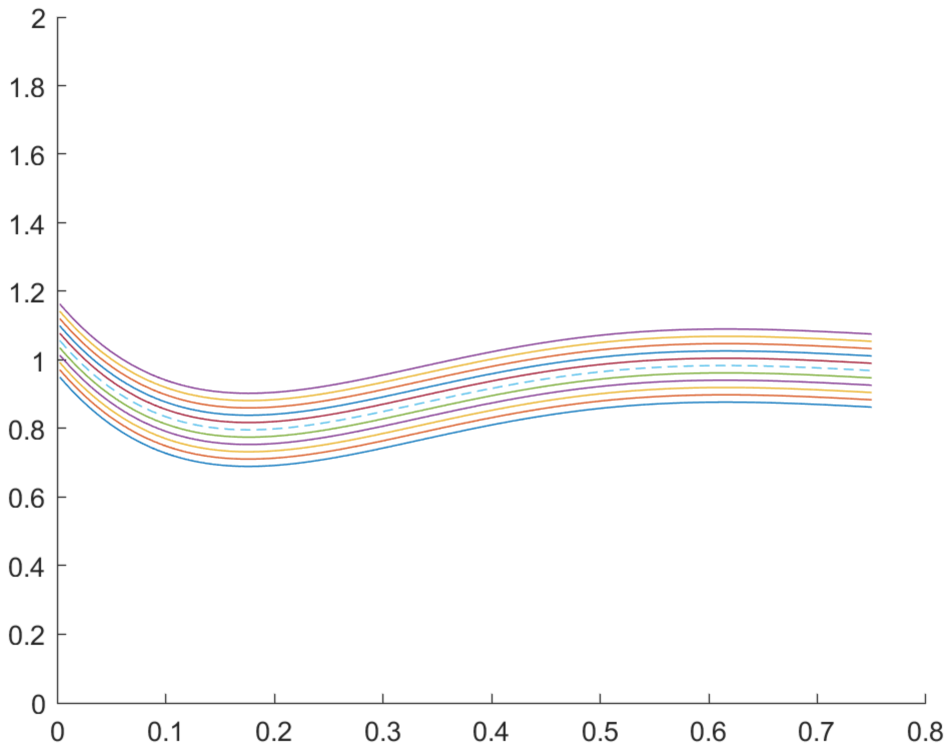


```
% GSK
plotPoly(WT_GSK_Coeffs(3,:), 1:500,1,0,2,0.9,1.1,0.02)
```

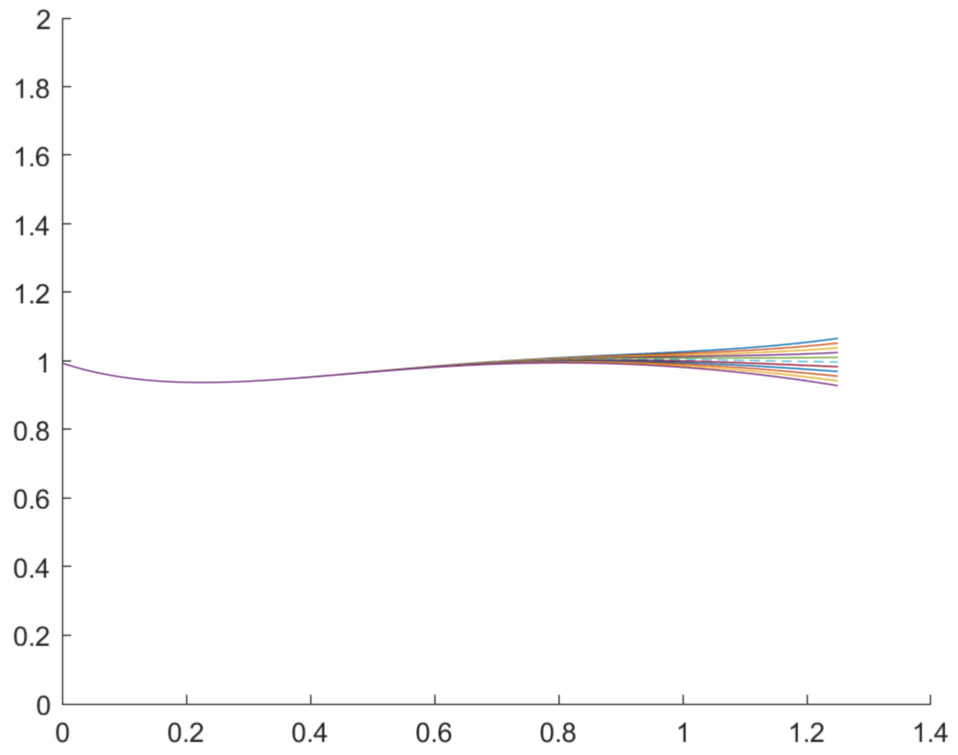


```
plotPoly(WT_GSK_Coeffs(3,:), 1:500,2,0,2,0.9,1.1,0.02)
```

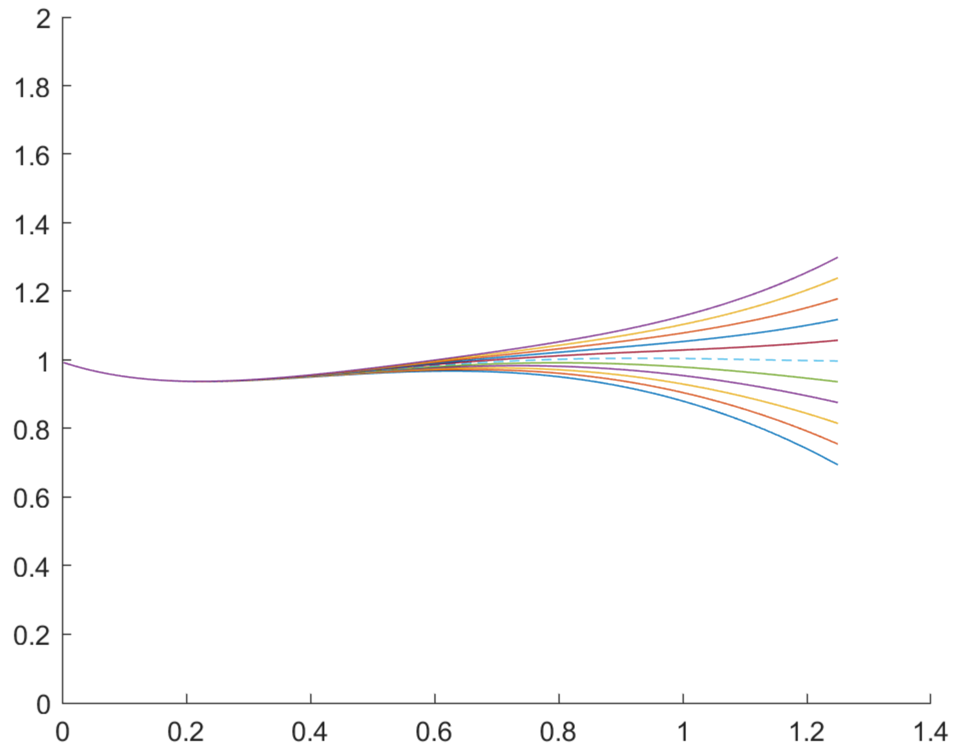

```
plotPoly(WT_GSK_Coeffs(3,:), 1:500,3,0,2,0.9,1.1,0.02)
```

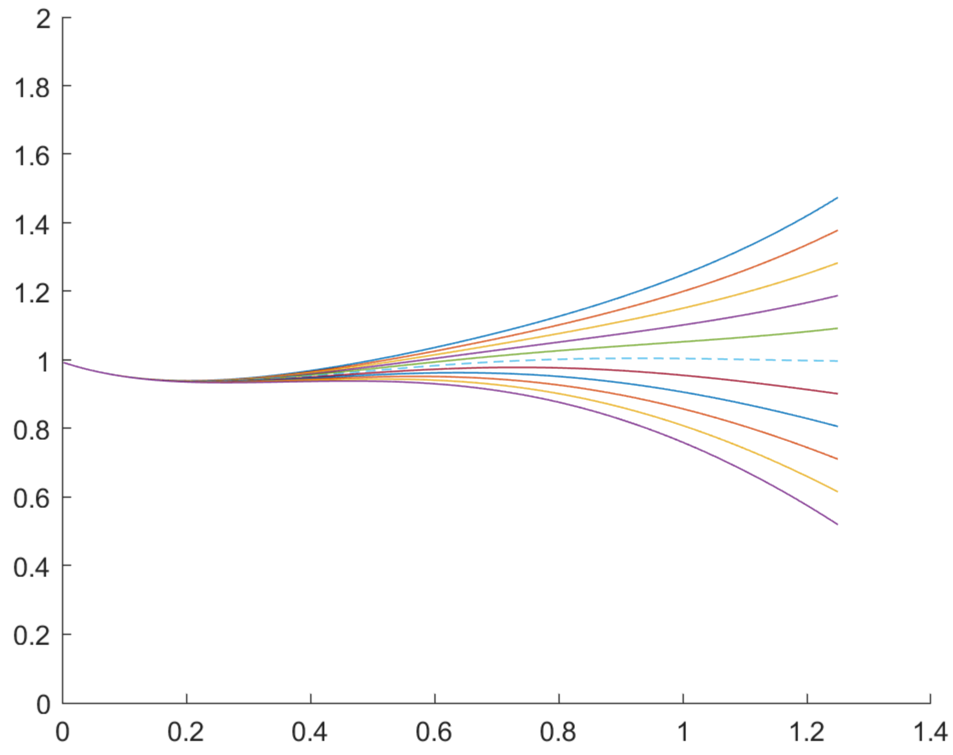


```
plotPoly(WT_GSK_Coeffs(3,:), 1:500,4,0,2,0.9,1.1,0.02)
```

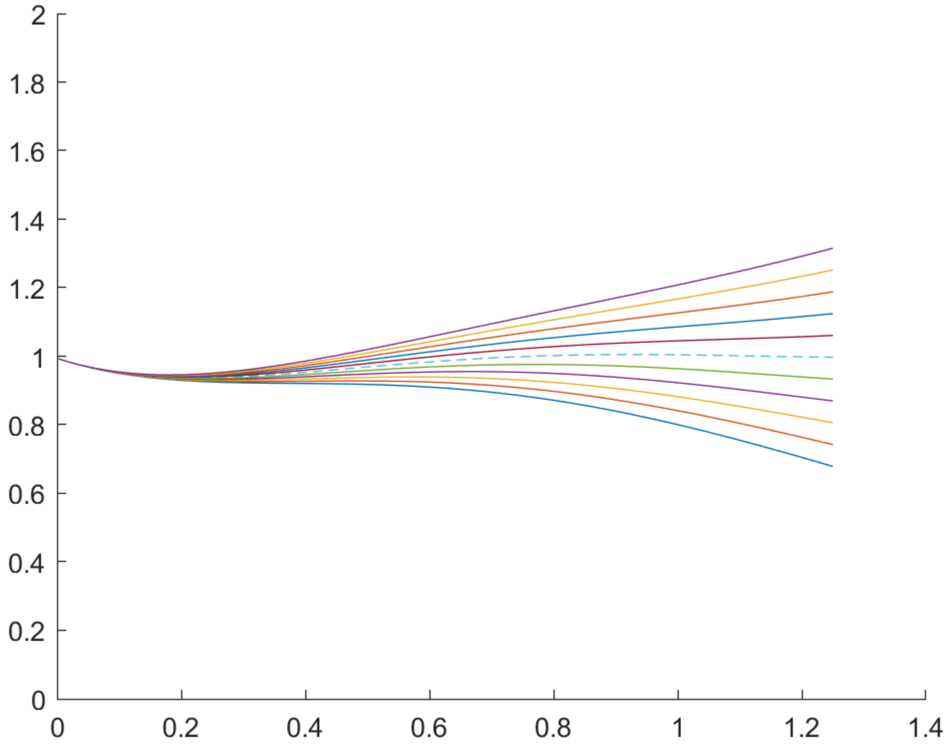


```
plotPoly(WT_GSK_Coeffs(3,:), 1:500,5,0,2,0.9,1.1,0.02)
```

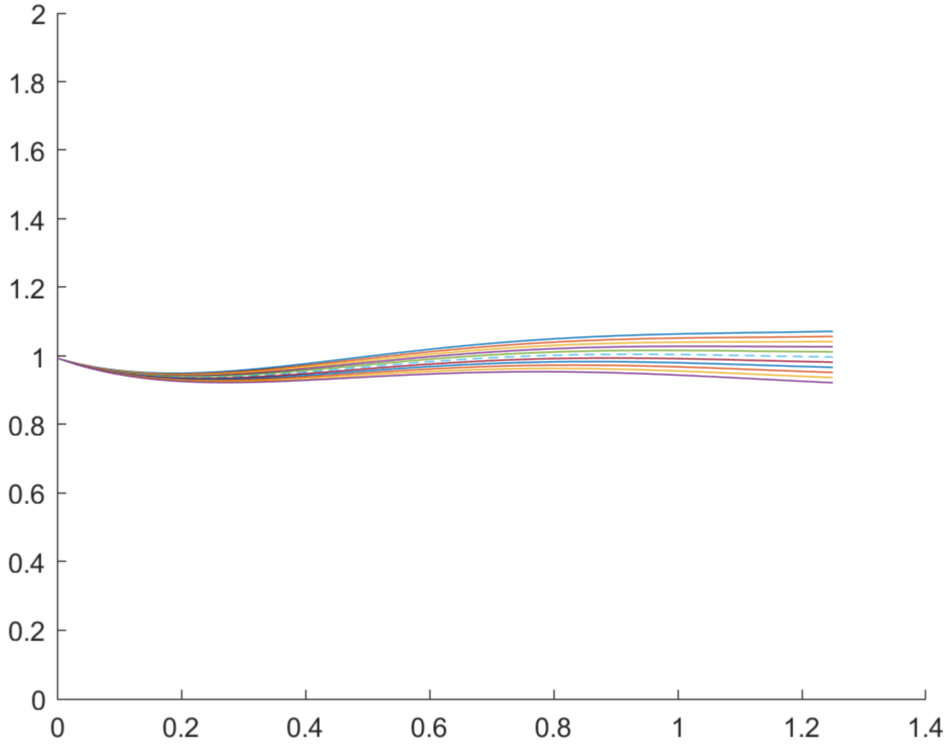

```
plotPoly(WT_GSK_Coeffs(3,:), 1:500,6,0,2,0.9,1.1,0.02)
```

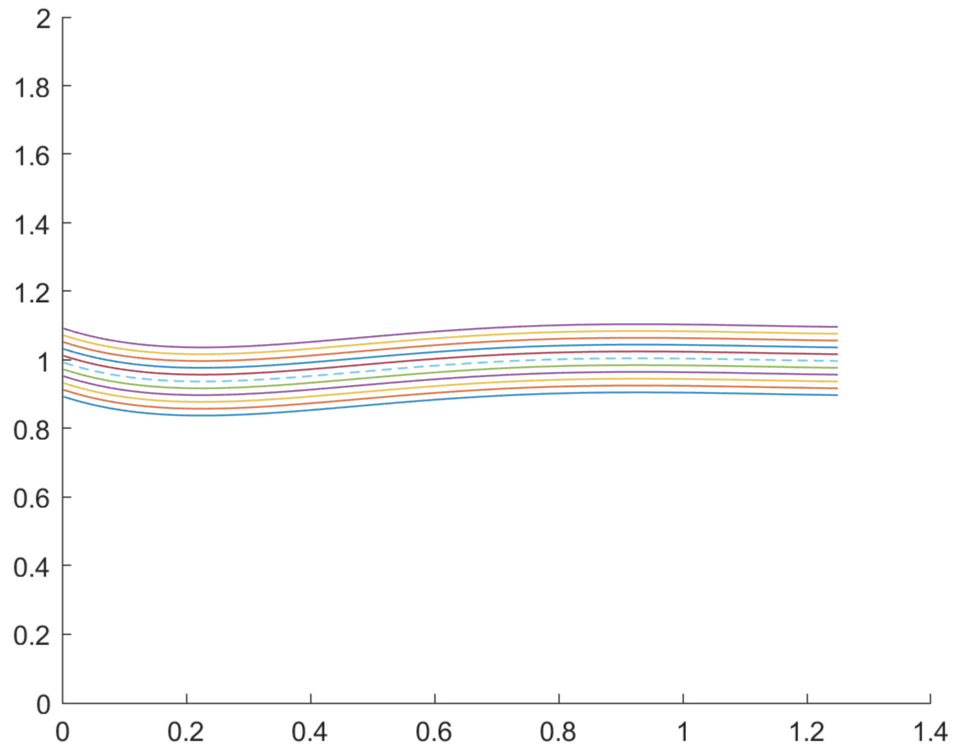

Summary Fits

Here, we are fitting the averaged response of all fits. In other words, for a given experiment with n trials, we have n polynomals of order 5.We now construct a summary fitted polynomial where each coefficient is the group average. In other words, given fit1 = a11x^5 + a21x^4.... and fitn = a1nx^5+..., the polynomials plotted here are avg(a11, a12..a1n)x^5 + avg(a21, a22..a2n)x^4+...

```

```

WT LPS Summary Fit

```
figure
hold on
plot(0:12, polyval(mean(WTCoeffslps50), 0:12))
plot(0:12, polyval(mean(FynCoeffslps50), 0:12))
ylim([0,2.5])
xlim([0,12])
hold off
```

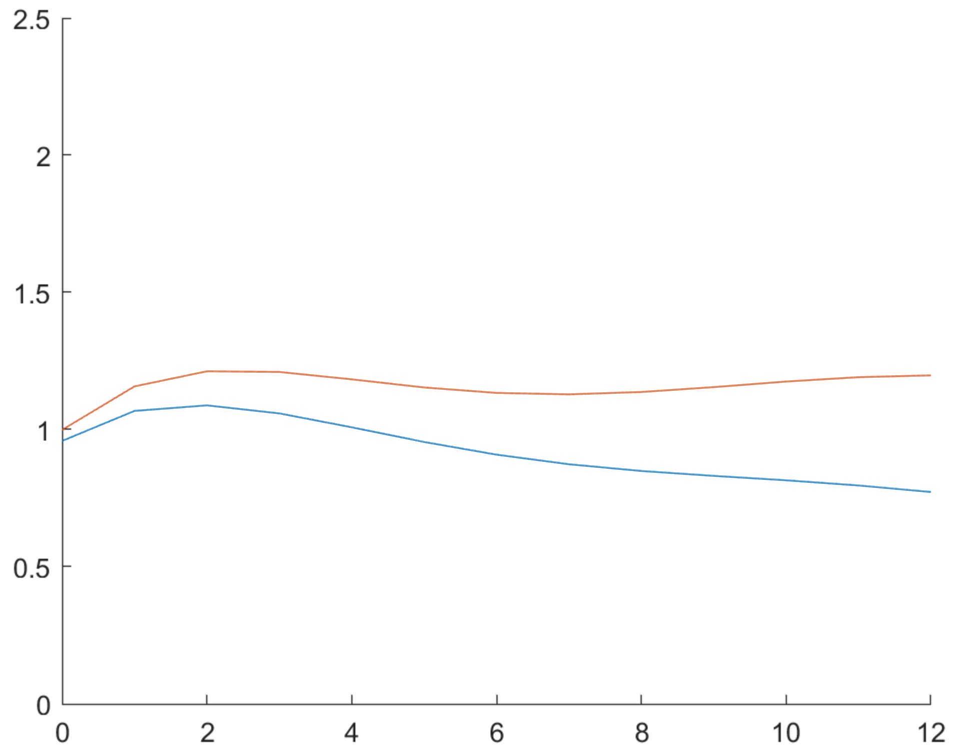


```
wtlps50_SummaryFits = (polyval(mean(WTCoeffslps50), 0:0.01:12)).'
```

```
wtlps50_SummaryFits = 1201×1
    0.9585
    0.9602
    0.9619
    0.9636
    0.9652
    0.9669
    0.9685
    0.9701
    0.9717
    0.9733
      ⋮
```

```
fynlps50_SummaryFits = (polyval(mean(FynCoeffslps50), 0:0.01:12)).'
```

```
fynlps50_SummaryFits = 1201×1
    0.9990
    1.0012
    1.0035
    1.0058
    1.0080
    1.0102
    1.0124
    1.0146
    1.0168
    1.0189
      ⋮
```

```
% WT LPS 50
figure
hold on
plot(0:12, polyval(mean(WTCoeffs), 0:12))
plot(0:12, polyval(mean(FynCoeffs), 0:12))
ylim([0,2.5])
hold off
```

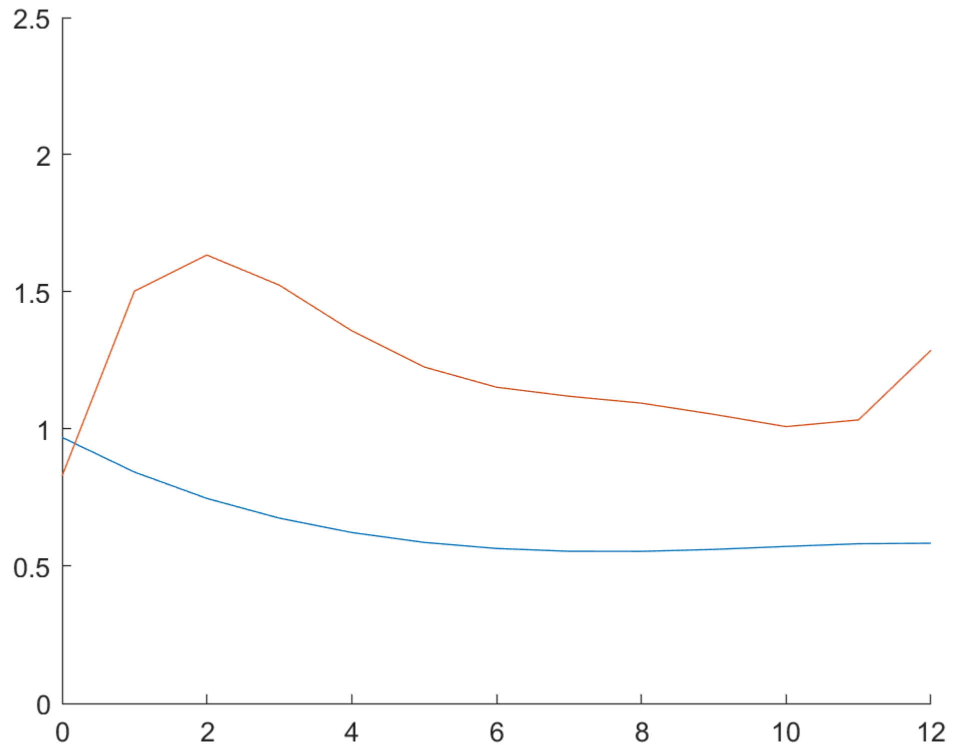


```
wt_lps100SummaryFits = (polyval(mean(WTCoeffs), 0:0.01:12)).'
```

```
wt_lps100SummaryFits = 1201×1
    0.9695
    0.9681
    0.9666
    0.9652
    0.9638
    0.9624
    0.9609
    0.9595
    0.9581
    0.9567
      ⋮
```

```
fyn_lps100SummaryFits = (polyval(mean(FynCoeffs), 0:0.01:12)).'
```

```
fyn_lps100SummaryFits = 1201×1
    0.8289
    0.8397
    0.8503
```

```
0.8609
0.8714
0.8818
0.8920
0.9022
0.9123
0.9223
  ⋮
```

```
figure
hold on

plot(0:0.01:1, polyval(mean(WT_GSK_Coeffs), 0:0.01:1))
plot(0:0.01:1, polyval(mean(CD36_GSK_Coeffs), 0:0.01:1))
WT_GSK_SummaryFit = polyval(mean(WT_GSK_Coeffs),0:0.01:1)
```

```
WT_GSK_SummaryFit = 1×101
    0.9920    0.9863    0.9809    0.9758    0.9712    0.9668    0.9628    0.9591 ···
```

```
CD36_GSK_SummaryFit = polyval(mean(CD36_GSK_Coeffs),0:0.01:1)
```

```
CD36_GSK_SummaryFit = 1×101
    0.9992    0.9927    0.9867    0.9811    0.9759    0.9710    0.9665    0.9624 ···
```

```
%plot(0:0.01:1, polyval(mean(fyn_GSK_Coeffs), 0:0.01:1))
ylim([0.9,1.1])
hold off
```

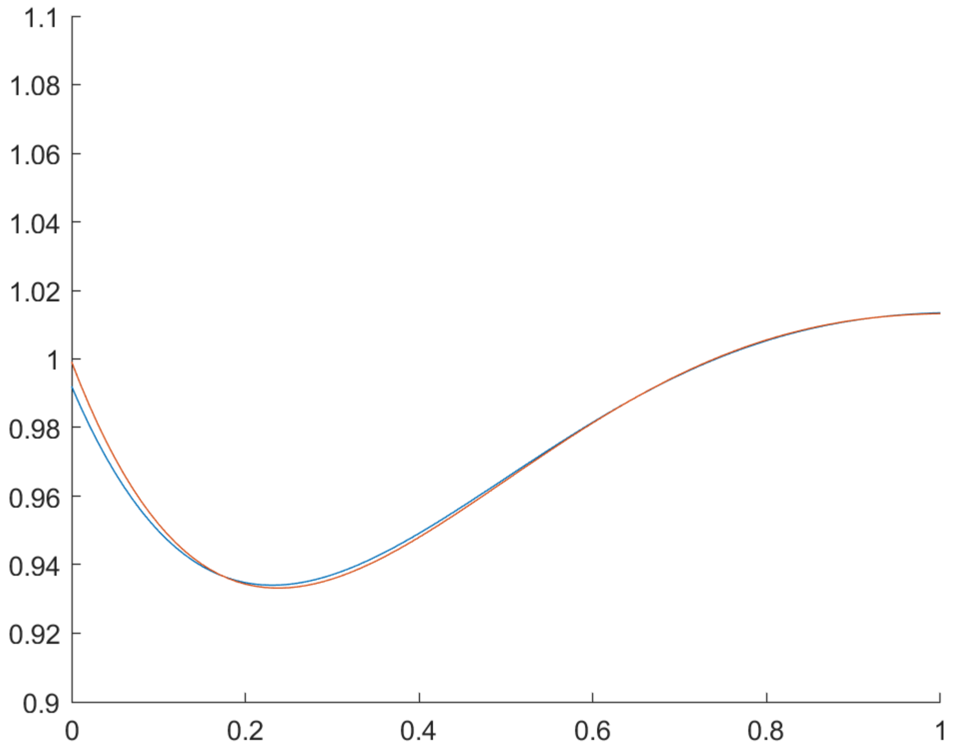

GSK Summary Fits

```
figure
hold on

plot(0:0.01:1, polyval(mean(WT_GSK_Coeffs_minus10), 0:0.01:1))
plot(0:0.01:1, polyval(mean(CD36_GSK_Coeffs_minus10), 0:0.01:1))
plot(0:0.01:1, polyval(mean(fyn_GSK_Coeffs_minus10), 0:0.01:1))
ylim([0.7,1.1])
hold off
```

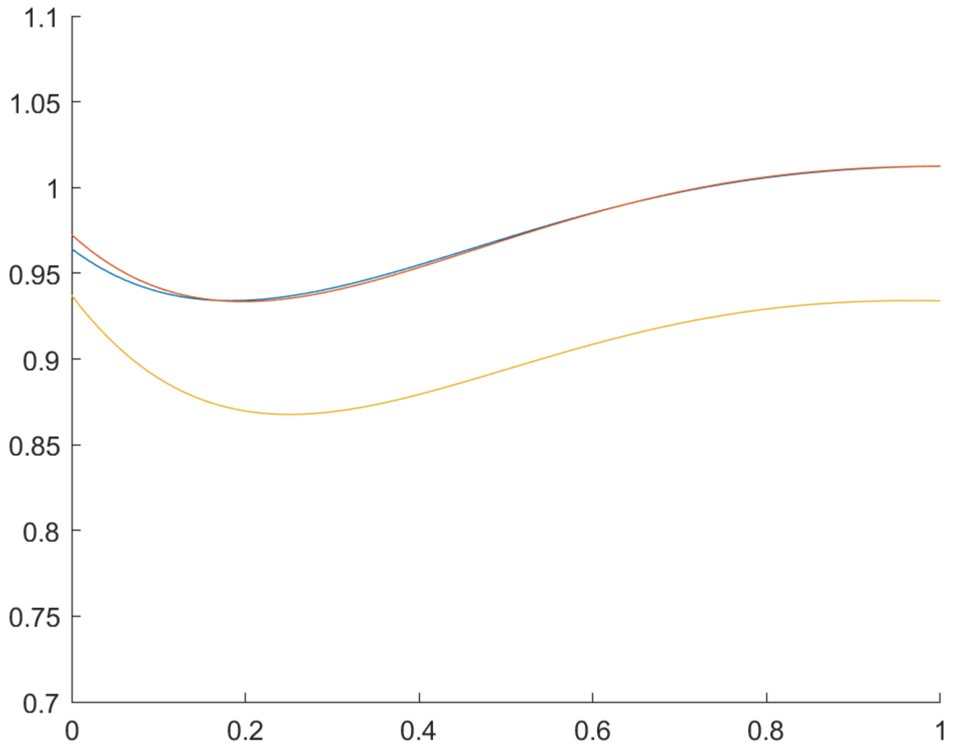


```
figure
hold on

plot(0:0.01:1, polyval(mean(WT_GSK_Coeffs_minus20), 0:0.01:1))
plot(0:0.01:1, polyval(mean(CD36_GSK_Coeffs_minus20), 0:0.01:1))
plot(0:0.01:1, polyval(mean(fyn_GSK_Coeffs_minus20), 0:0.01:1))
ylim([0.7,1.1])
hold off
```

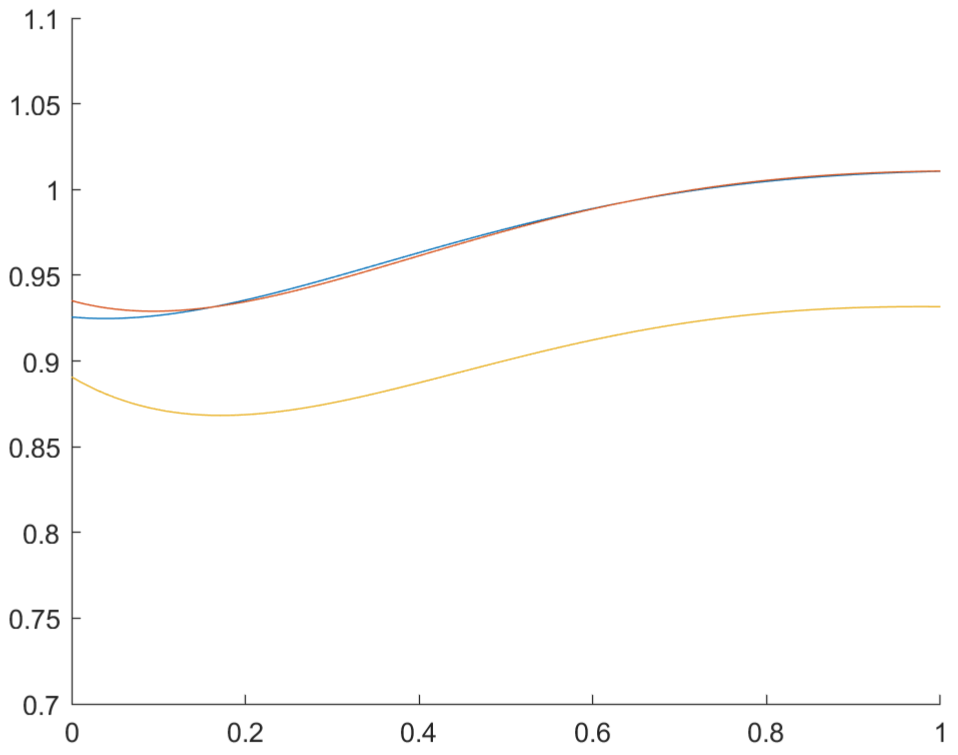


```
figure
hold on

plot(0:0.01:1, polyval(mean(WT_GSK_Coeffs_minus30), 0:0.01:1))
plot(0:0.01:1, polyval(mean(CD36_GSK_Coeffs_minus30), 0:0.01:1))
plot(0:0.01:1, polyval(mean(fyn_GSK_Coeffs_minus30), 0:0.01:1))
ylim([0.7,1.1])
hold off
```

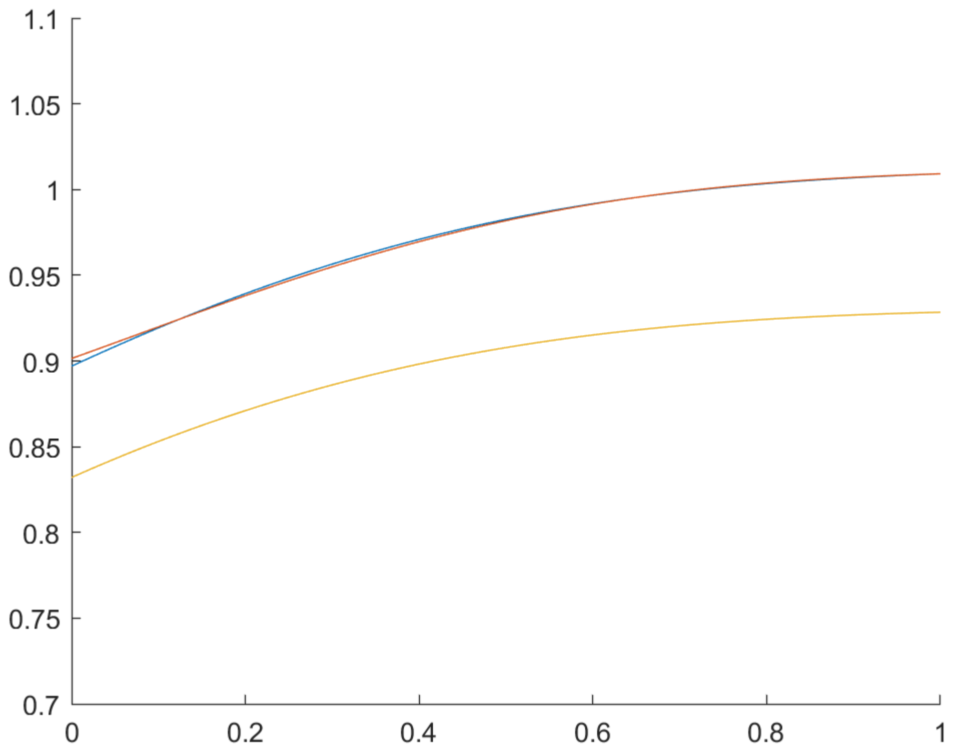


```
% show how ledge modification affects the fit

figure
hold on

plot(0:0.01:1, polyval(mean(WT_GSK_Coeffs), 0:0.01:1))
plot(0:0.01:1, polyval(mean(WT_GSK_Coeffs_minus10), 0:0.01:1))
plot(0:0.01:1, polyval(mean(WT_GSK_Coeffs_minus20), 0:0.01:1))
plot(0:0.01:1, polyval(mean(WT_GSK_Coeffs_minus30), 0:0.01:1))

ylim([0.7,1.1])
hold off
```

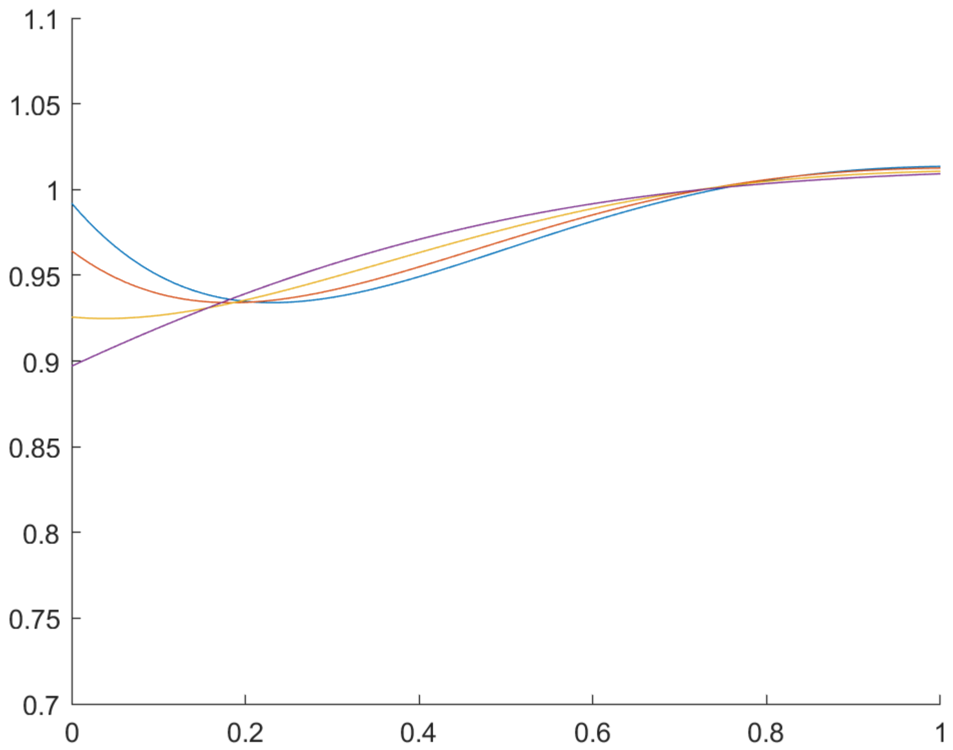


```
figure
hold on

plot(0:0.01:1, polyval(mean(CD36_GSK_Coeffs), 0:0.01:1))
plot(0:0.01:1, polyval(mean(CD36_GSK_Coeffs_minus10), 0:0.01:1))
plot(0:0.01:1, polyval(mean(CD36_GSK_Coeffs_minus20), 0:0.01:1))
plot(0:0.01:1, polyval(mean(CD36_GSK_Coeffs_minus30), 0:0.01:1))

ylim([0.7,1.1])
hold off
```

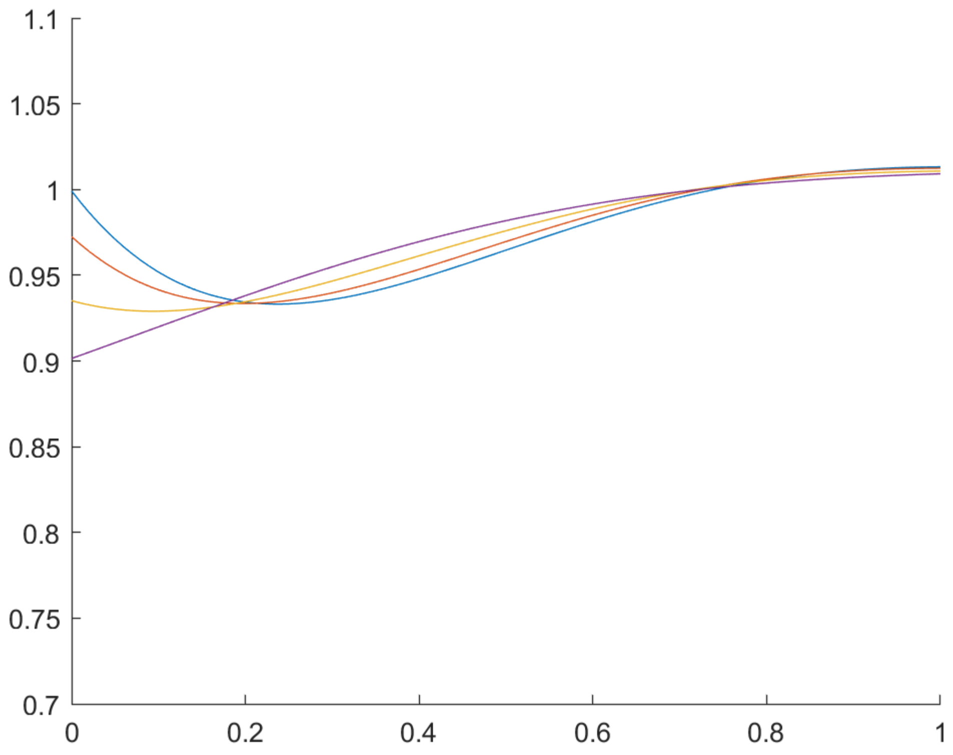


```
figure
hold on

plot(0:0.01:1, polyval(mean(fyn_GSK_Coeffs), 0:0.01:1))
plot(0:0.01:1, polyval(mean(fyn_GSK_Coeffs_minus10), 0:0.01:1))
plot(0:0.01:1, polyval(mean(fyn_GSK_Coeffs_minus20), 0:0.01:1))
plot(0:0.01:1, polyval(mean(fyn_GSK_Coeffs_minus30), 0:0.01:1))

ylim([0.7,1.1])
hold off
```

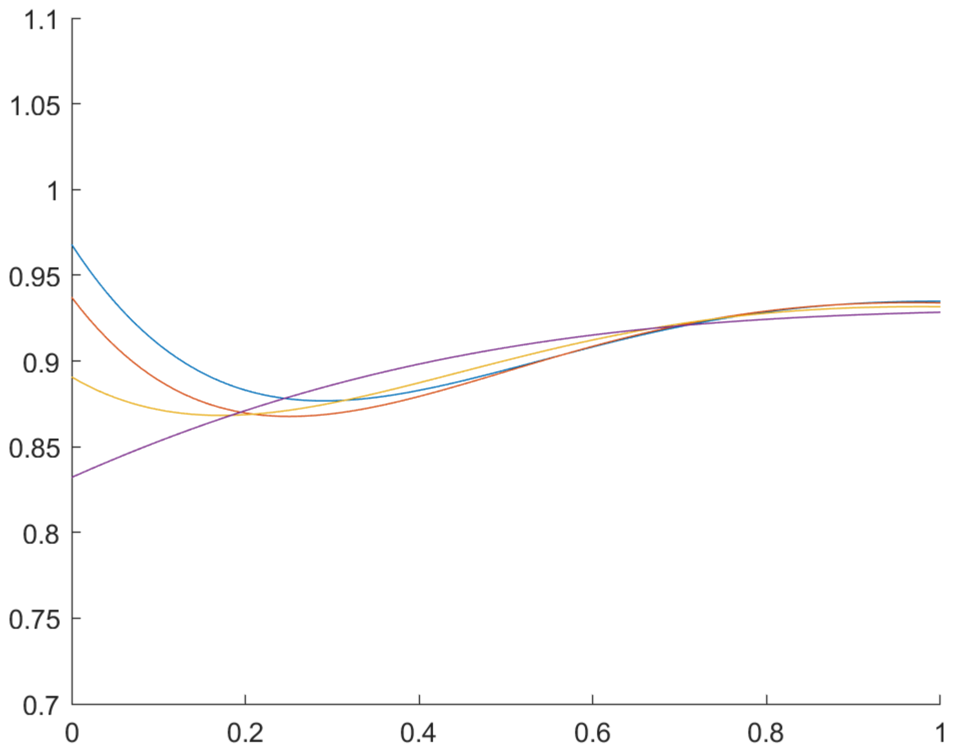

Thrombin Summary Fits

```
figure
hold on
plot(0:0.01:1.2, polyval(mean(thrombinCoeffs), 0:0.01:1.2))
plot(0:0.01:1.2, polyval(mean(thrombindevdCoeffs), 0:0.01:1.2))
ylim([0.5,1.3])
hold off
```

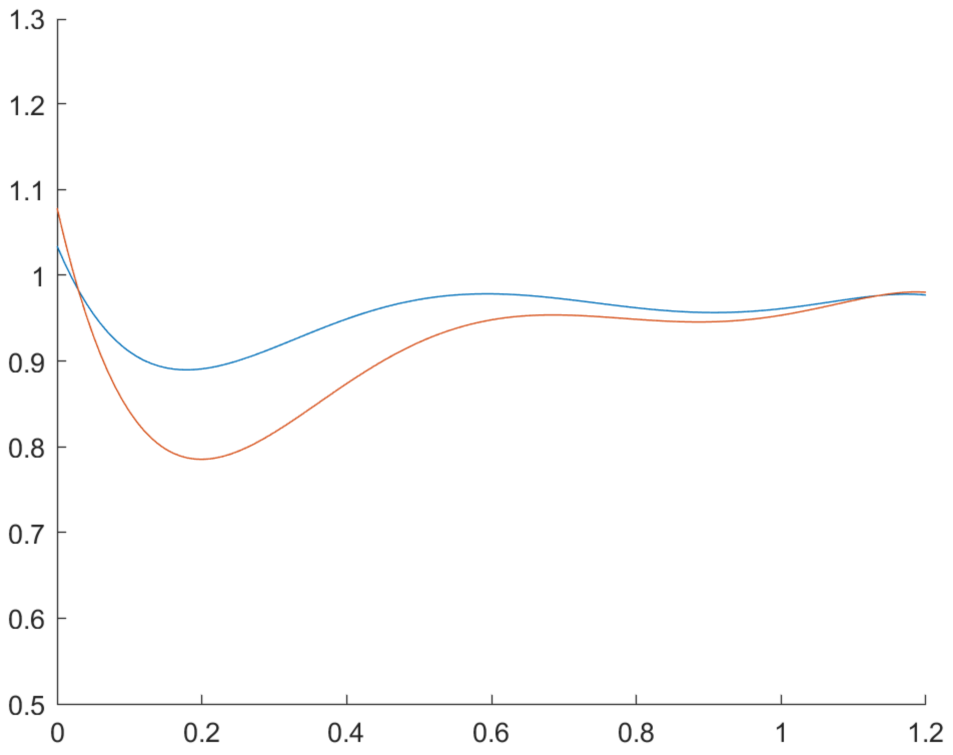


```
tr=0:0.0025:2.1
```

```
tr = 1×841
         0    0.0025    0.0050    0.0075    0.0100    0.0125    0.0150    0.0175 ⋯
```

```
tr=tr.'
```

```
tr = 841×1
         0
    0.0025
    0.0050
    0.0075
    0.0100
    0.0125
    0.0150
    0.0175
    0.0200
    0.0225
      ⋮
```

```
Thrombin_DEVD_Fitted = [tr,polyval(mean(thrombinCoeffs), tr), polyval(mean(thrombindevdCoeffs),
```

```
Thrombin_DEVD_Fitted = 841×3
         0    1.0342    1.0785
    0.0025    1.0292    1.0693
    0.0050    1.0243    1.0604
    0.0075    1.0196    1.0516
    0.0100    1.0150    1.0430
    0.0125    1.0105    1.0346
    0.0150    1.0060    1.0264
```

```
    0.0175    1.0017    1.0184
    0.0200    0.9976    1.0106
    0.0225    0.9935    1.0029
      ⋮
```

```
GSK_Fitted = [tr, polyval(mean(WT_GSK_Coeffs), tr), polyval(mean(CD36_GSK_Coeffs), tr), polyval
```

```
GSK_Fitted = 841×4
         0    0.9920    0.9992    0.9679
    0.0025    0.9905    0.9975    0.9660
    0.0050    0.9891    0.9959    0.9641
    0.0075    0.9877    0.9943    0.9622
    0.0100    0.9863    0.9927    0.9604
    0.0125    0.9849    0.9912    0.9585
    0.0150    0.9835    0.9897    0.9567
    0.0175    0.9822    0.9882    0.9550
    0.0200    0.9809    0.9867    0.9532
    0.0225    0.9796    0.9853    0.9515
      ⋮
```

Evaluate goodness of poly fits

```
GOF_wt = PolyGOF(wt, WTCoeffs,12)
```

```
GOFMatrix = 1×2
     0     0
pred = 1×4668
    1.0672    1.0675    1.0678    1.0681    1.0684    1.0686    1.0689    1.0692 ⋯
observed = 4668×1
    0.9432
    0.9431
    0.9432
    0.9426
    0.9422
    0.9422
    0.9424
    0.9419
    0.9425
    0.9436
      ⋮
observed2 = 4668×2
    1.0000    0.9432
    1.0000    0.9431
    1.0000    0.9432
    1.0000    0.9426
    1.0000    0.9422
    1.0000    0.9422
    1.0000    0.9424
    1.0000    0.9419
    1.0000    0.9425
    1.0000    0.9436
      ⋮
linreg = 2×1
```

```
    0.0322
    0.9448
GOFMatrix = 2×2
         0         0
    0.0322    0.9448
pred = 1×4668
    1.1397    1.1398    1.1398    1.1399    1.1399    1.1399    1.1400    1.1400 ···
observed = 4668×1
    0.9930
    0.9923
    0.9919
    0.9922
    0.9926
    0.9927
    0.9922
    0.9922
    0.9929
    0.9936
      ⋮
observed2 = 4668×2
    1.0000    0.9930
    1.0000    0.9923
    1.0000    0.9919
    1.0000    0.9922
    1.0000    0.9926
    1.0000    0.9927
    1.0000    0.9922
    1.0000    0.9922
    1.0000    0.9929
    1.0000    0.9936
      ⋮
linreg = 2×1
    0.0510
    0.9143
GOFMatrix = 3×2
         0         0
    0.0322    0.9448
    0.0510    0.9143
pred = 1×4668
    0.9930    0.9931    0.9933    0.9934    0.9935    0.9937    0.9938    0.9940 ···
observed = 4668×1
    0.9967
    0.9970
    0.9974
    0.9980
    0.9982
    0.9981
    0.9982
    0.9986
    0.9992
    0.9988
      ⋮
observed2 = 4668×2
    1.0000    0.9967
    1.0000    0.9970
    1.0000    0.9974
    1.0000    0.9980
    1.0000    0.9982
    1.0000    0.9981
    1.0000    0.9982
    1.0000    0.9986
    1.0000    0.9992
```

```
    1.0000    0.9988
      ⋮
linreg = 2×1
    0.0235
    0.9804
GOFMatrix = 4×2
         0         0
    0.0322    0.9448
    0.0510    0.9143
    0.0235    0.9804
pred = 1×4668
    1.0184    1.0184    1.0185    1.0185    1.0186    1.0186    1.0187    1.0187 ⋯
observed = 4668×1
    0.9972
    0.9975
    0.9975
    0.9978
    0.9975
    0.9976
    0.9979
    0.9978
    0.9978
    0.9980
      ⋮
observed2 = 4668×2
    1.0000    0.9972
    1.0000    0.9975
    1.0000    0.9975
    1.0000    0.9978
    1.0000    0.9975
    1.0000    0.9976
    1.0000    0.9979
    1.0000    0.9978
    1.0000    0.9978
    1.0000    0.9980
      ⋮
linreg = 2×1
    0.1507
    0.8672
GOFMatrix = 5×2
         0         0
    0.0322    0.9448
    0.0510    0.9143
    0.0235    0.9804
    0.1507    0.8672
pred = 1×4668
    0.8023    0.8012    0.8001    0.7991    0.7980    0.7969    0.7958    0.7948 ⋯
observed = 4668×1
    1.0041
    1.0040
    1.0038
    1.0038
    1.0032
    1.0034
    1.0034
    1.0041
    1.0039
    1.0037
      ⋮
observed2 = 4668×2
    1.0000    1.0041
```

```
    1.0000    1.0040
    1.0000    1.0038
    1.0000    1.0038
    1.0000    1.0032
    1.0000    1.0034
    1.0000    1.0034
    1.0000    1.0041
    1.0000    1.0039
    1.0000    1.0037
      :
linreg = 2×1
    0.0466
    0.8802
GOFMatrix = 6×2
         0         0
    0.0322    0.9448
    0.0510    0.9143
    0.0235    0.9804
    0.1507    0.8672
    0.0466    0.8802
pred = 1×4668
    0.9177    0.9169    0.9161    0.9152    0.9144    0.9136    0.9128    0.9119 ···
observed = 4668×1
    1.0008
    1.0004
    1.0008
    1.0006
    1.0001
    1.0001
    1.0004
    1.0011
    1.0012
    1.0007
      :
observed2 = 4668×2
    1.0000    1.0008
    1.0000    1.0004
    1.0000    1.0008
    1.0000    1.0006
    1.0000    1.0001
    1.0000    1.0001
    1.0000    1.0004
    1.0000    1.0011
    1.0000    1.0012
    1.0000    1.0007
      :
linreg = 2×1
    0.0123
    0.9710
GOFMatrix = 7×2
         0         0
    0.0322    0.9448
    0.0510    0.9143
    0.0235    0.9804
    0.1507    0.8672
    0.0466    0.8802
    0.0123    0.9710
pred = 1×4668
    0.9163    0.9148    0.9134    0.9120    0.9105    0.9091    0.9077    0.9063 ···
observed = 4668×1
    0.9935
    0.9936
```

```
    0.9934
    0.9952
    0.9950
    0.9945
    0.9948
    0.9950
    0.9959
    0.9959
      :
      :
observed2 = 4668×2
    1.0000    0.9935
    1.0000    0.9936
    1.0000    0.9934
    1.0000    0.9952
    1.0000    0.9950
    1.0000    0.9945
    1.0000    0.9948
    1.0000    0.9950
    1.0000    0.9959
    1.0000    0.9959
      :
      :
linreg = 2×1
    0.0909
    0.7820
GOFMatrix = 8×2
         0         0
    0.0322    0.9448
    0.0510    0.9143
    0.0235    0.9804
    0.1507    0.8672
    0.0466    0.8802
    0.0123    0.9710
    0.0909    0.7820
pred = 1×4668
    0.9172    0.9154    0.9137    0.9119    0.9102    0.9084    0.9067    0.9050 ···
observed = 4668×1
    1.0065
    1.0064
    1.0060
    1.0050
    1.0047
    1.0044
    1.0042
    1.0043
    1.0042
    1.0042
      :
      :
observed2 = 4668×2
    1.0000    1.0065
    1.0000    1.0064
    1.0000    1.0060
    1.0000    1.0050
    1.0000    1.0047
    1.0000    1.0044
    1.0000    1.0042
    1.0000    1.0043
    1.0000    1.0042
    1.0000    1.0042
      :
      :
linreg = 2×1
    0.1107
```

```
    0.7045
GOFMatrix = 9×2
         0         0
    0.0322    0.9448
    0.0510    0.9143
    0.0235    0.9804
    0.1507    0.8672
    0.0466    0.8802
    0.0123    0.9710
    0.0909    0.7820
    0.1107    0.7045
pred = 1×4668
    0.9588    0.9589    0.9589    0.9590    0.9590    0.9591    0.9591    0.9592 ···
observed = 4668×1
    0.9936
    0.9947
    0.9953
    0.9964
    0.9975
    0.9984
    0.9996
    1.0008
    1.0014
    1.0030
      ⋮
observed2 = 4668×2
    1.0000    0.9936
    1.0000    0.9947
    1.0000    0.9953
    1.0000    0.9964
    1.0000    0.9975
    1.0000    0.9984
    1.0000    0.9996
    1.0000    1.0008
    1.0000    1.0014
    1.0000    1.0030
      ⋮
linreg = 2×1
    0.0026
    0.9958
GOFMatrix = 10×2
         0         0
    0.0322    0.9448
    0.0510    0.9143
    0.0235    0.9804
    0.1507    0.8672
    0.0466    0.8802
    0.0123    0.9710
    0.0909    0.7820
    0.1107    0.7045
    0.0026    0.9958
pred = 1×4668
    0.9670    0.9670    0.9671    0.9671    0.9672    0.9673    0.9673    0.9674 ···
observed = 4668×1
    1.0058
    1.0072
    1.0091
    1.0096
    1.0103
    1.0110
    1.0110
    1.0112
    1.0117
```

```
    1.0114
      ⋮

observed2 = 4668×2
    1.0000    1.0058
    1.0000    1.0072
    1.0000    1.0091
    1.0000    1.0096
    1.0000    1.0103
    1.0000    1.0110
    1.0000    1.0110
    1.0000    1.0112
    1.0000    1.0117
    1.0000    1.0114
      ⋮

linreg = 2×1
    0.0008
    0.9987
GOFMatrix = 11×2
         0         0
    0.0322    0.9448
    0.0510    0.9143
    0.0235    0.9804
    0.1507    0.8672
    0.0466    0.8802
    0.0123    0.9710
    0.0909    0.7820
    0.1107    0.7045
    0.0026    0.9958
      ⋮

pred = 1×4668
    0.9599    0.9599    0.9600    0.9601    0.9602    0.9603    0.9604    0.9605 ⋯
observed = 4668×1
    1.0134
    1.0146
    1.0157
    1.0164
    1.0172
    1.0179
    1.0185
    1.0185
    1.0182
    1.0180
      ⋮

observed2 = 4668×2
    1.0000    1.0134
    1.0000    1.0146
    1.0000    1.0157
    1.0000    1.0164
    1.0000    1.0172
    1.0000    1.0179
    1.0000    1.0185
    1.0000    1.0185
    1.0000    1.0182
    1.0000    1.0180
      ⋮

linreg = 2×1
    0.0011
    0.9982
GOFMatrix = 12×2
```

```
         0         0
    0.0322    0.9448
    0.0510    0.9143
    0.0235    0.9804
    0.1507    0.8672
    0.0466    0.8802
    0.0123    0.9710
    0.0909    0.7820
    0.1107    0.7045
    0.0026    0.9958
      ⋮

pred = 1×4668
    0.9726    0.9726    0.9726    0.9727    0.9727    0.9728    0.9728    0.9729 ···
observed = 4668×1
    1.0059
    1.0063
    1.0065
    1.0068
    1.0068
    1.0072
    1.0075
    1.0078
    1.0086
    1.0091
      ⋮

observed2 = 4668×2
    1.0000    1.0059
    1.0000    1.0063
    1.0000    1.0065
    1.0000    1.0068
    1.0000    1.0068
    1.0000    1.0072
    1.0000    1.0075
    1.0000    1.0078
    1.0000    1.0086
    1.0000    1.0091
      ⋮

linreg = 2×1
    0.0009
    0.9986
GOFMatrix = 13×2
         0         0
    0.0322    0.9448
    0.0510    0.9143
    0.0235    0.9804
    0.1507    0.8672
    0.0466    0.8802
    0.0123    0.9710
    0.0909    0.7820
    0.1107    0.7045
    0.0026    0.9958
      ⋮

GOF_wt = 13×2
         0         0
    0.0322    0.9448
    0.0510    0.9143
    0.0235    0.9804
    0.1507    0.8672
    0.0466    0.8802
    0.0123    0.9710
    0.0909    0.7820
```

```
    0.1107    0.7045
    0.0026    0.9958
      ⋮
```

```
GOF_Thrombin = PolyGOF(thrombin, thrombinCoeffs,6)
```

```
GOFMatrix = 1×2
     0     0
pred = 1×512
    1.0139    1.0106    1.0073    1.0041    1.0010    0.9979    0.9948    0.9919 ⋯
observed = 512×1
    0.9998
    1.0002
    0.9991
    0.9989
    0.9992
    0.9993
    0.9999
    1.0007
    1.0968
    1.0066
      ⋮
observed2 = 512×2
    1.0000    0.9998
    1.0000    1.0002
    1.0000    0.9991
    1.0000    0.9989
    1.0000    0.9992
    1.0000    0.9993
    1.0000    0.9999
    1.0000    1.0007
    1.0000    1.0968
    1.0000    1.0066
      ⋮
linreg = 2×1
    0.0921
    0.9011
GOFMatrix = 2×2
         0         0
    0.0921    0.9011
pred = 1×512
    1.0341    1.0295    1.0251    1.0208    1.0166    1.0124    1.0084    1.0044 ⋯
observed = 512×1
    1.0005
    0.9998
    0.9998
    0.9993
    1.0002
    1.0006
    1.0005
    1.0003
    1.0003
    1.0002
      ⋮
observed2 = 512×2
    1.0000    1.0005
    1.0000    0.9998
    1.0000    0.9998
    1.0000    0.9993
    1.0000    1.0002
```

```
    1.0000    1.0006
    1.0000    1.0005
    1.0000    1.0003
    1.0000    1.0003
    1.0000    1.0002
      ⋮
linreg = 2×1
    0.0868
    0.9089
GOFMatrix = 3×2
         0         0
    0.0921    0.9011
    0.0868    0.9089
pred = 1×512
    1.0158    1.0144    1.0131    1.0117    1.0105    1.0092    1.0080    1.0068 ⋯
observed = 512×1
    1.0002
    0.9997
    1.0000
    1.0004
    1.0016
    1.0019
    1.0011
    1.0003
    1.0005
    1.0005
      ⋮
observed2 = 512×2
    1.0000    1.0002
    1.0000    0.9997
    1.0000    1.0000
    1.0000    1.0004
    1.0000    1.0016
    1.0000    1.0019
    1.0000    1.0011
    1.0000    1.0003
    1.0000    1.0005
    1.0000    1.0005
      ⋮
linreg = 2×1
    0.2551
    0.7398
GOFMatrix = 4×2
         0         0
    0.0921    0.9011
    0.0868    0.9089
    0.2551    0.7398
pred = 1×512
    1.0241    1.0218    1.0195    1.0173    1.0151    1.0130    1.0109    1.0089 ⋯
observed = 512×1
    1.0005
    1.0000
    1.0000
    0.9998
    0.9988
    0.9987
    0.9988
    0.9985
    0.9987
    0.9988
      ⋮
```

```
observed2 = 512×2
    1.0000    1.0005
    1.0000    1.0000
    1.0000    1.0000
    1.0000    0.9998
    1.0000    0.9988
    1.0000    0.9987
    1.0000    0.9988
    1.0000    0.9985
    1.0000    0.9987
    1.0000    0.9988
      ⋮
linreg = 2×1
    0.1698
    0.8238
GOFMatrix = 5×2
         0         0
    0.0921    0.9011
    0.0868    0.9089
    0.2551    0.7398
    0.1698    0.8238
pred = 1×512
    1.0567    1.0476    1.0387    1.0300    1.0215    1.0132    1.0051    0.9972 ⋯
observed = 512×1
    1.0000
    0.9999
    1.0005
    1.0011
    1.0007
    1.0009
    0.9999
    0.9988
    0.9971
    0.9968
      ⋮
observed2 = 512×2
    1.0000    1.0000
    1.0000    0.9999
    1.0000    1.0005
    1.0000    1.0011
    1.0000    1.0007
    1.0000    1.0009
    1.0000    0.9999
    1.0000    0.9988
    1.0000    0.9971
    1.0000    0.9968
      ⋮
linreg = 2×1
    0.1004
    0.8924
GOFMatrix = 6×2
         0         0
    0.0921    0.9011
    0.0868    0.9089
    0.2551    0.7398
    0.1698    0.8238
    0.1004    0.8924
pred = 1×512
    1.0306    1.0221    1.0139    1.0059    0.9981    0.9905    0.9832    0.9761 ⋯
observed = 512×1
    1.0000
    1.0005
```

```
    1.0002
    1.0005
    1.0007
    1.0011
    1.0023
    1.0036
    1.0034
    1.0034
      ⋮

observed2 = 512×2
    1.0000    1.0000
    1.0000    1.0005
    1.0000    1.0002
    1.0000    1.0005
    1.0000    1.0007
    1.0000    1.0011
    1.0000    1.0023
    1.0000    1.0036
    1.0000    1.0034
    1.0000    1.0034
      ⋮

linreg = 2×1
    0.2413
    0.7486
GOFMatrix = 7×2
         0         0
    0.0921    0.9011
    0.0868    0.9089
    0.2551    0.7398
    0.1698    0.8238
    0.1004    0.8924
    0.2413    0.7486
GOF_Thrombin = 7×2
         0         0
    0.0921    0.9011
    0.0868    0.9089
    0.2551    0.7398
    0.1698    0.8238
    0.1004    0.8924
    0.2413    0.7486
```

```
GOF_GSK = PolyGOF(WT_GSK, WT_GSK_Coeffs,6)
```

```
GOFMatrix = 1×2
     0     0
pred = 1×840
    0.9898    0.9885    0.9872    0.9859    0.9847    0.9835    0.9822    0.9810 ⋯
observed = 840×1
    1.0131
    1.0132
    1.0131
    1.0127
    1.0124
    1.0125
    1.0127
    1.0125
    1.0128
    1.0124
      ⋮

observed2 = 840×2
    1.0000    1.0131
```

```
    1.0000    1.0132
    1.0000    1.0131
    1.0000    1.0127
    1.0000    1.0124
    1.0000    1.0125
    1.0000    1.0127
    1.0000    1.0125
    1.0000    1.0128
    1.0000    1.0124
      :
      :
linreg = 2×1
    0.1853
    0.8108
GOFMatrix = 2×2
         0         0
    0.1853    0.8108
pred = 1×840
    0.9720    0.9706    0.9691    0.9678    0.9664    0.9651    0.9638    0.9625 ⋯
observed = 840×1
    1.0045
    1.0041
    1.0041
    1.0041
    1.0040
    1.0040
    1.0040
    1.0039
    1.0035
    1.0032
      :
      :
observed2 = 840×2
    1.0000    1.0045
    1.0000    1.0041
    1.0000    1.0041
    1.0000    1.0041
    1.0000    1.0040
    1.0000    1.0040
    1.0000    1.0040
    1.0000    1.0039
    1.0000    1.0035
    1.0000    1.0032
      :
      :
linreg = 2×1
    0.0971
    0.9023
GOFMatrix = 3×2
         0         0
    0.1853    0.8108
    0.0971    0.9023
pred = 1×840
    0.9924    0.9910    0.9895    0.9881    0.9867    0.9854    0.9840    0.9827 ⋯
observed = 840×1
    1.0274
    1.0276
    1.0276
    1.0272
    1.0271
    1.0266
    1.0265
    1.0262
    1.0263
    1.0262
```

```
      ⋮
observed2 = 840×2
    1.0000    1.0274
    1.0000    1.0276
    1.0000    1.0276
    1.0000    1.0272
    1.0000    1.0271
    1.0000    1.0266
    1.0000    1.0265
    1.0000    1.0262
    1.0000    1.0263
    1.0000    1.0262
      ⋮
linreg = 2×1
    0.2388
    0.7591
GOFMatrix = 4×2
         0         0
    0.1853    0.8108
    0.0971    0.9023
    0.2388    0.7591
pred = 1×840
    1.0192    1.0177    1.0162    1.0148    1.0134    1.0120    1.0106    1.0092 ⋯
observed = 840×1
    1.0443
    1.0439
    1.0438
    1.0438
    1.0437
    1.0438
    1.0434
    1.0436
    1.0433
    1.0434
      ⋮
observed2 = 840×2
    1.0000    1.0443
    1.0000    1.0439
    1.0000    1.0438
    1.0000    1.0438
    1.0000    1.0437
    1.0000    1.0438
    1.0000    1.0434
    1.0000    1.0436
    1.0000    1.0433
    1.0000    1.0434
      ⋮
linreg = 2×1
    0.2407
    0.7624
GOFMatrix = 5×2
         0         0
    0.1853    0.8108
    0.0971    0.9023
    0.2388    0.7591
    0.2407    0.7624
pred = 1×840
    0.9832    0.9814    0.9797    0.9780    0.9763    0.9747    0.9730    0.9715 ⋯
observed = 840×1
    1.0244
    1.0243
```

```
    1.0243
    1.0243
    1.0245
    1.0246
    1.0248
    1.0255
    1.0256
    1.0253
      ⋮
observed2 = 840×2
    1.0000    1.0244
    1.0000    1.0243
    1.0000    1.0243
    1.0000    1.0243
    1.0000    1.0245
    1.0000    1.0246
    1.0000    1.0248
    1.0000    1.0255
    1.0000    1.0256
    1.0000    1.0253
      ⋮
linreg = 2×1
    0.1891
    0.8088
GOFMatrix = 6×2
         0         0
    0.1853    0.8108
    0.0971    0.9023
    0.2388    0.7591
    0.2407    0.7624
    0.1891    0.8088
pred = 1×840
    0.9866    0.9854    0.9841    0.9829    0.9817    0.9806    0.9794    0.9783 ⋯
observed = 840×1
    1.0102
    1.0107
    1.0110
    1.0118
    1.0119
    1.0120
    1.0119
    1.0122
    1.0120
    1.0119
      ⋮
observed2 = 840×2
    1.0000    1.0102
    1.0000    1.0107
    1.0000    1.0110
    1.0000    1.0118
    1.0000    1.0119
    1.0000    1.0120
    1.0000    1.0119
    1.0000    1.0122
    1.0000    1.0120
    1.0000    1.0119
      ⋮
linreg = 2×1
    0.1578
    0.8420
GOFMatrix = 7×2
```

```
         0         0
    0.1853    0.8108
    0.0971    0.9023
    0.2388    0.7591
    0.2407    0.7624
    0.1891    0.8088
    0.1578    0.8420
GOF_GSK = 7×2
         0         0
    0.1853    0.8108
    0.0971    0.9023
    0.2388    0.7591
    0.2407    0.7624
    0.1891    0.8088
    0.1578    0.8420
```

```
% similar to the issue with PlotFits, for GOF, the scaling for S1p is
% data is different since this data was obtained on a different machine.
% We'll just use a different function for now

GOF_s1p = PolyGOF_s1p(s1p_Tys,s1p_Tys_Coeffs,14)
```

```
GOFMatrix = 1×2
     0     0
pred = 1×121
    1.0969    1.1567    1.2108    1.2596    1.3033    1.3423    1.3769    1.4073 ⋯
observed = 121×1
    1.0000
    0.9990
    0.9930
    0.9880
    1.1470
    1.5640
    1.6290
    1.6590
    1.6620
    1.6630
      ⋮
observed2 = 121×2
    1.0000    1.0000
    1.0000    0.9990
    1.0000    0.9930
    1.0000    0.9880
    1.0000    1.1470
    1.0000    1.5640
    1.0000    1.6290
    1.0000    1.6590
    1.0000    1.6620
    1.0000    1.6630
      ⋮
linreg = 2×1
    0.6717
    0.5119
GOFMatrix = 2×2
         0         0
    0.6717    0.5119
pred = 1×121
    1.0758    1.1309    1.1810    1.2265    1.2676    1.3045    1.3375    1.3669 ⋯
observed = 121×1
    1.0000
```

```
    1.0000
    0.9930
    0.9890
    1.1380
    1.5830
    1.6250
    1.5860
    1.5690
    1.5620
      :
      :
observed2 = 121×2
    1.0000    1.0000
    1.0000    1.0000
    1.0000    0.9930
    1.0000    0.9890
    1.0000    1.1380
    1.0000    1.5830
    1.0000    1.6250
    1.0000    1.5860
    1.0000    1.5690
    1.0000    1.5620
      :
      :
linreg = 2×1
    0.6040
    0.5590
GOFMatrix = 3×2
         0         0
    0.6717    0.5119
    0.6040    0.5590
pred = 1×121
    0.9861    0.9935    1.0000    1.0059    1.0112    1.0158    1.0199    1.0234 ···
observed = 121×1
    1.0000
    0.9640
    0.9370
    0.9170
    0.9060
    1.0450
    1.0970
    1.0990
    1.0900
    1.0880
      :
      :
observed2 = 121×2
    1.0000    1.0000
    1.0000    0.9640
    1.0000    0.9370
    1.0000    0.9170
    1.0000    0.9060
    1.0000    1.0450
    1.0000    1.0970
    1.0000    1.0990
    1.0000    1.0900
    1.0000    1.0880
      :
      :
linreg = 2×1
    0.3363
    0.6614
GOFMatrix = 4×2
         0         0
    0.6717    0.5119
```

```
    0.6040    0.5590
    0.3363    0.6614
pred = 1×121
    1.0333    1.1076    1.1754    1.2372    1.2932    1.3438    1.3894    1.4302 ···
observed = 121×1
    1.0000
    0.9990
    0.9960
    0.9940
    1.0230
    1.5690
    1.6520
    1.6470
    1.6680
    1.6630
      ⋮
observed2 = 121×2
    1.0000    1.0000
    1.0000    0.9990
    1.0000    0.9960
    1.0000    0.9940
    1.0000    1.0230
    1.0000    1.5690
    1.0000    1.6520
    1.0000    1.6470
    1.0000    1.6680
    1.0000    1.6630
      ⋮
linreg = 2×1
    0.4705
    0.6833
GOFMatrix = 5×2
         0         0
    0.6717    0.5119
    0.6040    0.5590
    0.3363    0.6614
    0.4705    0.6833
pred = 1×121
    0.8809    0.8510    0.8238    0.7990    0.7767    0.7565    0.7385    0.7224 ···
observed = 121×1
    1.0000
    0.9060
    0.8350
    0.7790
    0.7430
    0.7150
    0.6910
    0.6760
    0.6680
    0.6580
      ⋮
observed2 = 121×2
    1.0000    1.0000
    1.0000    0.9060
    1.0000    0.8350
    1.0000    0.7790
    1.0000    0.7430
    1.0000    0.7150
    1.0000    0.6910
    1.0000    0.6760
    1.0000    0.6680
    1.0000    0.6580
```

```
      ⋮
linreg = 2×1
    0.1502
    0.7789
GOFMatrix = 6×2
         0         0
    0.6717    0.5119
    0.6040    0.5590
    0.3363    0.6614
    0.4705    0.6833
    0.1502    0.7789
pred = 1×121
    1.0815    1.1188    1.1525    1.1827    1.2098    1.2338    1.2551    1.2737 ⋯
observed = 121×1
    1.0000
    1.0070
    1.0030
    0.9990
    1.0940
    1.5280
    1.4940
    1.4460
    1.4160
    1.4050
      ⋮
observed2 = 121×2
    1.0000    1.0000
    1.0000    1.0070
    1.0000    1.0030
    1.0000    0.9990
    1.0000    1.0940
    1.0000    1.5280
    1.0000    1.4940
    1.0000    1.4460
    1.0000    1.4160
    1.0000    1.4050
      ⋮
linreg = 2×1
    0.7820
    0.3824
GOFMatrix = 7×2
         0         0
    0.6717    0.5119
    0.6040    0.5590
    0.3363    0.6614
    0.4705    0.6833
    0.1502    0.7789
    0.7820    0.3824
pred = 1×121
    1.0576    1.0894    1.1182    1.1443    1.1679    1.1890    1.2078    1.2245 ⋯
observed = 121×1
    1.0000
    0.9970
    1.0060
    1.0010
    1.1380
    1.3770
    1.3830
    1.3600
    1.3330
    1.3190
```

```
      ⋮
observed2 = 121×2
    1.0000    1.0000
    1.0000    0.9970
    1.0000    1.0060
    1.0000    1.0010
    1.0000    1.1380
    1.0000    1.3770
    1.0000    1.3830
    1.0000    1.3600
    1.0000    1.3330
    1.0000    1.3190
      ⋮
linreg = 2×1
    0.4345
    0.6346
GOFMatrix = 8×2
         0         0
    0.6717    0.5119
    0.6040    0.5590
    0.3363    0.6614
    0.4705    0.6833
    0.1502    0.7789
    0.7820    0.3824
    0.4345    0.6346
pred = 1×121
    1.0328    1.0619    1.0886    1.1131    1.1353    1.1555    1.1738    1.1902 ⋯
observed = 121×1
    1.0000
    1.0060
    0.9960
    0.9960
    1.0800
    1.3070
    1.3330
    1.2990
    1.2790
    1.2650
      ⋮
observed2 = 121×2
    1.0000    1.0000
    1.0000    1.0060
    1.0000    0.9960
    1.0000    0.9960
    1.0000    1.0800
    1.0000    1.3070
    1.0000    1.3330
    1.0000    1.2990
    1.0000    1.2790
    1.0000    1.2650
      ⋮
linreg = 2×1
    0.5149
    0.5793
GOFMatrix = 9×2
         0         0
    0.6717    0.5119
    0.6040    0.5590
    0.3363    0.6614
    0.4705    0.6833
    0.1502    0.7789
```

```
    0.7820    0.3824
    0.4345    0.6346
    0.5149    0.5793
pred = 1×121
    1.0199    1.0766    1.1282    1.1750    1.2172    1.2550    1.2889    1.3189 ···
observed = 121×1
    1.0000
    0.9970
    1.0010
    1.0100
    1.0550
    1.4020
    1.5290
    1.4920
    1.4530
    1.4440
      :
      :
observed2 = 121×2
    1.0000    1.0000
    1.0000    0.9970
    1.0000    1.0010
    1.0000    1.0100
    1.0000    1.0550
    1.0000    1.4020
    1.0000    1.5290
    1.0000    1.4920
    1.0000    1.4530
    1.0000    1.4440
      :
      :
linreg = 2×1
    0.3994
    0.6966
GOFMatrix = 10×2
         0         0
    0.6717    0.5119
    0.6040    0.5590
    0.3363    0.6614
    0.4705    0.6833
    0.1502    0.7789
    0.7820    0.3824
    0.4345    0.6346
    0.5149    0.5793
    0.3994    0.6966
pred = 1×121
    1.0576    1.1116    1.1607    1.2051    1.2452    1.2811    1.3131    1.3415 ···
observed = 121×1
    1.0000
    0.9980
    1.0040
    1.0050
    1.0470
    1.4620
    1.5620
    1.5220
    1.5050
    1.5290
      :
      :
observed2 = 121×2
    1.0000    1.0000
    1.0000    0.9980
    1.0000    1.0040
    1.0000    1.0050
```

```
    1.0000    1.0470
    1.0000    1.4620
    1.0000    1.5620
    1.0000    1.5220
    1.0000    1.5050
    1.0000    1.5290
      :
      :
linreg = 2×1
    0.5403
    0.5922
GOFMatrix = 11×2
         0         0
    0.6717    0.5119
    0.6040    0.5590
    0.3363    0.6614
    0.4705    0.6833
    0.1502    0.7789
    0.7820    0.3824
    0.4345    0.6346
    0.5149    0.5793
    0.3994    0.6966
      :
      :
pred = 1×121
    1.0641    1.1199    1.1704    1.2158    1.2565    1.2927    1.3248    1.3529 ···
observed = 121×1
    1.0000
    0.9960
    1.0000
    1.0040
    1.1550
    1.4030
    1.5670
    1.5740
    1.5560
    1.5510
      :
      :
observed2 = 121×2
    1.0000    1.0000
    1.0000    0.9960
    1.0000    1.0000
    1.0000    1.0040
    1.0000    1.1550
    1.0000    1.4030
    1.0000    1.5670
    1.0000    1.5740
    1.0000    1.5560
    1.0000    1.5510
      :
      :
linreg = 2×1
    0.5341
    0.5947
GOFMatrix = 12×2
         0         0
    0.6717    0.5119
    0.6040    0.5590
    0.3363    0.6614
    0.4705    0.6833
    0.1502    0.7789
    0.7820    0.3824
    0.4345    0.6346
    0.5149    0.5793
```

```
    0.3994    0.6966
      :
      :
pred = 1×121
    1.0667    1.1371    1.2010    1.2587    1.3106    1.3570    1.3982    1.4346 ···
observed = 121×1
    1.0000
    0.9940
    1.0020
    1.0060
    1.1800
    1.4750
    1.7000
    1.6960
    1.6790
    1.6700
      :
      :
observed2 = 121×2
    1.0000    1.0000
    1.0000    0.9940
    1.0000    1.0020
    1.0000    1.0060
    1.0000    1.1800
    1.0000    1.4750
    1.0000    1.7000
    1.0000    1.6960
    1.0000    1.6790
    1.0000    1.6700
      :
      :
linreg = 2×1
    0.4849
    0.6521
GOFMatrix = 13×2
         0         0
    0.6717    0.5119
    0.6040    0.5590
    0.3363    0.6614
    0.4705    0.6833
    0.1502    0.7789
    0.7820    0.3824
    0.4345    0.6346
    0.5149    0.5793
    0.3994    0.6966
      :
      :
pred = 1×121
    0.8992    1.0039    1.1001    1.1881    1.2685    1.3416    1.4079    1.4677 ···
observed = 121×1
    1.0000
    1.0070
    1.0120
    1.0210
    1.0190
    1.3320
    1.4350
    1.5760
    1.6140
    1.6760
      :
      :
observed2 = 121×2
    1.0000    1.0000
```

```
    1.0000    1.0070
    1.0000    1.0120
    1.0000    1.0210
    1.0000    1.0190
    1.0000    1.3320
    1.0000    1.4350
    1.0000    1.5760
    1.0000    1.6140
    1.0000    1.6760
      ⋮
linreg = 2×1
    0.1101
    0.9271
GOFMatrix = 14×2
         0         0
    0.6717    0.5119
    0.6040    0.5590
    0.3363    0.6614
    0.4705    0.6833
    0.1502    0.7789
    0.7820    0.3824
    0.4345    0.6346
    0.5149    0.5793
    0.3994    0.6966
      ⋮
pred = 1×121
    0.8651    0.9688    1.0640    1.1512    1.2308    1.3033    1.3691    1.4284 ⋯
observed = 121×1
    1.0000
    0.9940
    1.0010
    0.9970
    1.0050
    1.2740
    1.3510
    1.4900
    1.5410
    1.6220
      ⋮
observed2 = 121×2
    1.0000    1.0000
    1.0000    0.9940
    1.0000    1.0010
    1.0000    0.9970
    1.0000    1.0050
    1.0000    1.2740
    1.0000    1.3510
    1.0000    1.4900
    1.0000    1.5410
    1.0000    1.6220
      ⋮
linreg = 2×1
    0.0849
    0.9435
GOFMatrix = 15×2
         0         0
    0.6717    0.5119
    0.6040    0.5590
    0.3363    0.6614
    0.4705    0.6833
    0.1502    0.7789
```

```
    0.7820    0.3824
    0.4345    0.6346
    0.5149    0.5793
    0.3994    0.6966
      ⋮

GOF_s1p = 15×2
         0         0
    0.6717    0.5119
    0.6040    0.5590
    0.3363    0.6614
    0.4705    0.6833
    0.1502    0.7789
    0.7820    0.3824
    0.4345    0.6346
    0.5149    0.5793
    0.3994    0.6966
      ⋮
```

Functions used for this script (code)

This is the code for the functions written for this script.

```
function fig = plotSpline(times,fitted,orig)
hold on
plot(times,fitted)
plot (times, orig)
hold off
end

function fitted = FitPoly(times, cond, order)
p=polyfit(times, cond,order);
fitted=polyval(p,times);
end

function coeff = ReturnCoeff(times,cond,order)
coeff=polyfit(times,cond,order);
end


function figures = plotFits(cond,order,n, ymin,ymax)
times=1:size(cond,1);
times=times/400;
times=times.';
figures=figure;
for i=1:n
    subplot(1,n,i)
    plotSpline(times, FitPoly(times,cond(:,i), order),cond(:,i));
    ylim([ymin,ymax])
end
end

function figures = plotFits2(cond,order,m,n, ymin,ymax)
times=1:size(cond,1);
times=times/400;
times=times.';
```

```
figures=figure;
for i=m:n
    subplot(1,(n-m)+1,(i-m)+1)
    plotSpline(times, FitPoly(times,cond(:,i), order),cond(:,i));
    ylim([ymin,ymax])
end

end

function figures = plotFits_s1p(cond,order,m,n, ymin,ymax)
times=1:size(cond,1);
times=times/12;
times=times.';
figures=figure;
for i=m:n
    subplot(1,(n-m)+1,(i-m)+1)
    plotSpline(times, FitPoly(times,cond(:,i), order),cond(:,i));
    ylim([ymin,ymax])
end

end

function coll = CollectCoefficients(cond, order,n)
coll=zeros(1,order+1);
for i=1:n
  times=1:size(cond,1);
  times=times/400;
coll = cat(1,coll,polyfit(times.', cond(:,i),order));
end
coll = coll(2:end,:);
end

function coll = CollectCoefficients_s1p(cond, order,n)
coll=zeros(1,order+1);
for i=1:n
  times=1:size(cond,1);
  times=times/12;
coll = cat(1,coll,polyfit(times.', cond(:,i),order));
end
coll = coll(2:end,:);
end

function figures = plotPoly(p,times,n, ylim1, ylim2, low_step, high_step, step)
times=times/400;
p_init=p(n);
figure
hold on
for i=low_step:step:high_step
   p(n)=i*p_init;

if i==1 plot(times, polyval(p, times), '--')
else plot(times, polyval(p, times))
end
```

```
ylim([ylim1, ylim2])
end
hold off

end

function rawdata = plotPolyrawdata(p,times,n, ylim1, ylim2, low_step, high_step, step)
times=times/400;
p_init=p(n);
count=1;

for i=low_step:step:high_step
   p(n)=i*p_init;

rawdata(count,:)= polyval(p, times)
count=count+1
end
end

function multiPlotCoeffs(a,b,n)
for i=1:n
    subplot (1,n,i)
    plotCoeffs(a(i,:), b(i,:))
end
end



function figures=plotCoeffs(a,b)
hold on
figures=bar(1:2, [mean(a), mean(b)], 'FaceColor', 'flat');
figures.CData(1,:)=[0,0,0];
figures.CData(2,:)=[0,0.5,0];
errorbar(1:2, [mean(a), mean(b)], [std(a)/sqrt(length(a)), std(b)/sqrt(length(b))], [std(a)/sqr
hold off
end

function figures=plotCoeffs3way(a,b,c)
hold on
figures=bar(1:3, [mean(a), mean(b), mean(c)], 'FaceColor', 'flat');
figures.CData(1,:)=[0,0,0];
figures.CData(2,:)=[0,0.5,0];
figures.CData(3,:)=[0,0,0.5];
errorbar(1:3, [mean(a), mean(b), mean(c)], [std(a)/sqrt(length(a)), std(b)/sqrt(length(b)), std
hold off
end

function multiPlotCoeffs3way(a,b,c,n)
for i=1:n
    subplot (1,n,i)
    plotCoeffs3way(a(i,:), b(i,:), c(i,:));
end
end
```

```
function g = CalcGOF(cond, order)
for i=1:length(cond(1,:))
 times=1:length(cond(:,i));
 times=times/400;
g(i)=goodnessOfFit(cond(:,i),FitPoly(times.', cond(:,i),order),'MSE');
end
end


function GOFMatrix=PolyGOF(rawdata,coeffmatrix,n)
GOFMatrix = zeros(1,2)

for i=1:n
pred=polyval(coeffmatrix(i,:), (1:length(rawdata))/400)
observed=rawdata(:,i)
observed2=[ones(length(observed),1) observed]
linreg=observed2\pred.'
GOFMatrix=cat(1,GOFMatrix,linreg.')
end

end

function GOFMatrix=PolyGOF_s1p(rawdata,coeffmatrix,n)
GOFMatrix = zeros(1,2)

for i=1:n
pred=polyval(coeffmatrix(i,:), (1:length(rawdata))/12)
observed=rawdata(:,i)
observed2=[ones(length(observed),1) observed]
linreg=observed2\pred.'
GOFMatrix=cat(1,GOFMatrix,linreg.')
end

end
```
